# Modular access to functionalized 5–8–5 fused ring systems *via* a photoinduced cycloisomerization reaction†
†Electronic supplementary information (ESI) available: Experimental procedures at characterization data. CCDC 1816383–1816390. For ESI and crystallographic data in CIF or other electronic format see DOI: 10.1039/c8sc00999f


**DOI:** 10.1039/c8sc00999f

**Published:** 2018-05-25

**Authors:** Anna E. Salvati, James A. Law, Josue Liriano, James H. Frederich

**Affiliations:** a Department of Chemistry and Biochemistry , Florida State University , 95 Cheiftan Way , Tallahassee , FL 32306 , USA . Email: frederich@chem.fsu.edu

## Abstract

A 5–8–5 carbocyclic ring system forms the core of over 30 distinct natural products. Several members of this family have gained attention for their diverse activity in cell culture. In these cases, biological function is mediated by the arrangement of substituents around a conserved 5–8–5 nucleus. Despite the potential applications of this privileged substructure in medicinal chemistry, modular strategies for its assembly are underdeveloped. Herein, we describe a cycloisomerization reaction that forms the 5–8–5 framework directly. This strategy uniquely allows access to gram quantities of this valuable scaffold in four steps.

## Abstract 2

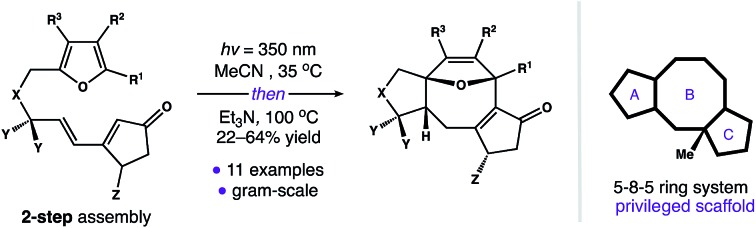
A photoinduced isomerization reaction enables stereocontrolled access to a range of fused 5–8–5 ring systems.

## Introduction

**Scheme 1:**
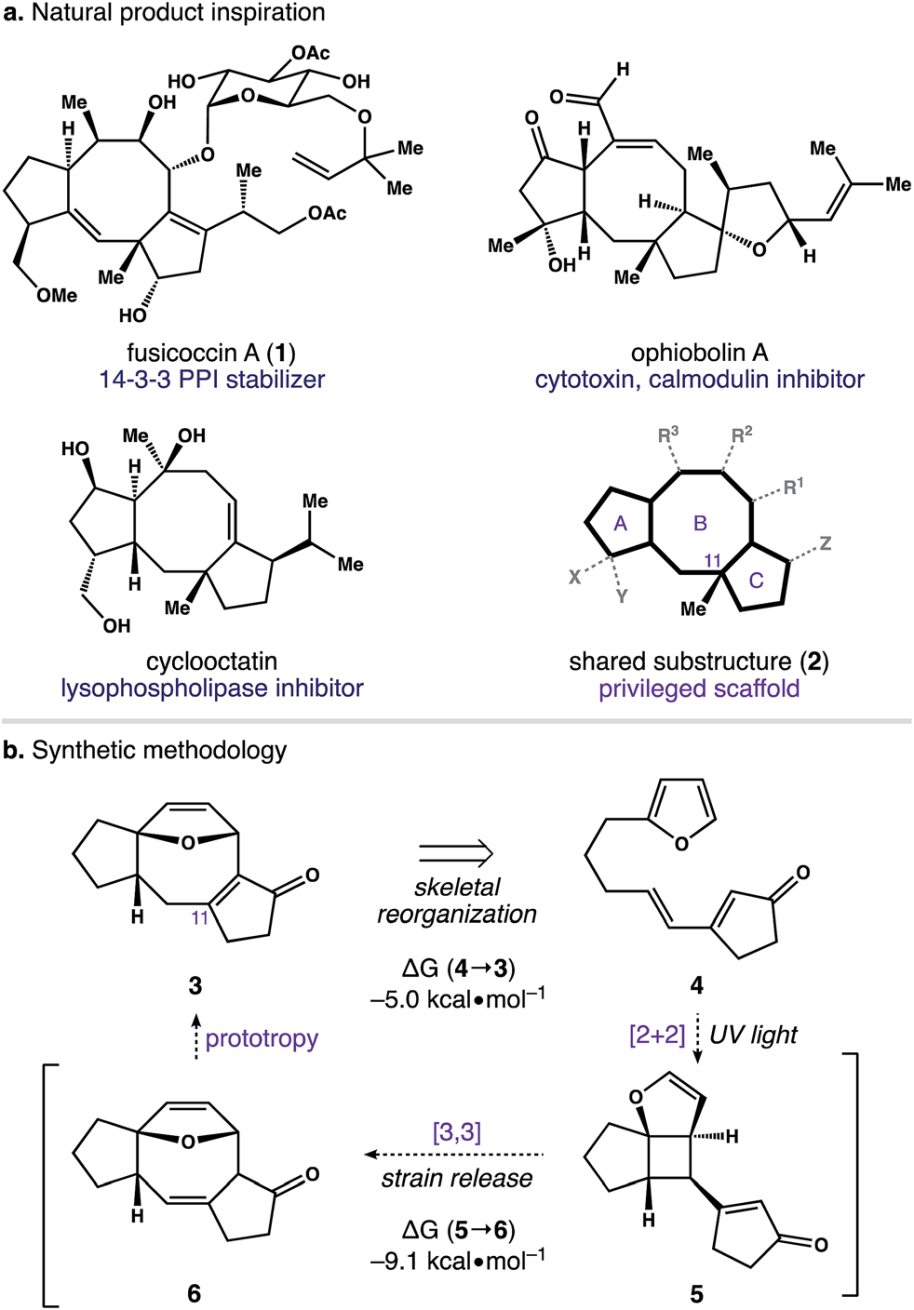
Our design of an efficient and modular entry point to 5–8–5 fused ring systems.

The dicyclopenta[*a*,*d*]cyclooctene (5–8–5) ring system is central to over 30 di- and sesterterpene natural products.^1^ This molecular framework is produced by an array of plants and microorganisms,^2^ and several members of this terpene family possess activity in human cell culture (Scheme 1). For example, fusicoccin A (**1**) functions as an orthosteric stabilizer of 14–3–3 protein–protein interactions (PPIs).^3^,^4^ Similarly, ophiobolin A is a potent cytotoxin that modulates calmodulin activity,^5^ and cyclooctatin inhibits lysophospholipases.^6^,^7^ Intriguingly, the avidity of these natural products for their disparate biological targets is tightly linked to both the identity and arrangement of peripheral groups surrounding a common 5–8–5 core (*i.e.***2**). Taken together, these observations indicate that substructure **2** is a privileged scaffold capable of serving as a ligand for a diverse group of receptors.^8^

Despite the therapeutic potential of molecules possessing a 5–8–5 nucleus, this motif is unrepresented in drug discovery libraries, primarily because of a lack of modular chemistry to prepare such structures.^9^ Several target-specific approaches to 5–8–5 ring systems have emerged from total synthesis.^10^,^11^ In contrast, more general methods to access this chemotype rely on cycloadditions to construct the eight-membered ring.^12^,^13^ Among these, photocycloadditions of pyridones^14^ and pyrones^15^ provide direct access to 5–8–5 scaffolds. However, a major limitation of existing strategies is the need for protracted, multi-step synthesis to prepare the requisite cycloaddition precursors. In addition, many reported methods require high temperatures and/or transition-metal catalysis to promote the desired chemistry. Taken together, these features hinder the widespread application of current cycloaddition approaches to 5–8–5 ring systems and the structural diversity they can achieve.

With an eye toward exploiting the potential pharmacology of substructure **2**, we set out to develop a route to this molecular architecture that would facilitate straightforward diversification of functional groups surrounding the 5–8–5 nucleus. Along these lines, we considered ring system **3** and the possibility of preparing this motif in one step from more readily accessible isomer **4**. A preliminary computational study revealed a thermodynamic driving force of 5.0 kcal mol^–1^ for the net isomerization of **4** to **3**. As such, we envisioned a pathway connecting these isomers initiated by a [2 + 2] photocycloaddition within **4** to give stereodefined cyclobutane **5**. We reasoned that ring strain amassed within this polycyclic system might then be used to promote a mild Cope rearrangement to generate cyclooctadiene **6**.^16^ Subsequent isomerization of **6** to conjugated enone **3** could then terminate the transformation and provide a functional handle to install the key C_11_ quaternary methyl group. Herein, we report the development of this photoinduced cycloisomerization strategy and demonstrate its application as a versatile entry point to scaffold **2**.

## Results and discussion

**Scheme 2:**
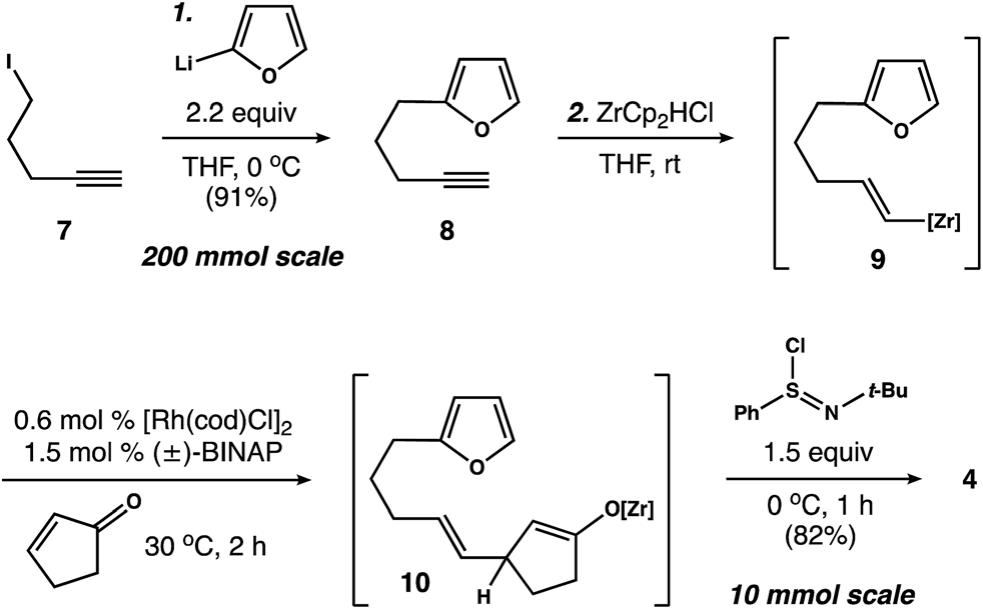
Assembly of photosubstrate **4**.

Our first objective was to develop a concise route to photosubstrate **4** that was flexible and amenable to scale. As shown in Scheme 2, we began by establishing a large-scale protocol for the alkylation of 5-iodopentyne (**7**) with 2-furyllithium to give **8** in 91% yield. Subsequent exposure of **8** to Schwartz's reagent formed vinyl zirconium **9**. This species was then added to a solution of cyclopentenone and catalytic amounts of [Rh(cod)Cl]_2_ and (±)-BINAP in THF.^17^ Warming the resulting solution to 30 °C for 2 h formed zirconium enolate **10**, which was cooled to 0 °C and exposed to *N-tert*-butylbenzenesulfinimidoyl chloride^18^ to afford **4** in 82% yield. This two-operation sequence allowed us to prepare gram quantities of **4** in a single pass. Moreover, the components within this assembly scheme could be readily varied to generate derivatives of **4** for subsequent studies.

**Scheme 3:**
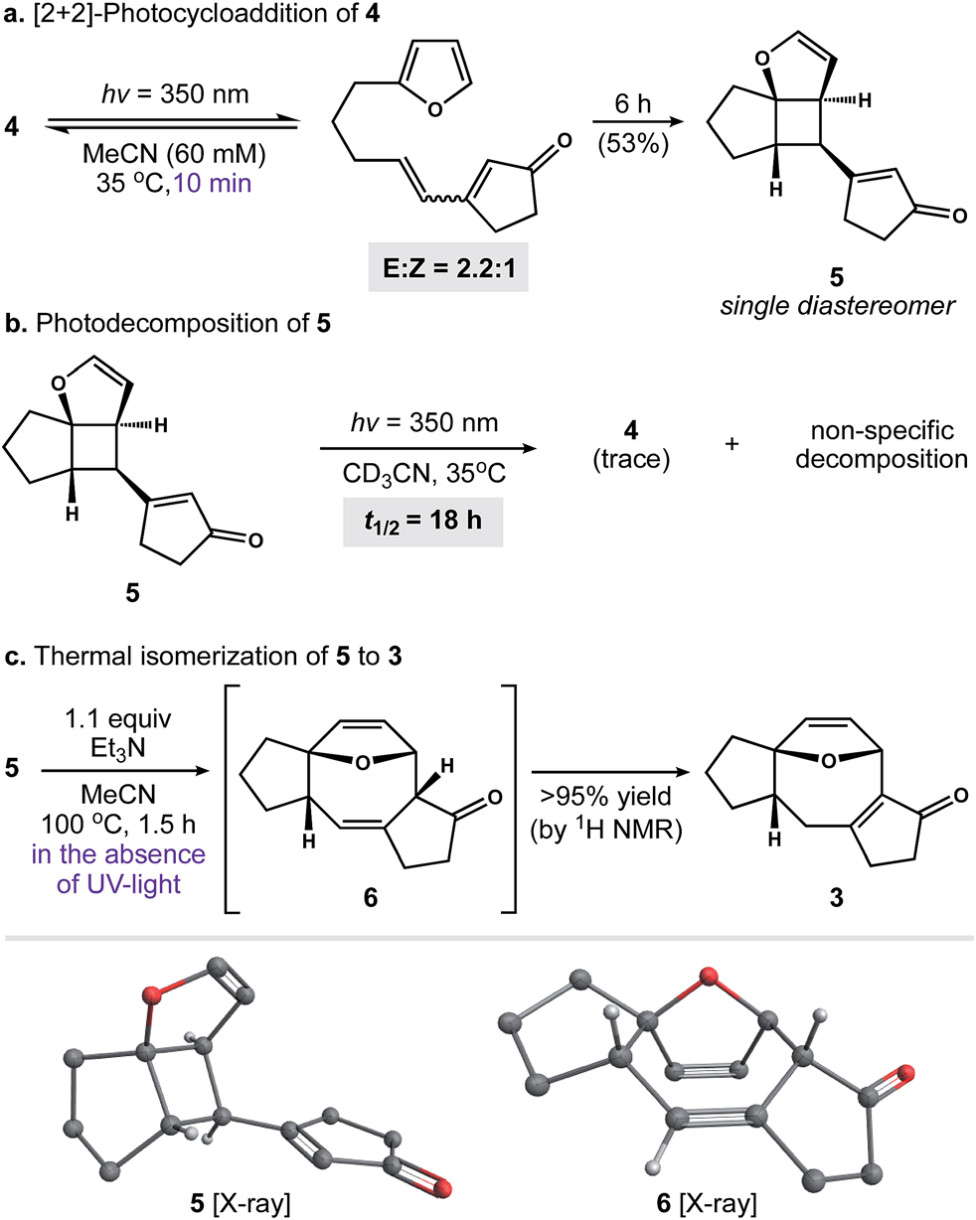
Mechanistic experiments.

With photosubstrate **4** in hand, we turned our attention toward implementing the chemistry shown in Scheme 1b. We quickly found that exposing solutions of **4** to UV-light (*hν* = 350 nm) in a Rayonet photoreactor generated cyclobutane **5**.^19^ After extensive experimentation, we identified MeCN and *n*-BuOH as optimal solvents for the [2 + 2] photocycloaddition. To our delight, irradiation of **4** in MeCN (60 mM, 35 °C) furnished **5** as a single diastereomer (Scheme 3a). This reactive species was isolated as a crystalline solid in 53% yield. The remaining mass balance of the reaction was largely an isomeric mixture of *E*- and *Z*-**4**. Further analysis revealed that UV-light promoted isomerization of the acyclic alkene within **4**. This reaction reached equilibrium within 10 min (*E* : *Z* ratio = 2.2 : 1, see the ESI for details†).^20^ Importantly, increasing the photoreaction time to 12 h markedly increased the extent of decomposition, resulting in a low yield of **5**. Similarly, the addition of triplet sensitizers (*e.g.* benzophenone) to the reaction media had no beneficial effect.

These observations prompted us to examine the stability of **5**. Accordingly, exposure of **5** to optimized photochemical conditions (*hν* = 350 nm, 60 mM CD_3_CN, 35 °C) resulted in slow decomposition and trace cycloreversion to **4** over 24 h (Scheme 3b, *t*_1/2_ = 18 h). Alternatively, when **5** was protected from light and heated above 35 °C we observed gradual formation of cyclooctadiene isomers **6** and **3**. After further experimentation, we found that **5** rearranged cleanly to **6** when heated to 100 °C for 1.5 h.^21^ Subsequent isomerization of **6** to enone **3** occurred slowly under neutral conditions, allowing us to crystallize **6** from toluene. In contrast, exposure of **6** to Et_3_N or silica gel promoted the rapid formation of **3**. Capitalizing on this observation, we found that **5** rearranged to **3** in quantitative yield when heated to 100 °C in the presence of Et_3_N (Scheme 3c).^22^ Taken together, these experiments establish a stereocontrolled mechanism for the cycloisomerization of **4** to fused 5–8–5 carbotricycle **3** by way of cyclobutane **5**.

Our next objective was to develop a procedure to convert **4** to ring system **3** in a single operation. Results from our optimization studies are summarized in Table 1. We began by comparing reactions carried out on 0.5 mmol scale (108 mg of **4**). The best results were achieved using a two-stage protocol, wherein a solution of **4** in MeCN (60 mM) was reacted at 35 °C in the Rayonet. After **12** h, the reaction mixture was moved to the bench-top, treated with Et_3_N (1.1 equiv.), and warmed to 100 °C for 6 h. This procedure afforded **3** in 51% yield along with 26% of unreacted **4** (Table 1, entry 1). We found that *n*-BuOH was a practical alternative for MeCN, providing **3** in 67% yield (entry 2). Importantly, addition of Et_3_N to the reaction was required to suppress formation of side product **11** (entry 3). We speculate that **11** is formed from intermediate **6***via* competitive ring-opening of the dihydrofuran and 6π electrocyclization within the resultant cyclooctatriene.^23^

**Table 1:** Summary of optimization studies

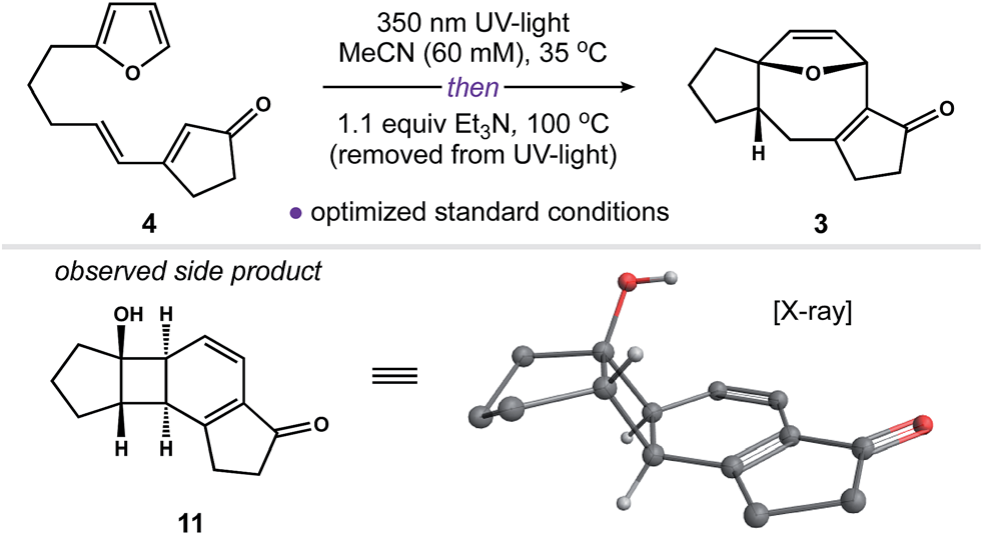				
Entry	Modification^*a*^ ^,^^*b*^ ^,^^*c*^	Time^*d*^ (h)	**3** ^*e*^ (%)	Recovered **4**^*e*^ (%)
1	None	12/6	51	26
2	*n*-BuOH	12/2	67	9
3	*n*-BuOH, no Et_3_N	12/2	50^*f*^	14
4	*hν* at 65 °C	21/0	18	0
5	*hν* at 65 °C, *n*-BuOH	8/0	36	0
6	*hν* at 65 °C with Et_3_N	24/0	24	0
7	None, gram-scale	55/6	61	24
8	*n*-BuOH, gram-scale	34/2	59	22

^*a*^Photochemistry was carried out using 24 W UV-lamps.

^*b*^Reactions performed in a 50 mL quartz flask on 0.5 mmol scale, unless otherwise noted.

^*c*^Gram-scale reactions were carried out a 100 mL quartz flask.

^*d*^Reaction times are reported as follows: time exposed to UV-light/time of conventional heating on the bench-top (see the ESI for a detailed description of reaction step).

^*e*^Isolated yield after purification.

^*f*^Side product **11** was formed in 10–15% yield.

The utility of this two-stage procedure notwithstanding, we also explored conditions to carry out both the [2 + 2] cycloaddition and Cope rearrangement in tandem at 65 °C in the Rayonet (entries 4–6). This modification invariably gave **3** in poor yield. Subsequent control experiments revealed that both **6** and **3** decompose at 65 °C when exposed to UV-light. Alternatively, we found that our two-stage process could be readily scaled without loss of reaction efficiency (entries 7–8). Increased reaction times were required for photoreactions carried out on scale (55 h in MeCN); however, gram quantities of **4** were processed to **3** in 61% yield (85% yield based on recovered **4**) employing these otherwise mild reaction conditions.

**Fig. 1:**
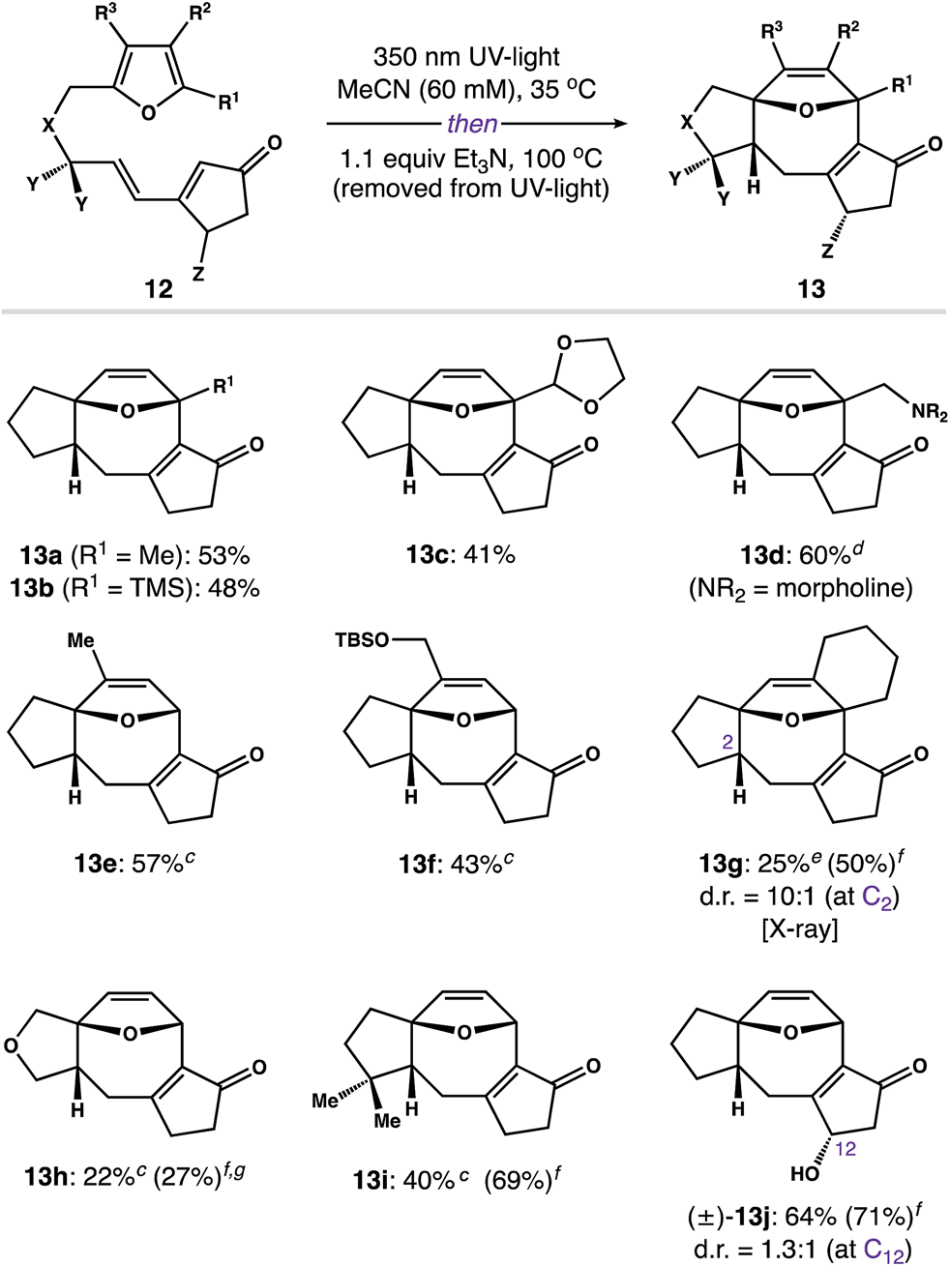
Reaction scope^*a*,*b*^ – ^*a*^Isolated yields of experiments on 0.2–0.5 mmol scale, unless otherwise noted. ^*b*^Reactions afforded **13** as a single diastereomer, unless otherwise noted. ^*c*^*n*-BuOH was used in place of MeCN. ^*d*^Yield of a gram-scale reaction. ^*e*^The C_2_ epimer was isolated in 26% yield and >20 : 1 d.r. when *n*-BuOH was used as the solvent. ^*f*^Yield was determined by ^1^H NMR using *N*-benzylbenzamide as an internal standard. ^*g*^Substrate **12h** required 24 h in the photoreactor to achieve a 27% conversion to the corresponding [2 + 2] photoadduct (see the ESI for details†).

Having developed a scalable route to **3**, we focused on exploring the scope of this process. Utilizing the two-step assembly described in Scheme 2,^24^ we prepared a library of 10 representative photoprecursors **12** designed to probe the scope of substitution patterns around the 5–8–5 core of **2**. As shown in Fig. 1, substrates with modifications to the furan subunit (R^1^–R^3^) reacted smoothly to give B-ring variants **13a–f** in 41–60% yield. In each case, we observed formation of a single diastereomer. The only exception was **12g**, which afforded **13g** in 25% isolated yield (50% yield by NMR) along with small amounts of the corresponding C_2_ diastereomer (d.r. = 10 : 1). Similarly, photosubstrates possessing changes to the hydrocarbon linker (*i.e.* X and Y) gave A-ring variants **13h** and **13i** as single diastereomers in 22% and 40% yield, respectively. In general, these products were challenging to isolate from impurities; however, the photoreaction of substrate **12h** was also notably inefficient, proceeding to only 27% conversion after 24 h. Finally, we examined the isomerization of chiral tether (±)-**12j** (Z = OH), which afforded C-ring derivative (±)-**13j** as a mixture of diastereomers (d.r. = 1.3 : 1 at C_12_) in 64% combined yield.

**Scheme 4:**
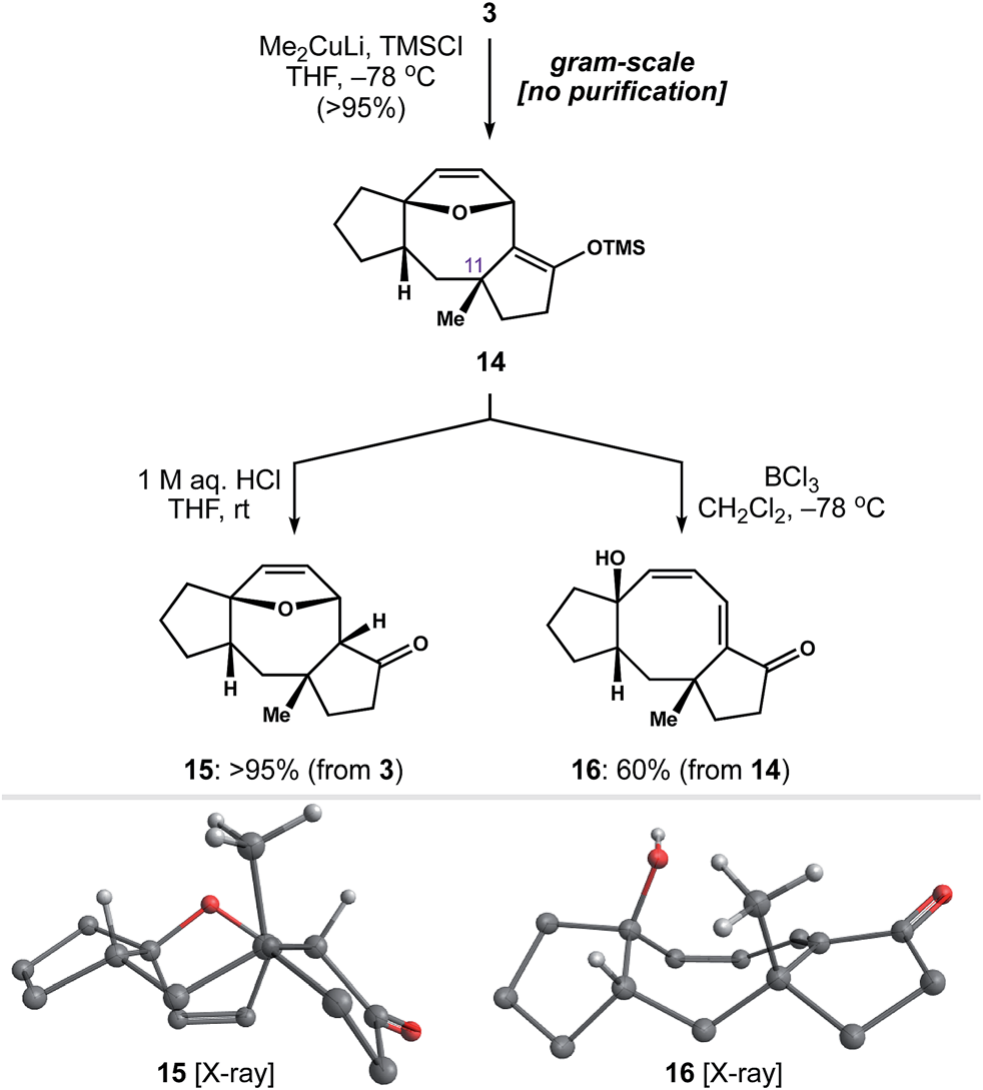
Installation of the C_11_ quaternary methyl group.

With a rapid and general entry to the 5–8–5 ring system in place, we set out to identify conditions to install the quaternary methyl group at C_11_. To our delight, exposure of enone **3** to lithium dimethylcuprate and TMSCl at –78 °C generated silyl enol ether **14** as a single diastereomer (Scheme 4). This reaction was carried out on gram-scale, without the need for chromatography, to afford **14** in >95% yield. Subsequent hydrolysis of **14** with 1 M aq. HCl gave ketone **15** as a crystalline solid, allowing us to establish the relative stereochemistry of 1,4-addition by single-crystal X-ray diffraction.^25^ Alternatively, we found that addition of BCl_3_ to a cooled solution of **14** resulted in ring-opening of the oxabicycle to afford conjugated dienone **16**.^26^ Notably, this chemistry establishes a scalable entry point to variations of **2** in four steps from readily available starting materials.

## Conclusions

In summary, this study describes a modular strategy for the synthesis of 5–8–5 scaffolds. Conceptually, the method described here is appealing because it harnesses strain amassed within stereodefined cyclobutanes **5** to achieve an otherwise challenging ring formation. This approach also enables installation of the C_11_ quaternary stereocenter, and thus, establishes a four-step entry point to the conserved core of multiple bioactive natural products. This chemistry is expected to facilitate structure–function studies of peripheral groups attached to privileged scaffold **2**. Work along these lines is currently underway in our laboratory.

## Conflicts of interest

The authors declare no conflicts of interest.

## Supplementary Material

Supplementary information

Crystal structure data
