# Supplementary material for: Modular access to functionalized 5–8–5 fused ring systems via a photoinduced cycloisomerization reaction
Source: Chem Sci. 2018 May 25;9(24):5389–93. doi: 10.1039/c8sc00999f (PMC6009507; doi:10.1039/c8sc00999f)

Electronic Supplementary Material (ESI) for Chemical Science.
This journal is © The Royal Society of Chemistry 2018

Modular access to functionalized 5-8-5 fused ring systems via a photoinduced cycloisomerization reaction

Anna E. Salvati, James A. Law, Josue Liriano, and James H. Frederich*

Supporting Information

Table of Contents

1. Materials and Methods S1
2. Experimental Procedures and Characterization Data S2–S13
   Scheme S1. Synthesis of 5-furylalkynes **S5** and **S6** S3
   Scheme S2. Preparation of photosubstrate **12i** S7
   Preparation of *N*-*tert*-butylbenzenesulfinimidoyl chloride S13
3. ^{1}H NMR Experiment: Photoequilibration of (*E*)-**4** S14
4. ^{1}H NMR Experiment: Photoreaction of (*Z*)-**4** S15
5. ^{1}H NMR Experiment: Photodecomposition of **5** S16
6. ^{1}H NMR Experiment: Thermal isomerization of **5** to **3** S17
7. ^{1}H NMR Experiment: NMR yields reported in Figure 1 S18–S21
8. Photochemical reaction setup and analysis of quartz vs. pyrex glassware S22–S23
9. SI References S23–S24
10. Copies of ^{1}H and ^{13}C NMR Spectra S25–S108

---

1. Materials and Methods

Unless otherwise stated, reactions were conducted in oven-dried glassware under an atmosphere of molecular nitrogen (N_2) using anhydrous solvents. Tetrahydrofuran (THF), acetonitrile (MeCN), dichloromethane (CH_2Cl_2), diethyl ether (Et_2O), toluene, benzene, and triethylamine (Et_3N) were dried by passage through activated alumina using a solvent purification system. *n*-Butanol was distilled from CaH_2 prior to use. TMSCl was distilled directly before use. *N*-*tert*-butylbenzenesulfinimidoyl chloride was prepared by a modification of a reported procedure[1] and used as a 2 M solution in benzene (see, pg. S13). All other commercial reagents were used as received.

Usually one representative reaction and yield of the product is described in detail; isolated yields reported in Table 1 and Table 2 are the average yields obtained from duplicate experiments. Photochemical reactions were performed using a Rayonet RPR-100 photoreactor equipped with 24 W UV-lamps centered at 350 nm. Reaction temperatures were controlled using a temperature modulator. Column chromatography was conducted on silica gel 60 (240-400 mesh) purchased from VWR. Thin layer chromatography (TLC) was performed using pre-coated, glass-backed plates (SiO_2, 60 PF254, 0.25 mm) and visualized by exposure to UV light (254 nm) or by anisaldehyde, ceric ammonium molybdate, and potassium permanganate staining.

^{1}H NMR spectra were recorded at 400 MHz or 600 MHz and are reported relative to deuterated solvent signals. Data for ^{1}H NMR spectra are reported as follows: chemical shift (δ ppm), multiplicity, coupling constant (Hz), and integration. Splitting patterns are abbreviated as follows: singlet (s), doublet (d), triplet (t), quartet (q), qunitet (quint), multiplet (m), broad (br), apparent (app), and combinations thereof. ^{13}C NMR spectra were recorded at 100 or 150 MHz. Data for ^{13}C NMR spectra are reported in order of carbon multiplicity (C = quaternary, CH = methine, CH_2 = methylene, CH_3 = methyl) and chemical shift. Carbon multiplicity was established by DEPT135 and/or HMQC experiments. Reported melting points of solids are uncorrected. IR spectra were recorded on an FT-IR spectrometer and are reported in terms of frequency (cm^{-1}). Mass spectra were collected on an LCT spectrometer utilizing either electrospray (ESI) or direct analysis in real time (DART) ionization techniques.

1. Experimental Procedures and Characterization Data

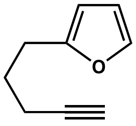

8: $C_9H_{10}O$
MW: 134.18

Large-scale preparation of 5-furylalkyne 8. A two-neck flask equipped with an addition funnel was charged with freshly distilled furan (57 mL, 0.78 mol) and THF (400 mL). The colorless solution was cooled to 0 °C and aged for 1 h. A solution *n*-BuLi in THF (266 mL, 520 mmol, 1.95 M) was then added drop wise via addition funnel over 45 min (**caution: pyrophoric reagent**). After addition, the reaction mixture was maintained at 0 °C during which time a precipitate formed. After 4 h, the resulting slurry was treated with freshly distilled 5-iodopentyne (**7**, 38.8 g, 200 mmol) and the resulting solution was allowed to warm to rt over 12 h. The reaction mixture was cooled to 0 °C and saturated aq. NH_4Cl (200 mL, **caution: exothermic**) was added slowly over 1 h. The resulting emulsion was transferred to a separatory funnel and extracted with Et_2O (3 x 500 mL). The combined organic extracts were washed with brine (1 L), dried over $MgSO_4$, filtered, and concentrated under reduced pressure.[2] The resulting crude oil was purified by distillation (bp = 68–73 °C; 13 torr) to afford **8** (24.6 g, 182 mmol, 91% yield) as a colorless oil: **^{1}H NMR** (400 MHz, $CDCl_3$) δ 7.31 (d, *J* = 1.8, 1H), 6.28 (dd, *J* = 3.1, 1.9, 1H), 6.02 (d, *J* = 3.2, 1H), 2.76 (t, *J* = 7.4, 2H), 2.23 (td, *J* = 7.1, 2.7, 2H), 1.98 (t, *J* = 2.6, 1H), 1.87 (quint, *J* = 7.4, 2H). All other characterization data was identical to previously reported values.[3]

Preparation of 5-furylalkynes S1–S4. *General Procedure.* A solution of freshly purified furan derivative (3.9 equiv) and THF (2 mL/mmol of furan) was cooled to –78 °C. A solution of *n*-BuLi in THF (2.6 equiv) was added drop wise via syringe. After addition, the mixture was warmed to 0 °C and maintained for 4 h. Freshly distilled 5-iodoalkyne (1.0 equiv) was added via syringe at 0 °C and the resulting solution was allowed to warm to rt over 12 h. The reaction was quenched by slow addition of NH_4Cl (**caution: exothermic**). The resulting emulsion was transferred to a separatory funnel and extracted with Et_2O (3 x 2 mL/mmol furan). The combined organic extracts were washed with an equal volume of brine, dried over $MgSO_4$, filtered, and concentrated under reduced pressure. The crude products were purified by distillation or flash chromatography on SiO_2.

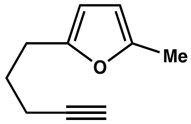

S1: $C_{10}H_{12}O$
MW: 148.21

2-methyl-5-(pent-4-yn-1-yl)furan (**S1**): Following the general procedure, the title compound was synthesized from 2-methylfuran (4.1 mL, 45 mmol) and 5-iodopentyne (3.9 g, 20 mmol). Purification by distillation (bp = 56 °C; 4.7 torr) afforded **S1** (2.5 g, 17 mmol, 85% yield) as a colorless oil: **^{1}H NMR** (400 MHz, $CDCl_3$) δ 5.88 (d, *J* = 2.8, 1H), 5.84 (d, *J* = 2.9, 1H), 2.25 (s, 3H), 2.23 (td, *J* = 7.0, 2.7, 2H), 1.97 (t, *J* = 2.7, 1H), 1.87 (quint, *J* = 7.4, 2H). All other characterization data was identical to previously reported values.[3]

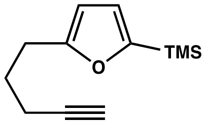

S2: $C_{12}H_{18}OSi$
MW: 206.36

trimethyl(5-(pent-4-yn-1-yl)furan-2-yl)silane (**S2**): Following the general procedure, the title compound was synthesized from furan-2-yltrimethylsilane (3.16 g, 22.5 mmol) and 5-iodopentyne (1.94 g, 10.0 mmol). Purification by flash chromatography (SiO_2; hexanes) afforded **S2** (1.43 g, 6.92 mmol, 69% yield) as a yellow oil: **^{1}H NMR** (400 MHz, $CDCl_3$) δ 6.52 (d, *J* = 3.1, 1H), 6.00 (d, *J* = 3.1, 1H), 2.78 (t, *J* = 7.5, 2H), 2.25 (td, *J* = 7.1, 2.6, 2H), 1.98 (t, *J* = 2.7, 1H), 1.88 (quint, *J* = 7.2, 2H), 0.24 (s, 9H); **^{13}C NMR** (100 MHz, $CDCl_3$) δ C: 159.5, 158.6, 83.9; CH: 120.4, 105.3, 68.7; CH_2: 27.2, 27.0, 18.0; CH_3: 1.5; **IR** (thin film): 2120 cm^{-1}; **HRMS**-DART (m/z) $[M+H]^+$ calculated for $C_{12}H_{19}OSi$ = 207.1200; found 207.1203.

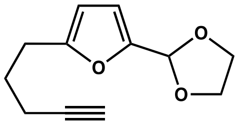

S3: $C_{12}H_{14}O_3$
MW: 206.24

2-(5-(pent-4-yn-1-yl)furan-2-yl)-1,3-dioxolane (**S3**): Following the general procedure, the title compound was synthesized from 2-(furan-2-yl)-1,3-dioxylane (3.15 g, 22.5 mmol) and 5-iodopentyne (1.94 g, 10.0 mmol). Purification by flash chromatography (SiO_2; hexanes) afforded **S3** (1.5 g, 7.3 mmol, 73% yield) as a yellow oil: **^{1}H NMR** (400 MHz, $CDCl_3$) δ 6.34 (d, *J* = 3.2, 1H), 5.99 (d, *J* = 3.2, 1H), 5.86 (s, 1H), 4.09–4.17 (m, 2H), 3.96–4.04 (m, 2H), 2.75 (t, *J* = 7.5, 2H), 2.24 (td, *J* = 7.0, 2.6, 2H), 1.97 (t, *J* = 2.6, 1H), 1.87 (quint, *J* = 7.2, 2H); **^{13}C NMR** (100 MHz, $CDCl_3$) δ C: 156.1, 149.2, 83.7; CH: 109.6, 105.9, 97.8, 68.9; CH_2: 65.1, 26.9, 26.7, 17.9; **IR** (thin film): 2117 cm^{-1}; **HRMS**-ESI (m/z) $[M+H]^+$ calculated for $C_{12}H_{15}O_3$ = 207.1016; found 207.1025.

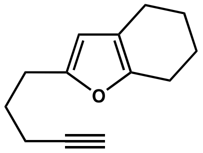

S4: $C_{13}H_{16}O$
MW: 188.27

2-(pent-4-yn-1-yl)-4,5,6,7-tetrahydrobenzofuran (**S4**): Following the general procedure, the title compound was prepared from 4,5,6,7-tetrahydrobenzofuran[4] (2.184 g, 17.9 mmol) and 5-iodopentyne (1.577 g, 8.13 mmol). Purification by flash chromatography (SiO_2; hexanes) afforded **S4** (1.11 g, 5.90 mmol, 73% yield) as a yellow oil: **^{1}H NMR** (400 MHz, $CDCl_3$) δ 5.82 (s, 1H), 2.68 (t, *J* = 7.4, 2H), 2.54 (t, *J* = 5.9, 2H), 2.36 (t, *J* = 6.0, 2H), 2.24 (td, *J* = 7.0, 2.5, 2H), 1.97 (t, *J* = 2.6, 1H), 1.86–1.76 (m, 4H), 1.71–1.68 (m, 2H); **^{13}C NMR** (100 MHz, $CDCl_3$) δ C: 152.7, 149.0, 117.2, 84.0; CH: 106.2, 68.7; CH_2: 27.1, 27.0, 23.2, 23.2, 23.1, 22.1, 17.9; **IR** (thin film): 2118 cm^{-1}; **HRMS**-DART (m/z) $[M+H]^+$ calculated for $C_{13}H_{16}O$ 188.27; found 189.1278.

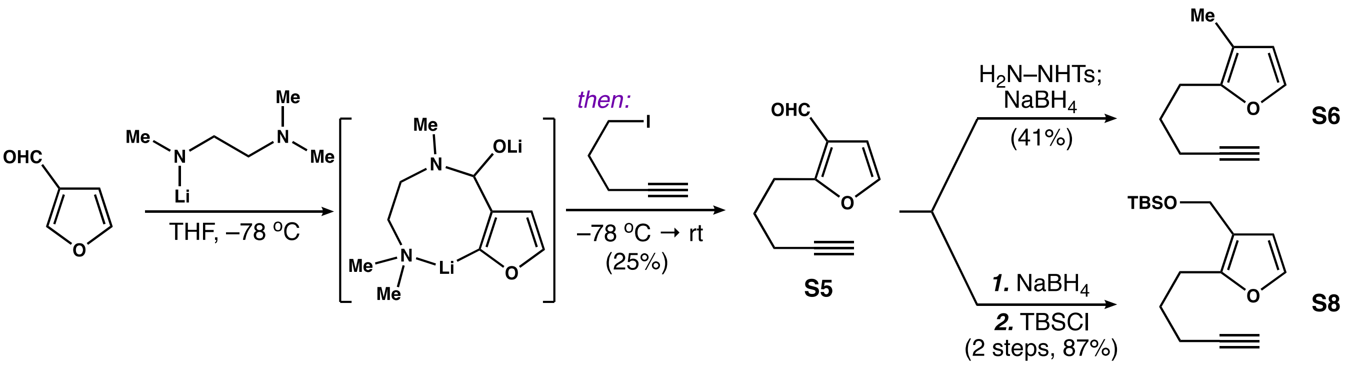

Scheme S1. Synthesis of 5-furylalkynes **S6** and **S8**.

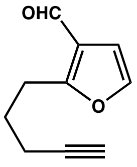

S5: $C_{10}H_{10}O_2$
MW: 162.19

2-(pent-4-yn-1-yl)-furan-3-carbaldehyde (**S5**): Following a modification of the procedure reported by Comins,[5] a solution of *N*,*N*,*N′*-trimethylethylenediamine (4.4 mL, 34 mmol) in THF (110 mL) was treated with a solution of *n*-BuLi in THF (16.9 mL, 33.8 mL, 2.0 M) at –20 °C. The reaction was maintained at –20 °C for 1 h and then 3-furylaldehyde (3.2 mL, 33.8 mmol) was added drop wise via syringe over 10 min. The reaction mixture was aged at –20 °C for an additional 1.5 h and then treated a second equivalent of *n*-BuLi (16.9 mL, 33.8 mmol, 2.0 M in THF). After an additional 1.5 h at –20 °C, 5-iodopentyne (2.91 g, 15.0 mmol) was added via syringe and the resulting solution was allowed to slowly warm to rt over 16 h. The reaction was then treated with saturated aq. NH_4Cl (20 mL). The resulting slurry was transferred to a separatory funnel and extracted with Et_2O (3 x 25 mL). The combined organic extracts were washed with brine (75 mL), dried over $MgSO_4$, filtered, and concentrated under reduced pressure. The resulting crude residue was purified by flash chromatography (SiO_2; 10:1 hexanes/EtOAc) to afford **S5** (616 mg, 3.80 mmol, 25% yield) as a yellow oil: **^{1}H NMR** (400 MHz, $CDCl_3$) δ 9.96 (s, 1H), 7.32 (d, *J* = 2.1, 1H), 6.68 (d, *J* = 2.0, 1H), 3.09 (t, *J* = 7.3, 2H), 2.23 (td, *J* = 6.8, 2.6, 2H), 2.01 (t, *J* = 2.7, 1H), 1.94 (quint, *J* = 7.1, 2H); **^{13}C NMR** (100 MHz, $CDCl_3$) δ C: 164.6, 123.1, 83.0; CH: 184.9, 142.4, 108.1, 69.7; CH_2: 26.8, 25.5, 17.8; **IR** (thin film): 2118, 1676, cm^{-1}; **HRMS**-ESI (m/z) $[M+H]^+$ calculated for $C_{10}H_{11}O_2$ = 163.0754; found 163.0757.

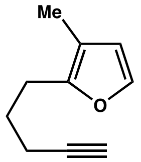

S6: $C_{10}H_{12}O$
MW: 148.21

3-methyl-2-(pent-4-yn-1-yl)furan (**S6**): A solution of **S5** (159 mg, 0.98 mmol) and benzenesulfonyl hydrazide (201 mg, 1.08 mmol) in MeOH (5 mL) was maintained at rt. After 3 h, the reaction mixture concentrated under reduced pressure. The resulting hydrazone (193 mg, ca. 90% purity) was used directly without further purification.

$NaBH_4$ (80 mg, 2.1 mmol) was added in a single portion to a solution of the hydrazone prepared above in AcOH (3 mL) at rt. The reaction mixture was warmed to 40 °C. After 3 h, the reaction was cooled to rt and then diluted with hexanes (25 mL). The mixture was transferred to a separatory funnel and washed with saturated aq. $NaHCO_3$ (2 x 20 mL) and brine (20 mL). The organic layer was dried over $MgSO_4$, filtered, and concentrated under reduced pressure. The resulting residue was purified by flash chromatography (SiO_2; hexanes) to afford **S6** (83 mg, 0.4 mmol, 41% yield) as a colorless oil: **^{1}H NMR** (400 MHz, $CDCl_3$) δ 7.22 (d, *J* = 1.8, 1H), 6.16 (d, *J* = 1.8, 1H), 2.69 (t, *J* = 7.2, 2H), 2.18 (td, *J* = 7.0, 2.7, 2H), 1.97–1.98 (m, 4H), 1.83 (quint, *J* = 7.1, 2H); **^{13}C NMR** (150 MHz, $CDCl_3$) δ C: 150.1, 114.5, 84.1; CH: 140.0, 112.7, 68.6; CH_2: 27.2, 24.6, 17.7; CH_3: 9.8; **IR** (thin film): 2923, 2853 cm^{-1}; **HRMS**-DART (m/z) $[M+H]^+$ calculated for $C_{10}H_{13}O$ = 149.0961; found 149.0967.

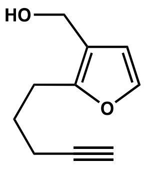

S7: $C_{10}H_{12}O_2$
MW: 164.20

(2-(pent-4-yn-1-yl)furan-3-yl)methanol (**S4**): $NaBH_4$ (110 mg, 5.60 mmol) was added in a single portion to a rapidly stirred slurry of **S3** (300 mg, 1.80 mmol) in a 9:1 mixture of Et_2O/H_2O (2 mL) at rt. After 3 h, and additional equivalent of $NaBH_4$ (68 mg, 1.8 mmol) was added and stirring was continued for an additional 2 h. The reaction was treated with H_2O (15 mL), transferred to a separatory funnel, and extracted with Et_2O (3 x 25 mL). The combined organic extracts were dried over $MgSO_4$, filtered, and concentrated. The resulting crude residue was purified by flash chromatography (SiO_2; 4:1 hexanes/EtOAc) to afford **S8** (300 mg, 1.80 mmol, >95% yield) as a colorless oil: **^{1}H NMR** (400 MHz, $CDCl_3$) δ 7.28 (d, J = 1.9, 1H), 6.38 (d, J = 1.9, 1H), 4.50 (s, 2H), 2.79 (t, J = 7.1, 2H), 2.17 (td, J = 6.8, 2.6, 2H), 2.01 (t, J = 2.7, 1H), 1.86 (quint, J = 7.1, 2H), 1.49 (br, 1H); **^{13}C NMR** (100 MHz, $CDCl_3$) δ C: 151.7, 119.8, 83.8; CH: 141.0, 110.9, 69.1; CH_2: 56.4, 26.9, 24.5, 17.5; **IR** (thin film): 3295, 2117 cm^{-1} **HRMS**-DART (m/z) $[M+NH_4]^+$ calculated for $C_{10}H_{16}NO_2$ = 182.1176; found 182.1183.

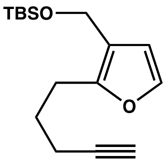

S8: $C_{16}H_{26}O_2Si$
MW: 278.47

tert-butyldimethyl((2-(pent-4-yn-1-yl)furan-3-yl)methoxy)silane (**S8**): A solution of **S5** (300 mg, 1.82 mmol) and imidazole (620 mg, 3.69 mmol) in CH_2Cl_2 (4 mL) was cooled to °C and treated with a solution of TBSCl (390 mg, 2.58 mmol) in CH_2Cl_2 (4 mL). The reaction mixture was warmed to rt and maintained for 30 min. The reaction was then treated with H_2O (10 mL). The resulting slurry was transferred to a separatory funnel and extracted with CH_2Cl_2 (3 x 10 mL). The combined organic extracts were dried over $MgSO_4$, filtered, and concentrated under reduced pressure. The resulting crude residue was purified by flash chromatography (SiO_2; hexanes) to afford **S8** (437 mg, 1.57 mmol, 87% yield) as a yellow oil: **^{1}H NMR** (400 MHz, $CDCl_3$) δ 7.25 (d, J = 1.9, 1H), 6.32 (d, J = 1.9, 1H), 4.52 (s, 2H), 2.74 (t, J = 7.3, 2H), 2.19 (td, J = 7.0, 2.7, 2H), 1.97 (t, J = 2.6, 1H), 1.84 (quint, J = 7.1, 2H), 0.91 (s, 9H), 0.08 (s, 6H). **^{13}C NMR** (150 MHz, $CDCl_3$) δ C: 150.7, 119.8, 83.9, 18.4; CH: 140.5, 110.9, 68.7; CH_2: 57.1, 27.2, 25.0, 17.8; CH_3: 26.0, –5.2; **IR** (thin film): 2121 cm^{-1}; **HRMS**-DART (m/z) $[M+NH_4]^+$ calculated for $C_{16}H_{30}NO_2Si$ = 296.2040; found 296.2050.

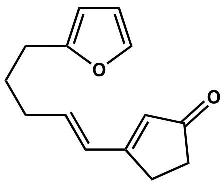

4: $C_{14}H_{16}O_2$
MW: 216.28

Synthesis of photosubstrates 4, 12a–c, 12e–h, 12j and 13–15. *Representative Procedure for* (*E*)-3-(5-(furan-2-yl)pent-1-en-1-yl)cyclopent-2-en-1-one (**4**): A solution of $[Rh(cod)Cl]_2$ (31 mg, 0.06 mmol) and (±)-BINAP (96 mg, 0.15 mmol) in THF (10 mL) was stirred for 1 h at rt. In parallel, a suspension of Cp_2ZrHCl (3.40 g, 13.2 mmol) in THF (20 mL) was treated with **8** (1.62 g, 12.0 mmol) and stirred for 1 h at rt. Freshly distilled cyclopentenone (0.84 mL, 10 mmol) was added to the catalyst mixture via syringe, followed by the alkenylzirconocene solution. The reaction mixture was warmed to 30 °C and maintained until cyclopentenone was fully consumed by TLC (R_f = 0.2; 4:1 hexanes/EtOAc). After 3 h, the dark reaction mixture was cooled to 0 °C and a solution of *N*-*tert*-butylphenylsulfinimidoyl chloride in PhH (5.7 mL, 11.3 mmol, 2 M) was added rapidly as a stream via syringe. After 1 h, the reaction mixture was poured into a solution of 1:1 hexanes/Et_2O (150 mL) and stirred for 15 min at rt. The resulting precipitate was removed by filtration and the crude filtrate was concentrated under reduced pressure. The resulting crude residue was purified by flash chromatography (SiO_2; 97:3 CH_2Cl_2/Et_2O) to afford **4** (1.77 g, 8.18 mmol, 82% yield) as a yellow oil: **^{1}H NMR** (400 MHz, $CDCl_3$) δ 7.29–7.28 (m, 1H), 6.55 (d, J = 15.8, 1H), 6.30 (dt, J = 15.7, 7.0, 1H), 6.28–6.27 (m, 1H), 6.00 (d, J = 3.1, 1H), 5.96 (s, 1H), 2.72 (m, 2H), 2.67 (t, J = 7.4, 2H), 2.45 (m, 2H), 2.28 (q, J = 7.2, 2H), 1.83 (quint, J = 7.5, 2H) **^{13}C NMR** (100 MHz, $CDCl_3$) δ C: 209.6, 172.6, 155.4; CH: 140.9, 140.1, 129.3, 127.1, 110.1, 105.2; CH_2: 34.8, 32.5, 27.4, 27.1, 27.0; **IR** (thin film): 1698, 1673, 1578, 1507 cm^{-1}; **HRMS**-ESI (m/z) $[M+Na]^+$ calculated for $C_{14}H_{16}O_2Na$ = 239.1048; found 239.1054.

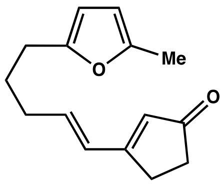

12a: $C_{15}H_{18}O_2$
MW: 230.31

Photosubstrate **12a**: Prepared following the procedure reported for **4** employing alkyne **S1** (298 mg, 2.40 mmol) and cyclopentenone (0.2 mL, 2.0 mmol). The crude reaction mixture was purified by flash chromatography (SiO_2; 97:3→47:3 CH_2Cl_2/Et_2O) to afford **12a** (203 mg, 0.88 mmol, 44% yield) as a yellow oil: **^{1}H NMR** (400 MHz, $CDCl_3$) δ 6.54 (d, J = 15.8, 1H), 6.31 (dt, J = 15.7, 7.0, 1H), 5.96 (s, 1H), 5.87–5.84 (m, 2H), 2.73–2.71 (m, 2H), 2.61 (t, J = 7.4, 2H), 2.46–2.43 (m, 2H), 2.29 (q, J = 7.8, 2H), 2.25 (s, 3H), 1.80 (quint, J = 7.6, 2H); **^{13}C NMR** (100 MHz, $CDCl_3$) δ C: 209.5, 172.6, 153.5, 150.3; CH: 140.3, 129.2, 127.0, 105.8, 105.7; CH_2: 34.8, 32.5, 27.5, 27.2, 27.0; CH_3: 13.5; **IR** (thin film): 1699, 1673, 1637, 1576 cm^{-1}; **HRMS**-ESI (m/z) $[M+Na]^+$ calculated for $C_{15}H_{18}O_2Na$= 253.1205; found 253.1206.

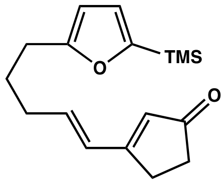

12b: $C_{17}H_{24}O_2Si$
MW: 288.46

Photosubstrate **12b**: Prepared following the procedure reported for **4** employing alkyne **S2** (495 mg, 2.40 mmol) and cyclopentenone (0.2 mL, 2.0 mmol).. The crude reaction mixture was purified by flash chromatography (SiO_2; 97:3→47:3 CH_2Cl_2/Et_2O) to afford **12b** (323 mg, 1.12 mmol, 56% yield) as a yellow oil: **^{1}H NMR** (400 MHz, $CDCl_3$) δ 6.57–6.52 (m, 2H), 6.31 (dt, *J* = 15.7, 7.0, 1H), 5.98 (d, *J* = 3.1, 1H), 5.96 (s, 1H), 2.74–2.68 (m, 4H), 2.46–2.43 (m, 2H), 2.30 (q, *J* = 7.4, 2H), 1.83 (quint, *J* = 7.5, 2H), 0.24 (s, 9H); **^{13}C NMR** (100 MHz, $CDCl_3$) δ C: 209.1, 172.4, 159.7, 158.3; CH: 140.1, 129.1, 127.0, 120.5, 105.2; CH_2: 34.7, 32.5, 27.5, 27.0, 26.9; CH_3: –1.5; **IR** (thin film): 2955, 1701, 1673, 1438 cm^{-1}; **HRMS**-ESI (m/z) $[M+H]^+$ calculated for $C_{17}H_{25}SiO_2$ = 289.1624; found 289.1617.

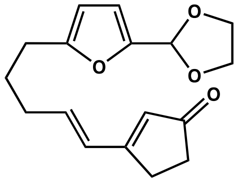

12c: $C_{17}H_{20}O_4$
MW: 288.34

Photosubstrate **12c**: Prepared following the procedure reported for **4** employing alkyne **S3** (495 mg, 2.40 mmol) and cyclopentenone (0.2 mL, 2.0 mmol). The crude reaction mixture was purified by flash chromatography (SiO_2; 97:3→47:3 CH_2Cl_2/Et_2O) to afford **12c** (332 mg, 1.15 mmol, 58% yield) as a yellow oil: : **^{1}H NMR** (400 MHz, $CDCl_3$) δ 6.55 (d, *J* = 15.7, 1H), 6.35 (d, *J* = 3.2, 1H), 6.30 (dt, *J* = 15.4, 7.0, 1H), 5.98–5.96 (m, 2H), 5.85 (s, 1H), 4.17–4.09 (m, 2H), 4.04–3.96 (m, 2H), 2.73–2.71 (m, 2H), 2.67 (t, *J* = 7.4, 2H), 2.46–2.43 (m, 2H), 2.29 (q, *J* = 7.0, 2H), 1.83 (quint, *J* = 7.7, 2H); **^{13}C NMR** (100 MHz, $CDCl_3$) δ C: 209.1 172.4, 156.2, 149.2; CH: 139.9, 129.0, 127.0, 109.4, 105.7, 97.7; CH_2: 64.9, 34.6, 32.3, 27.3, 26.9, 26.7; **IR** (thin film): 1693, 1673, 1473, 1096 cm^{-1}; **HRMS**-ESI (m/z) $[M+H]^+$ calculated for $C_{17}H_{21}O_4Na$ = 289.1434; found 289.1438.

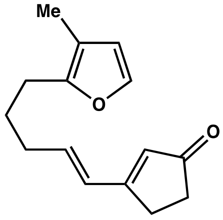

12e: $C_{15}H_{18}O_2$
MW: 230.31

Photosubstrate **12e**: **^{1}H NMR** (400 MHz, $CDCl_3$) δ 7.22 (d, *J* = 1.8, 1H), 6.53 (d, *J* = 15.8, 1H), 6.28 (dt, *J* = 15.7, 6.9, 1H), 6.16 (d, *J* = 1.8, 1H), 5.95 (s, 1H), 2.72 (dd, *J* = 4.9, 3.2, 2H), 2.60 (t, *J* = 7.2, 2H), 2.46–2.43 (m, 2H), 2.24 (q, *J* = 6.9, 2H), 1.95 (s, 3H), 1.80 (quint, *J* = 7.3, 2H); **^{13}C NMR** (150 MHz, $CDCl_3$) δ C: 209.6, 172.6, 150.4, 114.2; CH: 140.3, 139.9, 129.2, 126.9, 112.8; CH_2: 34.8, 32.5, 27.3, 27.1, 25.3; CH_3: 9.8; **IR** (thin film): 1702 cm^{-1}; **HRMS**-ESI (m/z) $[M+Na]^+$ calculated for $C_{14}H_{16}O_2Na$ = 253.1205; found 253.1201.

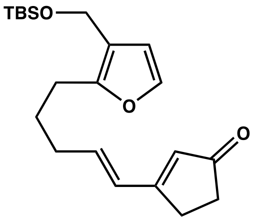

12f: $C_{21}H_{32}O_3Si$
MW: 360.57

Photosubstrate **12f**: Prepared following the procedure reported for **4** employing alkyne **S8** (334 mg, 1.20 mmol) and cyclopentenone (0.1 mL, 1.0 mmol). The crude reaction mixture was purified by flash chromatography (SiO_2; 97:3 CH_2Cl_2/Et_2O) to afford **12f** (120 mg, 0.33 mmol, 33% yield) as a yellow oil: : **^{1}H NMR** (400 MHz, $CDCl_3$) δ 7.24 (d, *J* = 1.8, 1H), 6.53 (d, *J* = 15.8, 1H), 6.32–6.24 (m, 2H), 5.95 (s, 1H), 4.49 (s, 2H), 2.72 (dd, *J* = 4.9, 3.4, 2H), 2.66 (t, *J* = 7.3, 2H), 2.45–2.43 (m, 2H), 2.25 (q, *J* = 7.0, 2H), 1.81 (quint, *J* = 7.0, 2H) 0.90 (s, 9H), 0.07 (s, 6H); **^{13}C NMR** (100 MHz, $CDCl_3$) δ C: 209.5, 172.5, 151.2, 119.4, 18.3; CH: 140.3, 140.2, 129.2, 126.9, 110.9; CH_2: 57.1, 34.7, 32.5, 27.4, 27.0, 25.7; CH_3: 25.9, –5.2; **IR** (thin film): 2929, 1702, 1672, 1079 cm^{-1}; **HRMS**-ESI (m/z) $[M+Na]^+$ $C_{21}H_{32}SiO_3$ = 383.2018; found 383.2014.

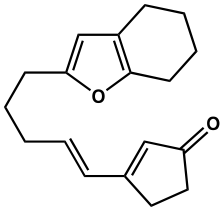

12g: $C_{18}H_{22}O_2$
MW: 270.37

Photosubstrate **12g**: Prepared following the procedure reported for **4** employing alkyne **S4** (323 mg, 1.72 mmol) and cyclopentenone (0.1 mL, 1.4 mmol). The crude reaction mixture was purified by flash chromatography (SiO_2; 97:3 CH_2Cl_2/Et_2O) to afford **12g** (174 mg, 0.644 mmol, 45% yield) as an orange oil: **^{1}H NMR** (400 MHz, $CDCl_3$) δ 6.54 (d, *J* = 15.8, 1H), 6.30 (dt, *J* = 15.7, 6.9, 1H), 5.96 (s, 1H), 5.80 (s, 1H), 2.73–2.71 (m, 2H), 2.60 (t, *J* = 7.4, 2H), 2.53 (t, *J*= 5.9, 2H), 2.46–2.43 (m, 2H), 2.38–2.35 (m, 2H), 2.29 (q, *J*= 7.1, 2H), 1.84–1.76 (m, 4H), 1.72–1.66 (m, 2H); **^{13}C NMR** (100 MHz, $CDCl_3$) δ C: 209.6, 172.6, 152.9, 148.9, 117.2; CH: 140.4, 129.2, 126.9, 106.1; CH_2: 34.8, 32.6, 27.6, 27.3, 27.0, 23.2, 23.2, 23.1, 22.1; **IR** (thin film): 1701, 1674, 1639, 1578 cm^{-1}; **HRMS**-ESI (m/z) $[M+Na]^+$ calculated for $C_{18}H_{22}O_2Na$ = 293.1518; found 293.1511.

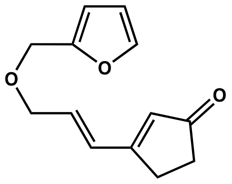

12h: $C_{13}H_{14}O_3$
MW: 218.25

Photosubstrate **12h**: Prepared following the procedure reported for **4** employing 2-((prop-2-yn-1-yloxy)methylfuran[6] (329 mg, 2.40 mmol) and cyclopentenone (0.2 mL, 2.0 mmol). The crude reaction mixture was purified by flash chromatography (SiO_2; 1:1 CH_2Cl_2/Hex → 95:5 hexanes/Et_2O) to afford **12h** (176 mg, 0.78 mmol, 39% yield) as a yellow foam: **^{1}H NMR** (400 MHz, $CDCl_3$) δ 7.42 (s, 1H), 6.75 (d, *J* = 15.9, 1H), 6.34–6.28 (m, 2H), 6.03 (s, 1H), 4.51 (s, 2H), 4.19 (dd, *J* = 1.2, 5.2, 2H), 2.74–2.72 (m, 2H), 2.46–2.44 (m, 2H); **^{13}C NMR** (100 MHz, $CDCl_3$) δ C: 209.3, 171.4, 151.2; CH: 142.9, 135.3, 130.5, 126.9, 110.3, 109.6; CH_2: 69.4, 64.4, 34.7, 26.9; **IR** (thin film): 2933, 1665, 1252, 998 cm^{-1}; **HRMS**-ESI (m/z) $[M+Na]^+$ $C_{13}H_{14}O_3$ = 219.1016; found 219.1020.

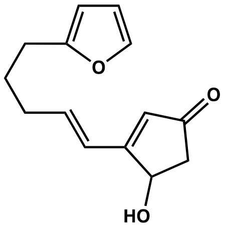

12j: $C_{14}H_{16}O_3$
MW: 232.28

Photosubstrate **12j**: Prepared in two steps. Following the procedure reported for **4**, alkyne **8** (177 mg, 1.31 mmol) was coupled with 4[(*tert*-butyldimethylsilyl)oxy]cyclopent-2-enone[7] (231 mg, 1.09 mmol). The crude reaction mixture was used directly in the subsequent step. An analytical sample of TBS-protected **12j** was prepared for characterization: **^{1}H NMR** (400 MHz, $CDCl_3$) δ 7.30 (s, 1H), 6.50 (dt, *J* = 22.8, 7.0, 1H), 6.37–6.25 (m, 2H), 6.03–5.96 (m, 2H), 5.04 (d, *J* = 4.5, 1H), 2.75 (dd, *J* = 18.0, 6.2, 2H), 2.66 (t, *J* = 7.4, 2H), 2.38–2.24 (m, 3H), 1.82 (pent, *J* = 7.4, 2H), 0.90 (s, 9H), 0.14 (d, *J* = 17.6, 6H); **^{13}C NMR** (100 MHz, $CDCl_3$) δ C: 205.1, 171.9, 155.4, 119.4, 18.3; CH: 140.3, 140.2, 129.2, 126.9, 110.9; CH_2: 57.1, 34.7, 32.5, 27.4, 27.0, 25.7; CH_3: 25.9, –5.2; **HRMS**-ESI (m/z) $[M+Na]^+$ $C_{20}H_{30}SiO_3$ = 369.18619; found 369.18559.

A solution of the unpurified intermediate prepared above in THF (5 mL) was cooled to 0 °C and treated with tetrabutylammonium fluoride (2.3 mmol, 2.3 mL, 1.0 M in THF). The reaction mixture was maintained at 0 °C for 5 min and then treated with water (15 mL). The resulting slurry was extracted with EtOAc (3 x 15 mL). The combined organic extracts were washed with brine (10 mL), dried over $MgSO_4$, filtered, and concentrated under reduced pressure. The resulting crude residue was purified by flash chromatography (SiO_2; 2:1 hexanes/EtOAc) to afford **12j** (371 mg, 1.60 mmol, 70% yield overall) as a yellow oil: **^{1}H NMR** (400 MHz, $CDCl_3$) δ 7.30 (d, *J* = 1.0, 1H), 6.60 (dt, *J* = 22.8, 7.0, 1H), 6.42 (d, *J* = 16.0, 1H), 6.29 (t, *J* = 2.0, 1H), 6.00–5.99 (m, 2H), 5.12 (t, *J* = 6.0, 1H), 2.84 (t, *J* = 7.4, 2H), 2.40–2.29 (m, 3H), 1.85 (quint, *J* = 7.2, 2H); **^{13}C NMR** (100 MHz, $CDCl_3$) δ C: 205.8, 172.3, 155.4; CH: 143.4, 129.1, 124.4, 110.2, 105.2, 69.6; CH_2: 49.5, 32.9, 27.4, 26.9; **IR** (thin film): 3388, 2931, 1678, 1639, 1434 cm^{-1}; **HRMS**-ESI (m/z) $[M+Na]^+$ calculated for $C_{14}H_{16}O_3Na$ = 255.09971; found 255.10023.

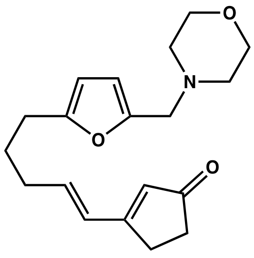

12d: $C_{19}H_{25}NO_3$
MW: 315.41

Photosubstrate **12d**: Following a modification of the procedure reported by Heaney,[8] a solution of **4** (541 mg, 2.50 mmol) in THF (3 mL) was added to a stirred suspension of *N*-(methylene)morpholinium chloride (229 mg, 2.50 mmol) in THF (5 mL). The resulting slurry was stirred at rt. After 16 h, the reaction mixture was transferred to a separatory funnel and partitioned between Et_2O (12 mL) and saturated aq. $NaHCO_3$ (35 mL). The organic layer was separated and the aqueous layer was extracted with Et_2O (4 x 10 mL). The combined organic layers were washed with water (10 mL) and brine (10 mL), dried over $MgSO_4$, filtered, and concentrated under reduced pressure. The resulting crude residue was purified by flash chromatography (SiO_2; 98:2 → 90:10 Et_2O/MeOH) to afford **12d** (724 mg, 2.27 mmol, 91% yield) as a yellow oil: **^{1}H NMR** (400 MHz, $CDCl_3$) δ 6.51 (d, *J* = 15.7, 1H), 6.31–6.24 (m, 1H), 6.08 (d, *J* = 3.0, 1H), 5.93 (s, 1H), 5.89 (d, *J* = 3.0, 1H), 3.69 (t, *J* = 4.48, 4H), 3.46 (s, 2H), 2.71–2.69 (m, 2H), 2.62 (t, *J* = 7.5, 2H), 2.44–2.40 (m, 6H), 2.26 (q, *J* = 7.3, 2H), 1.78 (quint, *J* = 7.4, 2H); **^{13}C NMR** (100 MHz, $CDCl_3$) δ C: 209.5, 172.4, 155.4, 149.4; CH: 140.0, 129.3, 127.2, 109.8, 105.7; CH_2: 66.8, 55.4, 53.2, 34.8, 32.5, 27.6, 27.0; **IR** (thin film): 2858, 1701, 1670, 1451 cm^{-1}; **HRMS**-ESI (m/z) $[M+Na]^+$ calculated for $C_{19}H_{25}NO_3Na$ = 338.1732; found 338.1739.

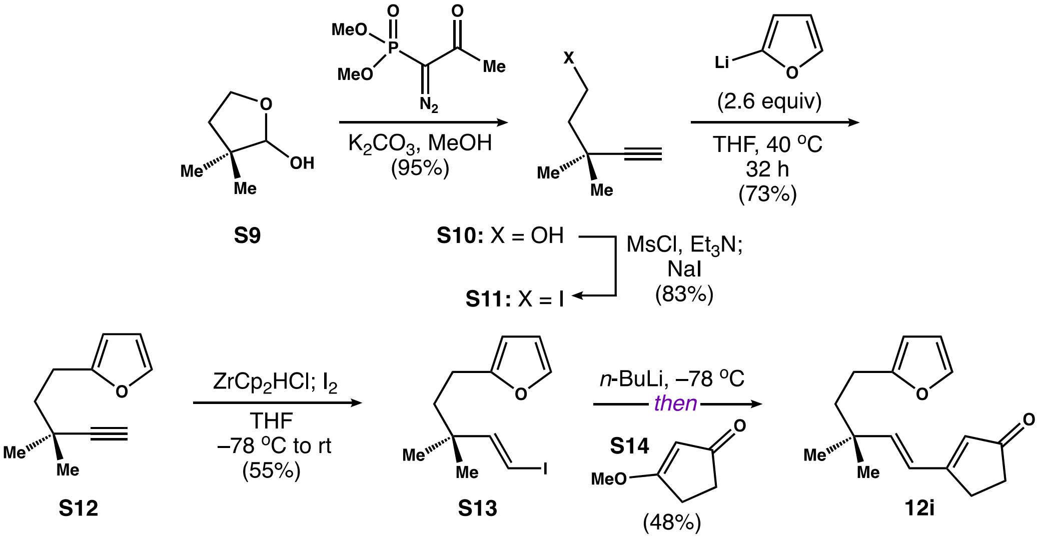

Scheme S2. Preparation of photosubstrate **12i**.

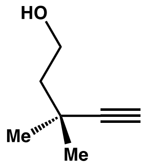

S10: $C_7H_{12}O$
MW: 112.17

3,3-dimethylpent-4-yn-1-ol (**S10**): Following a modification of the procedure reported by Perumal,[9] Ohira-Bestmann reagent (3.11 g, 16.2 mmol) was added drop wise via syringe to a stirred suspension of **S9** (935 mg, 8.10 mmol) and K_2CO_3 (5.60 g, 40.5 mmol) in MeOH (54 mL). After addition, the reaction was stirred at rt for 27 h and then concentrated under reduced pressure. The resulting residue was digested in water (50 mL) and extracted with Et_2O (4 x 50 mL). The combined organics were washed with brine (100 mL), dried over $MgSO_4$, filtered, and concentrated under reduced pressure. The crude residue was purified by flash chromatography (SiO_2; 9:1 hexanes/EtOAc) to afford **S10** (882 mg, 7.70 mmol, 95% yield) as a colorless oil: **^{1}H NMR** (400 MHz, $CDCl_3$) δ 3.86 (t, J = 6.6, 2H), 2.16 (s, 1H), 1.72 (t, J = 6.6, 2H), 1.26 (s, 6H). All other characterization data was identical to previous reported values.[10]

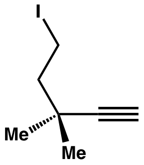

S11: $C_7H_{11}I$
MW: 222.07

5-iodo-3,3-dimethylpent-1-yne (**S11**): Freshly distilled methanesulfonly chloride (0.9 mL, 11 mmol) was added drop wise to a solution of **S10** (978 mg, 8.71 mmol) and Et_3N (3.6 mL, 25 mmol) in CH_2Cl_2 maintained at 0 °C. The resulting solution as warmed to rt and maintained for 1 h. The reaction mixture was then diluted with CH_2Cl_2 (40 mL) and treated with saturated aq. $NaHCO_3$ (30 mL). The resulting slurry was extracted with CH_2Cl_2 (3 x 20 mL). The combined organic extracts were washed with water (50 mL) and brine (50 mL), dried over $MgSO_4$, filtered, and concentrated under reduced pressure. The resulting crude mesylate (1.30 g) was used directly in the next step without further purification.

The crude residue prepared above was dissolved in acetone (17 mL) and NaI (4.05 g, 27.0 mmol) was added in a single portion. The resulting suspension was warmed to 70 °C and stirred for 18 h. The reaction mixture was then cooled to rt and concentrated under reduced pressure. The resulting solid was digested in water (50 mL) and extracted with Et_2O (4 x 30 mL). The combined organic extracts were dried over $MgSO_4$, filtered, and concentrated under reduced pressure. The resulting oil was purified by flash chromatography (SiO_2, hexanes) to afford **S11** (1.60 g, 7.20 mmol, 83% overall yield from **S10**) as a colorless oil: **^{1}H NMR** (400 MHz, $CDCl_3$) δ 3.31–3.27 (m, 2H), 2.14 (s, 1H), 2.08–2.03 (m, 2H), 1.22 (s, 6H); **^{13}C NMR** (100 MHz, $CDCl_3$) δ C: 89.7, 33.3; CH: 69.1; CH_2: 47.8, 28.7; CH_3: 0.11; **IR** (thin film): 3298, 2969, 1469, 1199 cm^{-1}; **HRMS**-ESI (m/z) $[M+H]^+$ calculated for $C_7H_{12}I$ = 222.9978; found 222.9969.

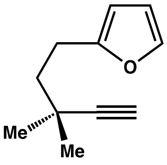

S12: $C_{11}H_{14}O$
MW: 162.23

2-(3,3-dimethylpent-4-yn-1-yl)furan (**S12**): Following a modification of the procedure reported for **S1**, a solution of furan (0.6 mL, 8.0 mmol) in THF (4 mL) was cooled to –78 °C. A solution of *n*-BuLi (2.9 mL, 5.2 mmol, 1.8 M in THF) was added drop wise via syringe. The resulting solution was warmed to 0 °C. After 4 h, **S11** (444 mg, 2.00 mmol) was added via syringe. The reaction mixture was warmed to rt over 15 min and then heated to 42 °C. After 32 h, the reaction was cooled to rt and treated with water (10 mL, **caution: exothermic**). The resulting slurry was extracted with Et_2O (3 x 10 mL). The combined organic extracts were washed with water (10

mL) and brine (10 mL), dried over $MgSO_4$, filtered, and concentrated under reduced pressure. The resulting crude oil was purified by flash chromatography (SiO_2; hexanes) to afford **S12** (240 mg, 1.47 mmol, 73% yield) as a yellow oil: **^{1}H NMR** (400 MHz, $CDCl_3$) δ 7.30 (dd, *J* = 0.7, 1.8, 1H), 6.28 (dd, *J* = 1.9, 3.1, 1H), 6.00–5.99 (m, 1H), 2.83–2.79 (m, 2H), 2.13 (s, 1H), 1.78–1.74 (m, 2H), 1.26 (s, 6H); **^{13}C NMR** (100 MHz, $CDCl_3$) δ C: 156.1, 68.47, 30.8; CH: 140.8, 110.1, 104.6, 90.9; CH_2: 41.1, 24.2; CH_3: 29.0; **IR** (thin film): 3302, 2857, 1597, 1007 cm^{-1}; **HRMS**-DART (m/z) $[M+H]^+$ calculated for $C_{11}H_{15}I$ = 163.1117; found 163.1114.

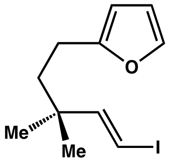

S13: $C_{11}H_{15}IO$
MW: 290.14

(*E*)-2-(5-iodo-3,3-dimethylpent-4-en-1-yl)furan (**S13**): A solution of **S12** (130 mg, 0.80 mmol) in THF (2 mL) was added drop wise to a stirred suspension of $ZrCp_2HCl$ (227 mg, 0.88 mmol) in THF (2 mL) at rt. After 1 h, the reaction mixture was cooled to –78 °C and a solution of I_2 (230 mg, 0.90 mmol) in THF (2 mL) was added drop wise via syringe over 10 min. The reaction was maintained at –78 °C for 30 min and then treated with wet Et_2O (15 mL, 1% H_2O by volume). The resulting slurry was warmed to rt and treated with saturated aq. $Na_2S_2O_3$ (5 mL). The organic layer was separated and washed with water (5 mL) and brine (5 mL). The combined organics were dried over $MgSO_4$, filtered, and concentrated under reduced pressure. The resulting crude oil was purified by flash chromatography (SiO_2; hexanes) to afford **S13** (130 mg, 0.44 mmol, 55% yield) as a yellow oil: **^{1}H NMR** (400 MHz, $CDCl_3$) δ 7.29 (d, *J* = 1.1, 1H), 6.50 (d, *J* = 14.6 1H), 6.27 (dd, *J* = 1.9, 3.1, 1H), 6.00–5.96 (m, 2H), 2.57–2.53 (m, 2H), 1.67–1.63 (m, 2H), 1.05 (s, 6H); **^{13}C NMR** (100 MHz, $CDCl_3$) δ C: 156.1, 40.1; CH: 155.0, 140.8, 110.1, 104.5, 73.1; CH_2: 40.7, 23.5; CH_3: 26.2; **IR** (thin film): 2960, 1597, 1006 cm^{-1}; **HRMS**-ESI (m/z) $[M+H]^+$ calculated for $C_{11}H_{16}IO$ = 219.0240; found 219.0242.

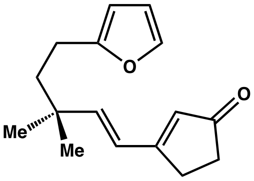

12i: $C_{16}H_{20}O_2$
MW: 244.33

Photoprecursor **12i**: Following a modification of the procedure reported by Mauduit,[11] *n*-BuLi (0.2 mL, 0.4 mmol, 2.1 M in THF) was added drop wise via syringe to a solution of **S13** (120 mg, 0.41 mmol) in THF (1 mL) at –78 °C. After 5 min, a solution of 3-methoxycyclopent-2-enone (50 mg, 0.5 mmol) in THF (0.3 mL) was added drop wise over 30 min. The reaction mixture then allowed to warm to –30 °C and maintained for 3 h. A solution of 5% aq. H_2SO_4 (0.5 mL) was added at –30 °C and then the reaction was allowed to warm to rt over 30 min. The resulting slurry was extracted with Et_2O (3 x 5 mL). The combined organic extracts were washed with water (5 mL) and brine (5 mL), dried over $MgSO_4$, filtered, and concentrated under reduced pressure. The resulting crude residue was purified by flash chromatography (SiO_2; 9:1 hexanes/acetone) to afford **12i** (48 mg, 0.2 mmol, 48% yield) as a yellow oil: **^{1}H NMR** (400 MHz, $CDCl_3$) δ 7.28 (d, *J* = 1.2, 1H), 6.47 (d, *J* = 16.1, 1H), 6.27–6.22 (m, 2H), 6.00 (s, 1H), 5.96 (dd, *J* = 3.1, 0.4, 1H), 2.72 (dd, *J* = 4.9, 3.4, 2H), 2.58–2.54 (m, 2H), 2.46–2.44 (m, 2H), 1.78–1.74 (m, 2H), 1.14 (s, 6H); **^{13}C NMR** (100 MHz, $CDCl_3$) δ C: 209.5, 172.8, 155.9, 36.8; CH: 149.1, 140.8, 129.7, 123.3, 110.2, 104.6; CH_2: 40.5, 36.8, 34.7, 27.0, 26.6, 13.6; CH_3: 26.6; **IR** (thin film): 2960, 1700, 1634, 1280 cm^{-1}; **HRMS**-ESI (m/z) $[M+Na]^+$ calculated for $C_{16}H_{20}O_2Na$ = 267.1356; found 267.1358.

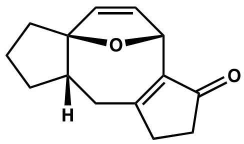

3: $C_{14}H_{16}O_2$
MW: 216.28

Gram-scale preparation of 3: A solution of **4** (1.00 g, 4.62 mmol) in *n*-BuOH (77 mL) was added to a 250 mL quartz round-bottom flask. The reaction mixture was exposed to UV light (h*v* = 350 nm) at 35 °C in a Rayonet photoreactor. After 55 h, the reaction was removed from the photoreactor, cooled to rt, and Et_3N (0.7 mL, 5 mmol) was added via syringe. The resulting solution as warmed to 100 °C in a pre-heated oil bath. After 6 h, the reaction mixture was cooled to rt and concentrated under reduced pressure. The resulting residue was purified by flash chromatography (neutralized SiO_2; 2:1 hexanes/EtOAc) to afford **3** (610 mg, 2.82 mmol, 61% yield) as a yellow solid: **mp** = 50–51 °C; **^{1}H NMR** (400 MHz, $CDCl_3$) δ 6.08 (d, *J* = 5.9, 1H), 5.86 (dd, *J* = 5.9, 1.8, 1H), 5.45 (br s, 1H), 2.55–2.46 (m, 3H), 2.41–2.25 (m, 2H), 2.16–2.11 (m, 2H), 1.93–1.70 (m, 5H), 1.32–1.25 (m, 1H); **^{13}C NMR** (150 MHz, $CDCl_3$) δ C: 206.7, 176.3, 142.1, 96.6; CH: 129.4, 128.6, 74.7, 49.2; CH_2: 34,6, 34.4, 33.9, 32.1, 28.9, 20.1; **IR** (thin film): 1738, 1692, 1683, 1625 cm^{-1}; **HRMS**-ESI (m/z) $[M+Na]^+$ calculated for $C_{14}H_{16}O_2Na$ = 239.1048; found 239.1057. Relative stereochemistry was established by single crystal X-ray diffraction (CCDC entry 1816385).[12]

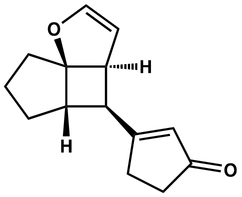

5: $C_{14}H_{16}O_2$
MW: 216.28

Cyclobutane **5**: A solution of photosubstrate **4** (1.00 g, 4.62 mmol) in MeCN (77 mL) was added to a quartz flask. The reaction mixture was exposed to UV light (h*v* = 350 nm) at 35 °C in a Rayonet photoreactor. After 21 h, the reaction was removed from the photoreactor and concentrated under reduced pressure. The resulting crude residue was purified by flash chromatography (neutralized SiO_2; 7:2 hexanes/EtOAc) to afford **5** (530 mg, 2.45 mmol, 53% yield): **mp** = 89–90 °C; **^{1}H NMR** (600 MHz, $CDCl_3$) δ 6.36 (dd, *J* = 2.8, 1.4, 1H), 5.98 (app q, *J* = 1.5, 1H), 4.83 (app t, *J* = 2.6, 1H), 3.57 (dt, *J* = 7.9, 1.3, 1H), 2.96 (t, *J* = 6.7, 1H), 2.87 (t, *J* = 7.3, 1H), 2.52–2.29 (m, 2H), 2.40 (t, *J* = 4.8, 2H), 2.04–1.98 (m, 2H), 1.98–1.90 (m, 1H), 1.89–1.82 (m, 2H), 1.53 (dd, *J* = 12.7, 1.5, 1H); **^{13}C NMR** (150 MHz, $CDCl_3$) δ C: 209.7, 181.1, 93.8; CH: 148.4, 129.6, 102.0, 50.1, 49.4, 43.7; CH_2: 35.5, 33.5, 30.9, 30.8, 24.7; **HRMS**-ESI (m/z) $[M+Na]^+$ calculated for $C_{14}H_{16}O_2Na$ = 239.1048; found 239.1050. Relative stereochemistry was established by single crystal X-ray diffraction (CCDC entry 1816383).[12]

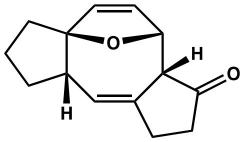

6: $C_{14}H_{16}O_2$
MW: 216.28

Cope-rearrangement product **6**: An analytic sample was prepared by charging an NMR tube with a degassed solution of **5** (6.5 mg, 0.03 mmol) in toluene-d_8 (0.5 mL) and heating the reaction mixture to 100 °C for 1.5 h. NMR spectra were collected from the crude reaction mixture: **^{1}H NMR** (600 MHz, toluene-d_8) δ 5.69–5.66 (m, 2H), 5.31–5.29 (m, 2H), 3.24 (d, *J* = 1.5, 1H), 2.82–2.77 (m, 1H), 2.17–2.05 (m, 1H), 1.99–1.93 (m, 1H), 1.81–1.74 (m, 1H), 1.65–1.54 (m, 5H), 1.51–1.43 (m, 1H), 1.13–1.05 (m, 1H); **^{13}C NMR** (150 MHz, toluene-d_8) δ C: 213.5, 136.6, 95.6; CH: 132.4, 127.4, 123.7, 81.4, 59.7, 50.5; CH_2: 38.3, 33.4, 28.9, 27.7, 19.5; **HRMS**-ESI (m/z) $[M+Na]^+$ calculated for $C_{14}H_{16}O_2Na$ = 239.1048; found 239.1063. Relative stereochemistry was established by single crystal X-ray diffraction (CCDC entry 1816390).[12]

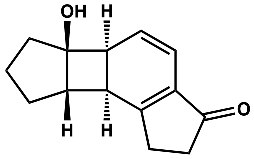

11: $C_{14}H_{16}O_2$
MW: 216.28

Byproduct **11**: An analytical sample was prepared by flash chromatography (SiO_2; 2:1 hexanes/EtOAc): **^{1}H NMR** (400 MHz, $CDCl_3$) δ 6.37 (d, *J* = 9.8, 1H), 5.63 (dd, *J* = 9.8, 5.1, 1H), 3.36 (dd, *J* = 9.2, 5.1, 1H), 2.67–2.54 (m, 4H), 2.46–2.43 (m, 2H), 2.36–2.30 (m, 1H), 1.98–1.79 (m, 4H), 1.69–1.63 (m, 2H); **^{13}C NMR** (150 MHz, $CDCl_3$) C: 205.3, 173.1, 132.5, 88.0; CH: 123.7, 119.6, 58.5, 45.3, 34.3; CH_2: 39.5, 35.0, 32.0, 27.2, 24.6; **IR** (thin film): 3360, 1671, 1632, 1603 cm^{-1}; **HRMS**-DART (m/z) $[M+H]^+$ calculated for $C_{14}H_{17}O_2$ = 217.1223; found 217.1231. Relative stereochemistry was established by single crystal X-ray diffraction (CCDC entry 1816388).[12]

Preparation of 5-8-5 ring system derivatives 13a–j. *General Procedure.* A solution of photosubstrate (1 equiv) in MeCN (60 mM) was added to a quartz flask and exposed to UV light (h*v* = 350 nm) at 35 °C in a Rayonet photoreactor for the indicated amount of time. The reaction was removed from the photoreactor, cooled to rt, and Et_3N (1.1 equiv) was added via syringe. The resulting solution was warmed to 80 °C or 100 °C in a pre-heated oil bath for the specified amount of time. The reaction mixture was cooled to rt and concentrated under reduced pressure. The resulting crude products were purified by flash chromatography using neutralized silica gel.[13]

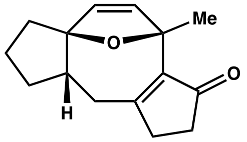

13a: $C_{15}H_{18}O_2$
MW: 230.31

Product **13a**: Following the general procedure, **13a** was synthesized from **12a** (96 mg, 0.42 mmol). The reaction mixture was irradiated at 35 °C for 7.5 h. Following addition of Et_3N, the resulting solution was heated to 80 °C for 2 h. The resulting crude residue was purified by flash chromatography (neutralized SiO_2; 19:1 CH_2Cl_2/Et_2O) to afford **13a** (51 mg, 0.22 mmol, 53% yield) as a colorless solid: **mp** = 88–89 °C; **^{1}H NMR** (400 MHz, $CDCl_3$) δ 5.96 (d, *J* = 5.7, 1H), 5.70 (dd, *J* = 5.8, 1H), 2.54–2.46 (m, 3H), 2.40–2.33 (m, 1H), 2.28–2.21 (m, 1H), 1.91–1.81 (m, 4H), 1.79 (s, 3H), 1.76–1.69 (m, 1H), 1.29–1.22 (m, 1H); **^{13}C NMR** (100 MHz, $CDCl_3$) δ C: 206.6, 176.8, 142.9, 96.9, 84.7; CH: 132.9, 127.9, 48.5; CH_2: 35.8, 35.3, 34.2, 32.2, 28.5, 20.2; CH_3: 22.7; **IR** (thin film): 1680, 1598 cm^{-1}; **HRMS**-ESI (m/z) $[M+Na]^+$ calculated for $C_{14}H_{16}O_2Na$ = 253.1205; found 253.1207.

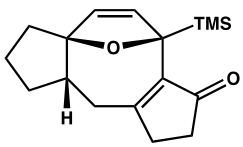

13b: $C_{17}H_{24}O_2Si$
MW: 288.46

Product **13b**: Following the general procedure, **13b** was synthesized from **12b** (145 mg, 0.50 mmol). The reaction mixture was irradiated at 35 °C for 12 h. Following addition of Et_3N, the resulting solution was heated to 80 °C for 15.5 h. The resulting crude residue was purified by flash chromatography (neutralized SiO_2; 9:1 hexanes/acetone) to afford **13b** (69 mg, 0.24 mmol, 48% yield) as a colorless solid: **mp** = 57–58 °C; **^{1}H NMR** (400 MHz, $CDCl_3$) δ 5.90 (d, *J* = 5.8, 1H), 5.70 (d, *J* = 5.9, 1H), 2.57–2.50 (m, 3H), 2.37–2.30 (m, 1H), 2.27–2.17 (m, 2H), 2.13–2.04 (m, 1H), 1.95–1.77 (m, 4H), 1.75–1.64 (m, 1H), 1.31–1.19 (m, 1H), 0.12 (s, 9H); **^{13}C NMR** (100 MHz, $CDCl_3$) δ C: 206.7, 177.9, 144.9, 98.1, 80.3; CH: 130.2, 126.6, 48.9; CH_2: 35.4, 34.1, 33.5, 33.2, 28.5, 20.3; CH_3: –2.1; **IR** (thin film): 1670, 1591 cm^{-1}; **HRMS**-ESI (m/z) $[M+Na]^+$ calculated for $C_{17}H_{24}O_2SiNa$ = 311.1443; found 311.1438.

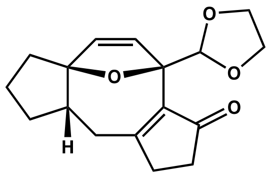

13c: $C_{17}H_{20}O_4$
MW: 288.34

Product **13c**: Following the general procedure, **13c** was synthesized from **12c** (146 mg, 0.50 mmol). The reaction mixture was irradiated at 35 °C for 18 h. Following addition of Et_3N, the resulting solution was heated to 80 °C for 20 h. The resulting crude residue was purified by flash chromatography (neutralized SiO_2; 9:1 hexanes/acetone) to afford **13c** (60 mg, 0.21 mmol, 41% yield) as a colorless solid: **mp** = 123–125 °C; **^{1}H NMR** (400 MHz, $CDCl_3$) δ 6.32 (d, *J* = 0.8, 1H), 6.09 (d, *J* = 6.1, 5.8 1H), 5.78 (d, *J* = 5.8, 1H) 4.13–4.10 (m, 1H), 4.05–3.92 (m, 3H), 2.58–2.51 (m, 3H), 2.47–2.24 (m, 3H), 2.09 (dd, *J* = 16.4, 12.4, 1H), 1.94–1.88 (m, 2H), 1.86–1.79 (m, 2H) 1.77–1.71 (m, 1H), 1.31–1.23 (m, 1H); **^{13}C NMR** (100 MHz, $CDCl_3$) δ C: 205.9, 178.3, 142.2, 97.2, 89.6; CH: 128.9, 128.2, 101.3, 49.1; CH_2: 66.2, 66.1, 49.1, 35.8, 35.1, 33.5, 32.9, 28.4, 20.3; **IR** (thin film): 1689, 1560 cm^{-1}; **HRMS**-ESI (m/z) $[M+Na]^+$ calculated for $C_{17}H_{20}O_4Na$ = 311.1259; found 311.1254.

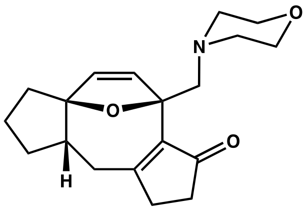

13d: $C_{19}H_{25}NO_3$
MW: 315.41

Product **13d**: Following the general procedure, **13d** was synthesized from **12d** (316 mg, 1.00 mmol). The reaction mixture was irradiated at 35 °C for 10 h. Following addition of Et_3N, the resulting solution was heated to 80 °C for 5 h. The resulting crude residue was purified by flash chromatography (neutralized SiO_2; 4:1 hexanes/acetone) to afford **13d** (190 mg, 0.60 mmol, 60% yield) as a pink solid: **mp** = 86–88 °C; **^{1}H NMR** (400 MHz, $CDCl_3$) δ 5.98 (d, *J* = 5.8, 1H), 5.74 (d, *J* = 5.8, 1H), 3.67–3.58 (m, 4H), 3.39 (d, *J* = 14.4, 1H), 3.14 (d, *J* = 14.4, 1H), 2.76–2.73 (m, 2H), 2.59–2.47 (m, 5H), 2.43–2.35 (m, 1H), 2.28–2.07 (m, 3H), 1.86–1.83 (m, 4H), 1.76–1.70 (m, 1H), 1.29–1.24 (m, 1H); **^{13}C NMR** (100 MHz, $CDCl_3$) C: 206.4, 176.8, 143.2, 96.9, 89.5; CH: 131.2, 127.8, 48.9; CH_2: 67.2, 60.0, 55.4, 36.1, 35.4, 33.8, 32.5, 28.4, 20.4; **IR** (thin film): 1690, 1591 cm^{-1}; **HRMS**-ESI (m/z) $[M+H]^+$ calculated for $C_{19}H_{26}O_3$ = 316.1913; found 316.1903.

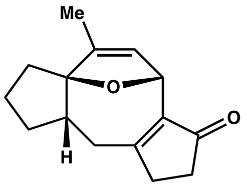

13e: $C_{15}H_{18}O_2$
MW: 230.31

Product **13e**: Following a variation of the general procedure employing *n*-BuOH in place of MeCN, **13e** was synthesized from **12e** (115 mg, 0.50 mmol). The reaction mixture was irradiated at 35 °C for 12 h. Following addition of Et_3N, the resulting solution was heated to 100 °C for 1 h. The resulting crude residue was purified by flash chromatography (neutralized SiO_2; 9:1 hexanes/acetone) to afford **13e** (65 mg, 0.28 mmol, 57% yield) as yellow oil: **^{1}H NMR** (400 MHz, $CDCl_3$) δ 5.56 (d, *J* = 1.2, 1H), 5.25 (br s, 1H), 2.54–2.48 (m, 3H), 2.41–2.18 (m, 4H), 2.06–2.00 (m, 1H), 2.00–1.91 (m, 3H), 1.87 (s, 3H), 1.80–1.71 (m, 1H), 1.44–1.35 (m, 1H); **^{13}C NMR** (100 MHz, $CDCl_3$) δ C: 209.8, 176.3, 143.4, 139.5, 97.3; CH: 125.1, 73.2, 50.3; CH_2: 34.5, 34.2, 32.9, 32.0, 29.9, 20.5; CH_3: 12.7 (s, 3H); **IR** (thin film): 1689, 1625 cm^{-1}; **HRMS**-ESI (m/z) $[M+Na]^+$ calculated for $C_{15}H_{18}O_2Na$ = 253.1205; found 253.1201.

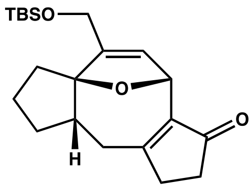

13f: $C_{21}H_{32}O_3Si$
MW: 360.57

Product **13f**: Following a variation of the general procedure employing *n*-BuOH in place of MeCN, **13f** was synthesized from **12f** (120 mg, 0.33 mmol). The reaction mixture was irradiated at 35 °C for 12 h. Following addition of Et_3N, the resulting solution was heated to 100 °C for 8 h. The resulting crude residue was purified by flash chromatography (neutralized SiO_2; 4:1 hexanes/EtOAc) to afford **13f** (51 mg, 0.14 mmol, 43% yield) as colorless solid: **mp** = 98–99 °C; **^{1}H NMR** (400 MHz, $CDCl_3$) δ 5.86 (d, *J* = 1.5, 1H), 5.35 (br s, 1H), 4.33 (s, 2H) 2.54 (t, *J* = 4.6, 2H), 2.50–2.47 (m, 1H), 2.44–2.27 (m, 4H), 2.09–1.87 (m, 4H), 1.75–1.65 (m, 1H), 1.47–1.39 (m, 1H), 0.91 (s, 9H), 0.08 (s, 6H); **^{13}C NMR** (100 MHz, $CDCl_3$) C: 206.7, 176.4, 144.1, 142.9, 96.8, 18.3; CH: 125.9, 73.3, 50.2; CH_2: 58.9, 34.5, 34.1,

33.3, 32.1, 29.2, 20.3; CH_3: 25.9, –5.4, –5.5; **IR** (thin film): 1692, 1606 cm^{-1}; **HRMS**-ESI (m/z) $[M+Na]^+$ calculated for $C_{21}H_{32}O_3SiNa$ = 383.2018; found 383.2015.

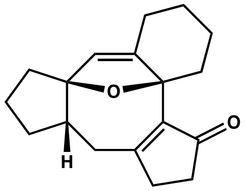

13g: $C_{18}H_{22}O_2$
MW: 270.37

Product **13g**: Following the general procedure, **13g** was synthesized from **12g** (135 mg, 0.50 mmol). The reaction mixture was irradiated at 35 °C for 20 h. Following addition of Et_3N, the resulting solution was heated to 80 °C for 1 h. The resulting crude residue was purified by flash chromatography (neutralized SiO_2; 97:3 CH_2Cl_2/Et_2O) to afford **13g** (34.0 mg, 0.126 mmol, 25% yield of the major diastereomer, d.r. = 10:1) as a colorless solid: **mp =** 120–124 °C **^{1}H NMR** (400 MHz, $CDCl_3$) δ 5.52 (s, 1H), 3.18 (d, J = 12.4, 1H), 2.53–2.51 (m, 2H), 2.47–2.42 (m, 2H), 2.36–2.26 (m, 3H), 2.00–1.92 (m, 1H), 1.90–1.56 (m, 9H), 1.39 (td, J = 12.6, 4.1, 1H), 1.33–1.15 (m, 2H); **^{13}C NMR** (100 MHz, $CDCl_3$) δ C: 206.6, 179.6, 143.2, 141.7, 95.3, 84.5; CH: 119.7, 46.6; CH_2: 35.6, 35.5, 35.0, 34.2, 32.7, 28.4, 26.8, 26.4, 23.6, 19.8; **IR** (thin film): 1681, 1590 cm^{-1}; **HRMS**-ESI (m/z) $[M+Na]^+$ calculated for $C_{18}H_{22}O_2Na$ = 293.1512; found 293.1518. Relative stereochemistry of the major diastereomer was established by single crystal X-ray diffraction (CCDC entry 1816389).[12]

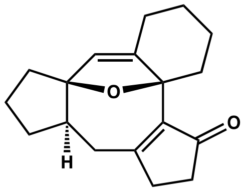

S14: $C_{18}H_{22}O_2$
MW: 270.37

Diastereomer **S14**: Following a variation of the general procedure employing *n*-BuOH in place of MeCN, **S14** was synthesized from **12g** (61.0 mg, 0.23 mmol). The reaction mixture was irradiated at 35 °C for 20 h. Following addition of Et_3N, the resulting solution was heated to 100 °C for 2 h. The resulting crude residue was purified by flash chromatography (neutralized SiO_2; 9:1 Hexanes/EtOAc) to afford **S14** (16 mg, 0.06 mmol, 26% yield) as a colorless solid: **^{1}H NMR** (400 MHz, $CDCl_3$) δ 5.39 (s, 1H), 2.91 (dd, J = 16.1, 9.6, 1H), 2.52–2.39 (m, 2H), 2.37–2.31 (m, 2H), 2.10–2.00 (m, 3H), 1.90–1.75 (m, 5H), 1.67–1.61 (m, 1H), 1.60–1.51 (m, 1H), 1.44–1.38 (m, 1H), 1.33–1.23 (m, 1H); **^{13}C NMR** (150 MHz, $CDCl_3$) δ C: 207.2, 176.7, 146.7, 145.3, 94.6, 86.9; CH: 125.3, 45.9; CH_2: 38.8, 36.7, 36.4, 35.8, 32.7, 32.5, 26.6, 25.8, 23.7, 22.3; **IR** (thin film): 1692, 1606 cm^{-1}; **HRMS**-ESI (m/z) $[M+Na]^+$ calculated for $C_{18}H_{22}O_2Na$ = 293.1518; found 293.1511. Relative stereochemistry was established by single crystal X-ray diffraction (CCDC entry 1816384).[12]

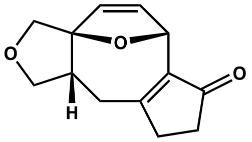

13h: $C_{13}H_{14}O_3$
MW: 218.25

Product **13h**: Following a variation of the general procedure employing *n*-BuOH in place of MeCN, **13h** was synthesized from **12h** (114 mg, 0.50 mmol). The reaction mixture was irradiated at 35 °C for 42 h. Following addition of Et_3N, the resulting solution was heated to 100 °C for 1 h. The resulting crude residue was purified by flash chromatography (neutralized SiO_2; 1:9 hexanes/MTBE) to afford **13h** (25.0 mg, 0.110 mmol, 22% yield) as a white foam: **^{1}H NMR** (400 MHz, $CDCl_3$) δ 6.23 (d, J = 5.8, 1H), 6.00 (d, J = 5.2, 1H), 5.51 (br s, 1H), 4.08 (t, J = 8.2, 1H), 3.90 (d, J = 8.5, 1H), 3.83 (d, J = 8.5, 1H), 3.51 (dd, J = 11.6, 8.5, 1H), 2.64–2.56 (m, 3H), 2.53–2.48 (m, 1H), 2.45–2.29 (m, 2H), 2.23 (dd, J = 15.5, 13.0, 1H); **^{13}C NMR** (100 MHz, $CDCl_3$) C: 206.4, 174.5, 142.0, 94.0; CH: 130.3, 127.8, 75.2, 48.4; CH_2: 72.2, 70.9, 34.3, 32.2, 30.4; **IR** (thin film): 1686, 1621 cm^{-1}; **HRMS**-DART (m/z) $[M+H]^+$ calculated for $C_{13}H_{15}O_3$ = 219.1016; found 219.1013.

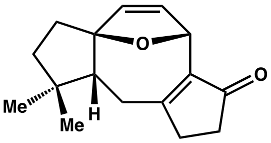

13i: $C_{16}H_{20}O_2$
MW: 244.33

Product **13i**: Following a variation of the general procedure employing *n*-BuOH in place of MeCN, **13i** was synthesized from **12i** (67 mg, 0.27 mmol). The reaction mixture was irradiated at 35 °C for 9 h. Following addition of Et_3N, the resulting solution was heated to 100 °C for 9 h. The resulting crude residue was purified by flash chromatography (neutralized SiO_2; 4:1 hexanes/EtOAc) to afford **13i** (27 mg, 0.11 mmol, 40% yield) as a white solid: **mp** = 117–122 °C; **^{1}H NMR** (600 MHz, $CDCl_3$) δ 6.06 (d, J = 5.6, 1H), 5.88 (dd, J = 5.9, 2.0, 1H), 5.42 (br s, 1H), 2.59–2.56 (m, 2H), 2.40 (dq, J = 18.9, 6.4, 2.9, 1H), 2.34–2.21 (m, 3H), 2.00–1.95 (m, 3H), 1.77–1.72 (m, 1H), 1.57 (dt, J = 16.0, 7.9, 1H), 1.06 (s, 3H), 0.88 (s, 3H); **^{13}C NMR** (100 MHz, $CDCl_3$) C: 206.7, 176.6, 141.9, 97.3, 38.6; CH: 131.3, 128.8, 74.4, 60.1; CH_2: 38.7, 34.5, 32.8, 32.3, 29.9; CH_3: 30.1, 24.7; **IR** (thin film): 1694, 1626 cm^{-1}; **HRMS**-ESI (m/z) $[M+H]^+$ calculated for $C_{16}H_{21}O_2$ = 245.1536; found 245.1541.

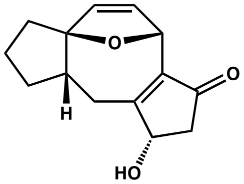

13j: $C_{14}H_{16}O_3$
MW: 232.28
d.r. = 1.3:1

Product **13j**: Following the general procedure, **13j** was synthesized from **12j** (182 mg, 0.78 mmol). The reaction mixture was irradiated at 35 °C for 6 h. Following addition of Et_3N, the resulting solution was heated to 100 °C for 1 h. The resulting crude residue was purified by flash chromatography (neutralized SiO_2; 1:1 hexanes/EtOAc) to afford **13i** (116 mg, 0.50 mmol, 64% combined yield, 1.3:1 mixture of diastereomers) as a white foam: **^{1}H NMR** (600 MHz, $CDCl_3$) major diastereomer, δ 5.84 (dd, *J* = 5.9, 1.9, 1H), 4.61 (br s, 1H), 2.55 (s, 1H); minor diastereomer, δ 5.91 (dd, *J* = 5.9, 1.9, 1H), 4.79 (br s, 1H), 2.42 (s, 1H); remaining ^{1}H resonances could not be resolved; **^{13}C NMR** (150 MHz, $CDCl_3$): major diastereomer, δ C: 203.1, 174.0, 143.0, 96.9; CH: 130.0, 127.7, 74.3, 71.1 49.7; CH_2: 44.5, 33.9, 30.2, 29.0, 20.2; minor diastereomer, δ C: 202.5, 173.8, 143.5, 96.8; CH: 130.1, 128.1, 73.8, 72.3 48.9; CH_2: 43.8, 34.0, 31.2, 29.0, 20.3; **IR** (thin film): 3424, 1702, 1690, 1635 cm^{-1}; **HRMS**-ESI (m/z) $[M+H]^+$ calculated for $C_{14}H_{17}O_3$ = 233.1172; found 233.1179.

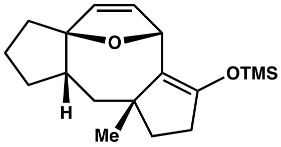

14: $C_{18}H_{28}O_2Si$
MW: 304.51

Silyl enol ether **14**: CuI (2.04 g, 10.7 mmol) was added to a flame-dried flask in a glove box. The flask was sealed, removed from the glove box, and THF (68 mL) was added via syringe. The resulting slurry was cooled to 0 °C and a solution of MeLi (13.5 mL, 21.6 mmol, 1.6 M in THF) was added drop wise over 15 min. The resulting solution was cooled to –78 °C. A solution of **3**[14] (1.16 mg, 5.35 mmol) and TMSCl (6.7 mL, 27 mmol) in THF (37 mL) was prepared in a separate flask and then added drop wise to the cooled mixture of Me_2CuLi over 1.5 h using an addition funnel. The resulting solution was maintained at –78 °C for 5 h, then the reaction was rapidly poured into a stirred suspension of Florisil (24 g) suspended in hexanes (220 mL) and Et_3N (24 mL).[15] The resulting slurry was stirred at 0 °C for 1 h, then filtered. The filter cake was vigorously washed with hexanes (3 x 25 mL). The combined filtrate was filtered again to remove precipitate and concentrated under reduced pressure to afford **14** (1.59 g, 5.22 mmol, 98% yield) as a brown oil in >95% purity. This sensitive intermediate[16] was used without further purification: **^{1}H NMR** (600 MHz, $CDCl_3$) δ 5.98 (dd, *J* = 5.8, 2.0, 1H), 5.74 (dd, *J* = 5.8, 0.8, 1H), 5.43 (br s, 1H), 2.46–2.41 (m, 1H), 2.35–2.29 (m, 1H), 2.00 (dd, *J* = 15.2, 8.5, 1H), 1.97–1.91 (m, 1H), 1.86–1.79 (m, 1H), 1.77–1.62 (m, 3H), 1.55–1.49 (m, 2H), 1.39 (dd, *J* = 13.4, 3.5, 1H), 1.34 (d, *J* = 11.9, 1H), 1.17 (s, 3H), 1.15–1.08 (m, 1H), 0.18 (s, 9H); **^{13}C NMR** (150 MHz, $CDCl_3$) δ C: 144.2, 126.2, 97.2, 47.0; CH: 132.4, 128.4, 79.7, 46.4; CH_2: 41.5, 39.6, 33.9, 31.6, 27.3, 19.8; CH_3: 24.9, 0.6.

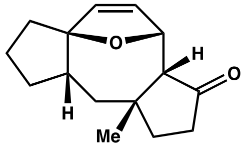

15: $C_{15}H_{20}O_2$
MW: 232.32

Ketone **15**: A solution of unpurified silyl enol ether **14** (1.15 g, 3.78 mmol) in THF (13 mL) was treated with 1 M aq. HCl (7.6 mL). The resulting slurry was stirred at rt for 1 h, then transferred to a separatory funnel and extracted with EtOAc (4 x 15 mL). The combined organic extracts were washed with water (25 mL) and brine (25 mL), dried over $MgSO_4$, filtered, and concentrated under reduced pressure to afford **15** (871 mg, 3.75 mmol, 99% yield) as a colorless solid. No further purification was required: **mp** = 89–91 °C; **^{1}H NMR** (600 MHz, $CDCl_3$) δ 6.20 (dd, *J* = 6.0, 1.5, 1H), 5.82 (d, *J* = 6.1, 1H), 5.13 (d, *J* = 8.2, 1H), 2.70 (d, *J* = 8.1, 1H), 2.39–2.33 (m, 1H), 2.23 (dd, *J* = 10.5, 5.9, 2H), 1.91–1.85 (m, 2H), 1.77–1.71 (m, 4H), 1.65–1.60 (m, 2H), 1.38 (s, 3H), 1.28 (dd, *J* = 13.9, 2.4, 1H), 1.10–1.02 (m, 1H); **^{13}C NMR** (150 MHz, $CDCl_3$) δ C: 220.3, 97.6, 44.8; CH: 132.0, 131.4, 82.2, 63.8, 46.4; CH_2: 38.7, 38.4, 37.4, 32.8, 27.0, 20.3; CH_3: 30.9; **IR** (thin film): 1725 cm^{-1}; **HRMS**-ESI (m/z) $[M+H]^+$ calculated for $C_{15}H_{21}O_2$ = 233.1542; found 233.1548. Relative stereochemistry was established by single crystal X-ray diffraction (CCDC entry 1816387).[12]

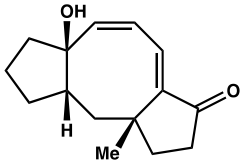

16: $C_{15}H_{20}O_2$
MW: 232.32

Dieneone **16**: A solution of unpurified silyl enol ether **14** (375 mg, 1.23 mmol) in CH_2Cl_2 (6 mL) was added drop wise over 25 min to a solution of BCl_3 (2.5 mL, 2.5 mmol, 1.0 M in heptane) in CH_2Cl_2 (6 mL) maintained at –78 °C. After 30 min, Et_3N (35 mL) followed by MeOH (35 mL) was added via syringe at –78 °C. The reaction mixture was warmed to rt and concentrated under reduced pressure. The resulting oil was diluted with CH_2Cl_2 (15 mL) and washed with H_2O (20 mL). The aqueous layer was then extracted with CH_2Cl_2 (4 x 10 mL). The combined organic extracts were dried over $MgSO_4$, filtered, and concentrated under reduced pressure. The resulting crude residue was purified by flash chromatography (SiO_2; 6:1 hexanes/acetone) to afford **16** (170 mg, 0.73 mmol, 60% yield) as a yellow solid: **mp** = 110–111 °C ; **^{1}H NMR** (600 MHz, $CDCl_3$) δ 6.56 (d, *J* = 6.2, 1H), 6.06 (dd, *J* = 12.2, 6.3, 1H), 5.91 (d, *J* = 12.2, 1H), 2.52–

2.45 (m, 1H), 2.45–2.39 (m, 1H) 2.23 (ddd, J = 19.2, 8.5, 1.7, 1H), 2.06 (br. s, 1H), 1.90–1.80 (m, 4H), 1.76–1.65 (m, 4H), 1.65–1.55 (m, 1H), 1.29 (s, 3H), 1.27–1.20 (m, 1H); **^{13}C NMR** (150 MHz, $CDCl_3$) δ C: 208.5, 149.0, 82.3, 43.4; CH: 141.8, 125.2, 125.1, 46.5; CH_2: 42.6, 39.6, 37.3, 34.2, 30.6, 20.3; CH_3: 23.3; **IR** (thin film): 3546, 1715, 1632 cm^{-1}; **HRMS**-ESI (m/z) $[M+Na]^+$ calculated for $C_{15}H_{20}O_2Na$ = 255.1361; found 255.1361. Relative stereochemistry was established by single crystal X-ray diffraction (CCDC entry 1816385).[12]

Preparation of *N-tert*-butylbenzenesulfinimidoyl chloride: Following a modification of the procedure reported by Mukiayama,[1] *S*-phenylthioacetate (2.0 mL, 15 mmol) was added drop wise to a solution *N*,*N*-dichloro-2-methylpropan-2-amine (2.69 g, 19 mmol) in benzene (6.5 mL) at rt. The resulting yellow solution was warmed to 80 ^oC during which time gas evolution occurred and the solution became red. After 2 h, the reaction mixture was concentrated under reduced pressure with care to minimize exposure to atmosphere. The resulting orange residue (3.85 g, 15 mmol, 100% yield) was analyzed for purity using ^{1}H NMR (>95% purity) and then digested in benzene (7.5 mL) to make a 2 M solution. The resultant orange solution was stable for ca. 1 week when stored under an N_2 atmosphere at rt: **^{1}H NMR** (400 MHz, C_6D_6) δ 7.92–7.88 (m, 2H), 6.94–6.90 (m, 3H), 1.38 (s, 9H). All other characterization data was identical to previously reported values.[1]

IMPORTANT: In early iterations of our chemistry we attempted to use commercially available *N-tert*-butylbenzenesulfinimidoyl chloride. This material resulted in low yield of the resultant photoproducts **12** and invariably arrived in low purity (ca. 50% purity by ^{1}H NMR). The use of oxidant prepared as described above was *critical* to achieve the yields reported in this manuscript.

3. ^{1}H NMR Experiment: Photoequilibration of (*E*)-4 [see, Scheme 3A].

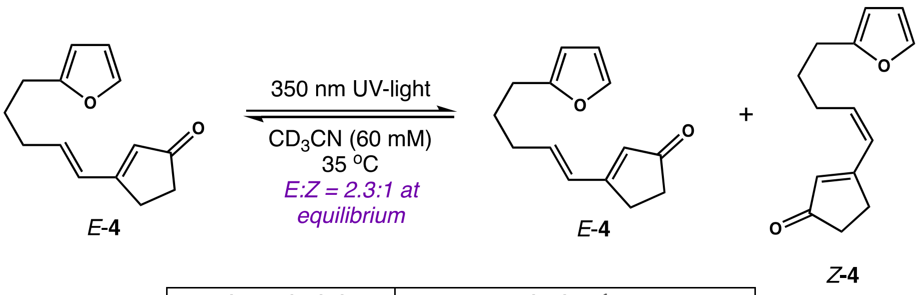

Time (min)	E:Z ratio by ^{1}H NMR
0	100:0
5	3:1
**10**	**2.2:1**
15	2.2:1
20	2.2:1

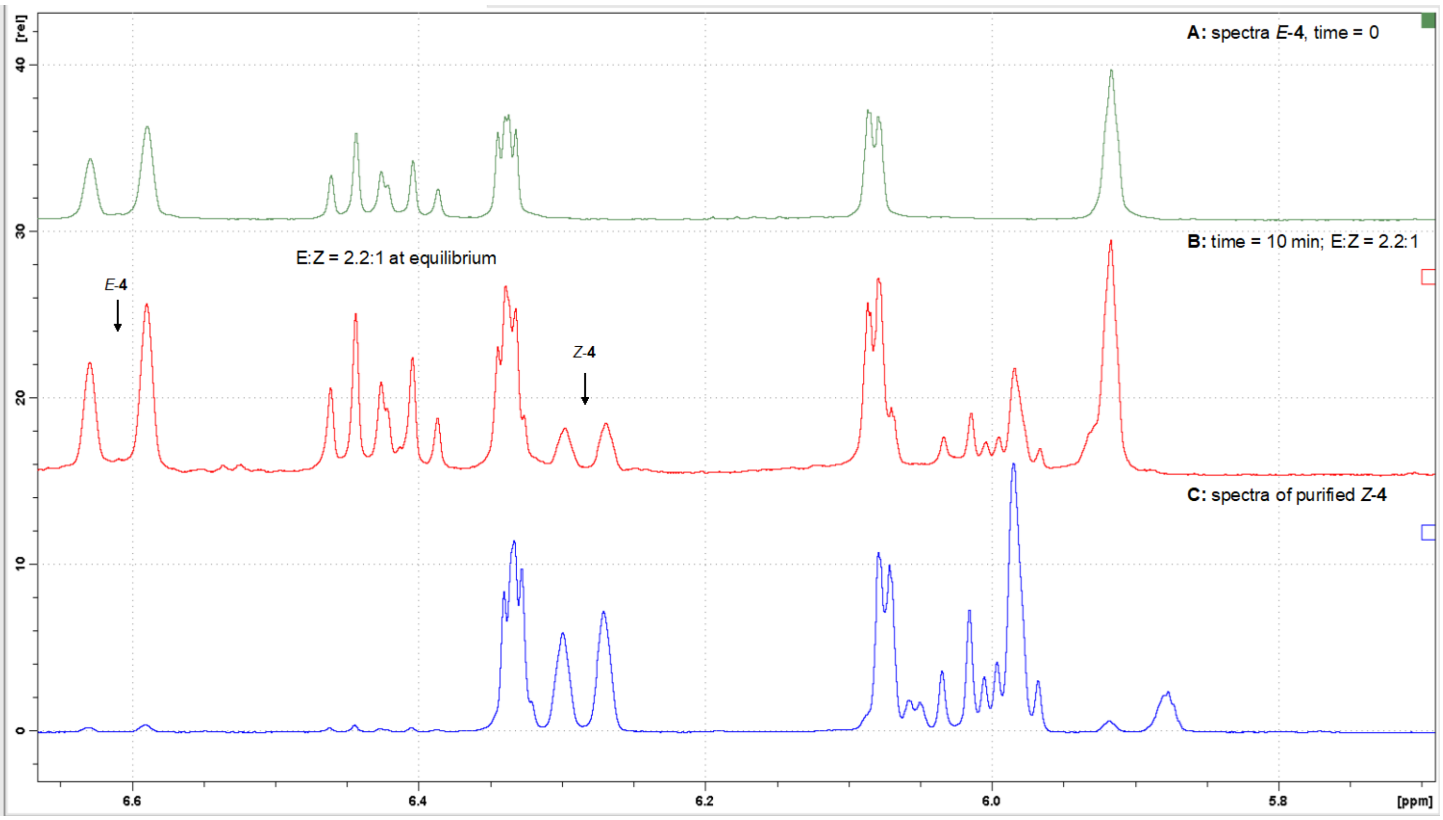

Procedure & Analysis: An NMR tube was charged with a solution of *E*-**4** (6.5 mg, 0.04 mmol) in CD_3CN (0.45 mL). A ^{1}H NMR spectra of the reaction mixture was obtained under ambient conditions (panel **A**, above). The reaction mixture was then exposed to 350 nm UV-light at 35 °C in a Rayonet photoreactor. The reaction mixture was analyzed by ^{1}H NMR in five-minute intervals. Equilibrium was established after 10 min (panel **B**). Results were compared to ^{1}H NMR spectra of independently prepared *Z*-**4** (panel **C**) in CD_3CN. We observed an equilibrium ratio of 2.3:1 under these conditions.

4. ^{1}H NMR Experiment: Photoequilibration of (*Z*)-4

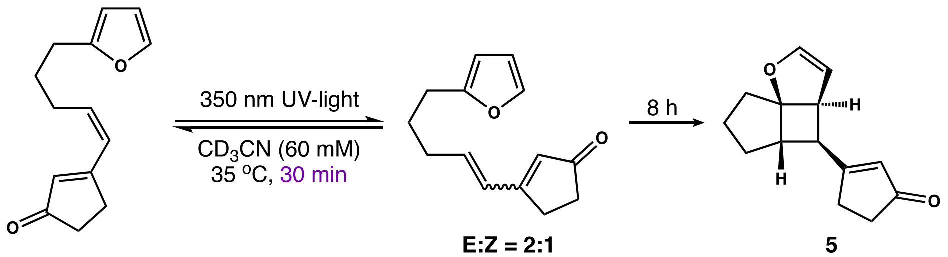

Z-**4** — **E:Z = 2:1** — **5**

single diastereomer observed by ^{1}H NMR

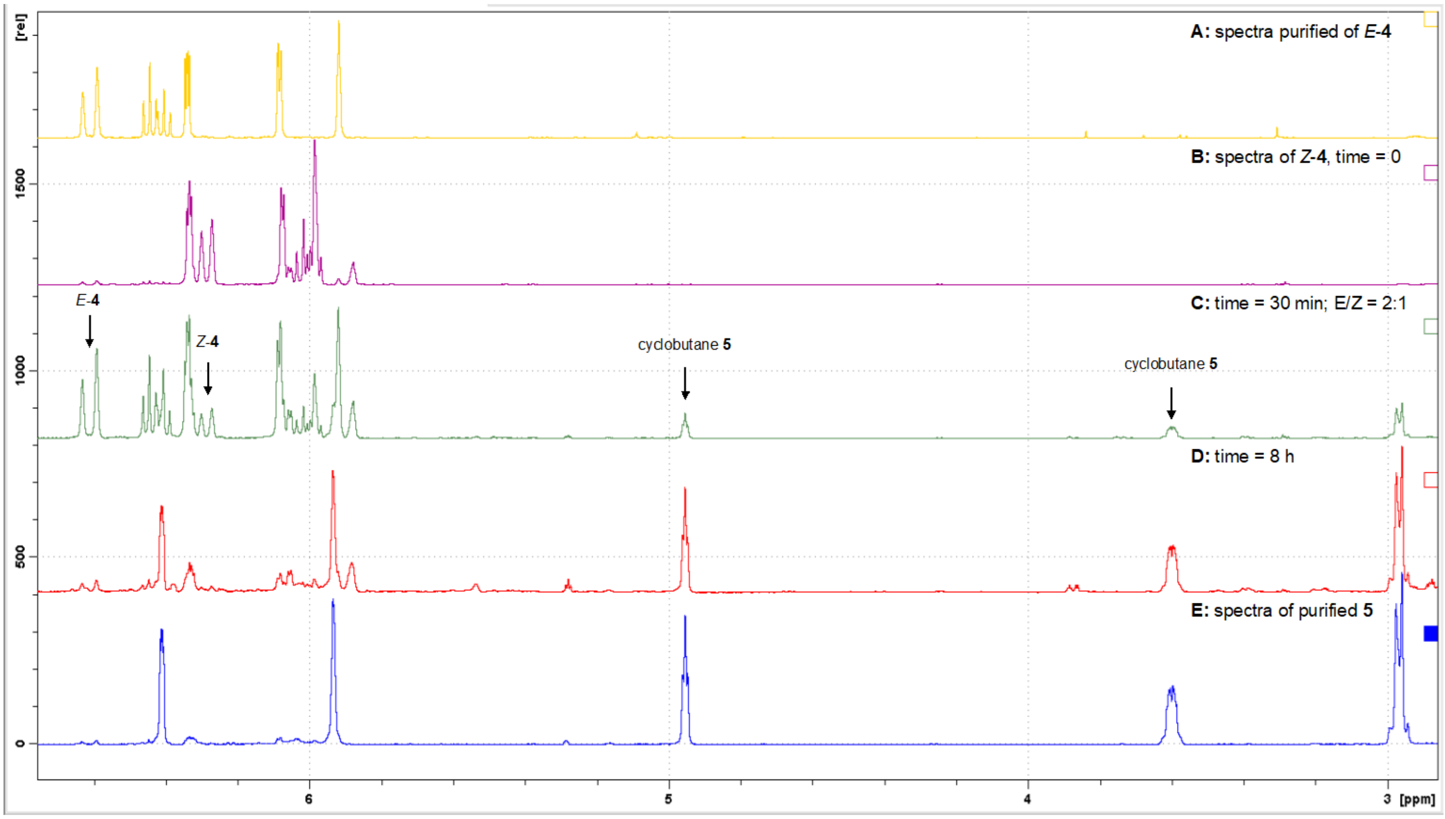

Procedure & Analysis: An NMR tube was charged with a solution of *Z*-**4** (6.5 mg, 0.04 mmol) in CD_3CN (0.45 mL). A ^{1}H NMR spectra of the reaction mixture was obtained under ambient conditions (panel **B**, above). The reaction mixture was then exposed to 350 nm UV-light at 35 °C in a Rayonet photoreactor. The unpurified reaction was analyzed by NMR after 30 min (panel **C**) and 8 h (panel **D**). Results were compared to ^{1}H NMR spectra of purified *E*-**4** (panel **A**) and **5** (panel **E**) in CD_3CN, respectively. We observed that *Z*-**4** readily equilibrated to a 2:1 mixture of *E*/*Z*-**4** within 30 min. During this time we also observed formation of **5**. The resulting equilibrium mixture of **4** slowly reacted to form **5** over the course of 8 h. This reaction proved to be highly stereoselective as product **5** was the only diastereomer observed.

5. ^{1}H NMR Experiment: Photodecomposition of **5** [see, Scheme 3B].

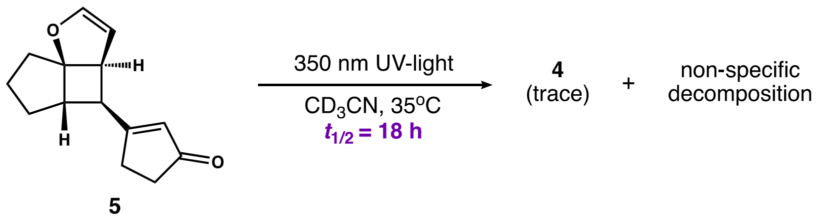

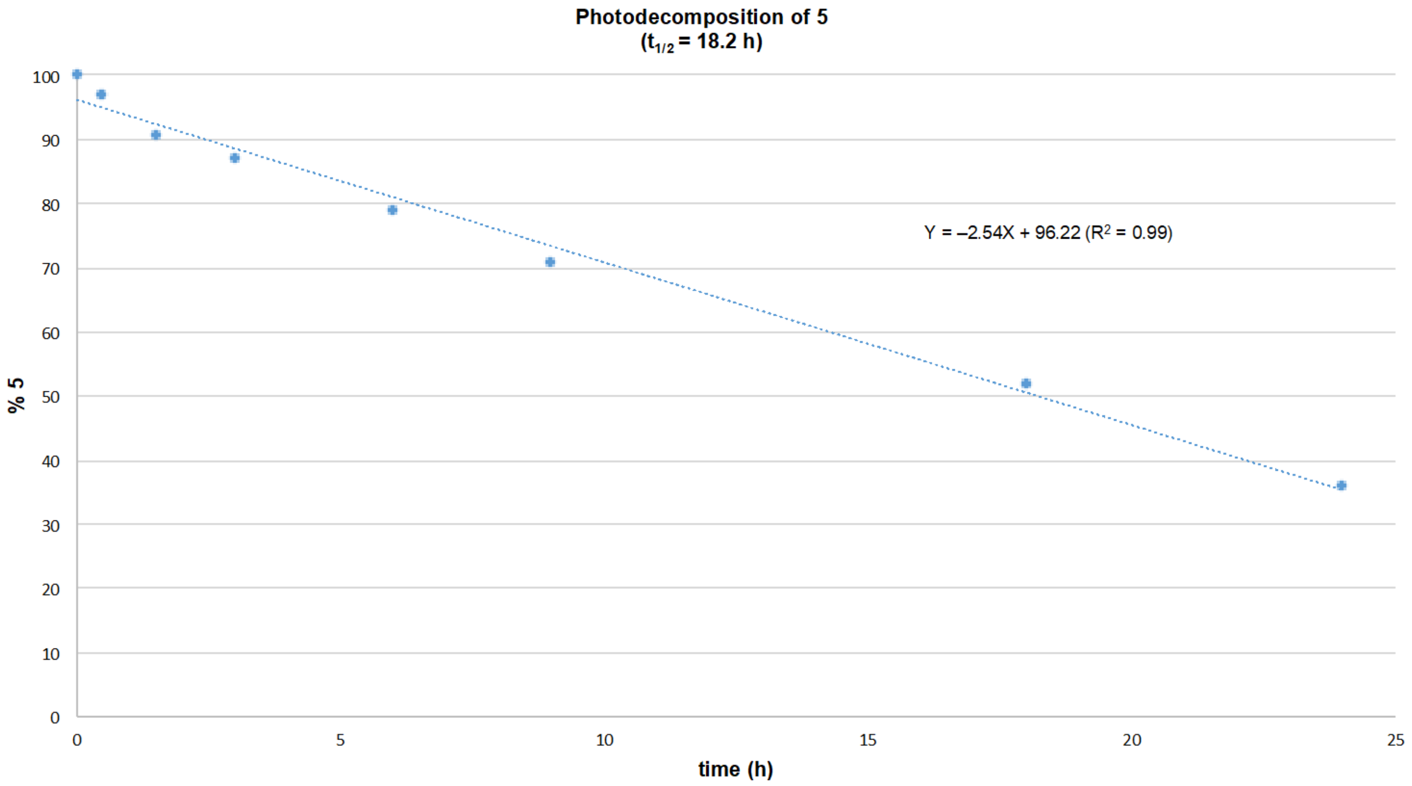

Procedure & Analysis: An NMR tube was charged with a solution of **5** (6.5 mg, 0.04 mmol) and *N*-benzylbenzamide (8.5 mg, 0.04 mmol, **internal standard**) in CD_3CN (0.45 mL). A ^{1}H NMR spectra of the reaction mixture was obtained under ambient conditions (time = 0, 100% **5**). The reaction mixture was then exposed to 350 nm UV-light at 35 ^{o}C in a Rayonet. Photodecomposition was determined by integration of **5** relative to an N-benzylbenzamide as an internal standard at various time points over a 24 period (see above). We determined a half-life ($t_{1/2}$) of 18.2 h for **5** under these reaction conditions.

6. [1]**H NMR Experiment:** Thermal isomerization of **5** to **3** [see, Scheme 3C].

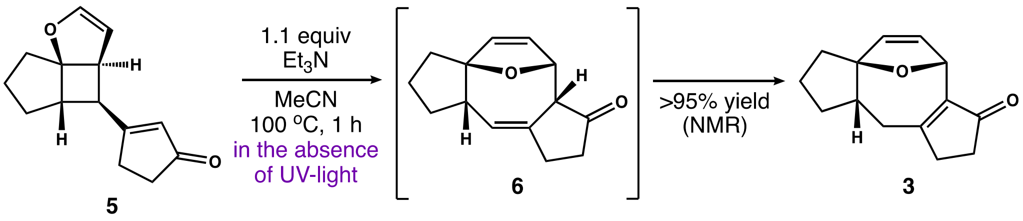

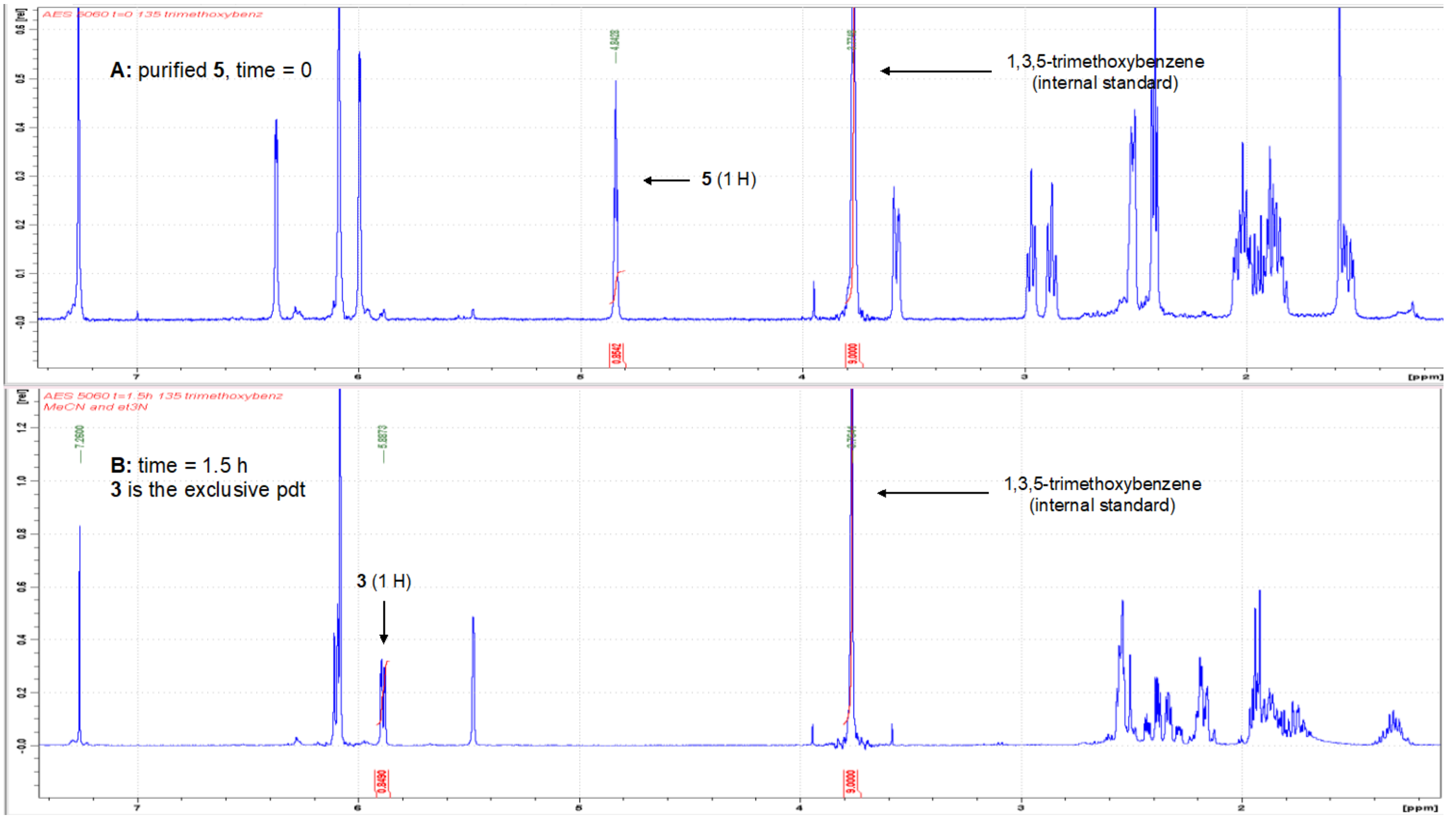

Procedure & Analysis: An NMR tube was charged with a solution of **5** (11 mg, 0.05 mmol) and 1,3,5-trimethoxybenzene (8.4 mg, 0.05 mmol, **internal standard**) in CH_3CN (0.8 mL). A ^{1}H NMR spectra of the reaction mixture was obtained under ambient conditions (400 mHz, $CDCl_3$, time = 0, 100% **5**, panel **A** above). Et_3N (0.01 mL, 0.06 mmol) was added and the reaction mixture was heated at 100 °C. After 1.5 h, the unpurified reaction mixture was analyzed by ^{1}H NMR (400 mHz, $CDCl_3$, panel **B**). We observed >95% NMR yield of **3.**

7. ^{1}H NMR Experiment: NMR yield for 13g [Figure 1].

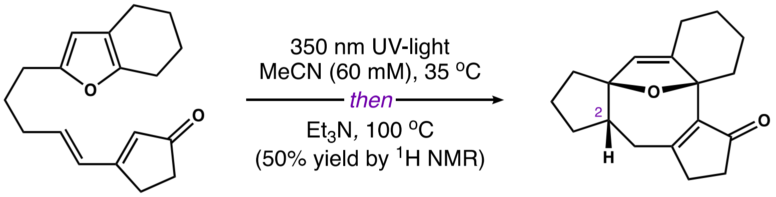

12g

13g
d.r. = 10:1 (at C_2)

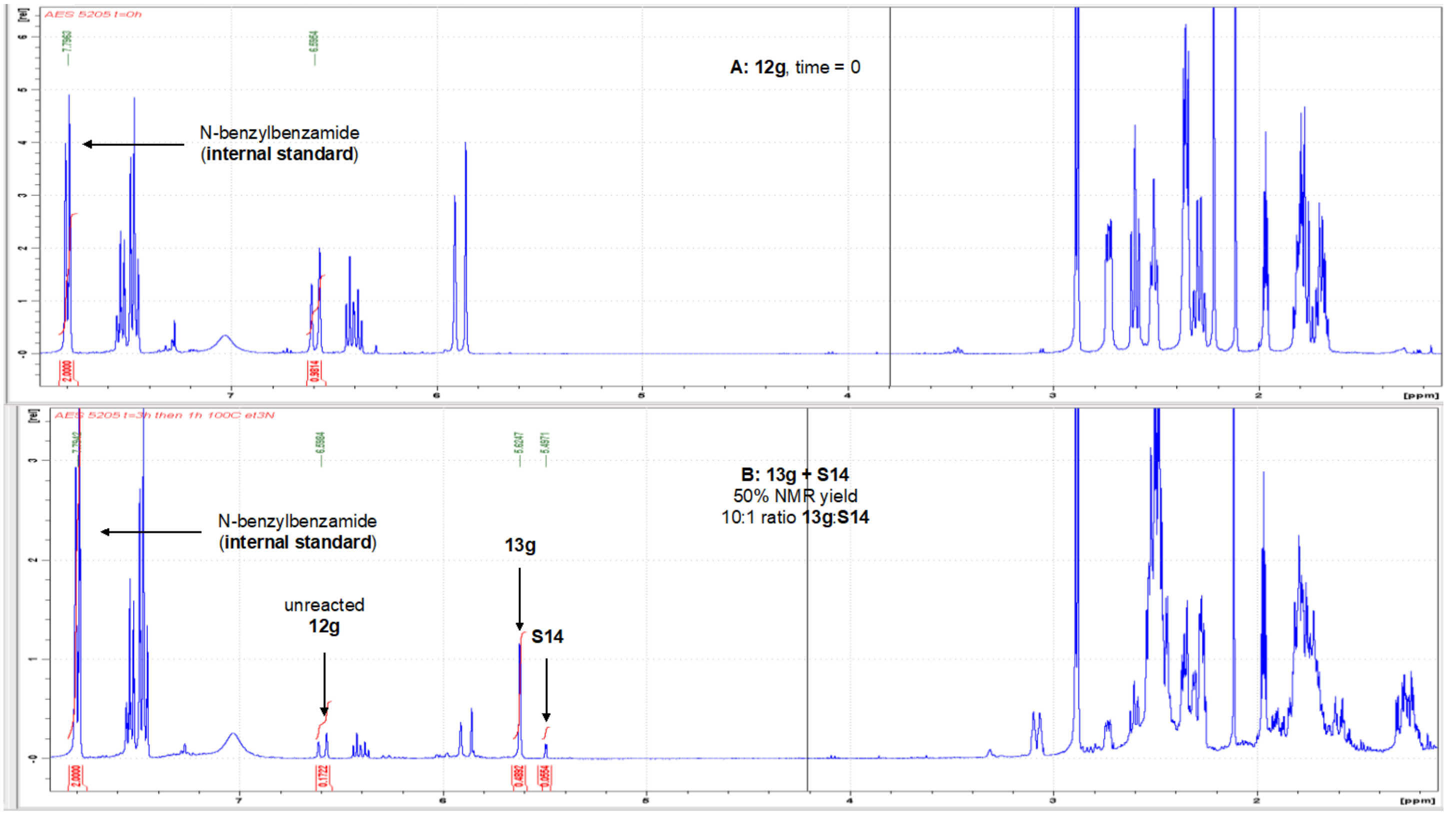

Procedure & Analysis: A quartz round-bottom flask was charged with a solution of **12g** (54 mg, 0.2 mmol) and N-benzylbenzamide (27 mg, 0.2 mmol, **internal standard**). An aliquot was taken via syringe to collect a ^{1}H NMR spectra under ambient conditions (400 mHz, $CDCl_3$, time = 0, 100% **12g**, panel **A** above). The reaction mixture was exposed to UV-light (*hv* = 350 nm) at 35 °C in a Rayonet. After 20 h, the reaction was removed from UV-light, cooled to rt, and treated with Et_3N (0.3 mL, 0.2 mmol). The reaction was heated to 100 °C. After 1 h, the reaction was cooled to rt. An aliquot was concentrated and analyzed by ^{1}H NMR (400 mHz, $CDCl_3$, panel **B**). This experiment established that **13g** was formed in 50% yield (by NMR) as a 10:1 mixture of diastereomers at C_2.

NMR yield for **13i** [Figure 1].

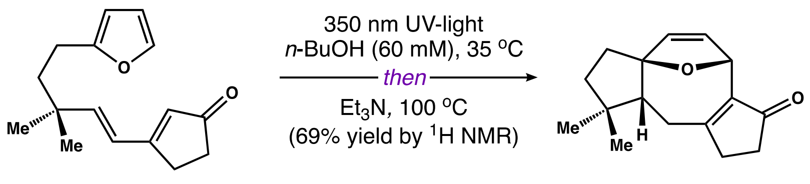

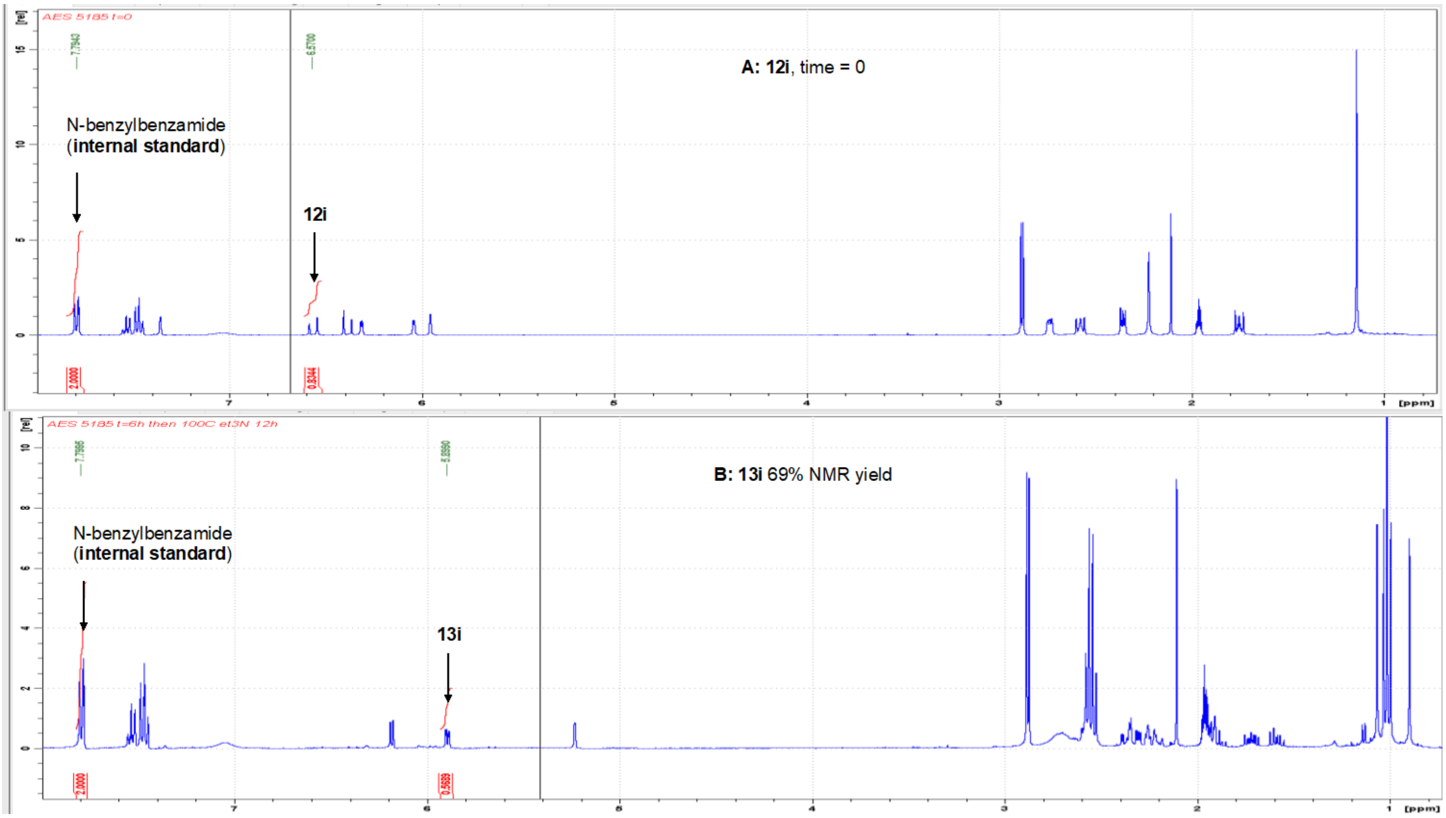

Procedure & Analysis: A quartz round-bottom flask was charged with a solution of **12i** (49 mg, 0.2 mmol) and N-benzylbenzamide (27 mg, 0.2 mmol, **internal standard**) in n-BuOH (3.3 mL). An aliquot was taken via syringe to collect a ^{1}H NMR spectra under ambient conditions (400 mHz, $CDCl_3$, time = 0, 100% **12i**, panel **A** above). The reaction mixture was exposed to UV-light (*hv* = 350 nm) at 35 °C in a Rayonet. After 9 h, the reaction was removed from UV-light, cooled to rt, and treated with Et_3N (0.3 mL, 0.2 mmol). The reaction was heated to 100 °C. After 9 h, the reaction was cooled to rt. An aliquot was concentrated and analyzed by ^{1}H NMR (400 mHz, $CDCl_3$, panel **B**). This experiment established that **13i** was formed in 69% yield (by NMR).

NMR yield for **13j** [Figure 1].

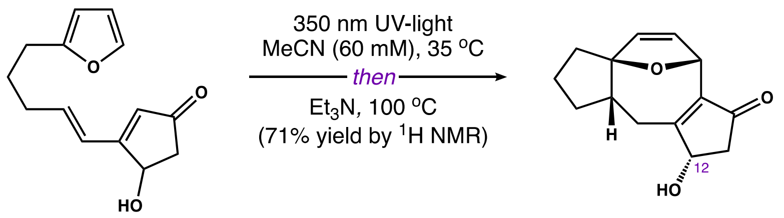

(±)-**12j**

(±)-**13j**
d.r. = 1.3:1 (at C_{12})

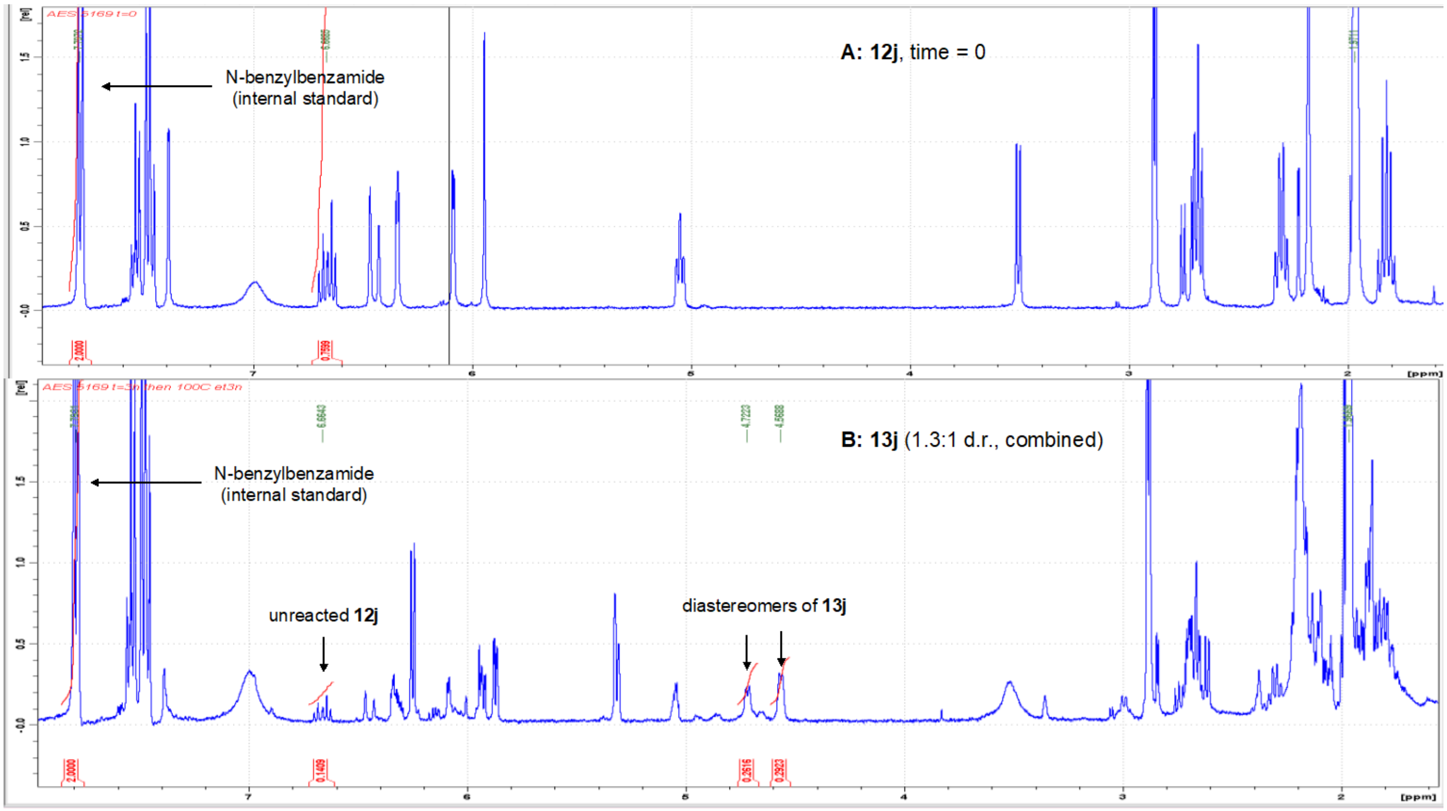

Procedure & Analysis: A quartz round-bottom flask was charged with a solution of **12j** (46 mg, 0.2 mmol) and N-benzylbenzamide (27 mg, 0.2 mmol, **internal standard**). An aliquot was taken via syringe to collect a ^{1}H NMR spectra under ambient conditions (400 mHz, $CDCl_3$, time = 0, 100% **12j**, panel **A** above). The reaction mixture was exposed to UV-light (*hv* = 350 nm) at 35 °C in a Rayonet. After 6 h, the reaction was removed from UV-light, cooled to rt, and treated with Et_3N (0.3 mL, 0.2 mmol). The reaction was heated to 100 °C. After 1 h, the reaction was cooled to rt. An aliquot was concentrated and analyzed by ^{1}H NMR (400 mHz, $CDCl_3$, panel **B**). This experiment established that **13j** was formed in 71% yield (by NMR) as a 1.3:1 mixture of diastereomers at C_{12}.

NMR yield for **13h** [Figure 1].

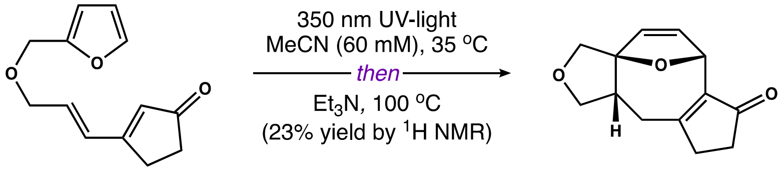

12h **13h**

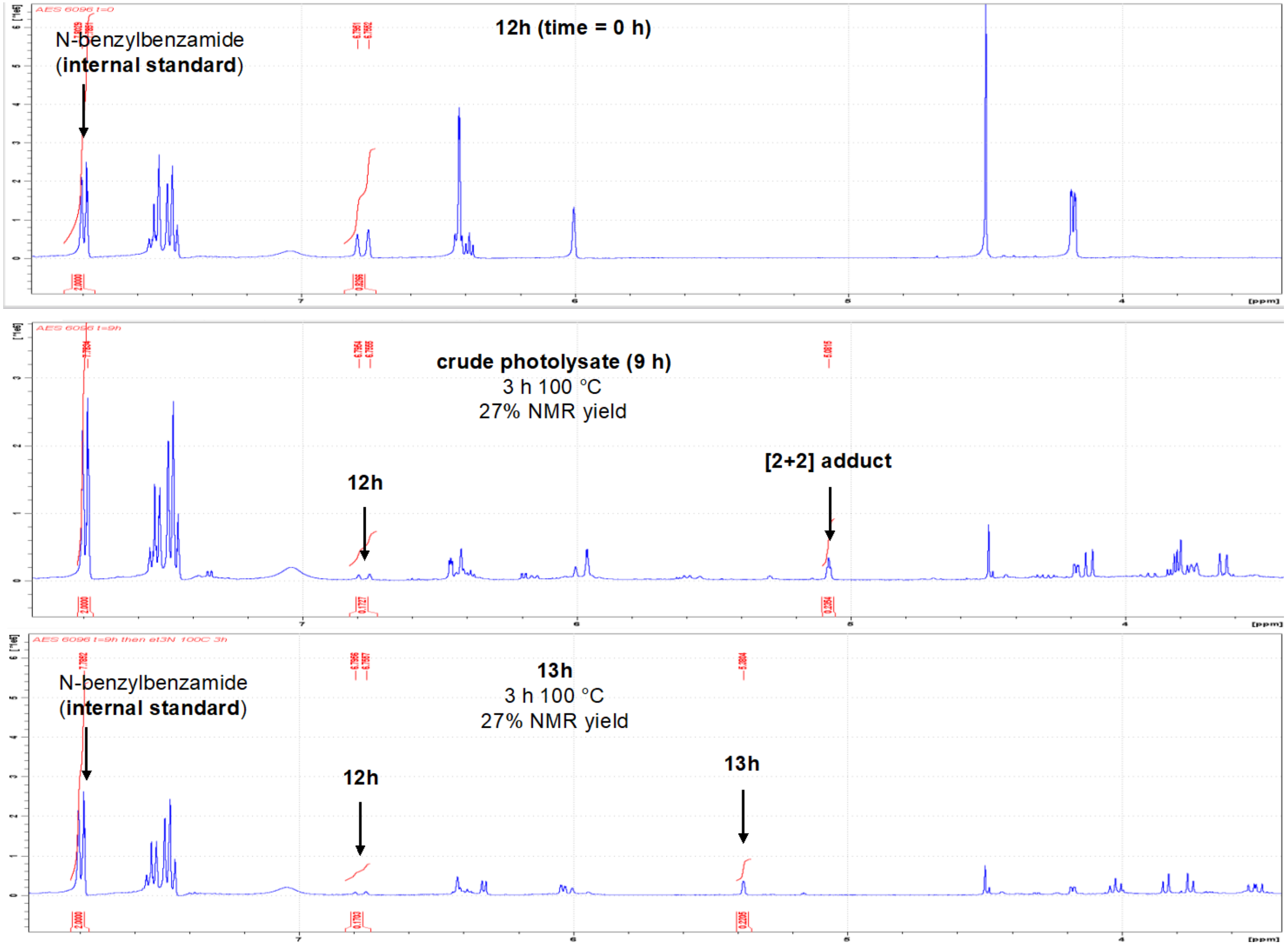

Procedure & Analysis: A quartz round-bottom flask was charged with a solution of **12h** (44 mg, 0.2 mmol) and N-benzylbenzamide (27 mg, 0.2 mmol, **internal standard**). Aliquots were taken via syringe to collect ^1H NMR spectra under ambient conditions (400 mHz, $CDCl_3$, time = 0, 100% **12h**, panel **A** above). The reaction mixture was exposed to UV-light (*hv* = 350 nm) at 35 °C in a Rayonet. After 9 h, the reaction was removed from UV-light cooled to rt, and and analyzed by ^1H NMR (panel **B**). The mixture was then treated with Et_3N (0.3 mL, 0.2 mmol) and heated to 100 °C. After 3 h, the reaction was cooled to rt. An aliquot was concentrated and analyzed by ^1H NMR (400 mHz, $CDCl_3$, panel **C**). This experiment established that **13h** was formed in 27% yield (by NMR).

8. Photochemical reaction setup and analysis of quartz vs. Pyrex glassware.

Pictures of reaction setup in the Rayonet are provided below. Reactions described in Tables 1 and 2 were carried out in quartz flasks as described in the manuscript; however, the use of quartz is not required. As shown in Table S1, Pyrex glassware affords identical results (+/- 3% yield, experiments repeated in duplicate) for the photoisomerization of substrate **4** to rearranged cyclooctadiene **3**.

Reaction setup – 0.5 mmol scale

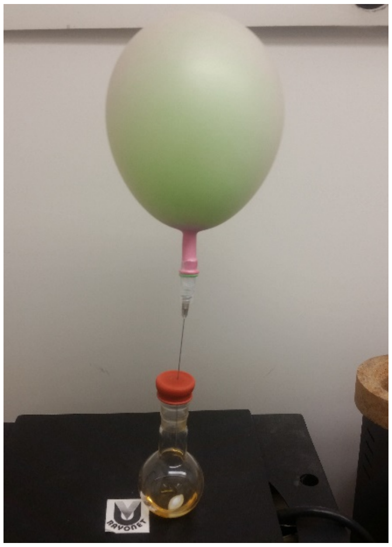

A

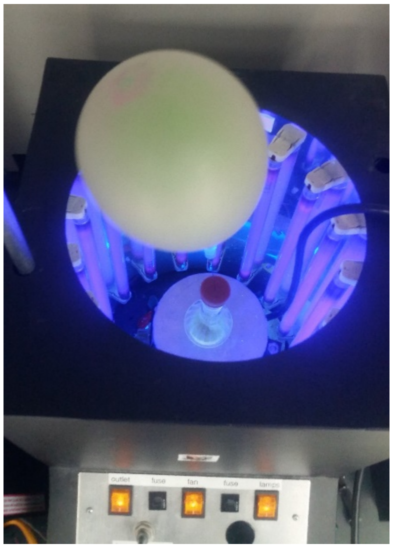

B

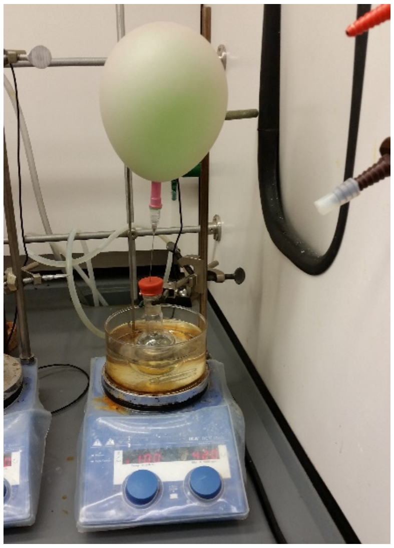

C

Reaction setup – gram scale

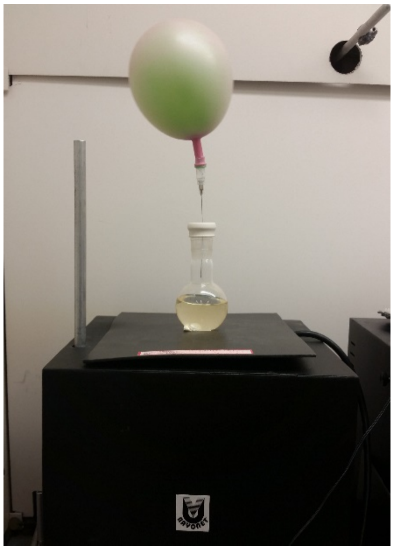

A

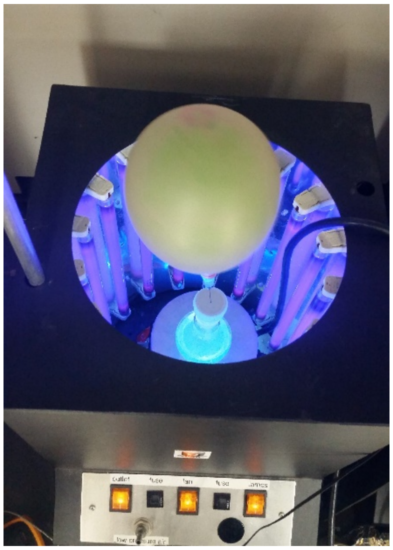

B

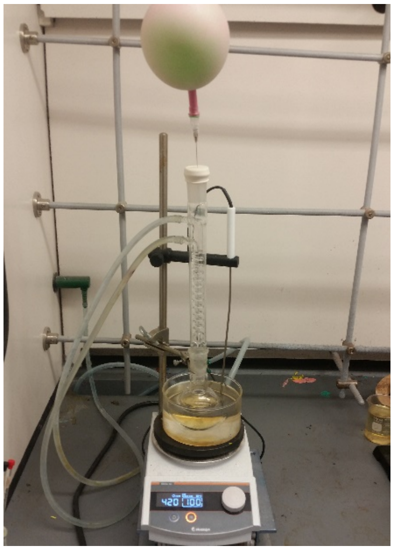

C

A = Flat-bottom quartz flask charged with photosubstrate in MeCN. The reaction was maintained under an atmosphere of N_2.
B = The reaction vessel was placed on a stir plate within the Rayonet and irradiated using 24 W lamps for the indicated period of time.
C = The reaction flask was removed from the Rayonet and directly added to a pre-heated oil bath for the indicated period of time. On larger scale, it was necessary to connect a reflux condenser.

Table S1. Direct comparison of quartz vs. Pyrex glassware[a]

entry	glassware	irradiation time	ratio of **4:5**[b]	yield **3** (%)[c]	recovered **4** (%)
1	quartz	12 h	1:2.6	51	26
2	Pyrex	12 h	1:2.3	48	17

[a] Photochemistry was carried out using 24 W UV-lamps as described above. Reactions were preformed on 0.5 mmol scale utilizing photosubstrate **4** in either a 50 mL quartz flask (entry 1) or 50 mL Pyrex flask of equal dimensions (entry 2).[b] Product ratio of E/Z-**4** and [2+2] photoadduct **5** after exposure to 350 nm UV-light for 12 h as determined by ^{1}H NMR of the unpurified photolysate (400 MHz, CD_3CN). Values reported as the average for duplicate experiments. [c] Isolated yield of **3** upon warming the crude photolysate to reflux for 6 h. Purification was preformed on neutralized SiO_2 as described above. Values reported as the average for duplicate experiments.

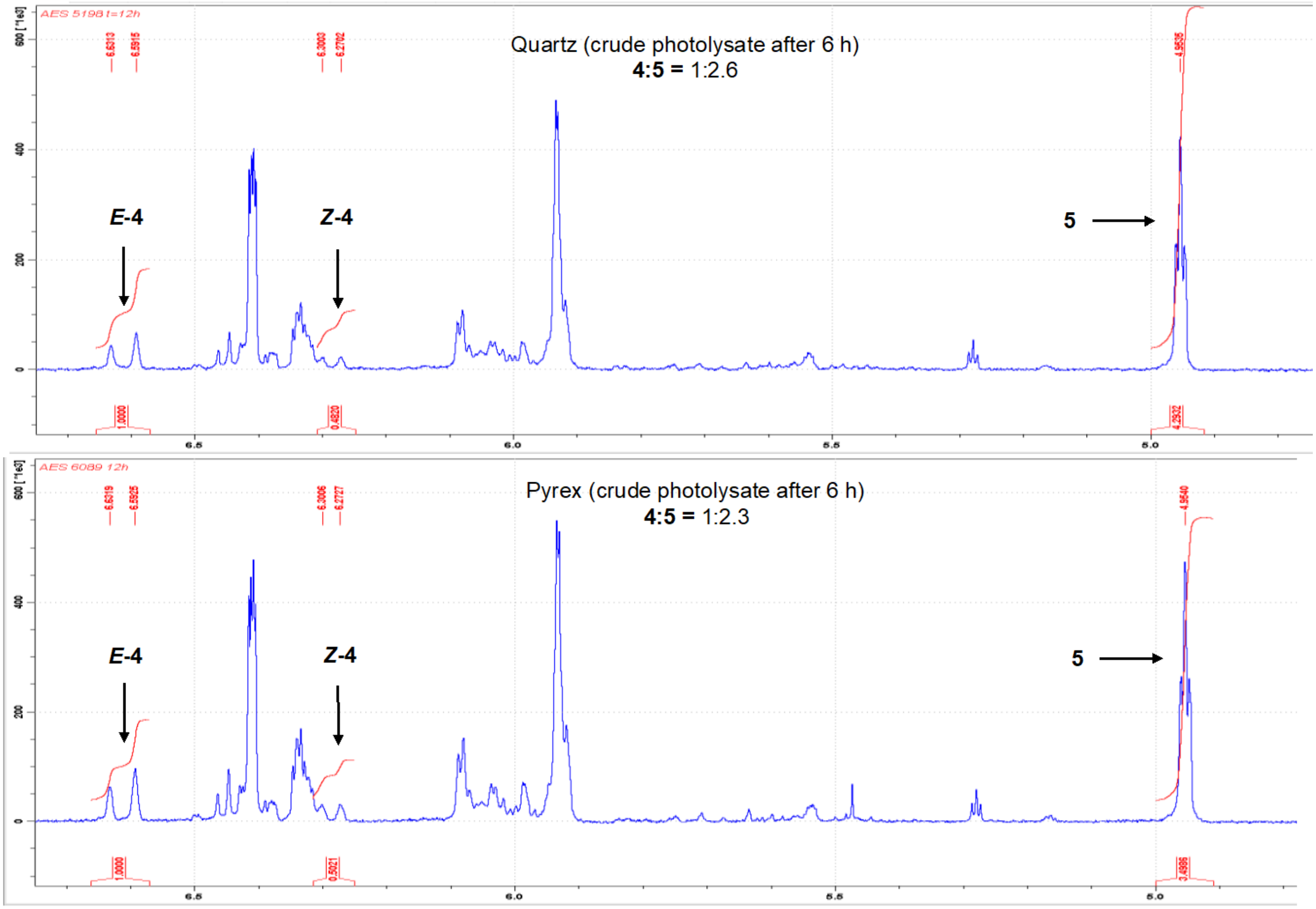

9. SI References.

[1] Mukiayama, T.; Matsuo, J.; Kitagawa, H. *Chem. Lett.* **2000**, *29*, 1350.

[2] Compound **8** and several other furyl-alkynes reported herein were volatile. Special care should be used to prevent loss of product on the Hi-Vac.

[3] Yamamoto, H.; Sasaki, I.; Imagawa, H.; Nishizawa, M. *Org. Lett.* **2007**, *9*, 1399.

[4] Lautens, M.; Fillion, E. *J. Org. Chem.* **1997**, *62*, 4418.

[5] Comins, D. L.; Killpack, M. O. *J. Org. Chem.* **1987**, *52*, 104.

[6] Martín-Matute, B.; Nevado, C.; Cárdenas, D. J.; Echavarren, A. M. *J. Am. Chem. Soc.* **2003**, *125*, 5757.

[7] Horn, E. J.; Silverston, J. S.; Vanderwal, C. D. *J. Org. Chem.* **2016**, *81*, 1819.

[8] Heaney, H.; Papageorgiou, G.; Wilkins, R. F. *Tetrahedron Lett.* **1988**, *29*, 2377.

[9] Nagarajan, R.; Magesh, C. J.; Perumal, P. T. *Synthesis* **2004**, 69.

[10] Rank, E.; Brückner, R. *Eur. J. Org. Chem.* **1998**, *6*, 1045.

[11] Wencel-Delord, J.; Alexakis, A.; Crévisy, C.; Mauduit, M. *Org. Lett.* **2010**, *12*, 4335.

[12] X-ray diffraction data have been deposited at the Cambridge Crystallographic Data Center (CCDC) and can be obtained free of charge at www.ccdc.cam.ac.uk.

[13] SiO_2 was saturated in pH 7 buffer and allowed to sit for 10 min. The resulting SiO_2 slurry was concentrated under reduced pressure on a rotovap to remove excess buffer then dried on the Hi-Vac overnight.

[14] The best yields were obtained when **3** was dried by azeoptroic removal of residual water using toluene prior to use. This was particularly important when samples of **3** stored in the freezer were used.

[15] This quench was modified from a related procedure developed by Baran and co-workers, see: Mendoza, A.; Ishihara, Y.; Baran, P. S. *Nat. Chem.* **2012**, *4*, 21.

[16] Compound **14** undergoes extensive hydrolysis when passed over SiO_2; however, we observed that material prepared in this way was stable for long periods of time (>5 months) when stored neat at –20 °C.

NAME	JL2262a 1H
EXPNO	1
PROCNO	1
Date_	20170228
Time	23.58
INSTRUM	spect
PROBHD	5 mm PABBO BB-
PULPROG	zg30
TD	65536
SOLVENT	CDCl3
NS	8
DS	2
SWH	8223.685 Hz
FIDRES	0.125483 Hz
AQ	3.9846387 sec
RG	203
DW	60.800 usec
DE	6.50 usec
TE	293.9 K
D1	1.00000000 sec
TD0	1

======== CHANNEL f1 ========

NUC1	1H
P1	10.00 usec
PL1	-2.78 dB
PL1W	27.10892296 W
SFO1	400.1724712 MHz
SI	32768
SF	400.1700083 MHz
WDW	EM
SSB	0
LB	0.30 Hz
GB	0
PC	1.00

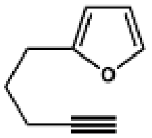

8: $C_9H_{10}O$
MW: 134.18

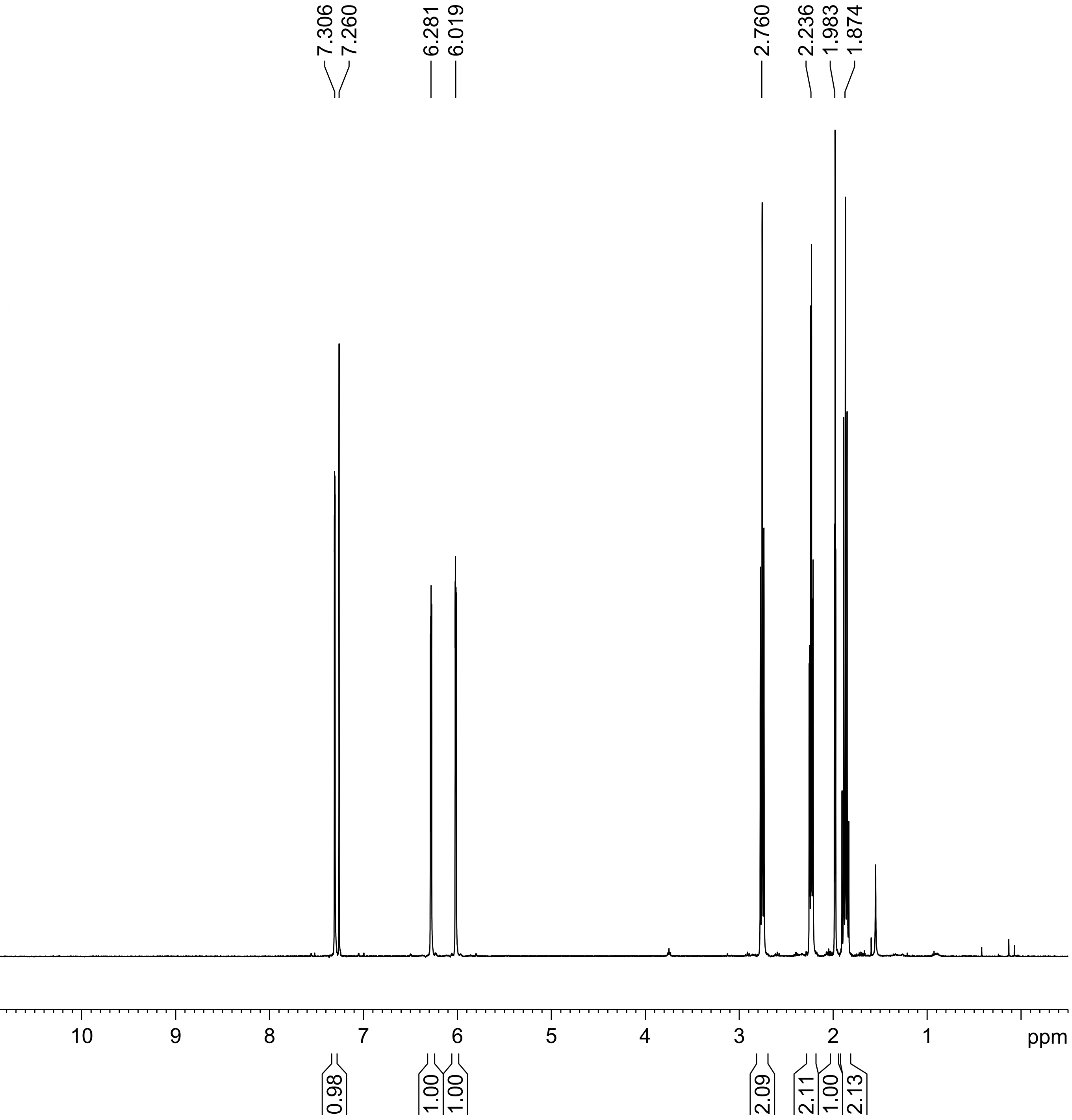

NAME JL2265a 1H
EXPNO 1
PROCNO 1
Date_ 20170303
Time 10.54
INSTRUM spect
PROBHD 5 mm PABBO BB-
PULPROG zg30
TD 65536
SOLVENT CDCl3
NS 8
DS 2
SWH 8223.685 Hz
FIDRES 0.125483 Hz
AQ 3.9846387 sec
RG 203
DW 60.800 usec
DE 6.50 usec
TE 292.8 K
D1 1.00000000 sec
TD0 1

======== CHANNEL f1 ========
NUC1 1H
P1 10.00 usec
PL1 -2.78 dB
PL1W 27.10892296 W
SFO1 400.1724712 MHz
SI 32768
SF 400.1700088 MHz
WDW EM
SSB 0
LB 0.30 Hz
GB 0
PC 1.00

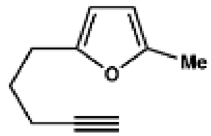

S1: $C_{10}H_{12}O$
MW: 148.21

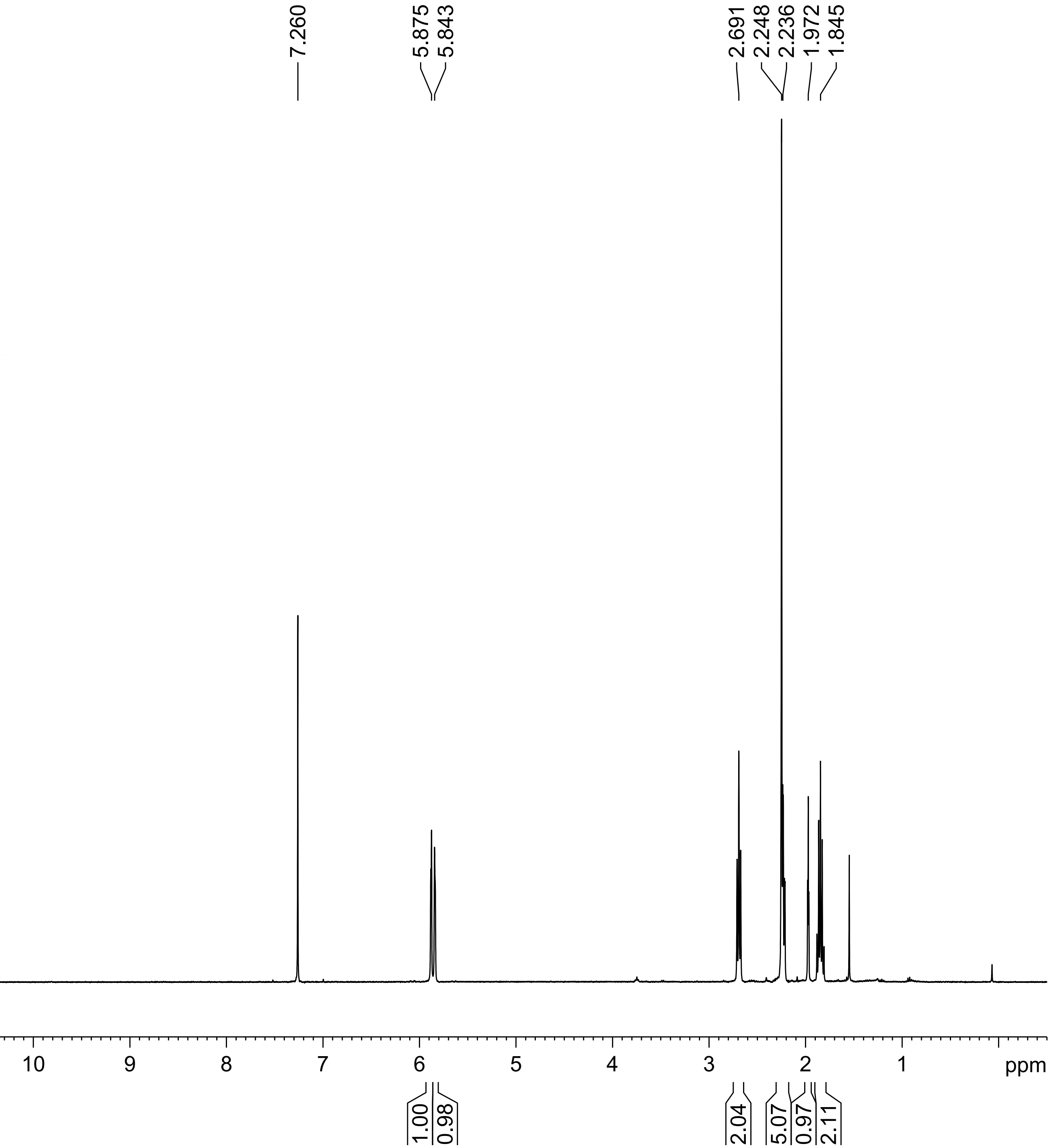

NAME JL2281cd 1H
EXPNO 2
PROCNO 1
Date_ 20170312
Time 20.27
INSTRUM spect
PROBHD 5 mm PABBO BB-
PULPROG zg30
TD 65536
SOLVENT CDCl3
NS 8
DS 2
SWH 8223.685 Hz
FIDRES 0.125483 Hz
AQ 3.9846387 sec
RG 203
DW 60.800 usec
DE 6.50 usec
TE 292.9 K
D1 1.00000000 sec
TD0 1

======== CHANNEL f1 ========
NUC1 1H
P1 10.00 usec
PL1 -2.78 dB
PL1W 27.10892296 W
SFO1 400.1724712 MHz
SI 32768
SF 400.1700088 MHz
WDW EM
SSB 0
LB 0.30 Hz
GB 0
PC 1.00

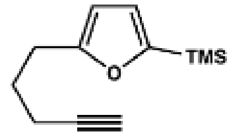

S2: $C_{12}H_{18}OSi$
MW: 206.36

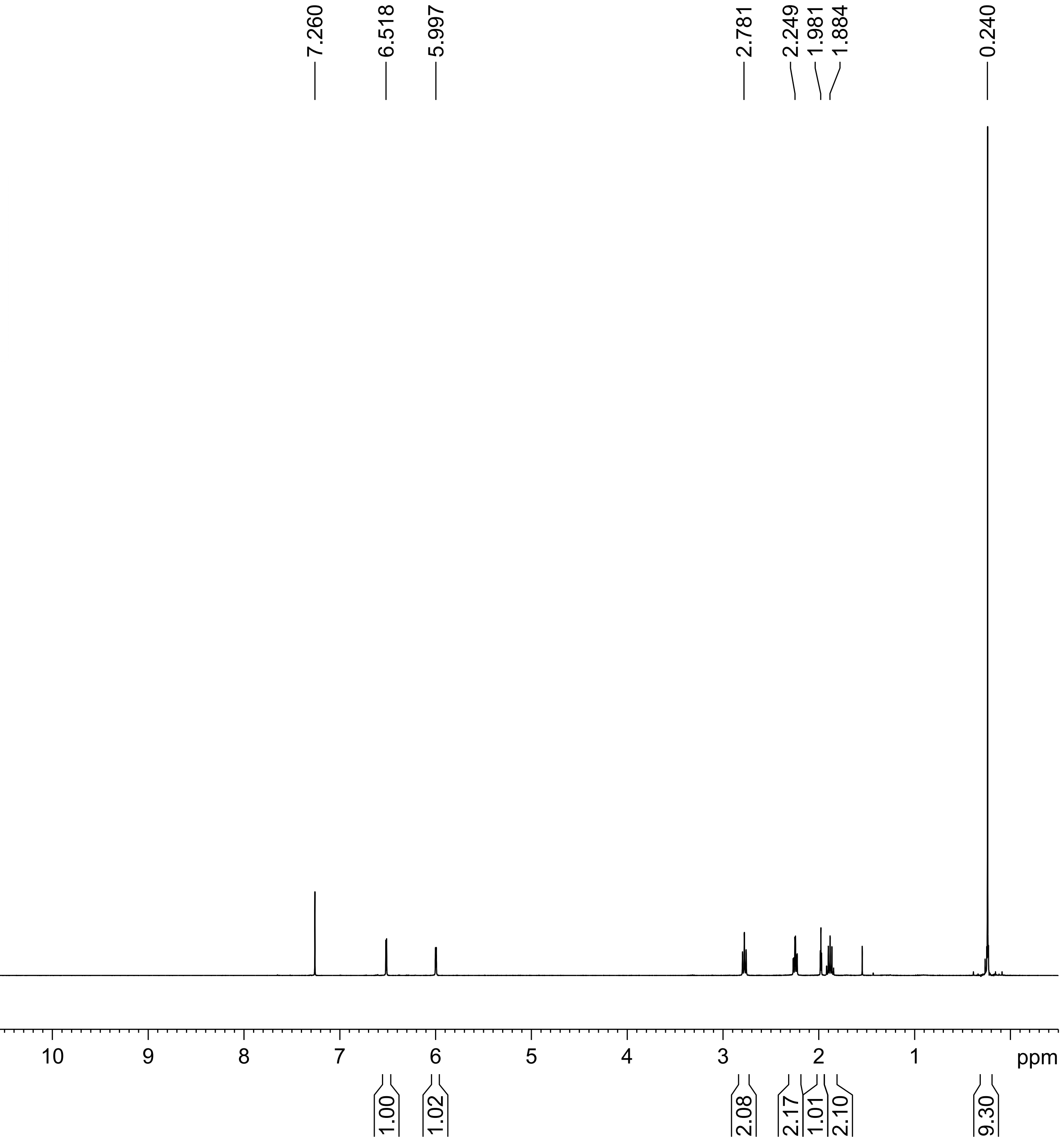

NAME JL2281cd 13C
EXPNO 1
PROCNO 1
Date_ 20170312
Time 22.01
INSTRUM spect
PROBHD 5 mm PABBO BB-
PULPROG zgpg30
TD 65536
SOLVENT CDCl3
NS 34
DS 4
SWH 24038.461 Hz
FIDRES 0.366798 Hz
AQ 1.3631988 sec
RG 203
DW 20.800 usec
DE 6.50 usec
TE 293.2 K
D1 2.00000000 sec
D11 0.03000000 sec
TD0 1

======== CHANNEL f1 ========
NUC1 13C
P1 10.00 usec
PL1 -1.78 dB
PL1W 47.30532455 W
SFO1 100.6328888 MHz

======== CHANNEL f2 ========
CPDPRG2 waltz16
NUC2 1H
PCPD2 80.00 usec
PL2 -2.78 dB
PL12 15.28 dB
PL13 18.00 dB
PL2W 27.10892296 W
PL12W 0.42375252 W
PL13W 0.22652298 W
SFO2 400.1716007 MHz
SI 32768
SF 100.6228270 MHz
WDW EM
SSB 0
LB 1.00 Hz
GB 0
PC 1.40

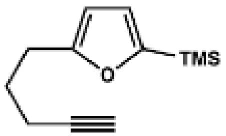

S2: $C_{12}H_{18}OSi$
MW: 206.36

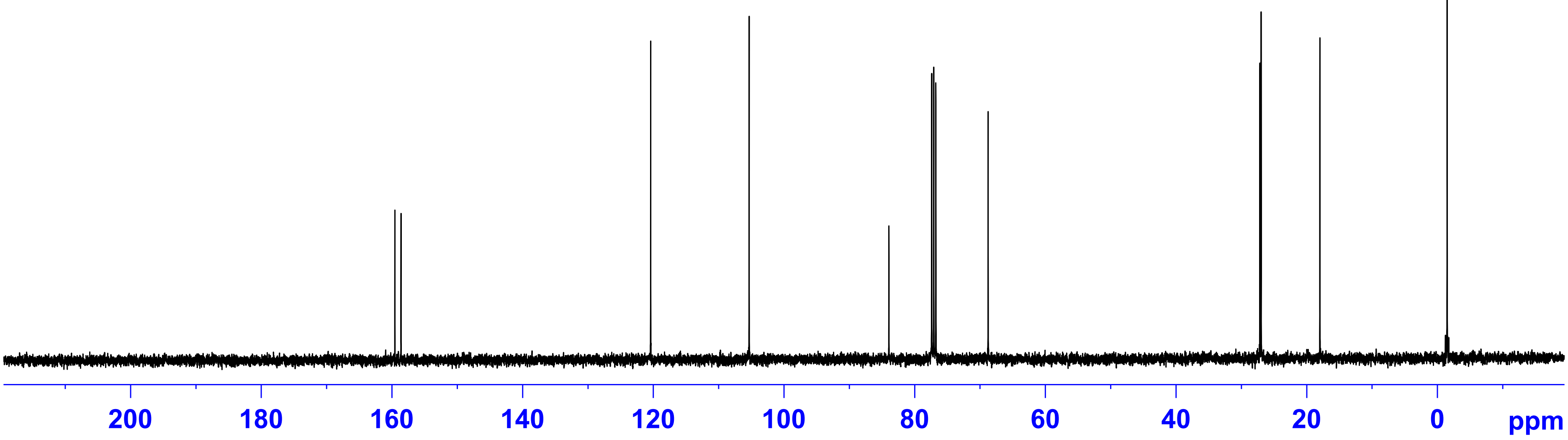

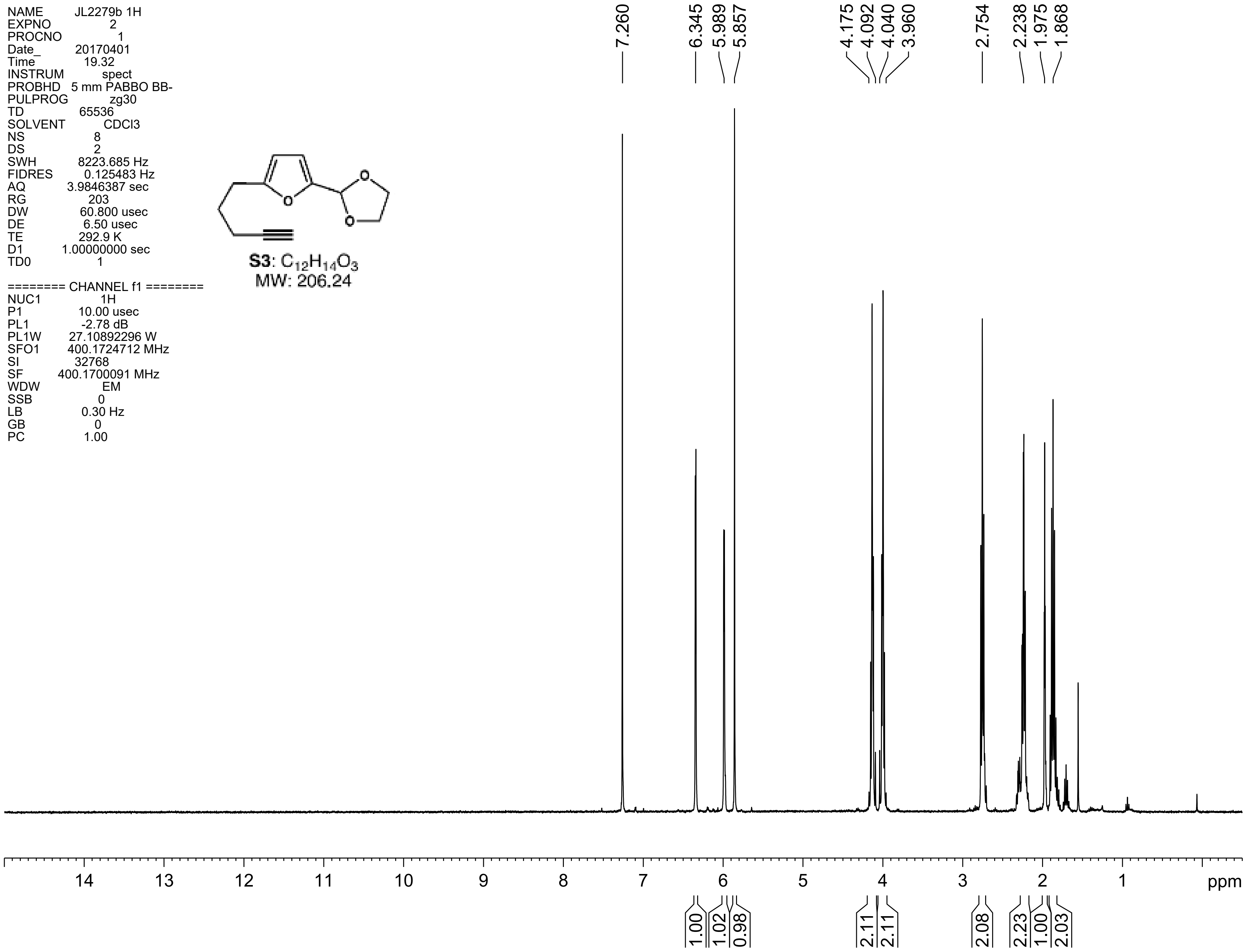
NAME JL2279b 1H
EXPNO 2
PROCNO 1
Date_ 20170401
Time 19.32
INSTRUM spect
PROBHD 5 mm PABBO BB-
PULPROG zg30
TD 65536
SOLVENT CDCl3
NS 8
DS 2
SWH 8223.685 Hz
FIDRES 0.125483 Hz
AQ 3.9846387 sec
RG 203
DW 60.800 usec
DE 6.50 usec
TE 292.9 K
D1 1.00000000 sec
TD0 1
======== CHANNEL f1 ========
NUC1 1H
P1 10.00 usec
PL1 -2.78 dB
PL1W 27.10892296 W
SFO1 400.1724712 MHz
SI 32768
SF 400.1700091 MHz
WDW EM
SSB 0
LB 0.30 Hz
GB 0
PC 1.00
S3: C12H14O3
MW: 206.24
7.260
6.345
5.989
5.857
4.175
4.092
4.040
3.960
2.754
2.238
1.975
1.868
14
13
12
11
10
9
8
7
6
5
4
3
2
1
ppm
1.00
1.02
0.98
2.11
2.11
2.08
2.23
1.00
2.03

NAME JL2279b 13C
EXPNO 1
PROCNO 1
Date_ 20170314
Time 1.27
INSTRUM spect
PROBHD 5 mm PABBO BB-
PULPROG zgpg30
TD 65536
SOLVENT CDCl3
NS 33
DS 4
SWH 24038.461 Hz
FIDRES 0.366798 Hz
AQ 1.3631988 sec
RG 203
DW 20.800 usec
DE 6.50 usec
TE 293.3 K
D1 2.00000000 sec
D11 0.03000000 sec
TD0 1

======== CHANNEL f1 ========
NUC1 13C
P1 10.00 usec
PL1 -1.78 dB
PL1W 47.30532455 W
SFO1 100.6328888 MHz

======== CHANNEL f2 ========
CPDPRG2 waltz16
NUC2 1H
PCPD2 80.00 usec
PL2 -2.78 dB
PL12 15.28 dB
PL13 18.00 dB
PL2W 27.10892296 W
PL12W 0.42375252 W
PL13W 0.22652298 W
SFO2 400.1716007 MHz
SI 32768
SF 100.6228270 MHz
WDW EM
SSB 0
LB 1.00 Hz
GB 0
PC 1.40

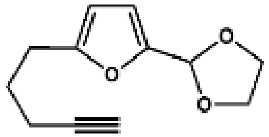

S3: $C_{12}H_{14}O_3$
MW: 206.24

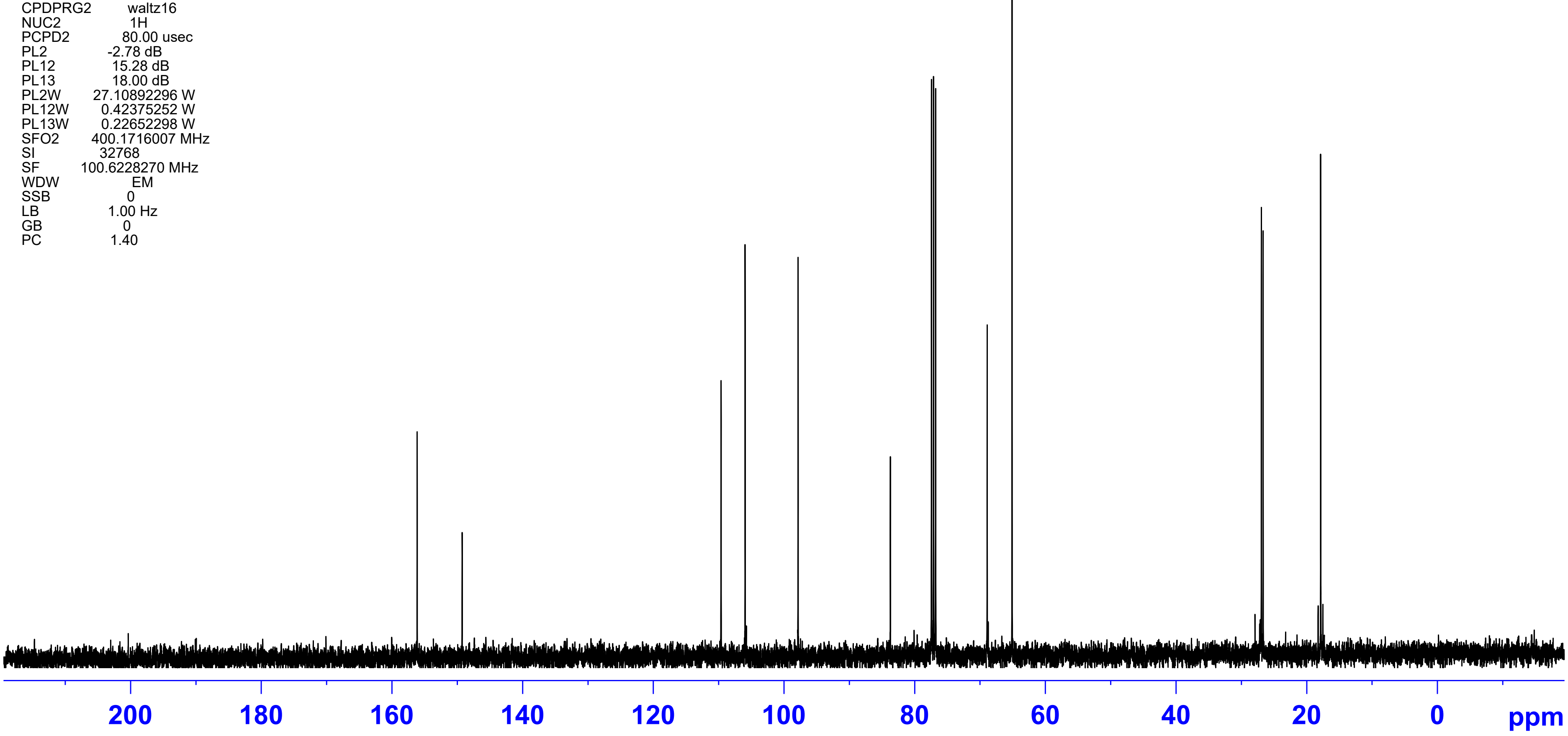

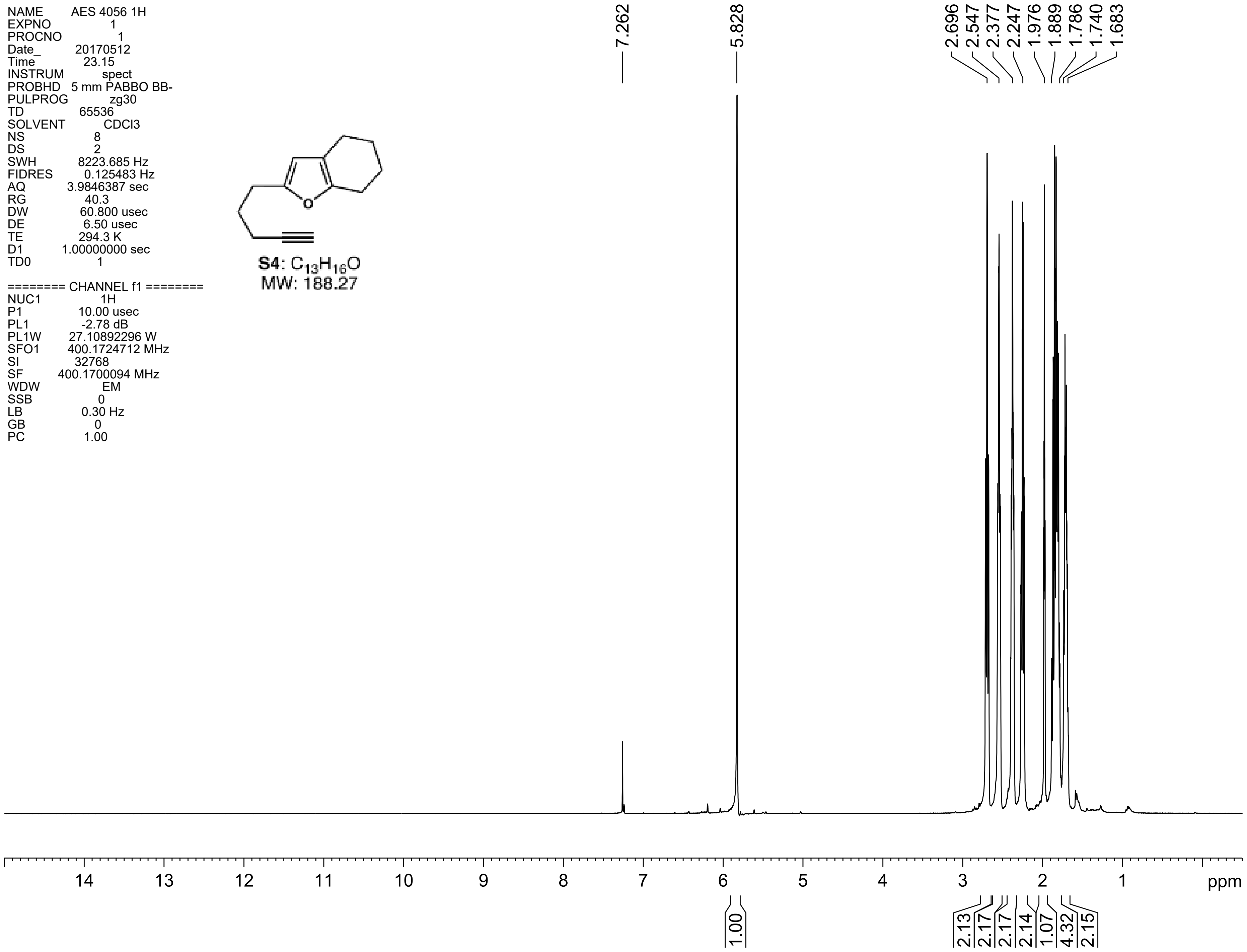

S4: $C_{13}H_{16}O$
MW: 188.27

NAME AES 4056 C13
EXPNO 1
PROCNO 1
Date_ 20170512
Time 23.33
INSTRUM spect
PROBHD 5 mm PABBO BB-
PULPROG zgpg30
TD 65536
SOLVENT CDCl3
NS 256
DS 4
SWH 24038.461 Hz
FIDRES 0.366798 Hz
AQ 1.3631988 sec
RG 203
DW 20.800 usec
DE 6.50 usec
TE 295.3 K
D1 2.00000000 sec
D11 0.03000000 sec
TD0 1

======== CHANNEL f1 ========
NUC1 13C
P1 10.00 usec
PL1 -1.78 dB
PL1W 47.30532455 W
SFO1 100.6328888 MHz

======== CHANNEL f2 ========
CPDPRG2 waltz16
NUC2 1H
PCPD2 80.00 usec
PL2 -2.78 dB
PL12 15.28 dB
PL13 18.00 dB
PL2W 27.10892296 W
PL12W 0.42375252 W
PL13W 0.22652298 W
SFO2 400.1716007 MHz
SI 32768
SF 100.6228270 MHz
WDW EM
SSB 0
LB 1.00 Hz
GB 0
PC 1.40

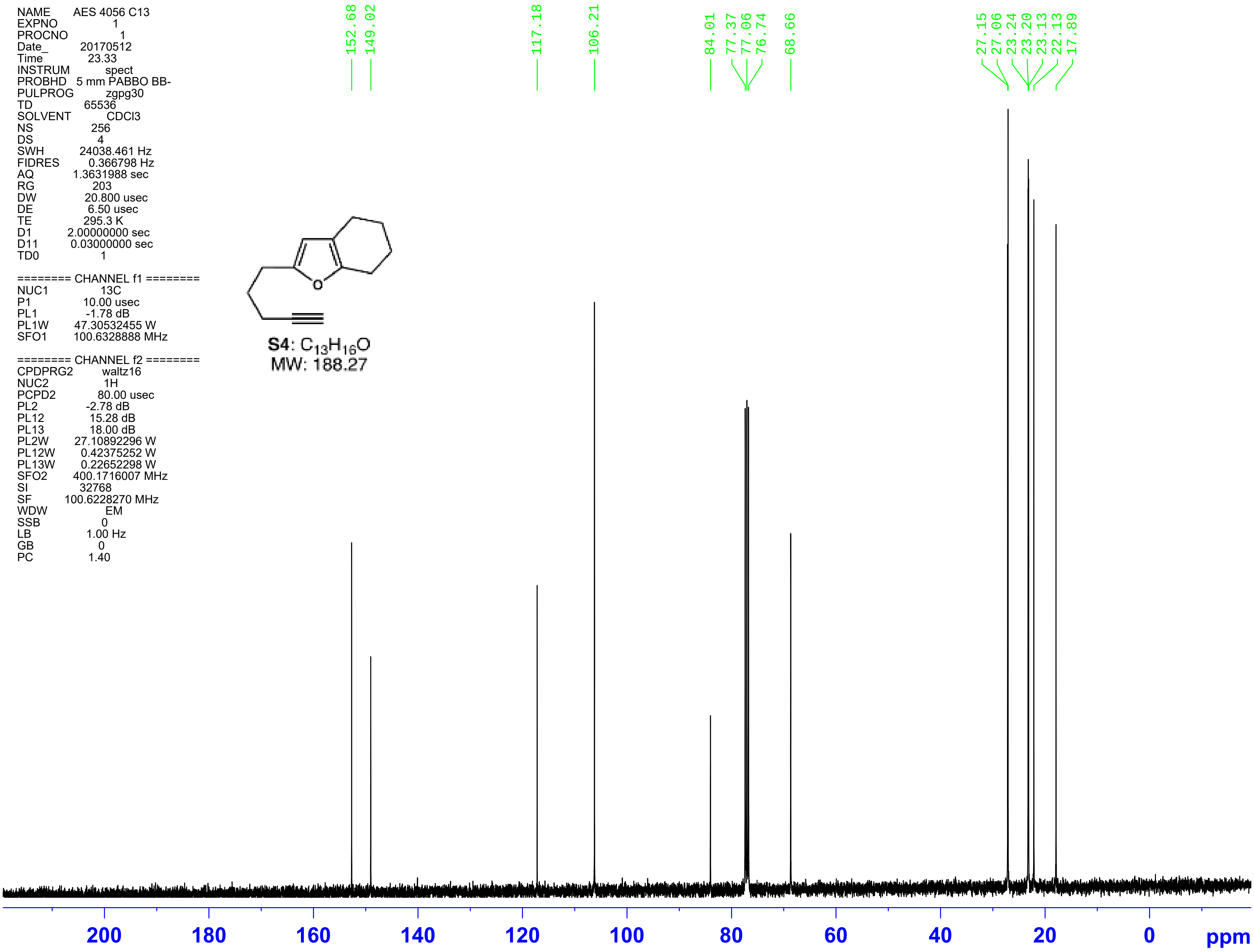

NAME JL3007a 1H
EXPNO 2
PROCNO 1
Date_ 20170412
Time 14.10
INSTRUM spect
PROBHD 5 mm PABBO BB-
PULPROG zg30
TD 65536
SOLVENT CDCl3
NS 8
DS 2
SWH 8223.685 Hz
FIDRES 0.125483 Hz
AQ 3.9846387 sec
RG 101
DW 60.800 usec
DE 6.50 usec
TE 293.2 K
D1 1.00000000 sec
TD0 1

======== CHANNEL f1 ========
NUC1 1H
P1 10.00 usec
PL1 -2.78 dB
PL1W 27.10892296 W
SFO1 400.1724712 MHz
SI 32768
SF 400.1700088 MHz
WDW EM
SSB 0
LB 0.30 Hz
GB 0
PC 1.00

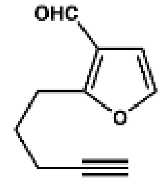

S5: $C_{10}H_{10}O_2$
MW: 162.19

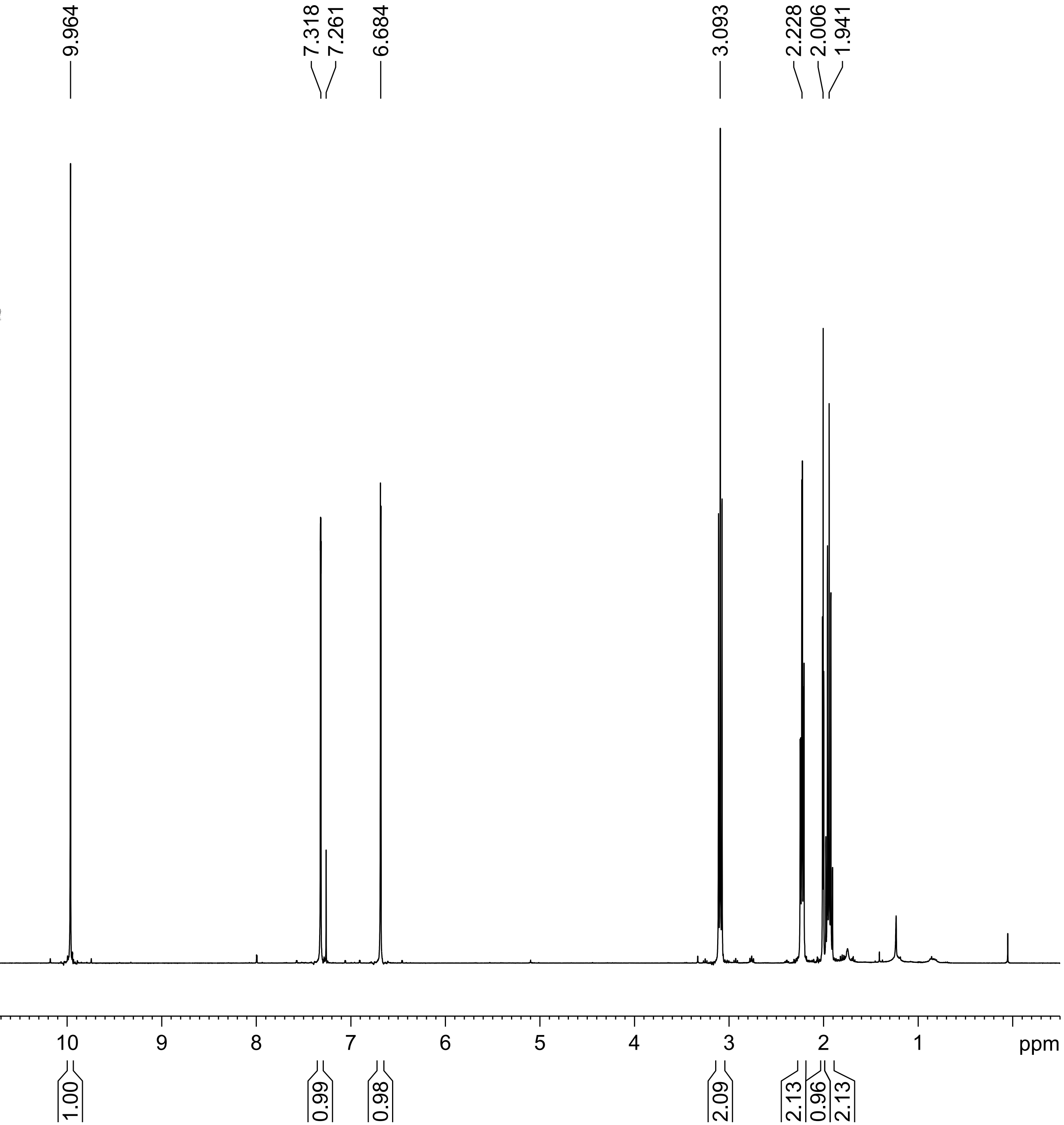

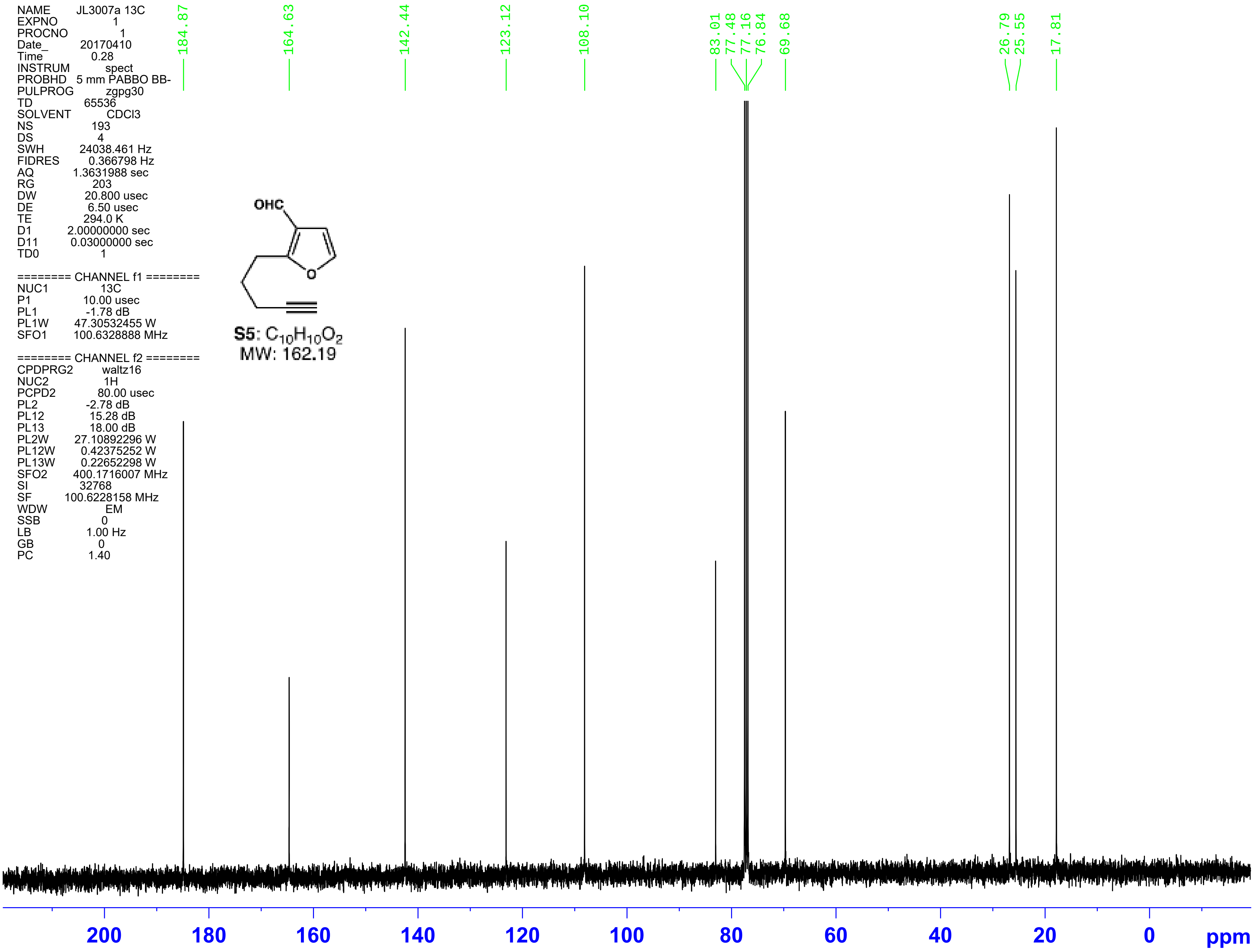
NAME JL3007a 13C
EXPNO 1
PROCNO 1
Date_ 20170410
Time 0.28
INSTRUM spect
PROBHD 5 mm PABBO BB-
PULPROG zgpg30
TD 65536
SOLVENT CDCl3
NS 193
DS 4
SWH 24038.461 Hz
FIDRES 0.366798 Hz
AQ 1.3631988 sec
RG 203
DW 20.800 usec
DE 6.50 usec
TE 294.0 K
D1 2.00000000 sec
D11 0.03000000 sec
TD0 1
======== CHANNEL f1 ========
NUC1 13C
P1 10.00 usec
PL1 -1.78 dB
PL1W 47.30532455 W
SFO1 100.6328888 MHz
======== CHANNEL f2 ========
CPDPRG2 waltz16
NUC2 1H
PCPD2 80.00 usec
PL2 -2.78 dB
PL12 15.28 dB
PL13 18.00 dB
PL2W 27.10892296 W
PL12W 0.42375252 W
PL13W 0.22652298 W
SFO2 400.1716007 MHz
SI 32768
SF 100.6228158 MHz
WDW EM
SSB 0
LB 1.00 Hz
GB 0
PC 1.40
184.87
164.63
142.44
123.12
108.10
83.01
77.48
77.16
76.84
69.68
26.79
25.55
17.81
OHC
O
S5: $C_{10}H_{10}O_2$
MW: 162.19
200 180 160 140 120 100 80 60 40 20 0 ppm

NAME JL3059a 1H
EXPNO 2
PROCNO 1
Date_ 20170602
Time 14.24
INSTRUM spect
PROBHD 5 mm PABBO BB-
PULPROG zg30
TD 65536
SOLVENT CDCl3
NS 8
DS 2
SWH 8223.685 Hz
FIDRES 0.125483 Hz
AQ 3.9846387 sec
RG 181
DW 60.800 usec
DE 6.50 usec
TE 294.0 K
D1 1.00000000 sec
TD0 1

======== CHANNEL f1 ========
NUC1 1H
P1 10.00 usec
PL1 -2.78 dB
PL1W 27.10892296 W
SFO1 400.1724712 MHz
SI 32768
SF 400.1700091 MHz
WDW EM
SSB 0
LB 0.30 Hz
GB 0
PC 1.00

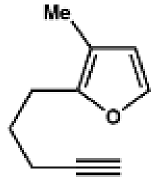

S6: $C_{10}H_{12}O$
MW: 148.21

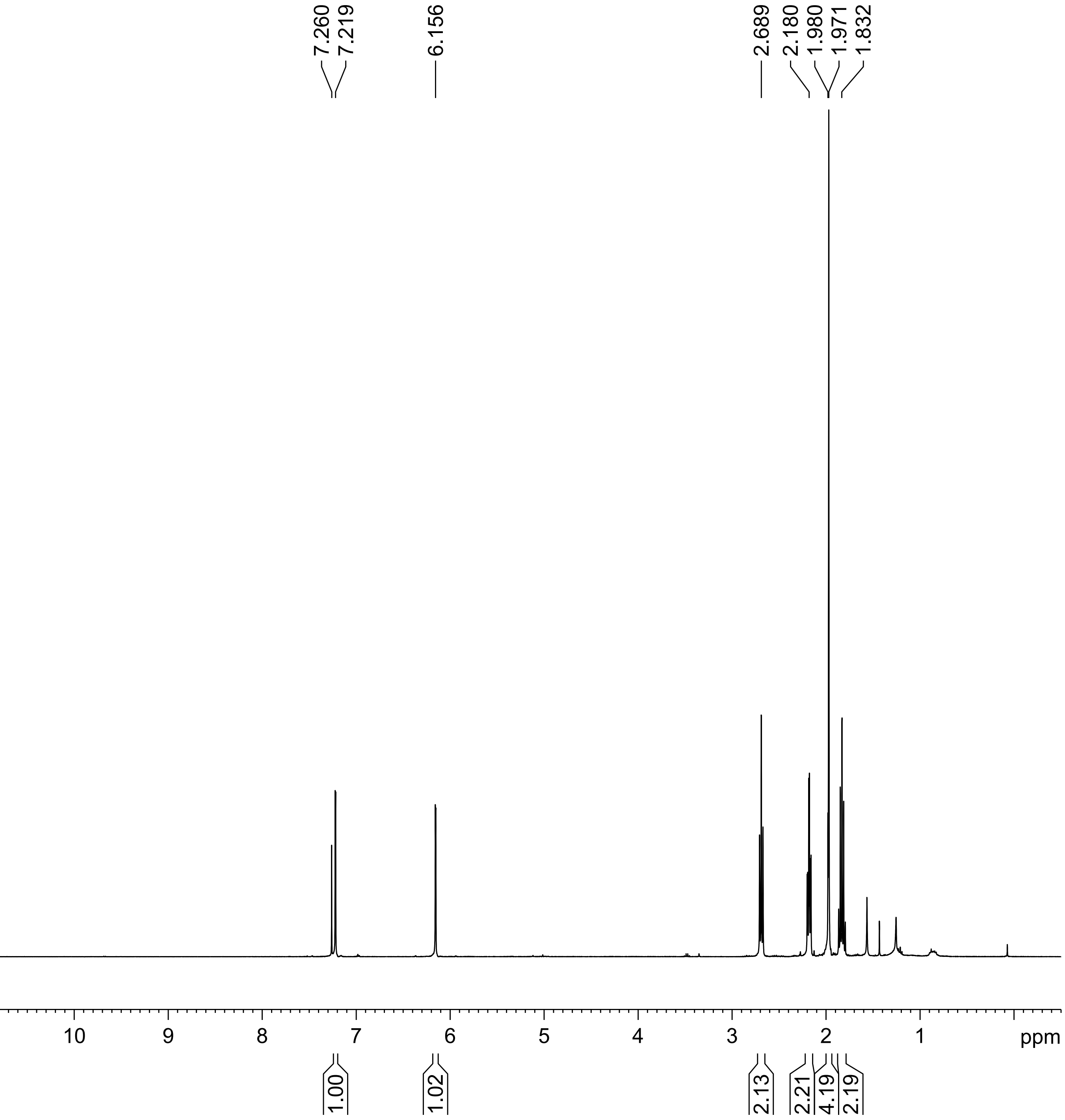

NAME JL3059a 13C
EXPNO 1
PROCNO 1
Date_ 20170514
Time 16.55
INSTRUM spect
PROBHD 5 mm PABBO BB-
PULPROG zgpg30
TD 65536
SOLVENT CDCl3
NS 1024
DS 4
SWH 24038.461 Hz
FIDRES 0.366798 Hz
AQ 1.3631988 sec
RG 203
DW 20.800 usec
DE 6.50 usec
TE 294.4 K
D1 2.00000000 sec
D11 0.03000000 sec
TD0 1

======== CHANNEL f1 ========
NUC1 13C
P1 10.00 usec
PL1 -1.78 dB
PL1W 47.30532455 W
SFO1 100.6328888 MHz

======== CHANNEL f2 ========
CPDPRG2 waltz16
NUC2 1H
PCPD2 80.00 usec
PL2 -2.78 dB
PL12 15.28 dB
PL13 18.00 dB
PL2W 27.10892296 W
PL12W 0.42375252 W
PL13W 0.22652298 W
SFO2 400.1716007 MHz
SI 32768
SF 100.6228270 MHz
WDW EM
SSB 0
LB 1.00 Hz
GB 0
PC 1.40

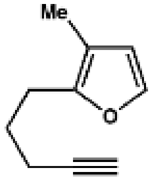

S6: $C_{10}H_{12}O$
MW: 148.21

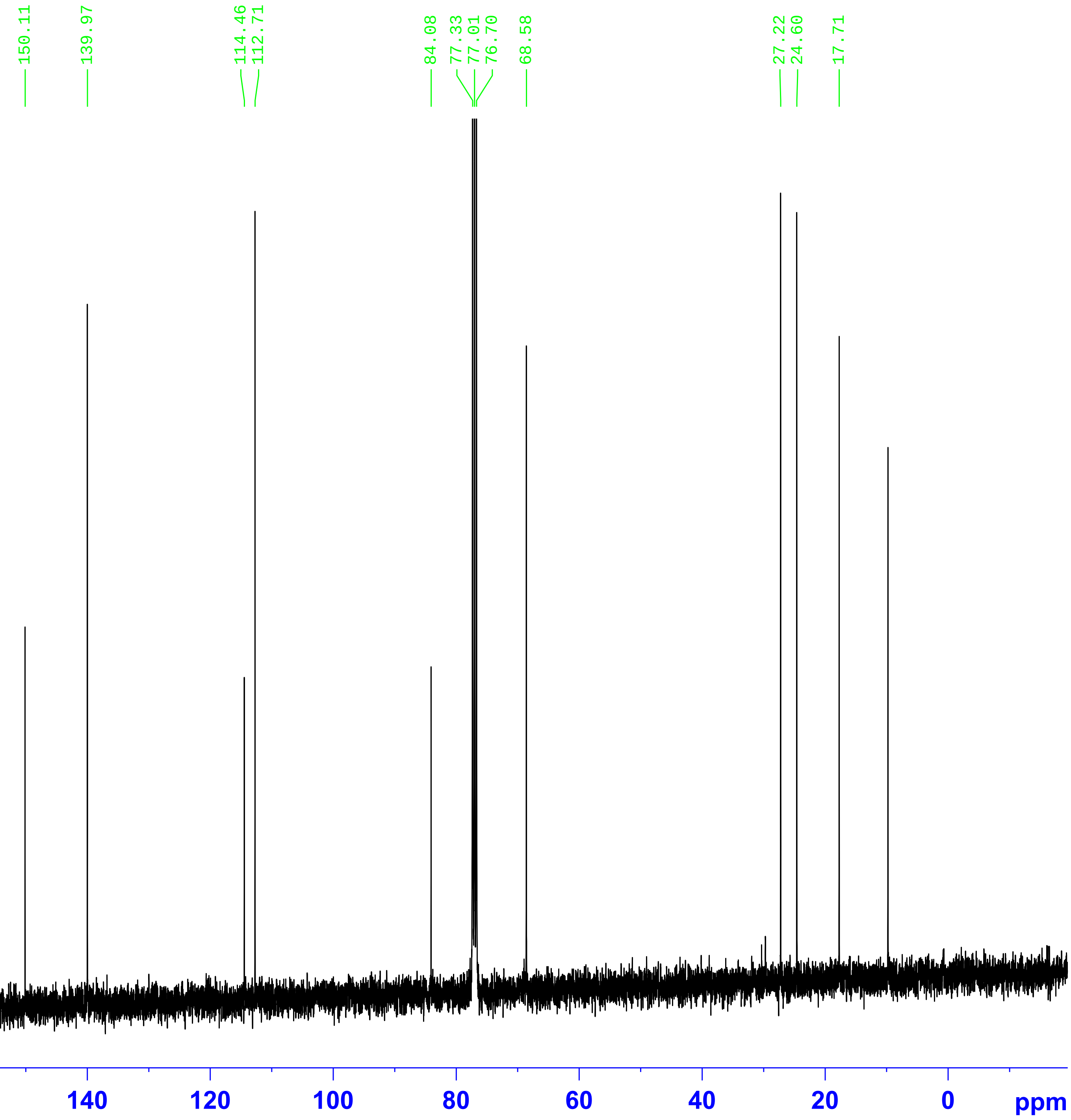

NAME JL3043b 1H
EXPNO 3
PROCNO 1
Date_ 20170503
Time 16.18
INSTRUM spect
PROBHD 5 mm PABBO BB-
PULPROG zg30
TD 65536
SOLVENT CDCl3
NS 8
DS 2
SWH 8223.685 Hz
FIDRES 0.125483 Hz
AQ 3.9846387 sec
RG 203
DW 60.800 usec
DE 6.50 usec
TE 293.7 K
D1 1.00000000 sec
TD0 1

======== CHANNEL f1 ========
NUC1 1H
P1 10.00 usec
PL1 -2.78 dB
PL1W 27.10892296 W
SFO1 400.1724712 MHz
SI 32768
SF 400.1700088 MHz
WDW EM
SSB 0
LB 0.30 Hz
GB 0
PC 1.00

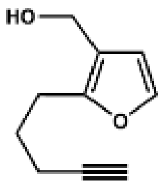

S7: $C_{10}H_{12}O_2$
MW: 164.20

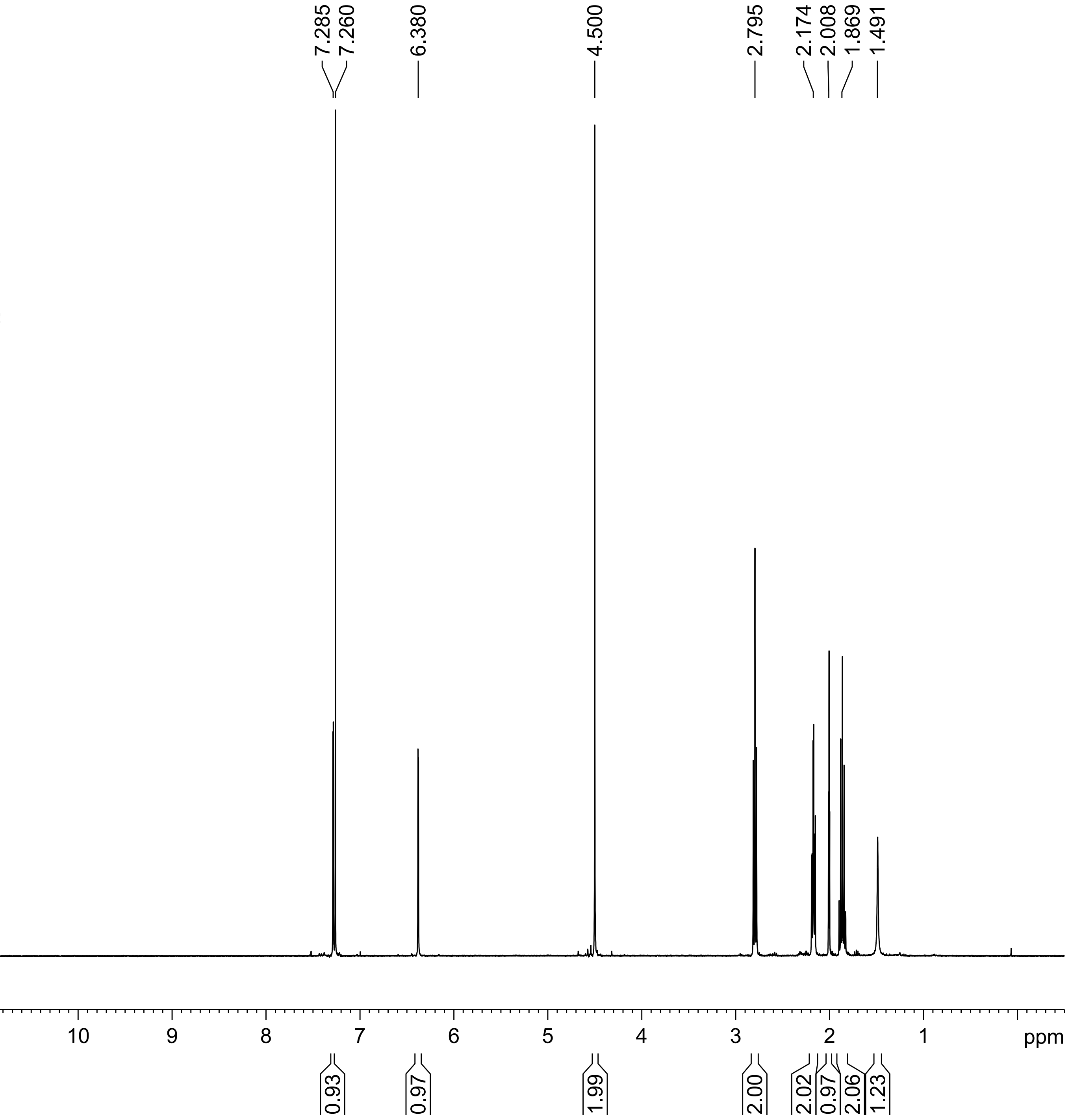

NAME JL3043b 13C
EXPNO 1
PROCNO 1
Date_ 20170503
Time 17.06
INSTRUM spect
PROBHD 5 mm PABBO BB-
PULPROG zgpg30
TD 65536
SOLVENT CDCl3
NS 65
DS 4
SWH 24038.461 Hz
FIDRES 0.366798 Hz
AQ 1.3631988 sec
RG 203
DW 20.800 usec
DE 6.50 usec
TE 294.5 K
D1 2.00000000 sec
D11 0.03000000 sec
TD0 1

======== CHANNEL f1 ========
NUC1 13C
P1 10.00 usec
PL1 -1.78 dB
PL1W 47.30532455 W
SFO1 100.6328888 MHz

======== CHANNEL f2 ========
CPDPRG2 waltz16
NUC2 1H
PCPD2 80.00 usec
PL2 -2.78 dB
PL12 15.28 dB
PL13 18.00 dB
PL2W 27.10892296 W
PL12W 0.42375252 W
PL13W 0.22652298 W
SFO2 400.1716007 MHz
SI 32768
SF 100.6228270 MHz
WDW EM
SSB 0
LB 1.00 Hz
GB 0
PC 1.40

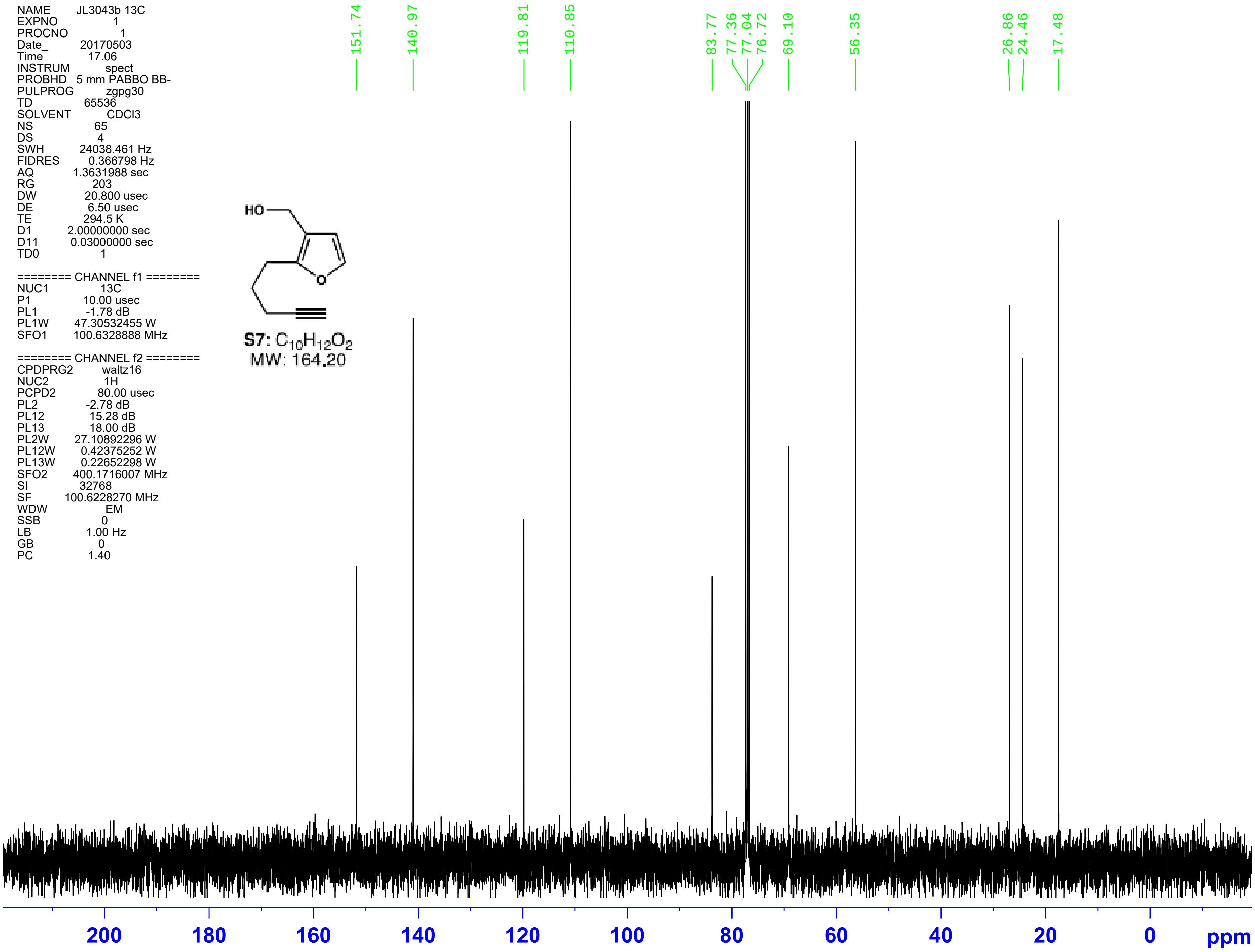

S7: $C_{10}H_{12}O_2$
MW: 164.20

NAME JL3042a 1H
EXPNO 1
PROCNO 1
Date_ 20170503
Time 16.13
INSTRUM spect
PROBHD 5 mm PABBO BB-
PULPROG zg30
TD 65536
SOLVENT CDCl3
NS 8
DS 2
SWH 8223.685 Hz
FIDRES 0.125483 Hz
AQ 3.9846387 sec
RG 203
DW 60.800 usec
DE 6.50 usec
TE 293.7 K
D1 1.00000000 sec
TD0 1

======== CHANNEL f1 ========
NUC1 1H
P1 10.00 usec
PL1 -2.78 dB
PL1W 27.10892296 W
SFO1 400.1724712 MHz
SI 32768
SF 400.1700088 MHz
WDW EM
SSB 0
LB 0.30 Hz
GB 0
PC 1.00

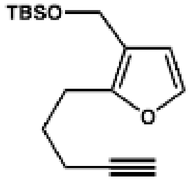

S8: $C_{16}H_{26}O_2Si$
MW: 278.47

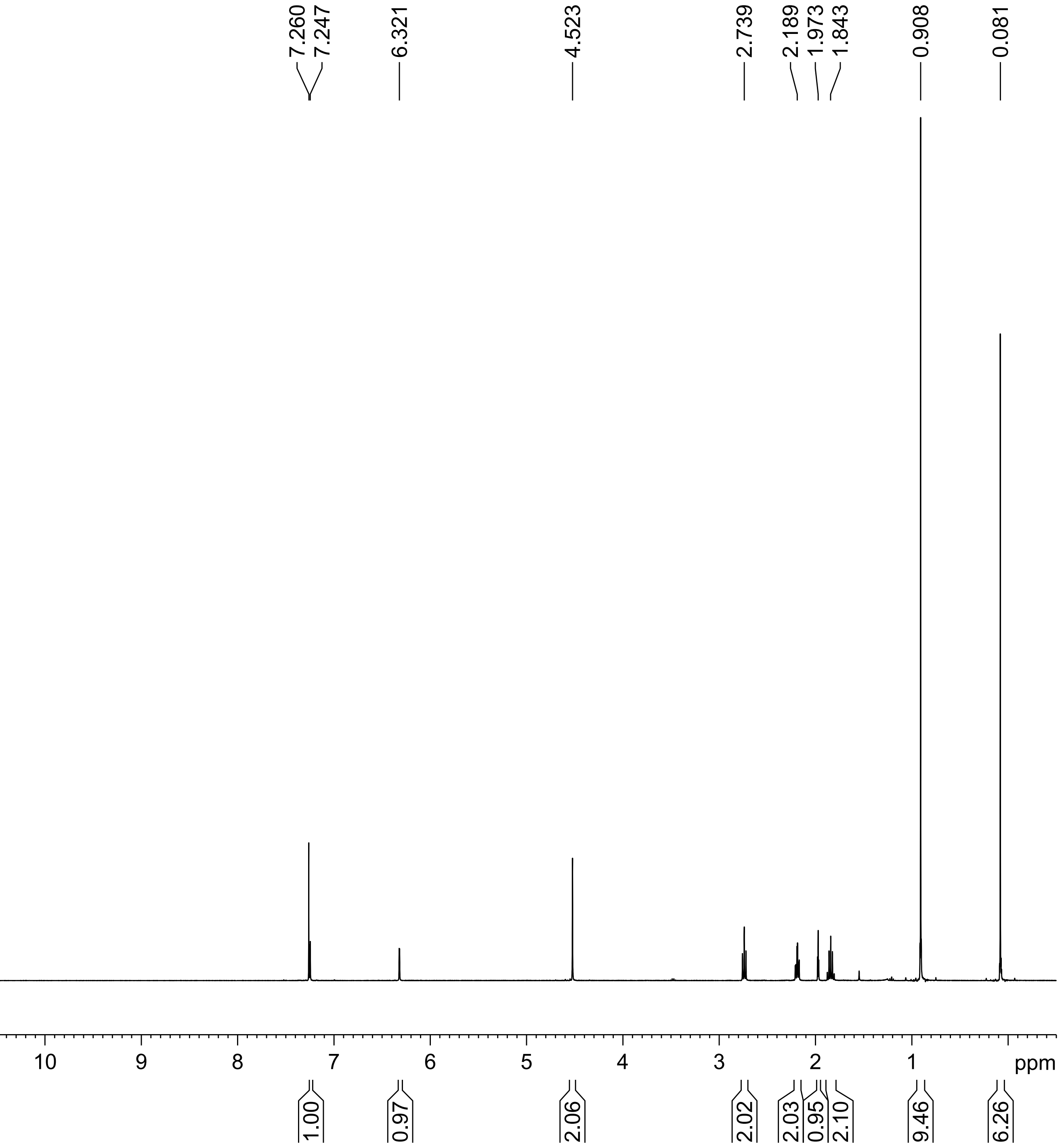

NAME	JL3042a 13C
EXPNO	1
PROCNO	1
Date_	20170503
Time	16.44
INSTRUM	spect
PROBHD	5 mm PABBO BB-
PULPROG	zgpg30
TD	65536
SOLVENT	CDCl3
NS	129
DS	4
SWH	24038.461 Hz
FIDRES	0.366798 Hz
AQ	1.3631988 sec
RG	203
DW	20.800 usec
DE	6.50 usec
TE	294.3 K
D1	2.00000000 sec
D11	0.03000000 sec
TD0	1

======== CHANNEL f1 ========

NUC1	13C
P1	10.00 usec
PL1	-1.78 dB
PL1W	47.30532455 W
SFO1	100.6328888 MHz

======== CHANNEL f2 ========

CPDPRG2	waltz16
NUC2	1H
PCPD2	80.00 usec
PL2	-2.78 dB
PL12	15.28 dB
PL13	18.00 dB
PL2W	27.10892296 W
PL12W	0.42375252 W
PL13W	0.22652298 W
SFO2	400.1716007 MHz
SI	32768
SF	100.6228270 MHz
WDW	EM
SSB	0
LB	1.00 Hz
GB	0
PC	1.40

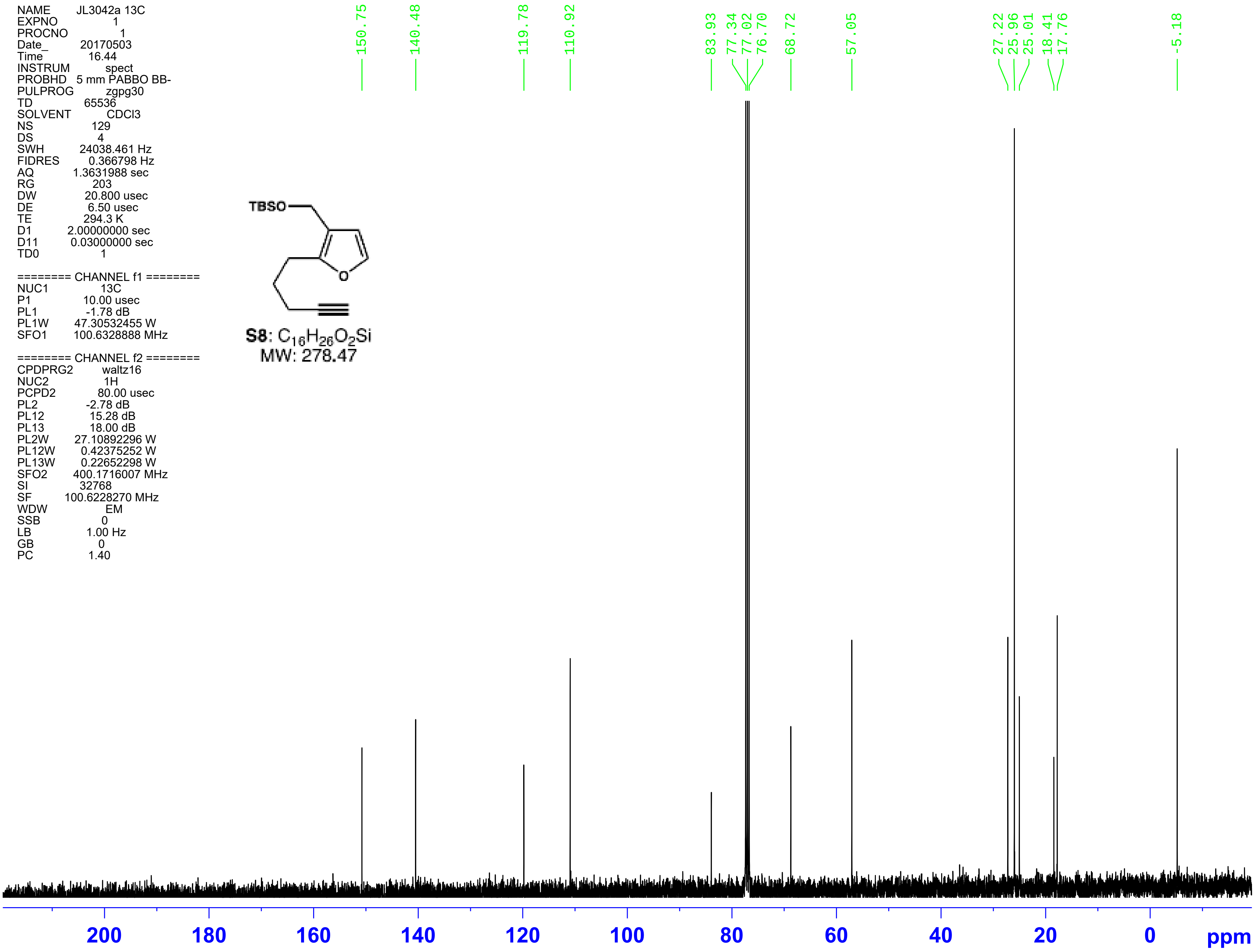

S8: $C_{16}H_{26}O_2Si$
MW: 278.47

NAME AES 2167b
EXPNO 1
PROCNO 1
Date_ 20160429
Time 23.55
INSTRUM spect
PROBHD 5 mm PABBO BB-
PULPROG zg30
TD 65536
SOLVENT CDCl3
NS 8
DS 2
SWH 8223.685 Hz
FIDRES 0.125483 Hz
AQ 3.9846387 sec
RG 203
DW 60.800 usec
DE 6.50 usec
TE 293.9 K
D1 1.00000000 sec
TD0 1

======== CHANNEL f1 ========
NUC1 1H
P1 10.00 usec
PL1 -2.78 dB
PL1W 27.10892296 W
SFO1 400.1724712 MHz
SI 32768
SF 400.1700088 MHz
WDW EM
SSB 0
LB 0.30 Hz
GB 0
PC 1.00

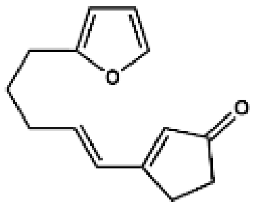

4: $C_{14}H_{16}O_2$
MW: 216.28

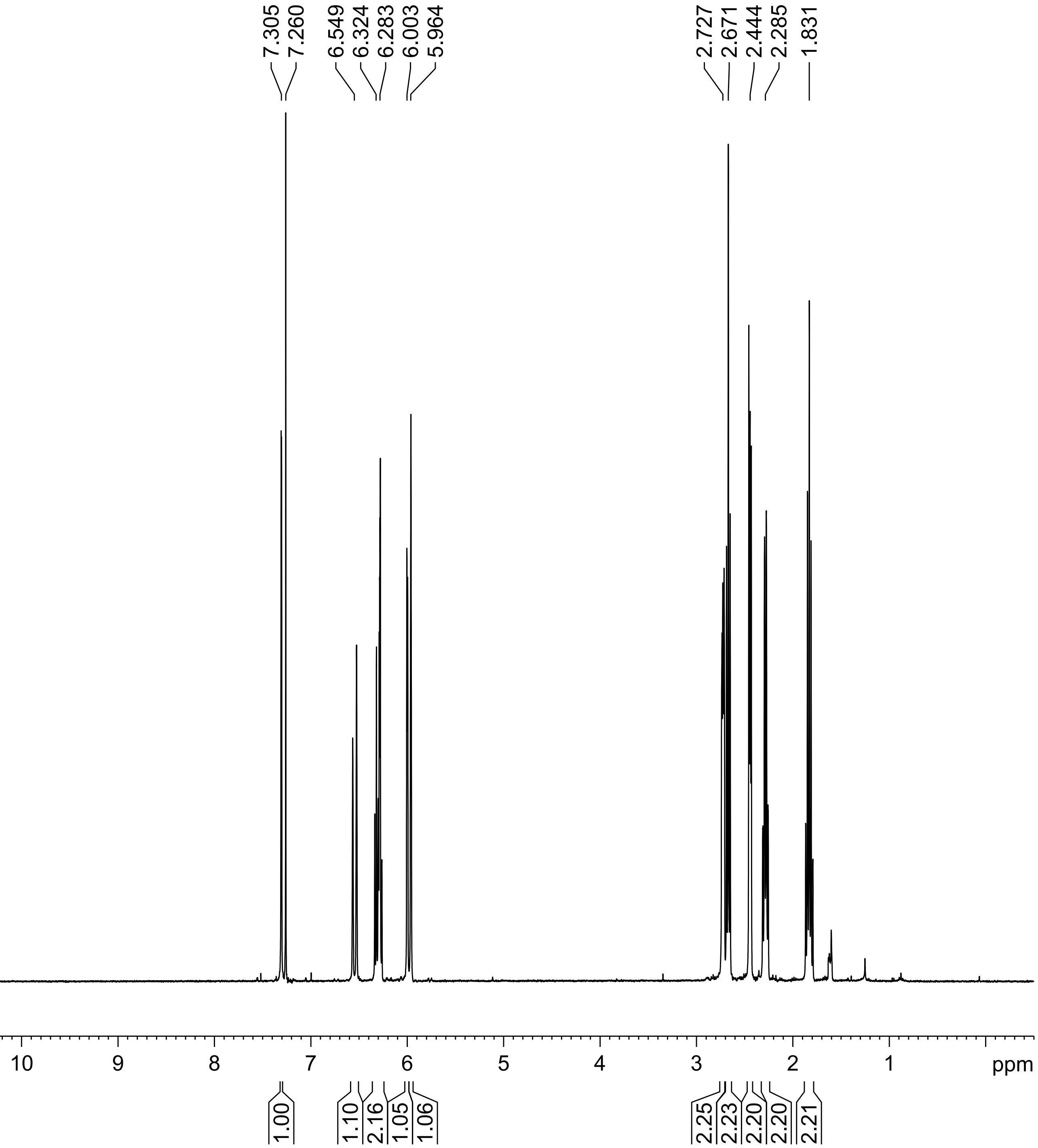

NAME AES 2027 C13
EXPNO 1
PROCNO 1
Date_ 20151210
Time 20.34
INSTRUM spect
PROBHD 5 mm PABBO BB-
PULPROG zgpg30
TD 65536
SOLVENT CDCl3
NS 256
DS 4
SWH 24038.461 Hz
FIDRES 0.366798 Hz
AQ 1.3631988 sec
RG 144
DW 20.800 usec
DE 6.50 usec
TE 294.6 K
D1 2.00000000 sec
D11 0.03000000 sec
TD0 1

======== CHANNEL f1 ========
NUC1 13C
P1 10.00 usec
PL1 -1.78 dB
PL1W 47.30532455 W
SFO1 100.6328888 MHz

======== CHANNEL f2 ========
CPDPRG2 waltz16
NUC2 1H
PCPD2 80.00 usec
PL2 -2.78 dB
PL12 15.28 dB
PL13 18.00 dB
PL2W 27.10892296 W
PL12W 0.42375252 W
PL13W 0.22652298 W
SFO2 400.1716007 MHz
SI 32768
SF 100.6228270 MHz
WDW EM
SSB 0
LB 1.00 Hz
GB 0
PC 1.40

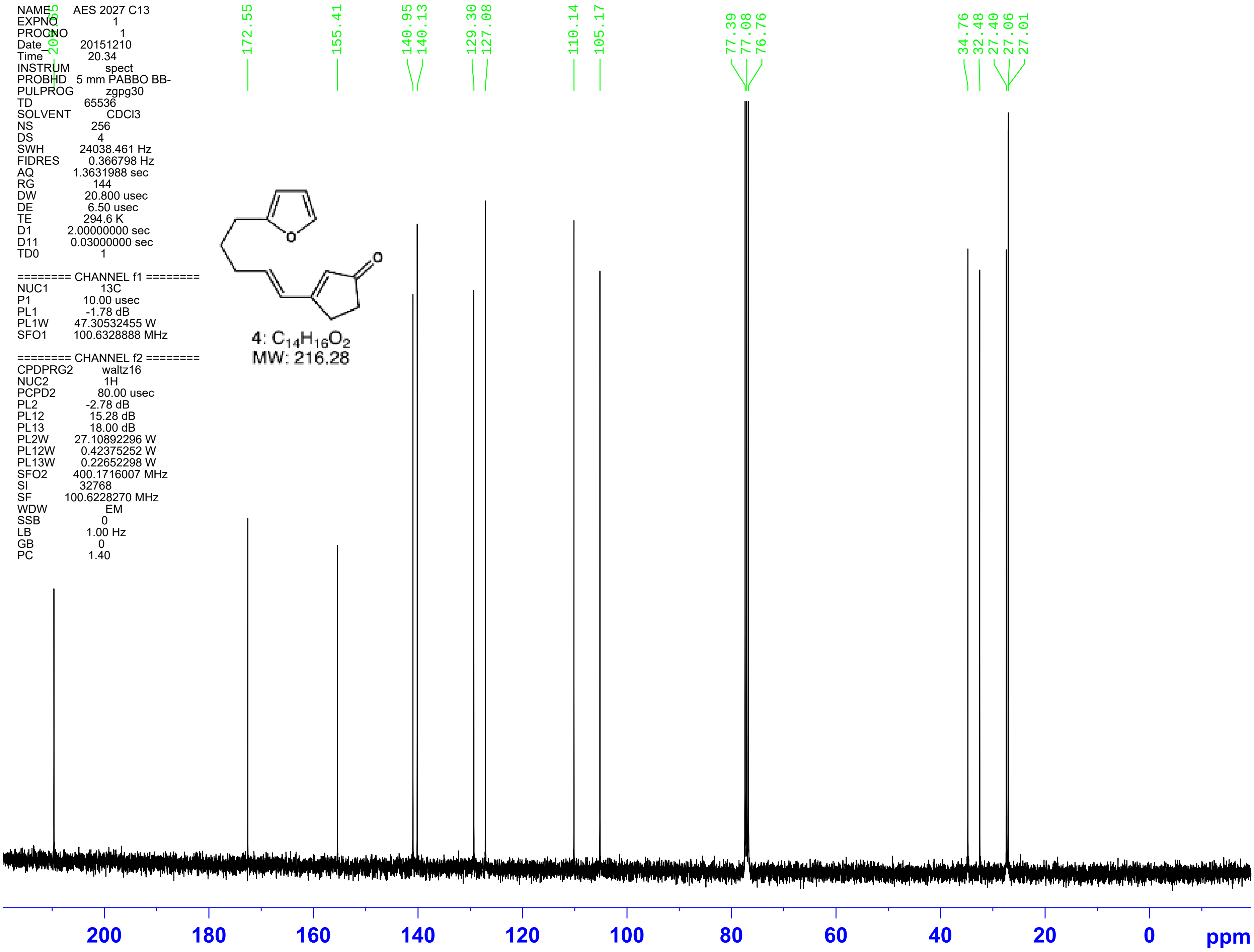

4: $C_{14}H_{16}O_2$
MW: 216.28

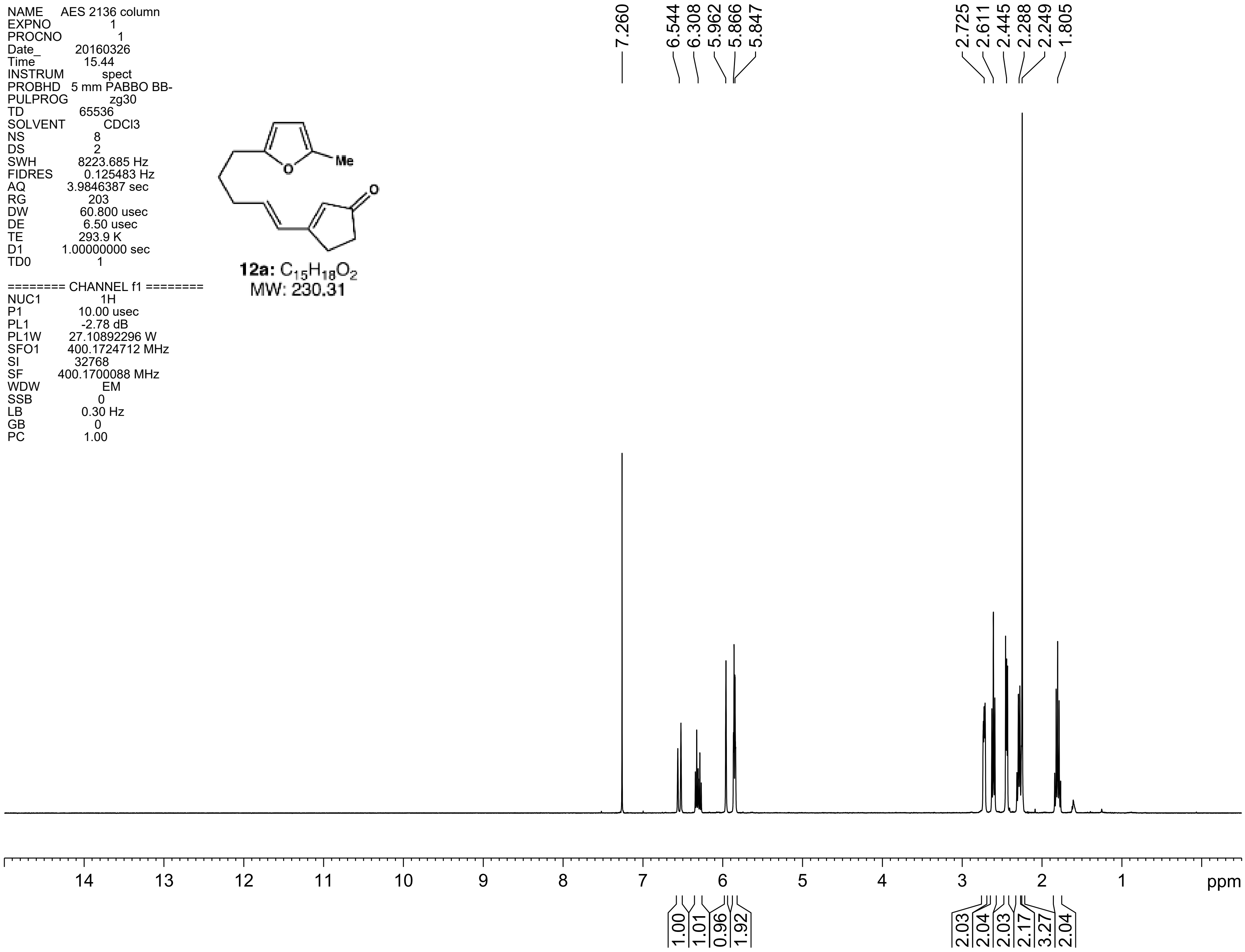

12a: $C_{15}H_{18}O_2$
MW: 230.31

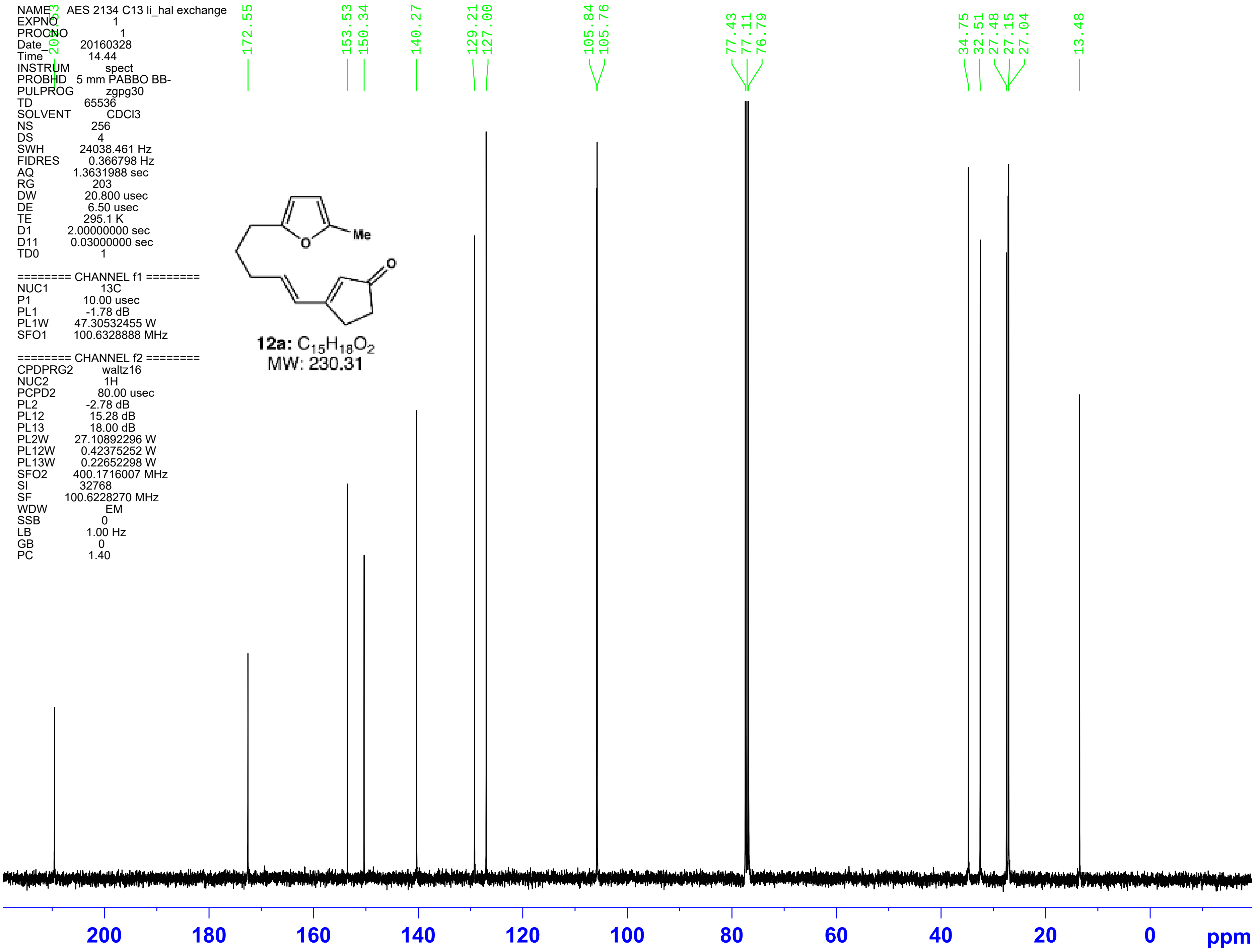

NAME AES 2134 C13 li_hal exchange
EXPNO 1
PROCNO 1
Date_ 20160328
Time 14.44
INSTRUM spect
PROBHD 5 mm PABBO BB-
PULPROG zgpg30
TD 65536
SOLVENT CDCl3
NS 256
DS 4
SWH 24038.461 Hz
FIDRES 0.366798 Hz
AQ 1.3631988 sec
RG 203
DW 20.800 usec
DE 6.50 usec
TE 295.1 K
D1 2.00000000 sec
D11 0.03000000 sec
TD0 1

======== CHANNEL f1 ========
NUC1 13C
P1 10.00 usec
PL1 -1.78 dB
PL1W 47.30532455 W
SFO1 100.6328888 MHz

======== CHANNEL f2 ========
CPDPRG2 waltz16
NUC2 1H
PCPD2 80.00 usec
PL2 -2.78 dB
PL12 15.28 dB
PL13 18.00 dB
PL2W 27.10892296 W
PL12W 0.42375252 W
PL13W 0.22652298 W
SFO2 400.1716007 MHz
SI 32768
SF 100.6228270 MHz
WDW EM
SSB 0
LB 1.00 Hz
GB 0
PC 1.40

12a: $C_{15}H_{18}O_2$
MW: 230.31

NAME JAL2109D 1H
EXPNO 3
PROCNO 1
Date_ 20170808
Time 15.52
INSTRUM spect
PROBHD 5 mm PABBO BB-
PULPROG zg30
TD 65536
SOLVENT CDCl3
NS 8
DS 2
SWH 8223.685 Hz
FIDRES 0.125483 Hz
AQ 3.9846387 sec
RG 203
DW 60.800 usec
DE 6.50 usec
TE 295.9 K
D1 1.00000000 sec
TD0 1

======== CHANNEL f1 ========
NUC1 1H
P1 10.00 usec
PL1 -2.78 dB
PL1W 27.10892296 W
SFO1 400.1724712 MHz
SI 32768
SF 400.1700083 MHz
WDW EM
SSB 0
LB 0.30 Hz
GB 0
PC 1.00

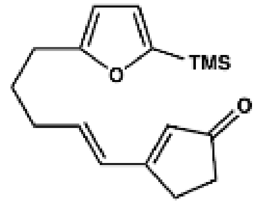

12b: $C_{17}H_{24}O_2Si$
MW: 288.46

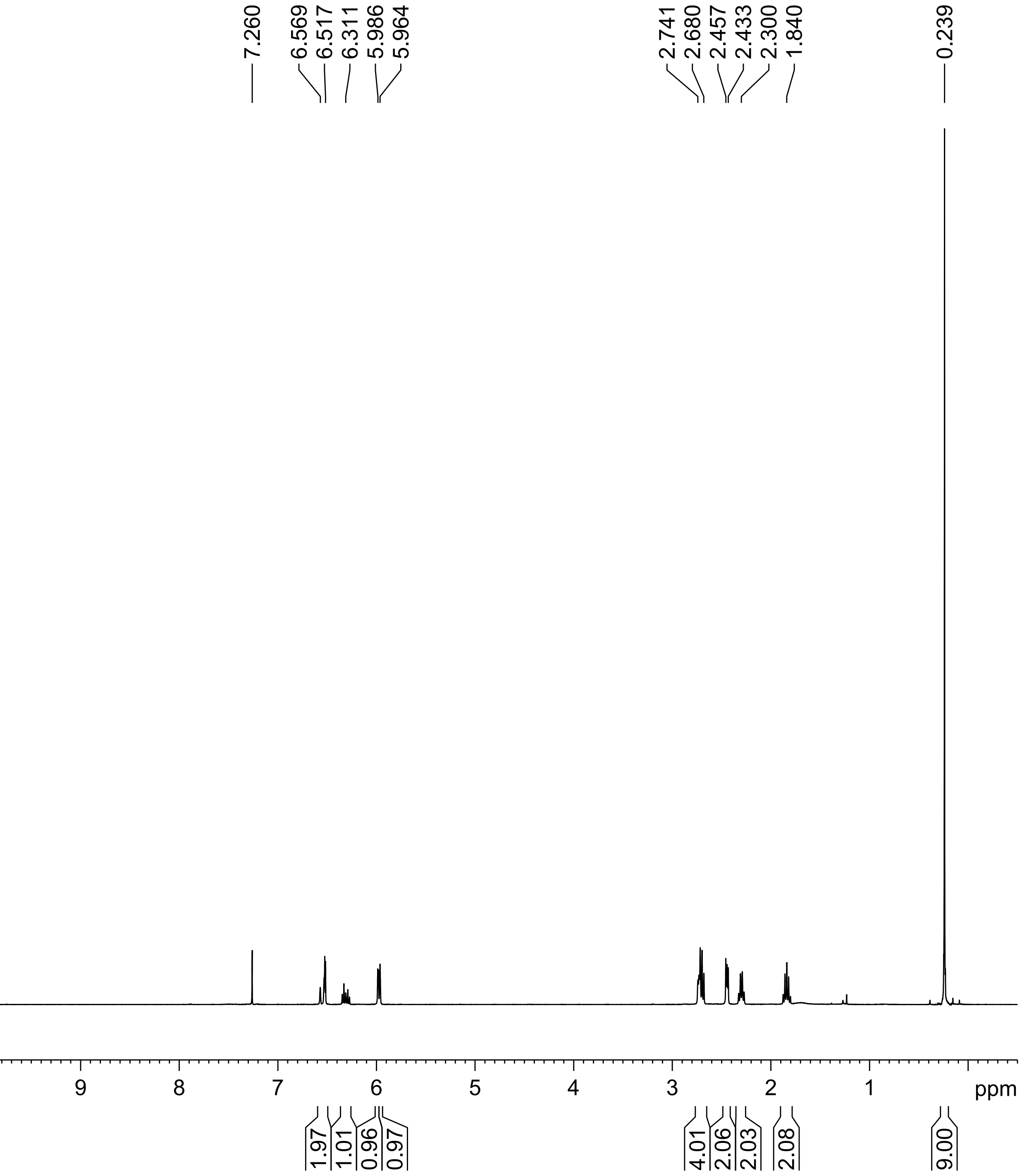

NAME JAL2018D 13C
EXPNO 2
PROCNO 1
Date_ 20170315
Time 14.40
INSTRUM spect
PROBHD 5 mm PABBO BB-
PULPROG zgpg30
TD 65536
SOLVENT CDCl3
NS 41
DS 4
SWH 24038.461 Hz
FIDRES 0.366798 Hz
AQ 1.3631988 sec
RG 203
DW 20.800 usec
DE 6.50 usec
TE 293.2 K
D1 2.00000000 sec
D11 0.03000000 sec
TD0 1

======== CHANNEL f1 ========
NUC1 13C
P1 10.00 usec
PL1 -1.78 dB
PL1W 47.30532455 W
SFO1 100.6328888 MHz

======== CHANNEL f2 ========
CPDPRG2 waltz16
NUC2 1H
PCPD2 80.00 usec
PL2 -2.78 dB
PL12 15.28 dB
PL13 18.00 dB
PL2W 27.10892296 W
PL12W 0.42375252 W
PL13W 0.22652298 W
SFO2 400.1716007 MHz
SI 32768
SF 100.6228270 MHz
WDW EM
SSB 0
LB 1.00 Hz
GB 0
PC 1.40

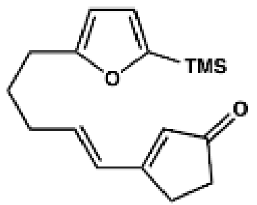

12b: $C_{17}H_{24}O_2Si$
MW: 288.46

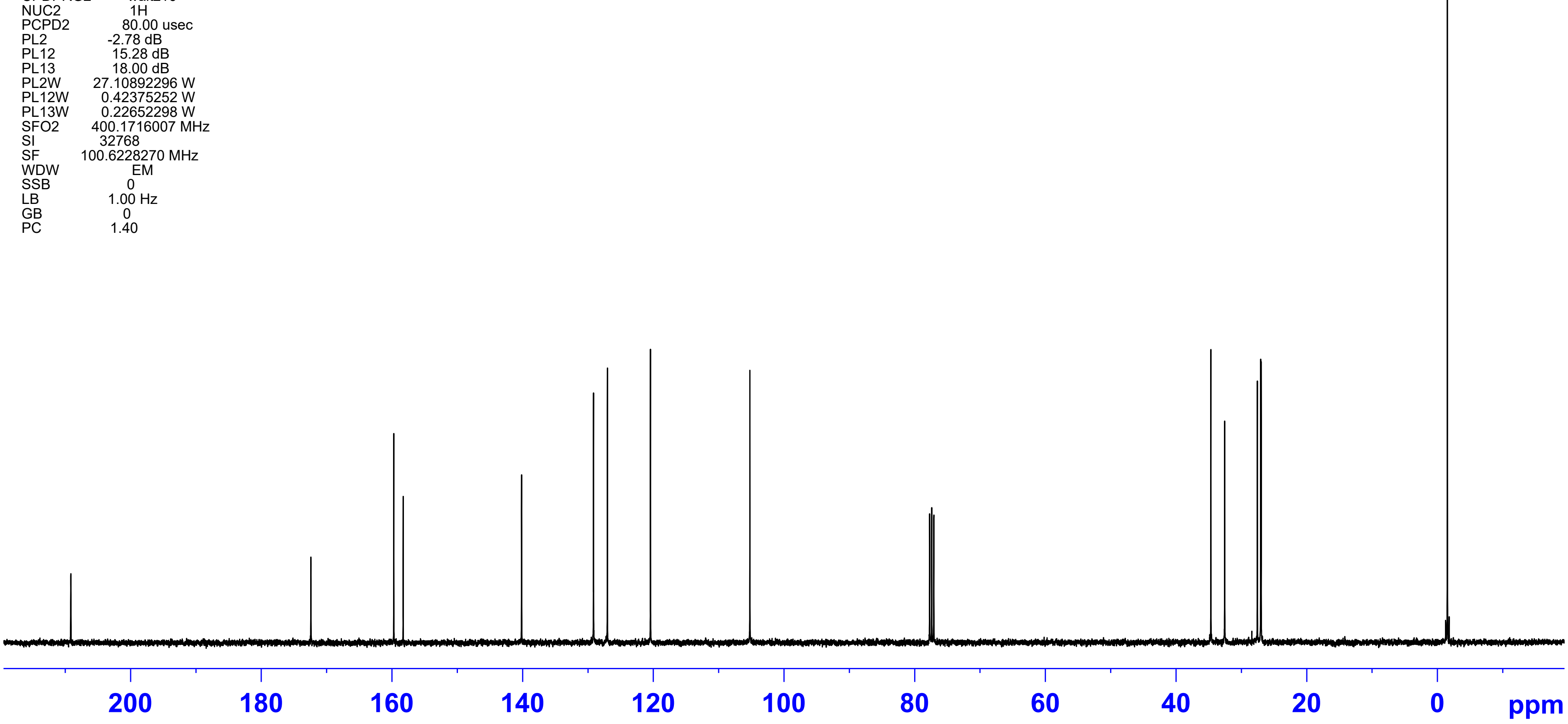

NAME AES 4028 column2
EXPNO 2
PROCNO 1
Date_ 20170426
Time 21.14
INSTRUM spect
PROBHD 5 mm PABBO BB-
PULPROG zg30
TD 65536
SOLVENT CDCl3
NS 16
DS 2
SWH 8223.685 Hz
FIDRES 0.125483 Hz
AQ 3.9846387 sec
RG 203
DW 60.800 usec
DE 6.50 usec
TE 294.9 K
D1 1.00000000 sec
TD0 1

======== CHANNEL f1 ========
NUC1 1H
P1 10.00 usec
PL1 -2.78 dB
PL1W 27.10892296 W
SFO1 400.1724712 MHz
SI 32768
SF 400.1700088 MHz
WDW EM
SSB 0
LB 0.30 Hz
GB 0
PC 1.00

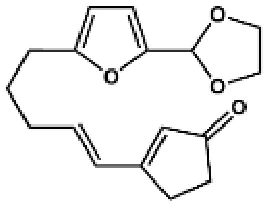

12c: $C_{17}H_{20}O_4$
MW: 288.34

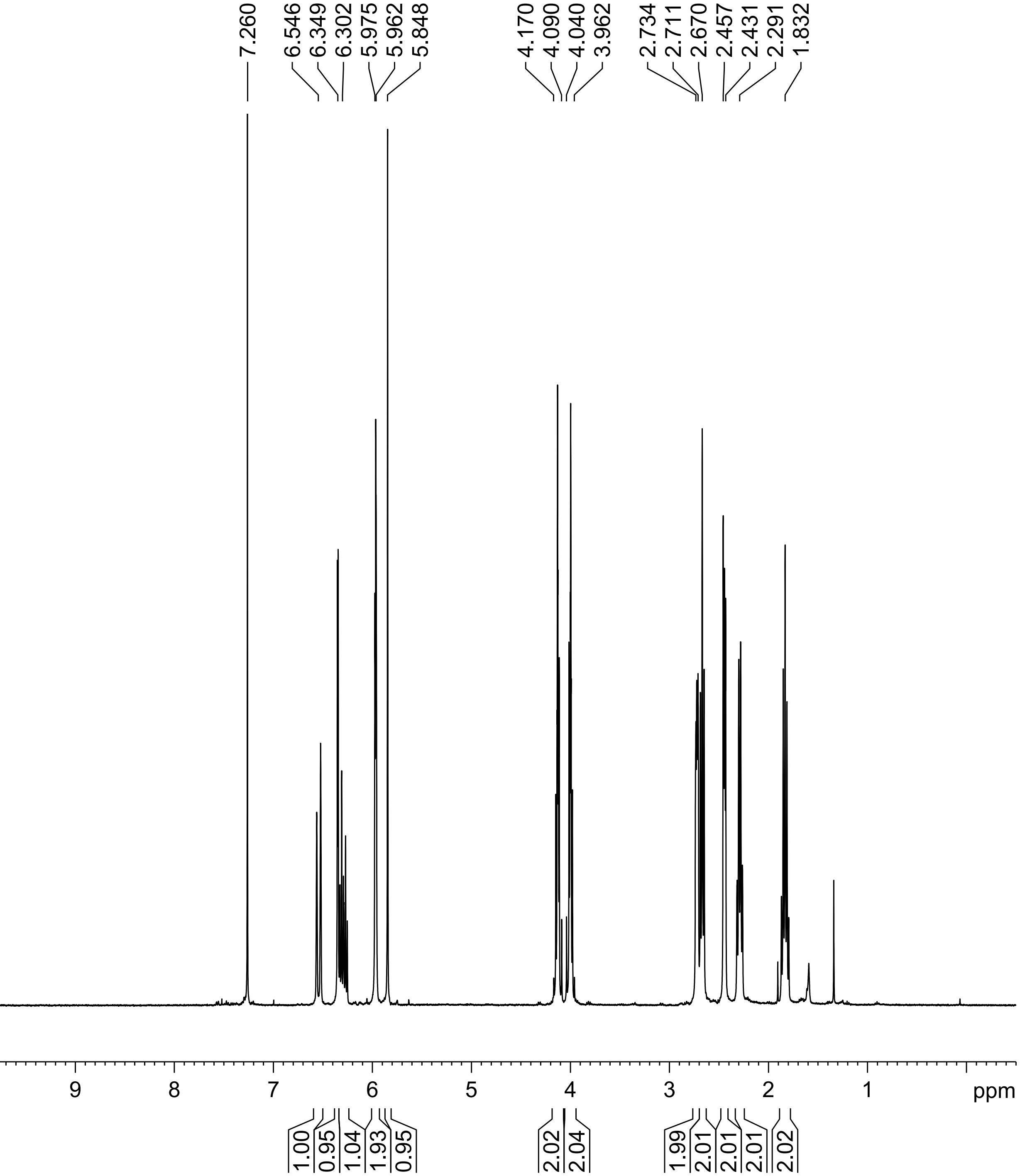

NAME JAL2015Db repurified 13C
EXPNO 2
PROCNO 1
Date_ 20170315
Time 14.25
INSTRUM spect
PROBHD 5 mm PABBO BB-
PULPROG zgpg30
TD 65536
SOLVENT CDCl3
NS 50
DS 4
SWH 24038.461 Hz
FIDRES 0.366798 Hz
AQ 1.3631988 sec
RG 203
DW 20.800 usec
DE 6.50 usec
TE 293.2 K
D1 2.00000000 sec
D11 0.03000000 sec
TD0 1

======== CHANNEL f1 ========
NUC1 13C
P1 10.00 usec
PL1 -1.78 dB
PL1W 47.30532455 W
SFO1 100.6328888 MHz

======== CHANNEL f2 ========
CPDPRG2 waltz16
NUC2 1H
PCPD2 80.00 usec
PL2 -2.78 dB
PL12 15.28 dB
PL13 18.00 dB
PL2W 27.10892296 W
PL12W 0.42375252 W
PL13W 0.22652298 W
SFO2 400.1716007 MHz
SI 32768
SF 100.6228270 MHz
WDW EM
SSB 0
LB 1.00 Hz
GB 0
PC 1.40

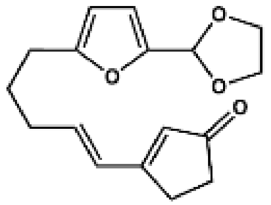

12c: $C_{17}H_{20}O_4$
MW: 288.34

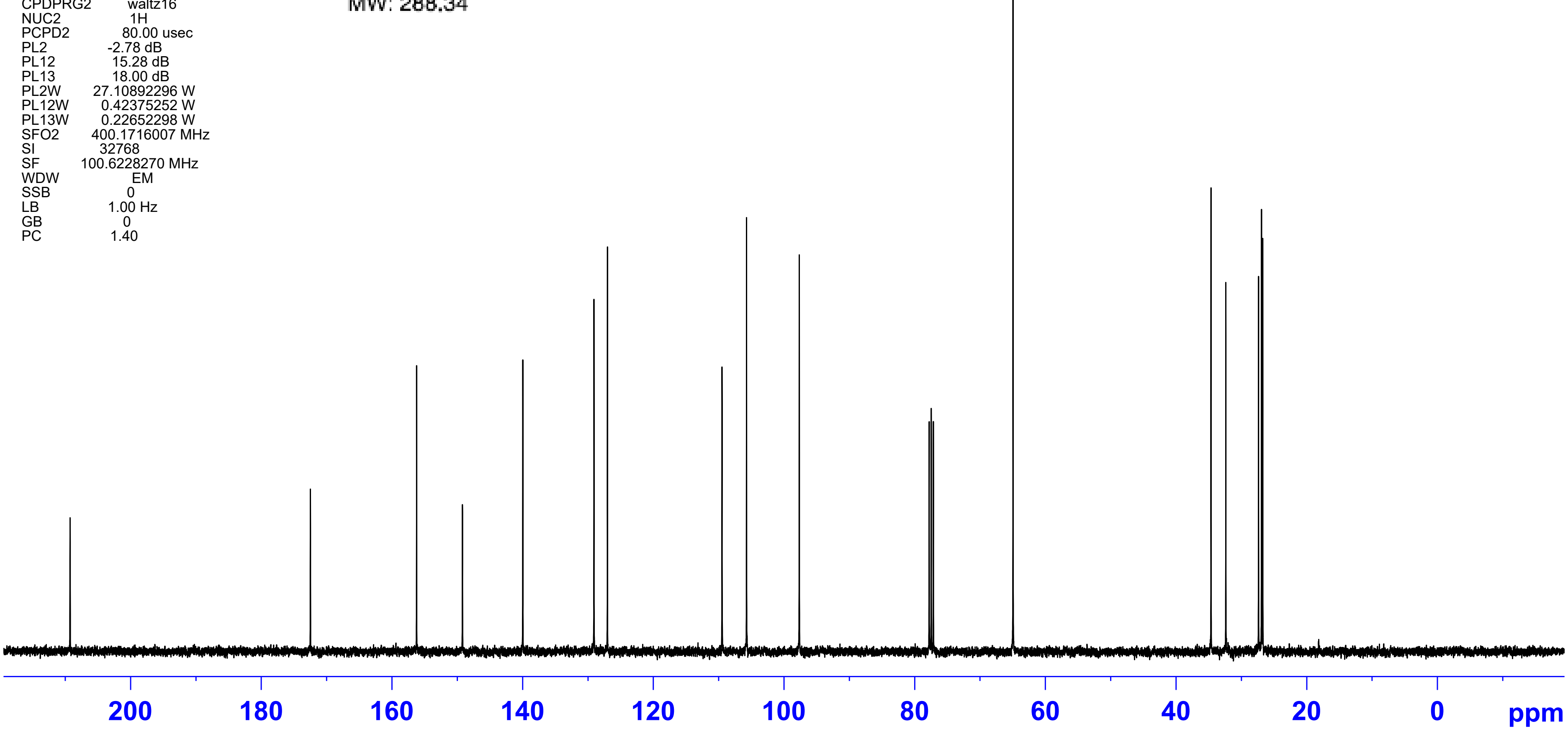

NAME JAL1067B
EXPNO 1
PROCNO 1
Date_ 20160727
Time 19.09
INSTRUM spect
PROBHD 5 mm PABBO BB-
PULPROG zg30
TD 65536
SOLVENT CDCl3
NS 8
DS 2
SWH 8223.685 Hz
FIDRES 0.125483 Hz
AQ 3.9846387 sec
RG 50.8
DW 60.800 usec
DE 6.50 usec
TE 293.9 K
D1 1.00000000 sec
TD0 1

======== CHANNEL f1 ========
NUC1 1H
P1 10.00 usec
PL1 -2.78 dB
PL1W 27.10892296 W
SFO1 400.1724712 MHz
SI 32768
SF 400.1700091 MHz
WDW EM
SSB 0
LB 0.30 Hz
GB 0
PC 1.00

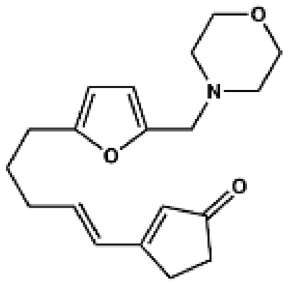

12d: $C_{19}H_{25}NO_3$
MW: 315.41

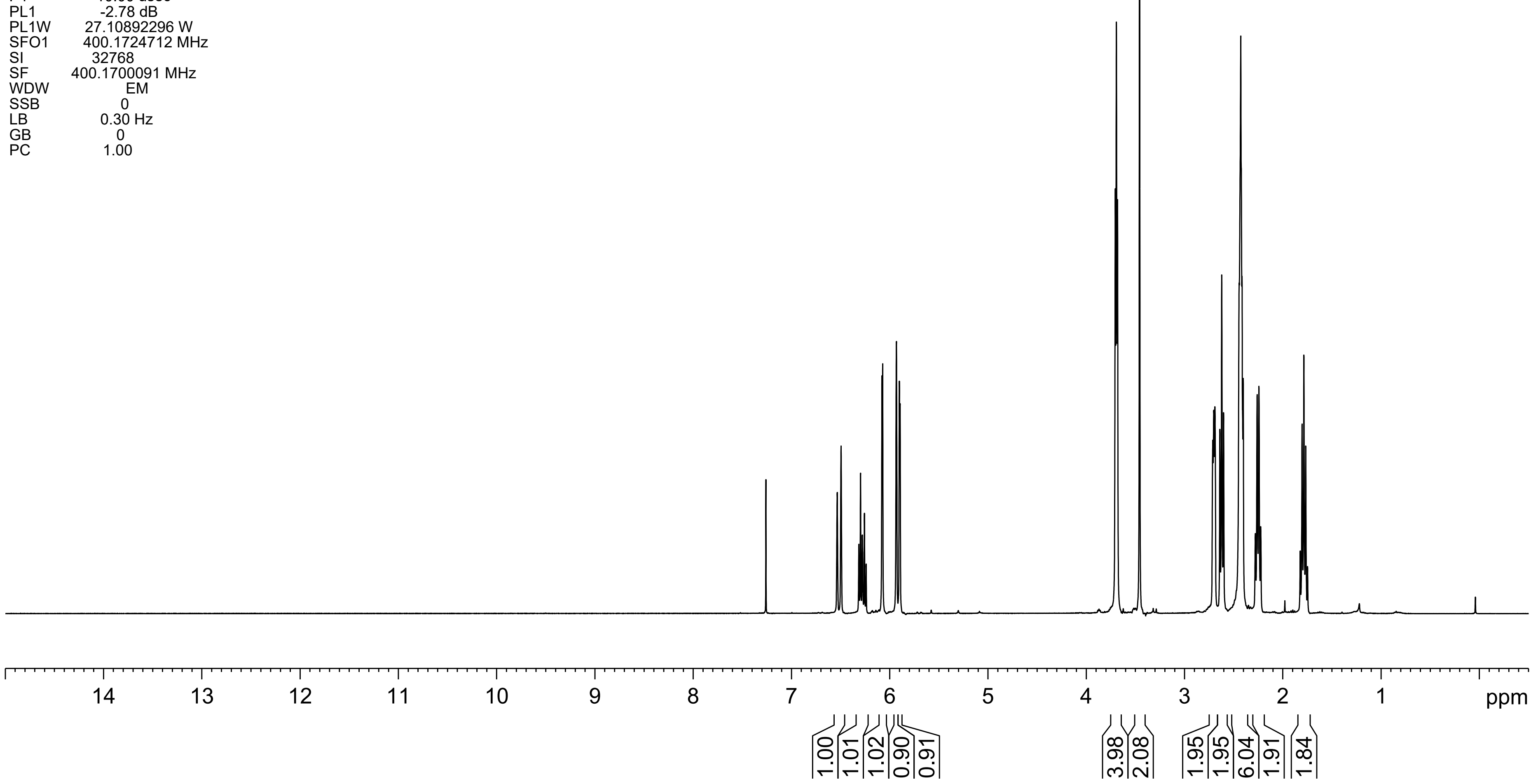

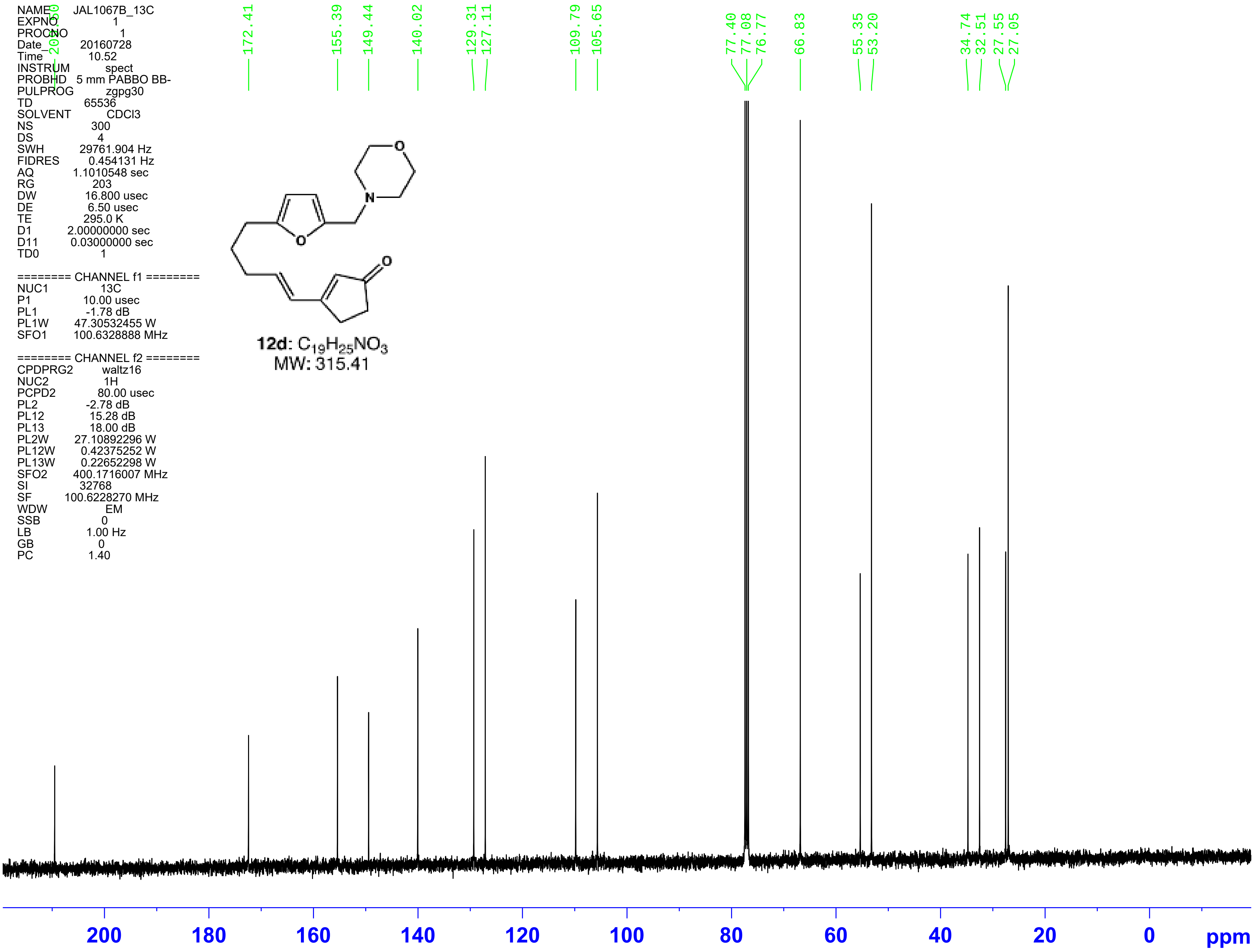

NAME JAL1067B_13C
EXPNO 1
PROCNO 1
Date_ 20160728
Time 10.52
INSTRUM spect
PROBHD 5 mm PABBO BB-
PULPROG zgpg30
TD 65536
SOLVENT CDCl3
NS 300
DS 4
SWH 29761.904 Hz
FIDRES 0.454131 Hz
AQ 1.1010548 sec
RG 203
DW 16.800 usec
DE 6.50 usec
TE 295.0 K
D1 2.00000000 sec
D11 0.03000000 sec
TD0 1
======== CHANNEL f1 ========
NUC1 13C
P1 10.00 usec
PL1 -1.78 dB
PL1W 47.30532455 W
SFO1 100.6328888 MHz
======== CHANNEL f2 ========
CPDPRG2 waltz16
NUC2 1H
PCPD2 80.00 usec
PL2 -2.78 dB
PL12 15.28 dB
PL13 18.00 dB
PL2W 27.10892296 W
PL12W 0.42375252 W
PL13W 0.22652298 W
SFO2 400.1716007 MHz
SI 32768
SF 100.6228270 MHz
WDW EM
SSB 0
LB 1.00 Hz
GB 0
PC 1.40
209.50
172.41
155.39
149.44
140.02
129.31
127.11
109.79
105.65
77.40
77.08
76.77
66.83
55.35
53.20
34.74
32.51
27.55
27.05
O
N
O
O
12d: C19H25NO3
MW: 315.41
200
180
160
140
120
100
80
60
40
20
0
ppm

NAME JL2232b 1H
EXPNO 2
PROCNO 1
Date_ 20170412
Time 14.05
INSTRUM spect
PROBHD 5 mm PABBO BB-
PULPROG zg30
TD 65536
SOLVENT CDCl3
NS 8
DS 2
SWH 8223.685 Hz
FIDRES 0.125483 Hz
AQ 3.9846387 sec
RG 203
DW 60.800 usec
DE 6.50 usec
TE 293.2 K
D1 1.00000000 sec
TD0 1

======== CHANNEL f1 ========
NUC1 1H
P1 10.00 usec
PL1 -2.78 dB
PL1W 27.10892296 W
SFO1 400.1724712 MHz
SI 32768
SF 400.1700088 MHz
WDW EM
SSB 0
LB 0.30 Hz
GB 0
PC 1.00

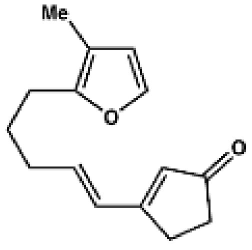

12e: $C_{15}H_{18}O_2$
MW: 230.31

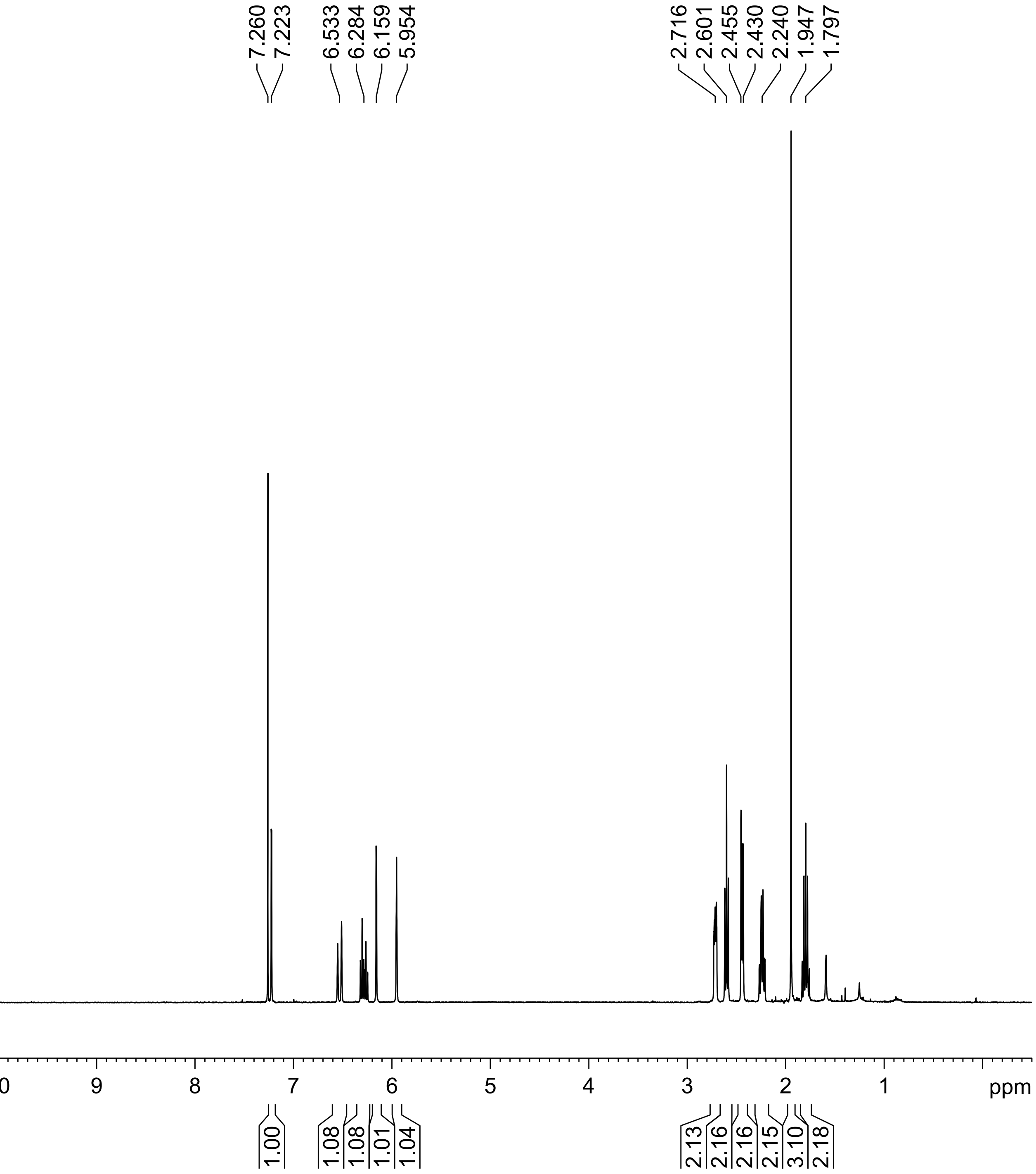

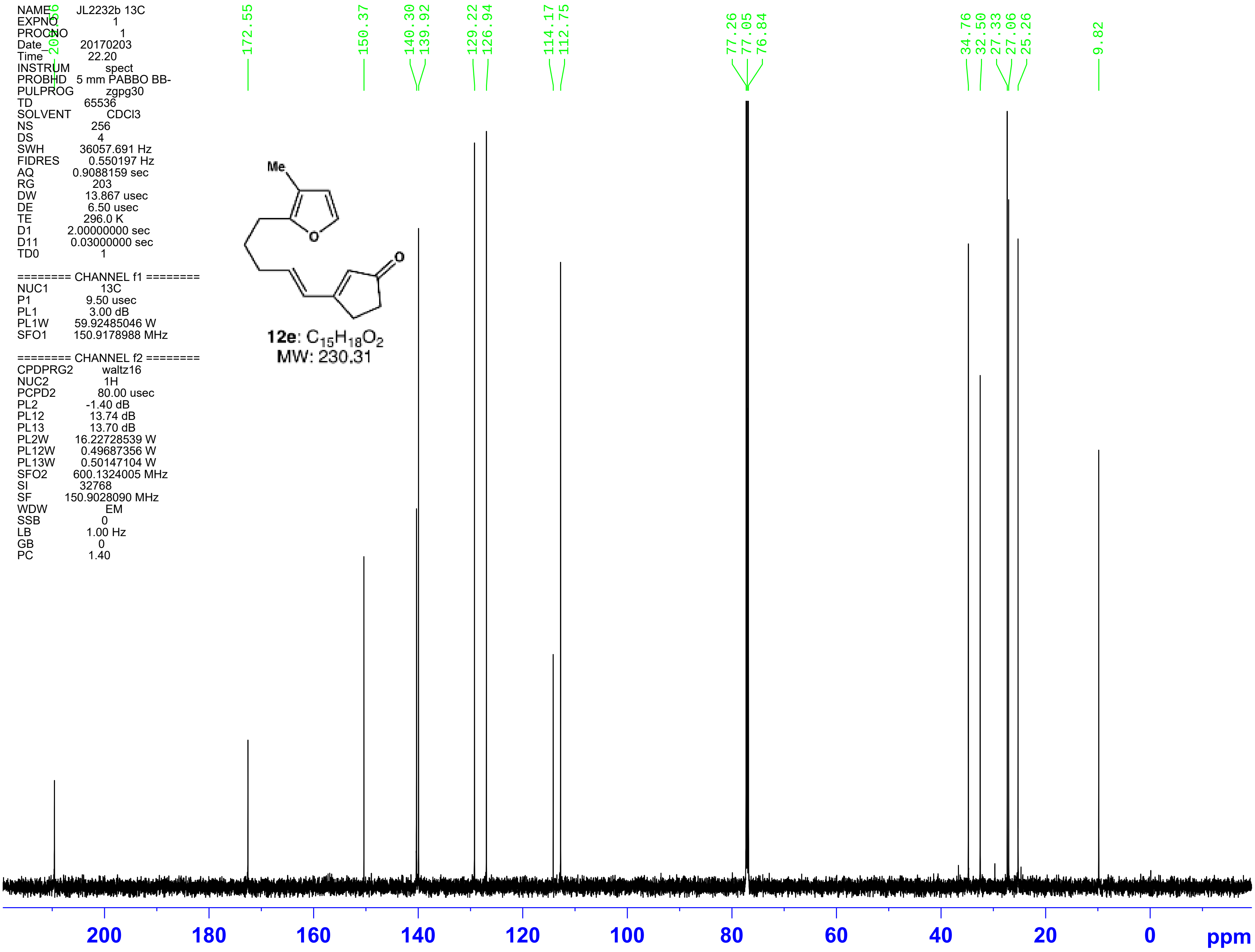

NAME JL2232b 13C
EXPNO 1
PROCNO 1
Date_ 20170203
Time 22.20
INSTRUM spect
PROBHD 5 mm PABBO BB-
PULPROG zgpg30
TD 65536
SOLVENT CDCl3
NS 256
DS 4
SWH 36057.691 Hz
FIDRES 0.550197 Hz
AQ 0.9088159 sec
RG 203
DW 13.867 usec
DE 6.50 usec
TE 296.0 K
D1 2.00000000 sec
D11 0.03000000 sec
TD0 1
======== CHANNEL f1 ========
NUC1 13C
P1 9.50 usec
PL1 3.00 dB
PL1W 59.92485046 W
SFO1 150.9178988 MHz
======== CHANNEL f2 ========
CPDPRG2 waltz16
NUC2 1H
PCPD2 80.00 usec
PL2 -1.40 dB
PL12 13.74 dB
PL13 13.70 dB
PL2W 16.22728539 W
PL12W 0.49687356 W
PL13W 0.50147104 W
SFO2 600.1324005 MHz
SI 32768
SF 150.9028090 MHz
WDW EM
SSB 0
LB 1.00 Hz
GB 0
PC 1.40
209.56
172.55
150.37
140.30
139.92
129.22
126.94
114.17
112.75
77.26
77.05
76.84
34.76
32.50
27.33
27.06
25.26
9.82
Me
O
O
12e: C15H18O2
MW: 230.31
200
180
160
140
120
100
80
60
40
20
0
ppm

NAME JAL2121Db 1H
EXPNO 1
PROCNO 1
Date_ 20170509
Time 9.02
INSTRUM spect
PROBHD 5 mm PABBO BB-
PULPROG zg30
TD 65536
SOLVENT CDCl3
NS 8
DS 2
SWH 8223.685 Hz
FIDRES 0.125483 Hz
AQ 3.9846387 sec
RG 203
DW 60.800 usec
DE 6.50 usec
TE 293.8 K
D1 1.00000000 sec
TD0 1

======== CHANNEL f1 ========
NUC1 1H
P1 10.00 usec
PL1 -2.78 dB
PL1W 27.10892296 W
SFO1 400.1724712 MHz
SI 32768
SF 400.1700091 MHz
WDW EM
SSB 0
LB 0.30 Hz
GB 0
PC 1.00

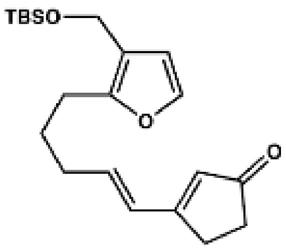

12f: $C_{21}H_{32}O_3Si$
MW: 360.57

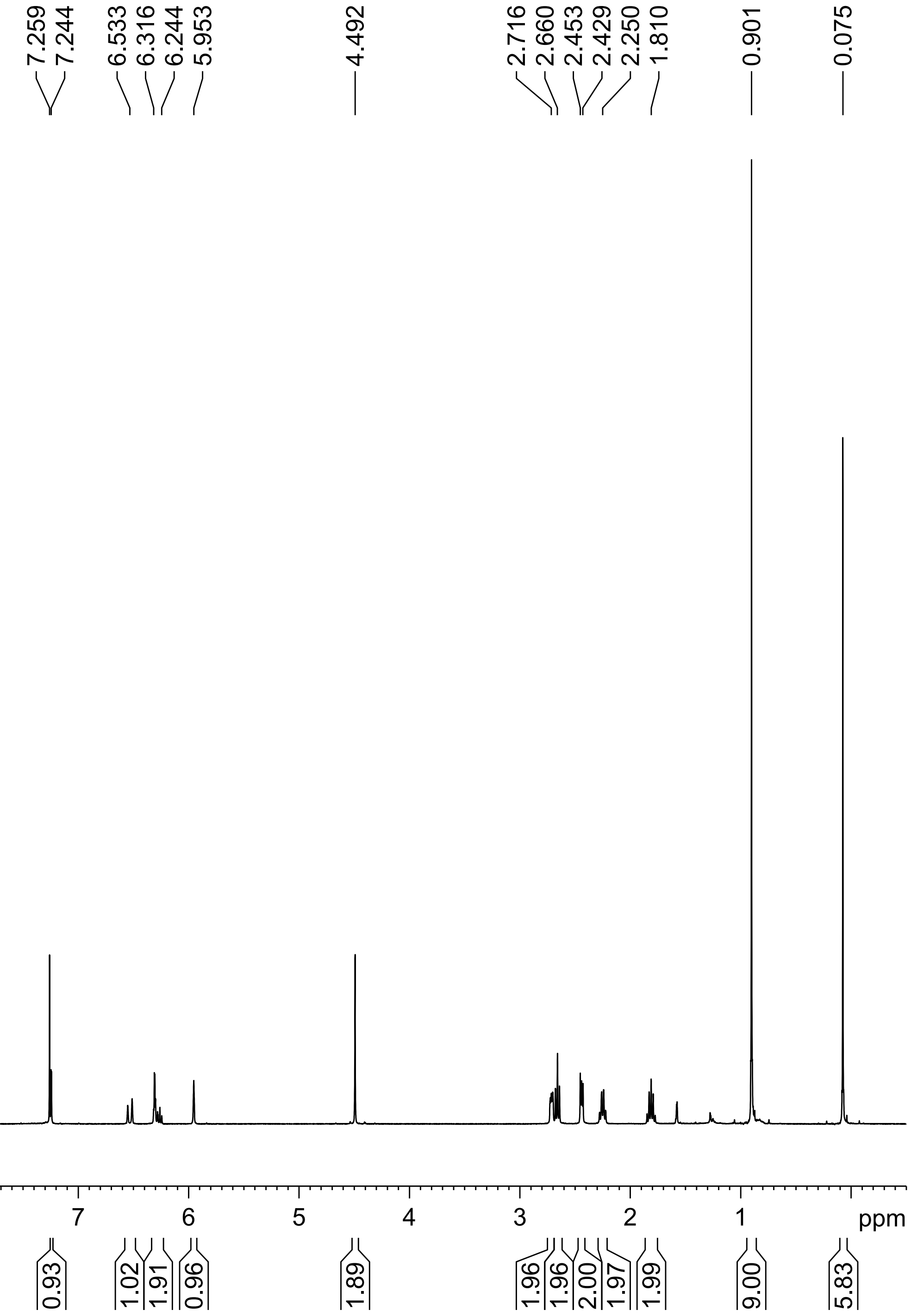

NAME JAL2121Db 13C
EXPNO 1
PROCNO 1
Date_ 20170510
Time 1.07
INSTRUM spect
PROBHD 5 mm PABBO BB-
PULPROG zgpg30
TD 65536
SOLVENT CDCl3
NS 25
DS 4
SWH 24038.461 Hz
FIDRES 0.366798 Hz
AQ 1.3631988 sec
RG 203
DW 20.800 usec
DE 6.50 usec
TE 295.1 K
D1 2.00000000 sec
D11 0.03000000 sec
TD0 1

======== CHANNEL f1 ========
NUC1 13C
P1 10.00 usec
PL1 -1.78 dB
PL1W 47.30532455 W
SFO1 100.6328888 MHz

======== CHANNEL f2 ========
CPDPRG2 waltz16
NUC2 1H
PCPD2 80.00 usec
PL2 -2.78 dB
PL12 15.28 dB
PL13 18.00 dB
PL2W 27.10892296 W
PL12W 0.42375252 W
PL13W 0.22652298 W
SFO2 400.1716007 MHz
SI 32768
SF 100.6228270 MHz
WDW EM
SSB 0
LB 1.00 Hz
GB 0
PC 1.40

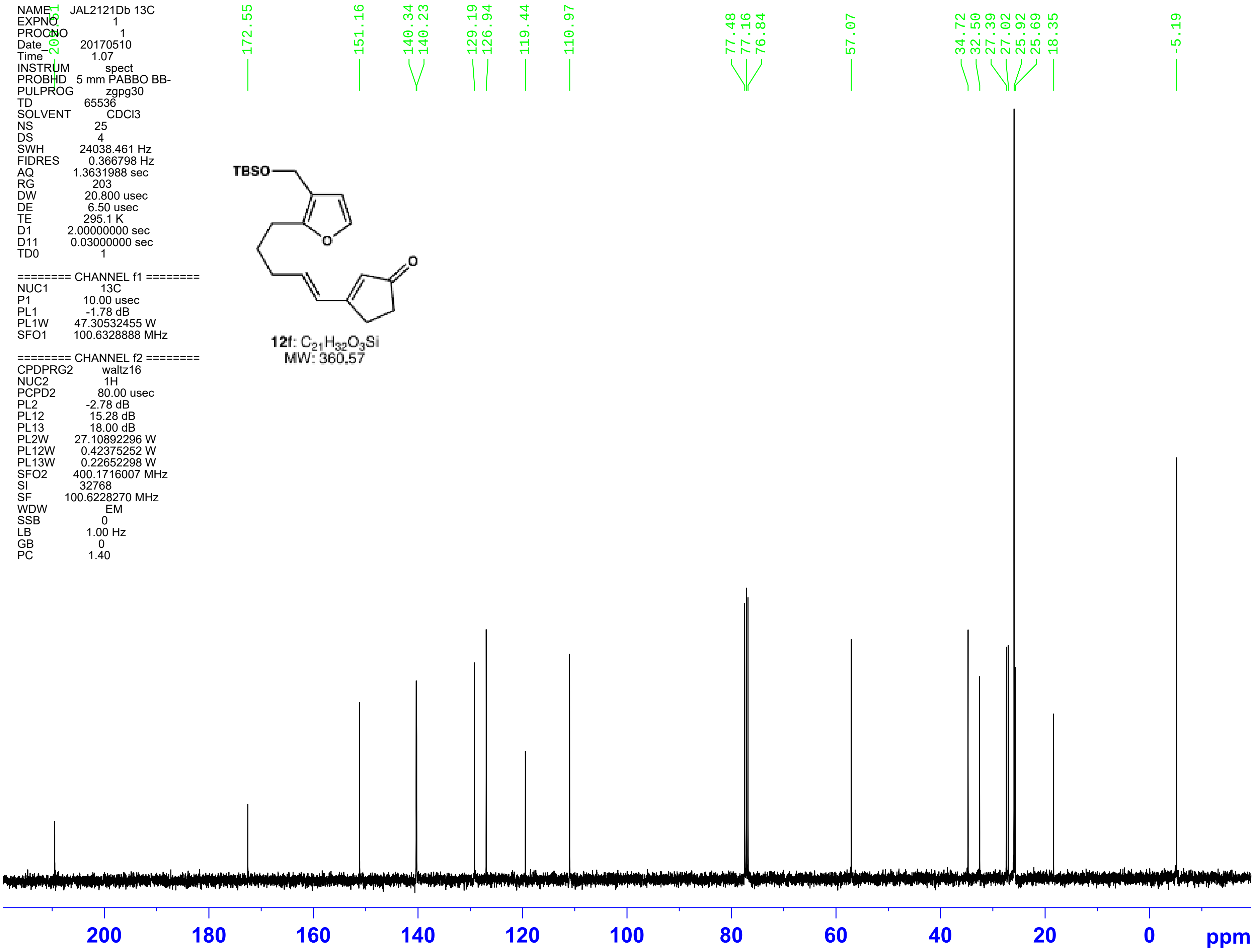

12f: $C_{21}H_{32}O_3Si$
MW: 360.57

NAME AES 4060 column 3
EXPNO 1
PROCNO 1
Date_ 20170517
Time 17.46
INSTRUM spect
PROBHD 5 mm PABBO BB-
PULPROG zg30
TD 65536
SOLVENT CDCl3
NS 10
DS 2
SWH 8223.685 Hz
FIDRES 0.125483 Hz
AQ 3.9846387 sec
RG 203
DW 60.800 usec
DE 6.50 usec
TE 294.1 K
D1 1.00000000 sec
TD0 1

======== CHANNEL f1 ========
NUC1 1H
P1 10.00 usec
PL1 -2.78 dB
PL1W 27.10892296 W
SFO1 400.1724712 MHz
SI 32768
SF 400.1700091 MHz
WDW EM
SSB 0
LB 0.30 Hz
GB 0
PC 1.00

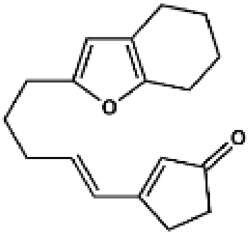

12g: $C_{18}H_{22}O_2$
MW: 270.37

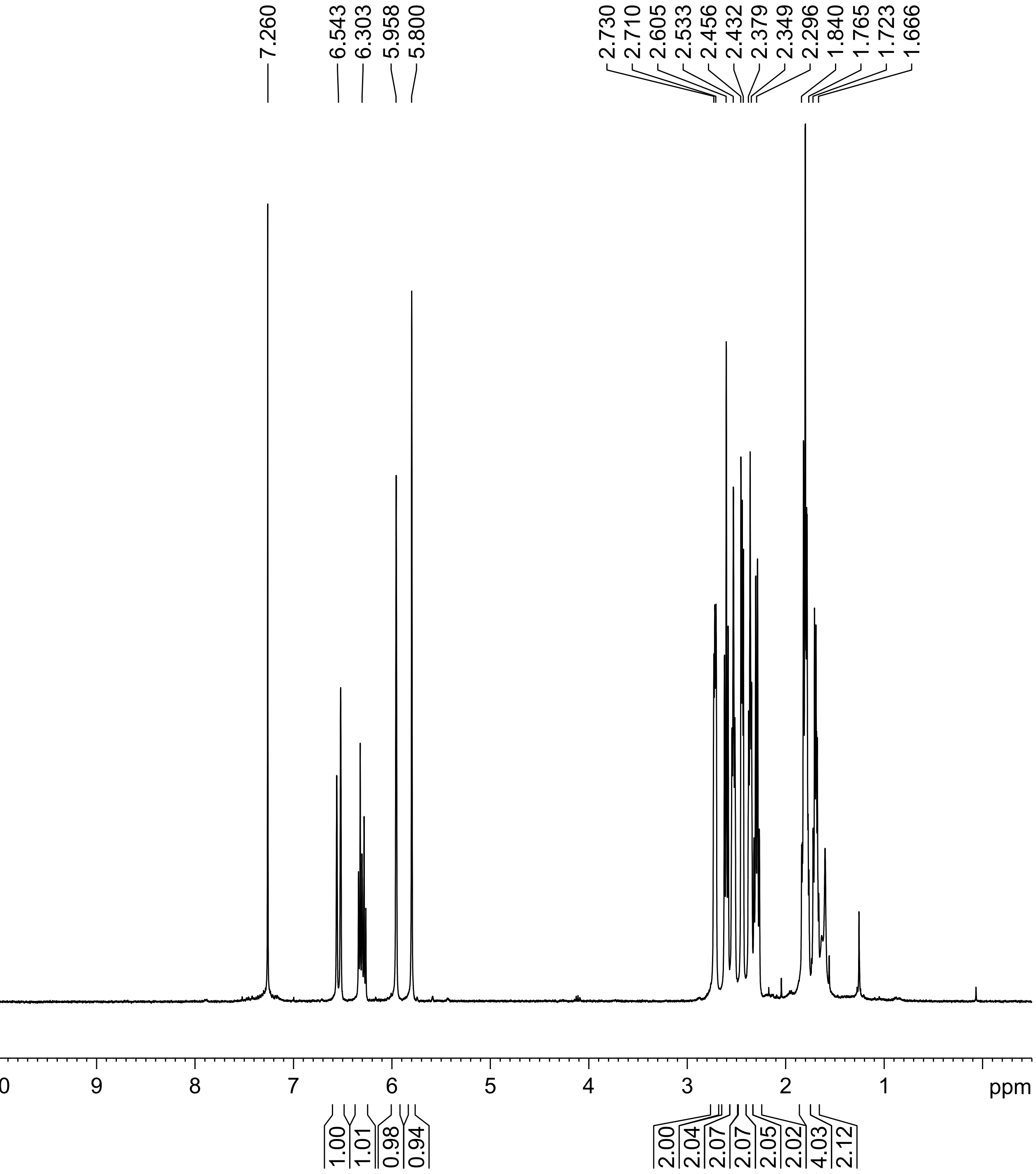

NAME AES 4060 C13
EXPNO 1
PROCNO 1
Date_ 20170517
Time 19.57
INSTRUM spect
PROBHD 5 mm PABBO BB-
PULPROG zgpg30
TD 65536
SOLVENT CDCl3
NS 256
DS 4
SWH 24038.461 Hz
FIDRES 0.366798 Hz
AQ 1.3631988 sec
RG 203
DW 20.800 usec
DE 6.50 usec
TE 295.0 K
D1 2.00000000 sec
D11 0.03000000 sec
TD0 1

======== CHANNEL f1 ========
NUC1 13C
P1 10.00 usec
PL1 -1.78 dB
PL1W 47.30532455 W
SFO1 100.6328888 MHz

======== CHANNEL f2 ========
CPDPRG2 waltz16
NUC2 1H
PCPD2 80.00 usec
PL2 -2.78 dB
PL12 15.28 dB
PL13 18.00 dB
PL2W 27.10892296 W
PL12W 0.42375252 W
PL13W 0.22652298 W
SFO2 400.1716007 MHz
SI 32768
SF 100.6228270 MHz
WDW EM
SSB 0
LB 1.00 Hz
GB 0
PC 1.40

12g: $C_{18}H_{22}O_2$
MW: 270.37

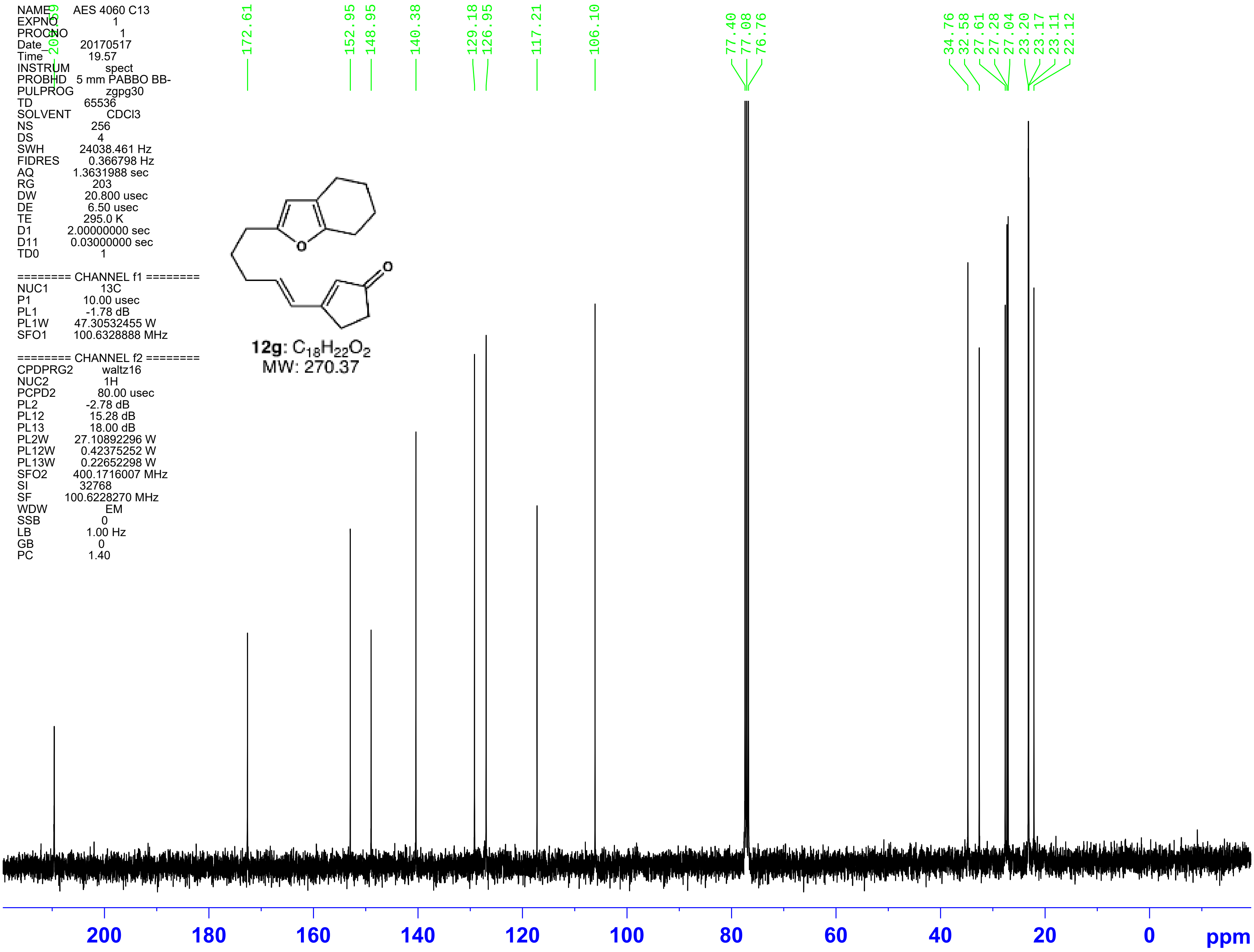

NAME JAL2290Dii 1H
EXPNO 1
PROCNO 1
Date_ 20170810
Time 22.16
INSTRUM spect
PROBHD 5 mm PABBO BB-
PULPROG zg30
TD 65536
SOLVENT CDCl3
NS 8
DS 2
SWH 8223.685 Hz
FIDRES 0.125483 Hz
AQ 3.9846387 sec
RG 181
DW 60.800 usec
DE 6.50 usec
TE 295.8 K
D1 1.00000000 sec
TD0 1

======== CHANNEL f1 ========
NUC1 1H
P1 10.00 usec
PL1 -2.78 dB
PL1W 27.10892296 W
SFO1 400.1724712 MHz
SI 32768
SF 400.1700108 MHz
WDW EM
SSB 0
LB 0.30 Hz
GB 0
PC 1.00

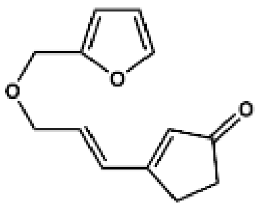

12h: $C_{13}H_{14}O_3$
MW: 218.25

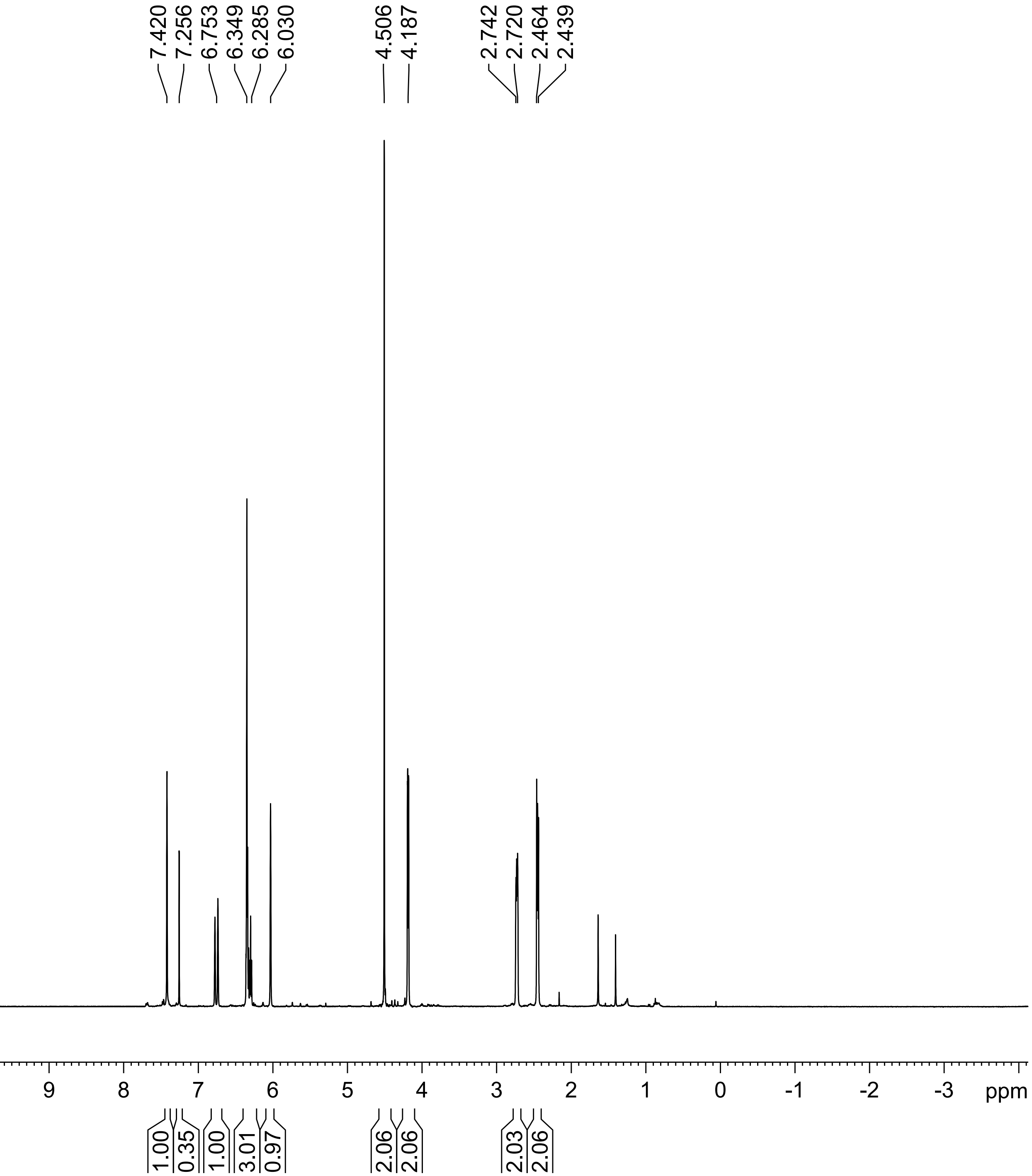

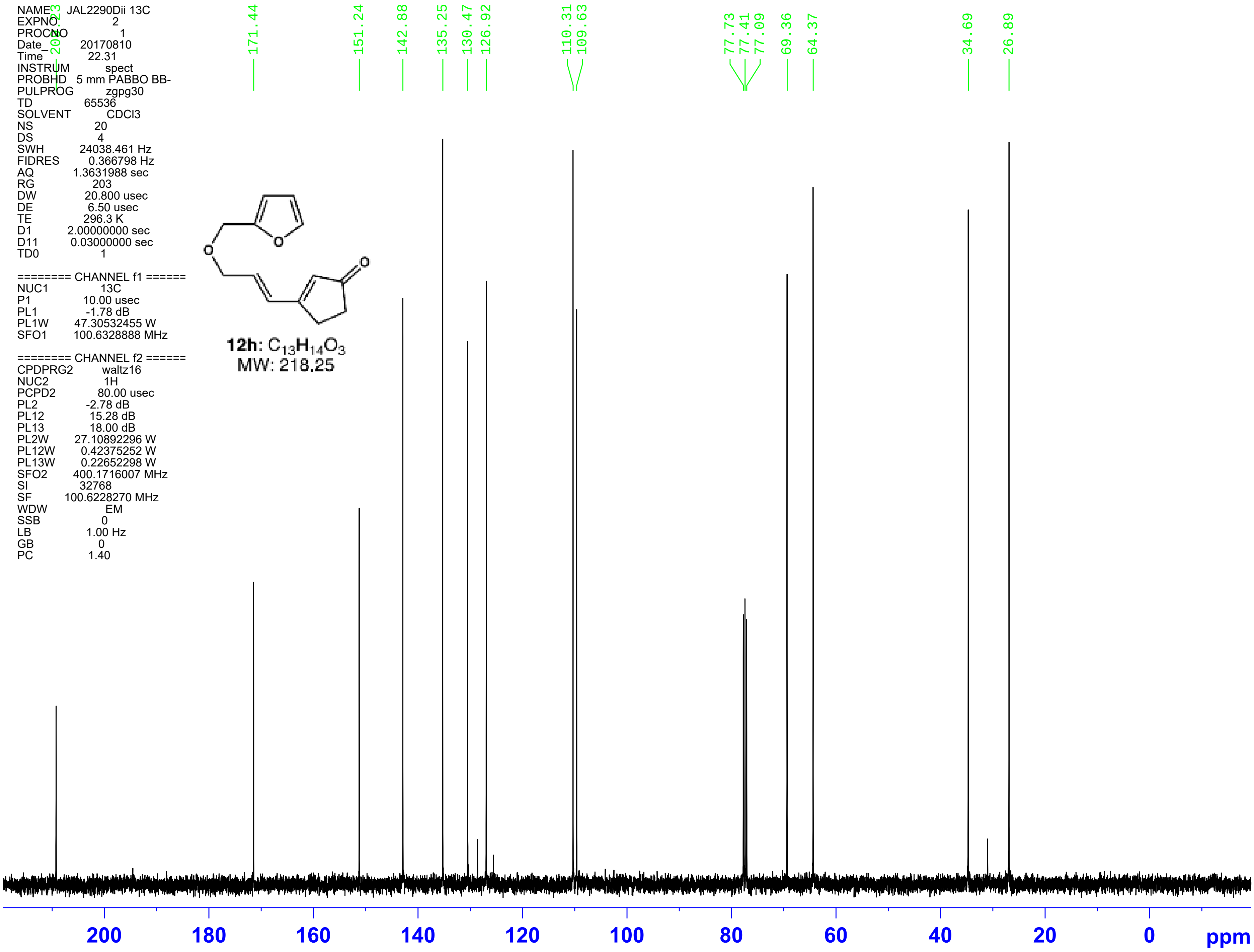

12h: $C_{13}H_{14}O_3$
MW: 218.25

NAME JAL3217C 1H
EXPNO 1
PROCNO 1
Date_ 20171214
Time 14.05
INSTRUM spect
PROBHD 5 mm PABBO BB-
PULPROG zg30
TD 65536
SOLVENT CDCl3
NS 8
DS 2
SWH 8223.685 Hz
FIDRES 0.125483 Hz
AQ 3.9846387 sec
RG 203
DW 60.800 usec
DE 6.50 usec
TE 294.8 K
D1 1.00000000 sec
TD0 1

======== CHANNEL f1 ========
NUC1 1H
P1 10.00 usec
PL1 -2.78 dB
PL1W 27.10892296 W
SFO1 400.1724712 MHz
SI 32768
SF 400.1700085 MHz
WDW EM
SSB 0
LB 0.30 Hz
GB 0
PC 1.00

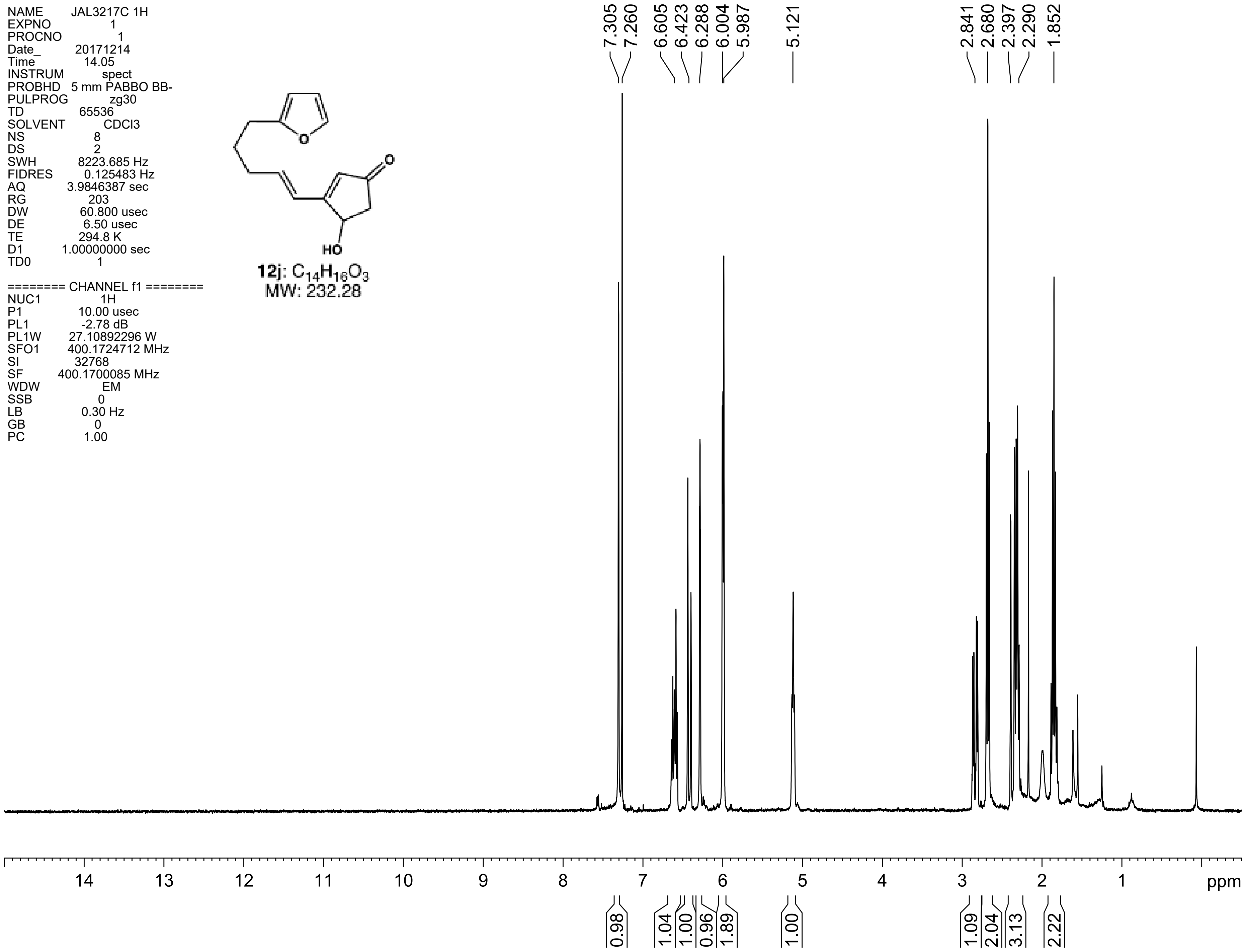

12j: $C_{14}H_{16}O_3$
MW: 232.28

NAME JAL2126A 13C
EXPNO 1
PROCNO 1
Date_ 20170511
Time 0.15
INSTRUM spect
PROBHD 5 mm PABBO BB-
PULPROG zgpg30
TD 65536
SOLVENT CDCl3
NS 385
DS 4
SWH 24038.461 Hz
FIDRES 0.366798 Hz
AQ 1.3631988 sec
RG 203
DW 20.800 usec
DE 6.50 usec
TE 295.1 K
D1 2.00000000 sec
D11 0.03000000 sec
TD0 1

======== CHANNEL f1 ========
NUC1 13C
P1 10.00 usec
PL1 -1.78 dB
PL1W 47.30532455 W
SFO1 100.6328888 MHz

======== CHANNEL f2 ========
CPDPRG2 waltz16
NUC2 1H
PCPD2 80.00 usec
PL2 -2.78 dB
PL12 15.28 dB
PL13 18.00 dB
PL2W 27.10892296 W
PL12W 0.42375252 W
PL13W 0.22652298 W
SFO2 400.1716007 MHz
SI 32768
SF 100.6228270 MHz
WDW EM
SSB 0
LB 1.00 Hz
GB 0
PC 1.40

12j: $C_{14}H_{16}O_3$
MW: 232.28

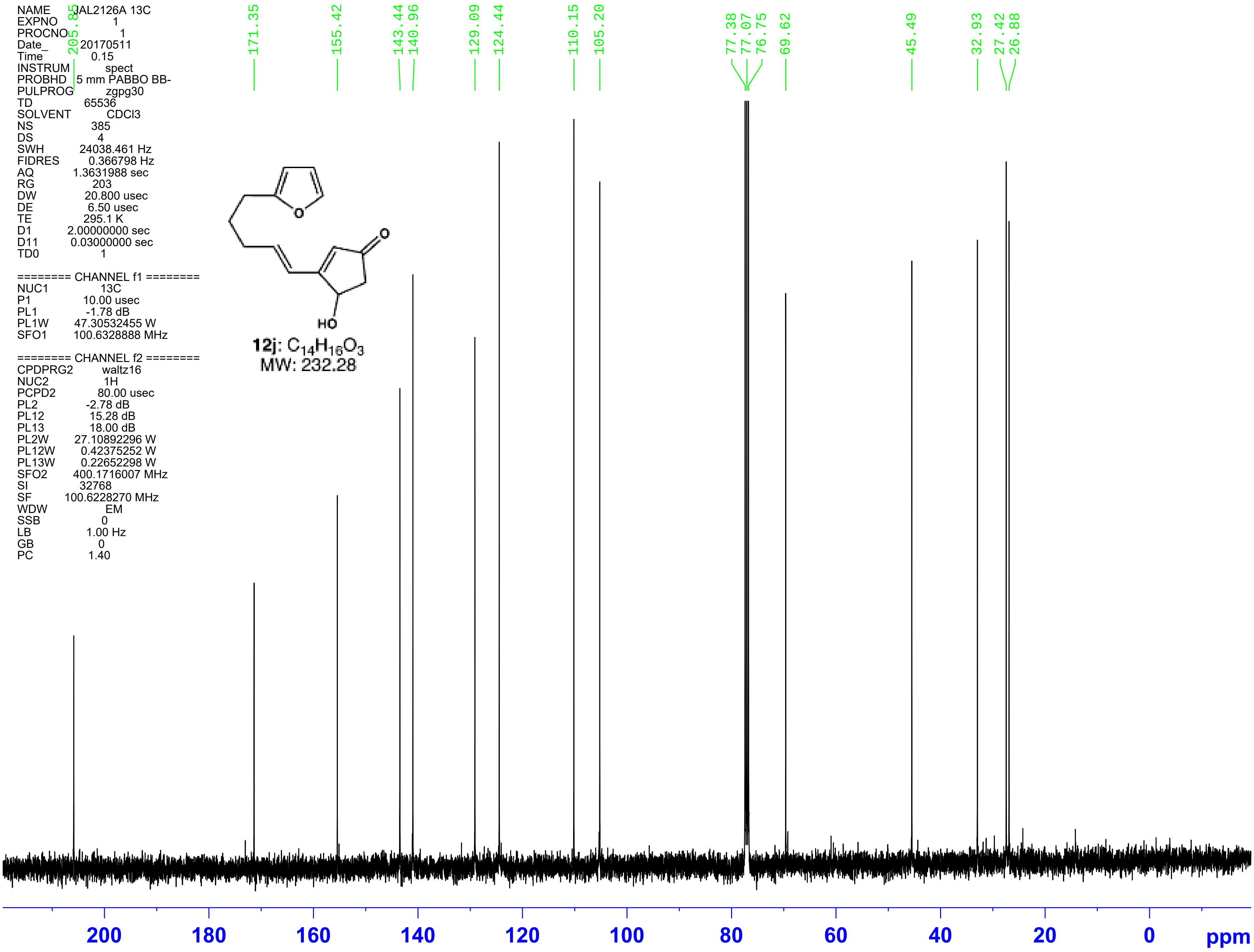

NAME JAL3146C 1H
EXPNO 2
PROCNO 1
Date_ 20171027
Time 9.52
INSTRUM spect
PROBHD 5 mm PABBO BB-
PULPROG zg30
TD 65536
SOLVENT CDCl3
NS 8
DS 2
SWH 8223.685 Hz
FIDRES 0.125483 Hz
AQ 3.9846387 sec
RG 203
DW 60.800 usec
DE 6.50 usec
TE 294.2 K
D1 1.00000000 sec
TD0 1

======== CHANNEL f1 ========
NUC1 1H
P1 10.00 usec
PL1 -2.78 dB
PL1W 27.10892296 W
SFO1 400.1724712 MHz
SI 32768
SF 400.1700088 MHz
WDW EM
SSB 0
LB 0.30 Hz
GB 0
PC 1.00

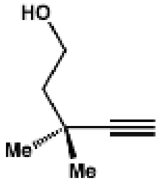

S10: $C_7H_{12}O$
MW: 112.17

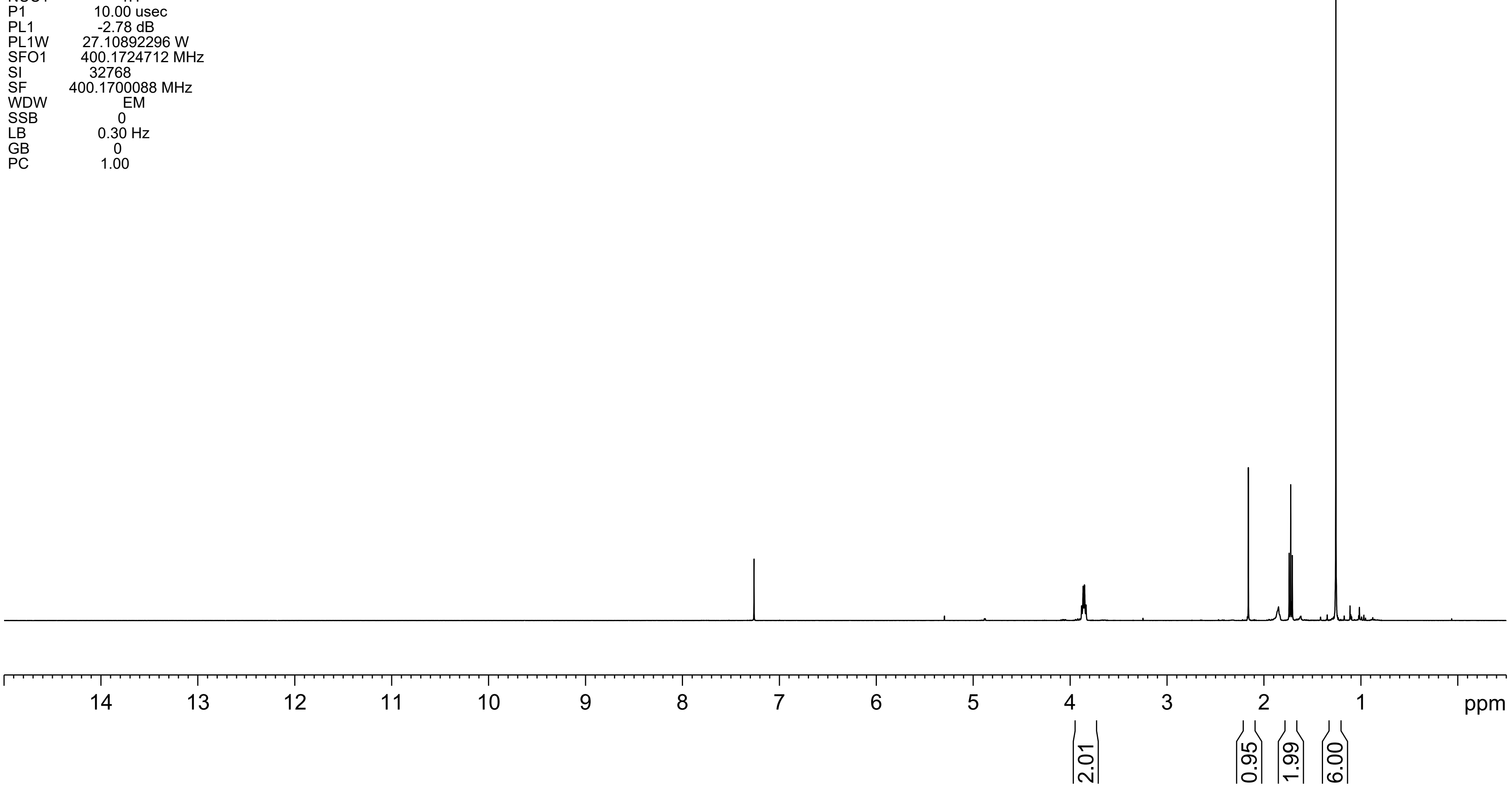

NAME JAL3056A 1H
EXPNO 1
PROCNO 1
Date_ 20170916
Time 14.34
INSTRUM spect
PROBHD 5 mm PABBO BB-
PULPROG zg30
TD 65536
SOLVENT CDCl3
NS 8
DS 2
SWH 8223.685 Hz
FIDRES 0.125483 Hz
AQ 3.9846387 sec
RG 101
DW 60.800 usec
DE 6.50 usec
TE 293.5 K
D1 1.00000000 sec
TD0 1

======== CHANNEL f1 ========
NUC1 1H
P1 10.00 usec
PL1 -2.78 dB
PL1W 27.10892296 W
SFO1 400.1724712 MHz
SI 32768
SF 400.1700091 MHz
WDW EM
SSB 0
LB 0.30 Hz
GB 0
PC 1.00

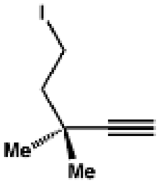

S11: $C_7H_{11}I$
MW: 222.07

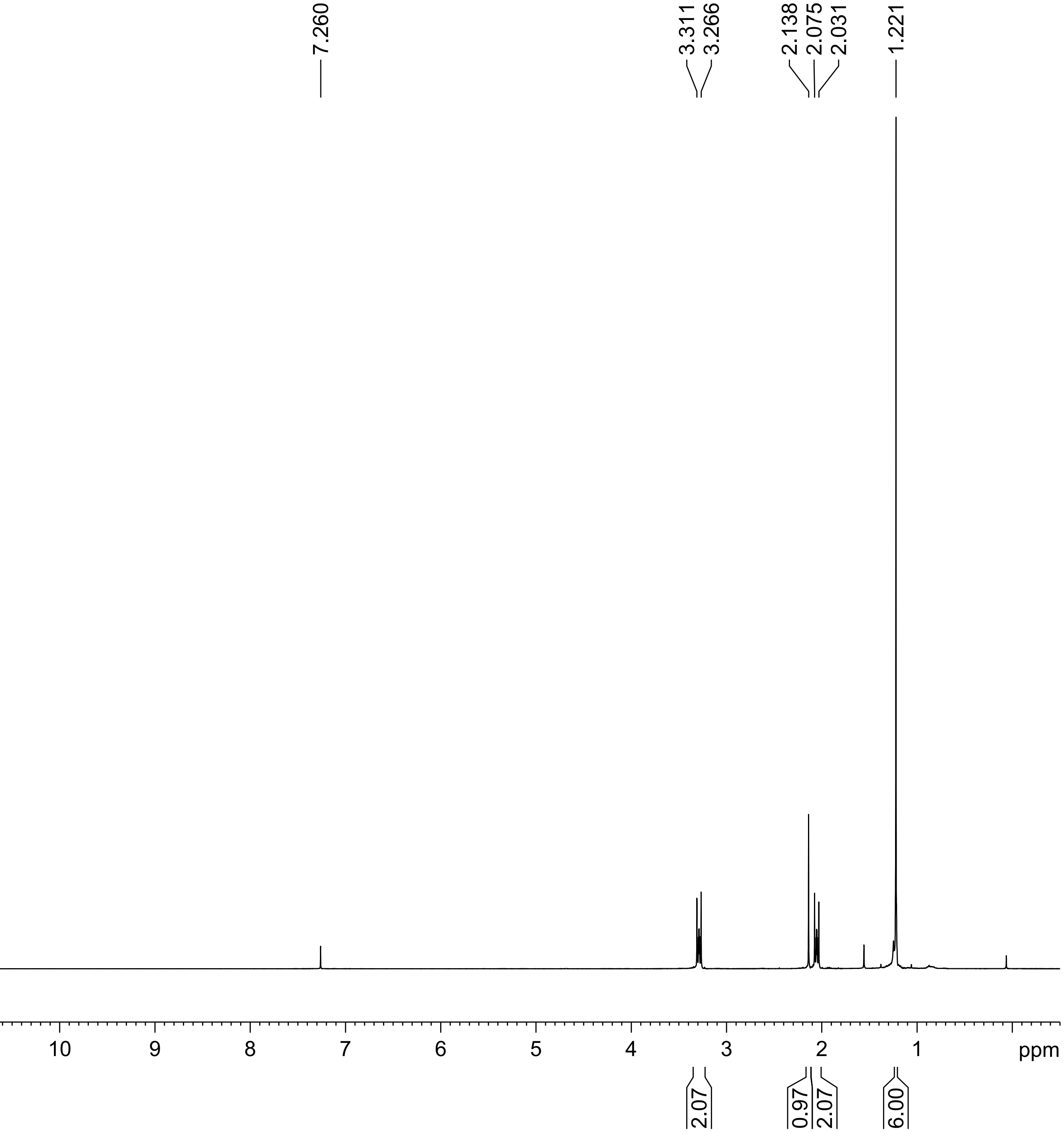

NAME JAL3056A 13C
EXPNO 1
PROCNO 1
Date_ 20170916
Time 14.49
INSTRUM spect
PROBHD 5 mm PABBO BB-
PULPROG zgpg30
TD 65536
SOLVENT CDCl3
NS 150
DS 4
SWH 24038.461 Hz
FIDRES 0.366798 Hz
AQ 1.3631988 sec
RG 203
DW 20.800 usec
DE 6.50 usec
TE 294.4 K
D1 2.00000000 sec
D11 0.03000000 sec
TD0 1

======== CHANNEL f1 ========
NUC1 13C
P1 10.00 usec
PL1 -1.78 dB
PL1W 47.30532455 W
SFO1 100.6328888 MHz

======== CHANNEL f2 ========
CPDPRG2 waltz16
NUC2 1H
PCPD2 80.00 usec
PL2 -2.78 dB
PL12 15.28 dB
PL13 18.00 dB
PL2W 27.10892296 W
PL12W 0.42375252 W
PL13W 0.22652298 W
SFO2 400.1716007 MHz
SI 32768
SF 100.6228270 MHz
WDW EM
SSB 0
LB 1.00 Hz
GB 0
PC 1.40

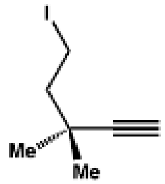

S11: $C_7H_{11}I$
MW: 222.07

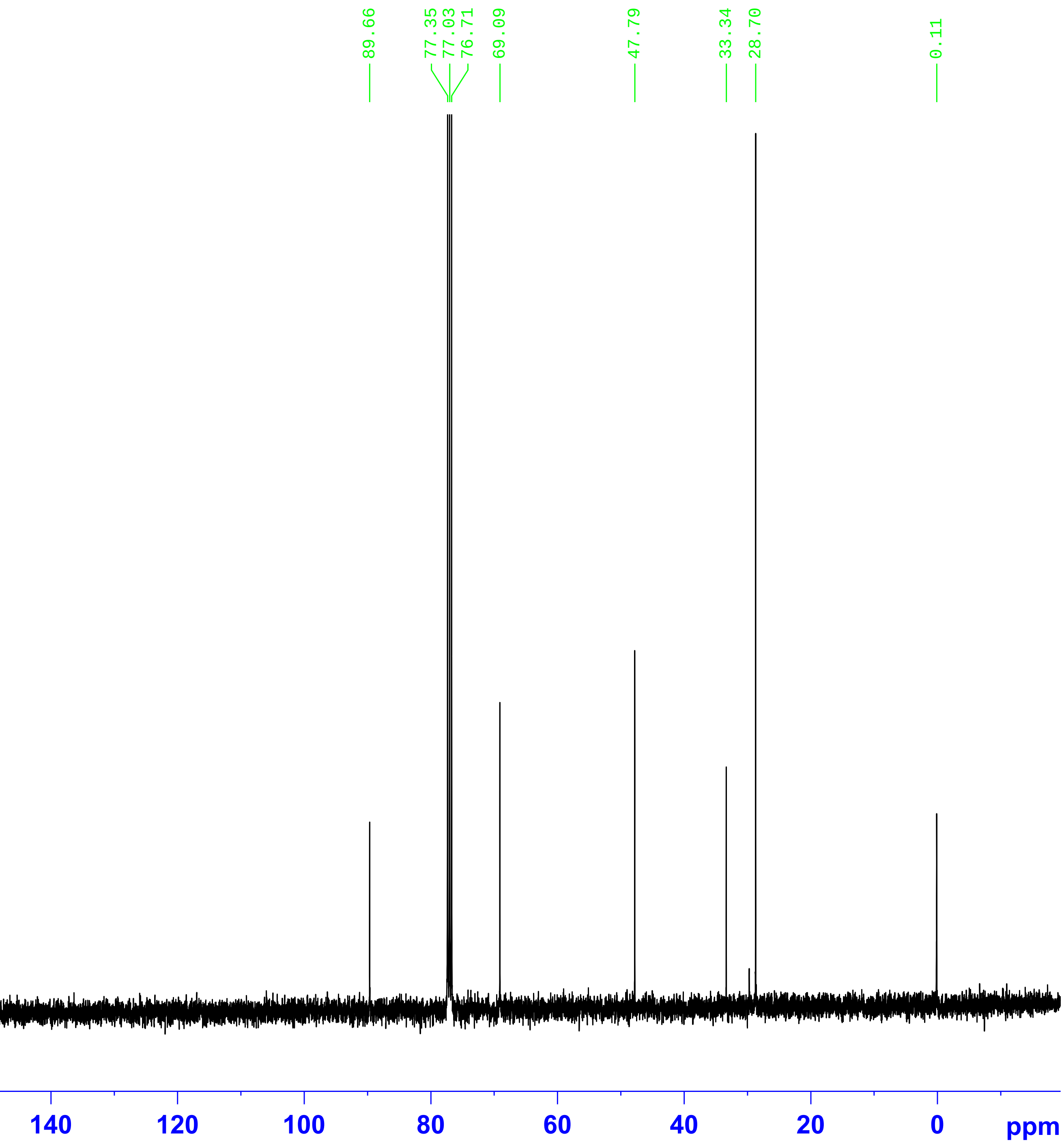

NAME JAL3144AB 1H
EXPNO 2
PROCNO 1
Date_ 20171027
Time 9.39
INSTRUM spect
PROBHD 5 mm PABBO BB-
PULPROG zg30
TD 65536
SOLVENT CDCl3
NS 8
DS 2
SWH 8223.685 Hz
FIDRES 0.125483 Hz
AQ 3.9846387 sec
RG 144
DW 60.800 usec
DE 6.50 usec
TE 294.5 K
D1 1.00000000 sec
TD0 1

======== CHANNEL f1 ========
NUC1 1H
P1 10.00 usec
PL1 -2.78 dB
PL1W 27.10892296 W
SFO1 400.1724712 MHz
SI 32768
SF 400.1700088 MHz
WDW EM
SSB 0
LB 0.30 Hz
GB 0
PC 1.00

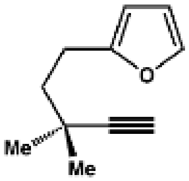

S12: $C_{11}H_{14}O$
MW: 162.23

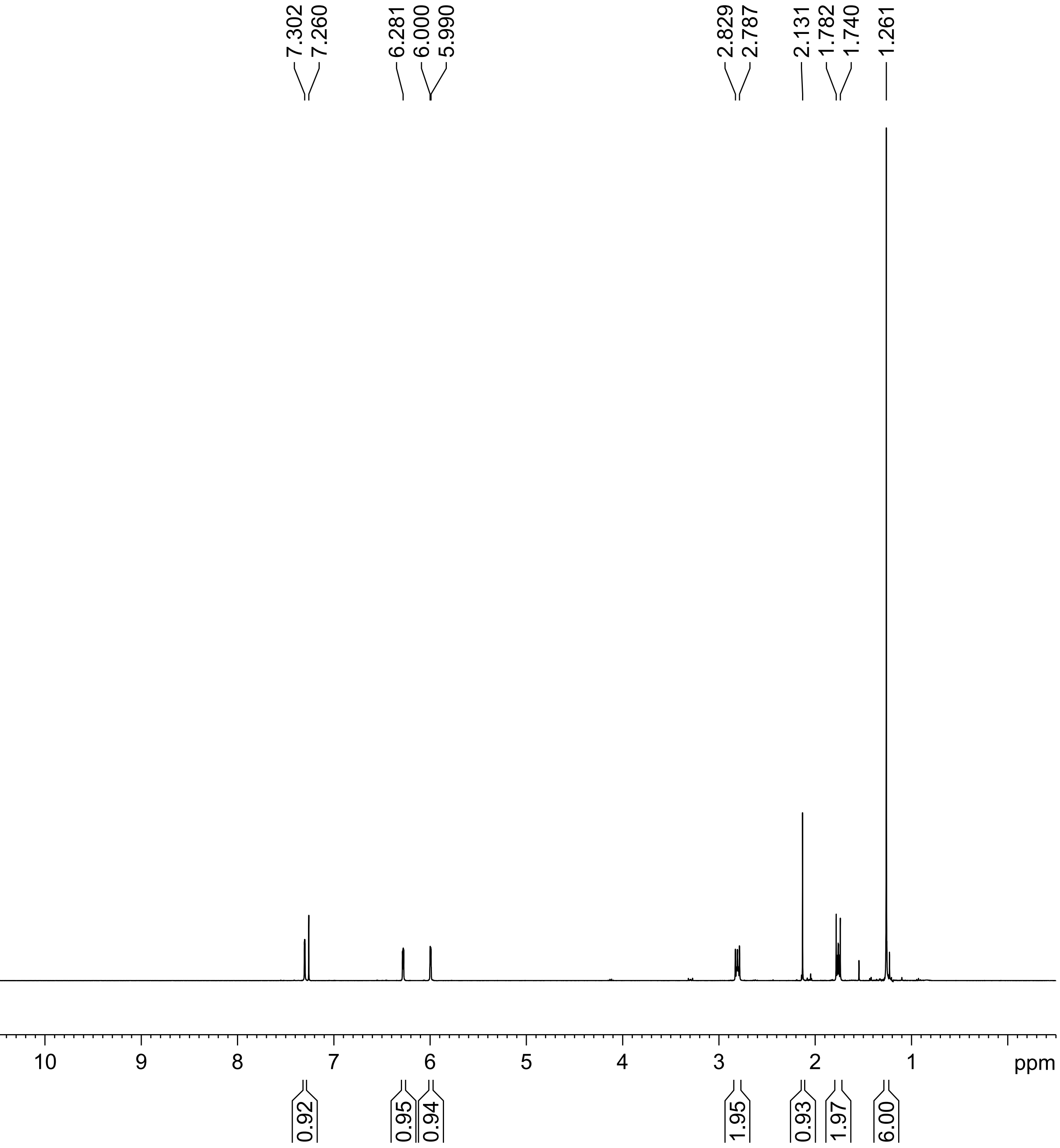

NAME JAL3144AB 13C
EXPNO 3
PROCNO 1
Date_ 20171027
Time 18.13
INSTRUM spect
PROBHD 5 mm PABBO BB-
PULPROG zgpg30
TD 65536
SOLVENT CDCl3
NS 10
DS 4
SWH 24038.461 Hz
FIDRES 0.366798 Hz
AQ 1.3631988 sec
RG 203
DW 20.800 usec
DE 6.50 usec
TE 294.0 K
D1 2.00000000 sec
D11 0.03000000 sec
TD0 1

======== CHANNEL f1 ========
NUC1 13C
P1 10.00 usec
PL1 -1.78 dB
PL1W 47.30532455 W
SFO1 100.6328888 MHz

======== CHANNEL f2 ========
CPDPRG2 waltz16
NUC2 1H
PCPD2 80.00 usec
PL2 -2.78 dB
PL12 15.28 dB
PL13 18.00 dB
PL2W 27.10892296 W
PL12W 0.42375252 W
PL13W 0.22652298 W
SFO2 400.1716007 MHz
SI 32768
SF 100.6228270 MHz
WDW EM
SSB 0
LB 1.00 Hz
GB 0
PC 1.40

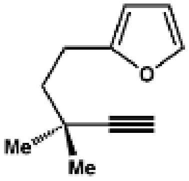

S12: $C_{11}H_{14}O$
MW: 162.23

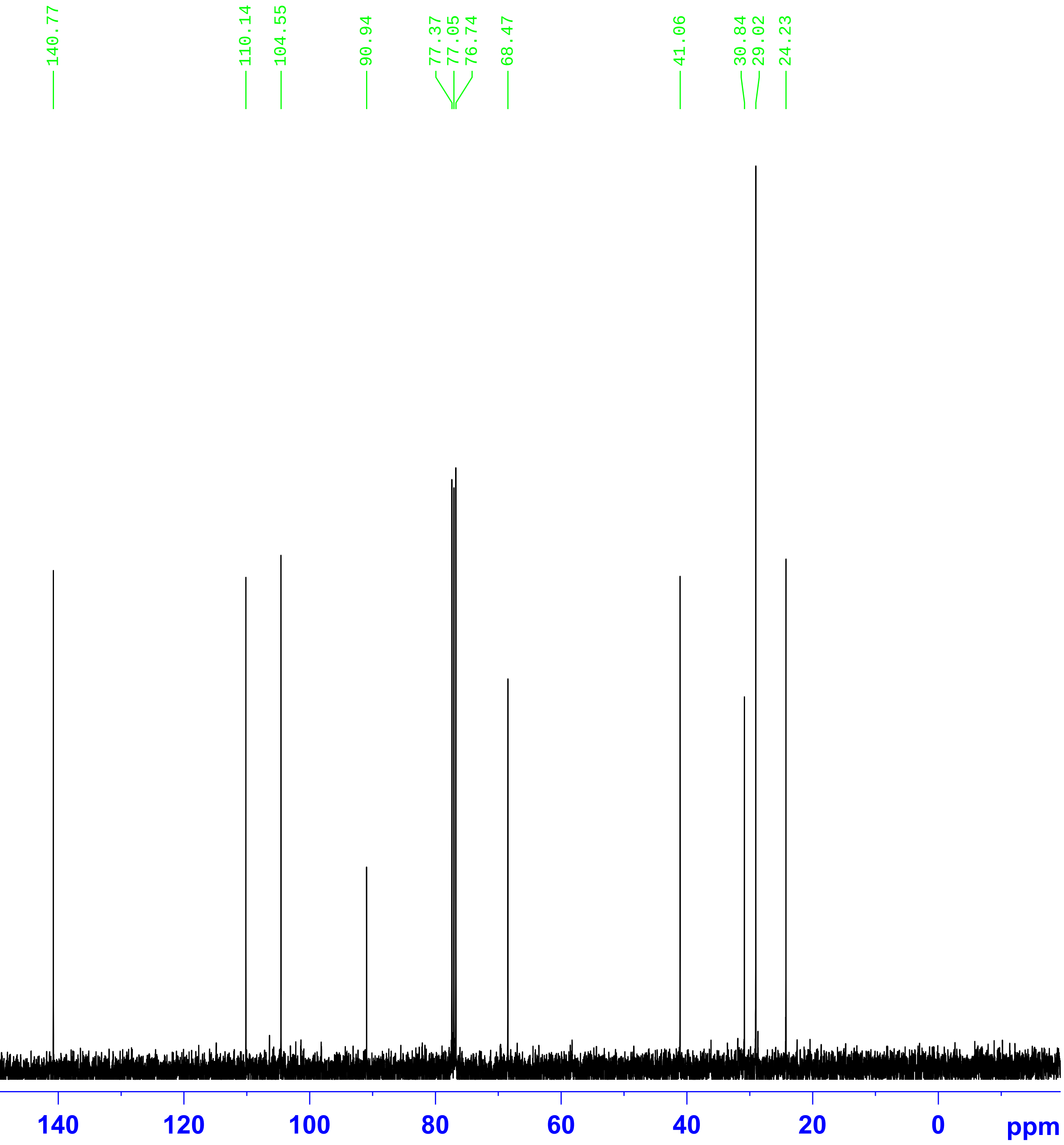

NAME JAL3214Ai 1H
EXPNO 1
PROCNO 1
Date_ 20171211
Time 21.41
INSTRUM spect
PROBHD 5 mm PABBO BB-
PULPROG zg30
TD 65536
SOLVENT CDCl3
NS 8
DS 2
SWH 8223.685 Hz
FIDRES 0.125483 Hz
AQ 3.9846387 sec
RG 203
DW 60.800 usec
DE 6.50 usec
TE 294.8 K
D1 1.00000000 sec
TD0 1

======== CHANNEL f1 ========
NUC1 1H
P1 10.00 usec
PL1 -2.78 dB
PL1W 27.10892296 W
SFO1 400.1724712 MHz
SI 32768
SF 400.1700083 MHz
WDW EM
SSB 0
LB 0.30 Hz
GB 0
PC 1.00

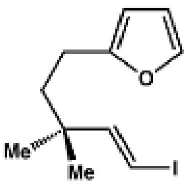

S13: $C_{11}H_{15}IO$
MW: 290.14

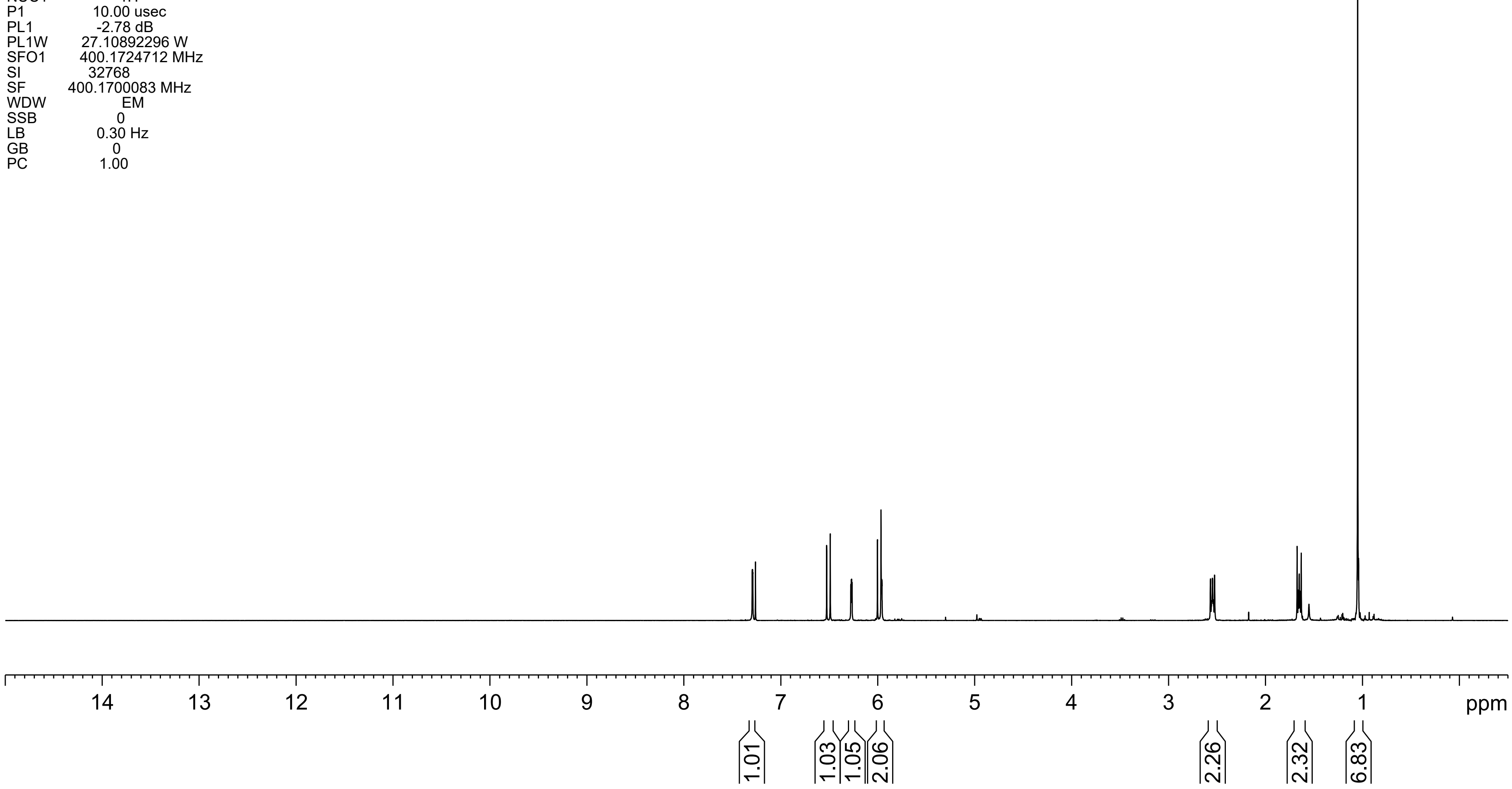

NAME JAL3214Ai 13C
EXPNO 1
PROCNO 1
Date_ 20171211
Time 23.26
INSTRUM spect
PROBHD 5 mm PABBO BB-
PULPROG zgpg30
TD 65536
SOLVENT CDCl3
NS 315
DS 4
SWH 24038.461 Hz
FIDRES 0.366798 Hz
AQ 1.3631988 sec
RG 203
DW 20.800 usec
DE 6.50 usec
TE 295.6 K
D1 2.00000000 sec
D11 0.03000000 sec
TD0 1

======== CHANNEL f1 ========
NUC1 13C
P1 10.00 usec
PL1 -1.78 dB
PL1W 47.30532455 W
SFO1 100.6328888 MHz

======== CHANNEL f2 ========
CPDPRG2 waltz16
NUC2 1H
PCPD2 80.00 usec
PL2 -2.78 dB
PL12 15.28 dB
PL13 18.00 dB
PL2W 27.10892296 W
PL12W 0.42375252 W
PL13W 0.22652298 W
SFO2 400.1716007 MHz
SI 32768
SF 100.6228270 MHz
WDW EM
SSB 0
LB 1.00 Hz
GB 0
PC 1.40

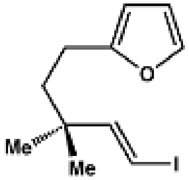

S13: $C_{11}H_{15}IO$
MW: 290.14

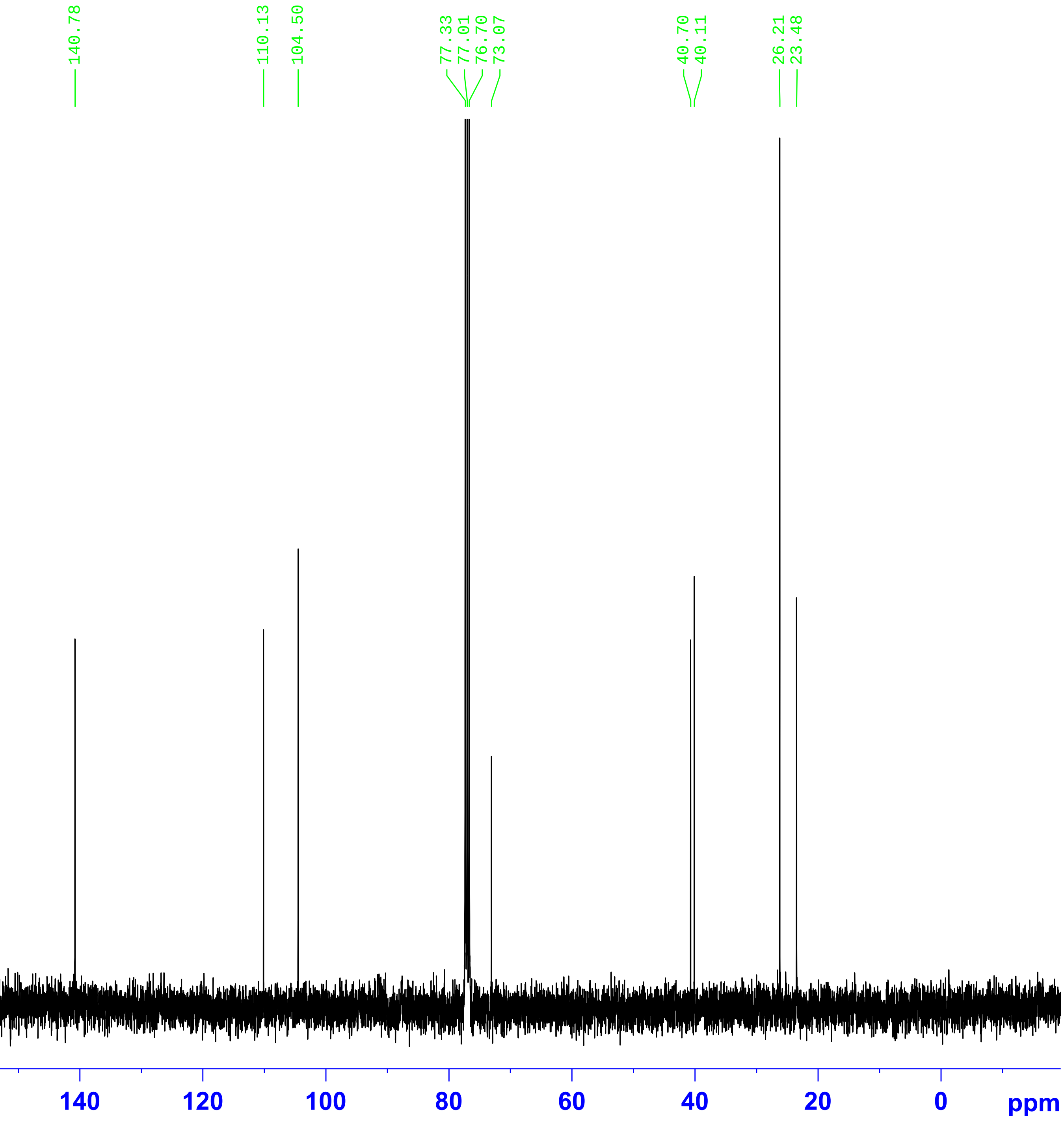

NAME JAL3216C 1H
EXPNO 1
PROCNO 1
Date_ 20171212
Time 22.48
INSTRUM spect
PROBHD 5 mm PABBO BB-
PULPROG zg30
TD 65536
SOLVENT CDCl3
NS 8
DS 2
SWH 8223.685 Hz
FIDRES 0.125483 Hz
AQ 3.9846387 sec
RG 203
DW 60.800 usec
DE 6.50 usec
TE 294.7 K
D1 1.00000000 sec
TD0 1

======== CHANNEL f1 ========
NUC1 1H
P1 10.00 usec
PL1 -2.78 dB
PL1W 27.10892296 W
SFO1 400.1724712 MHz
SI 32768
SF 400.1700087 MHz
WDW EM
SSB 0
LB 0.30 Hz
GB 0
PC 1.00

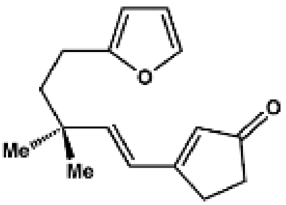

12i: $C_{16}H_{20}O_2$
MW: 244.33

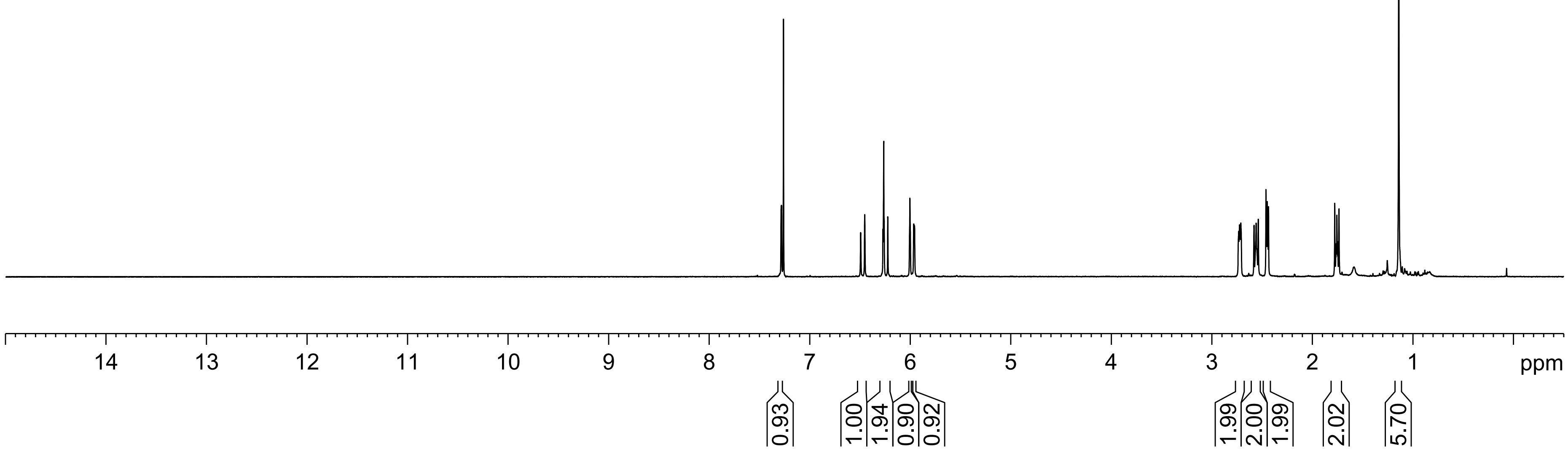

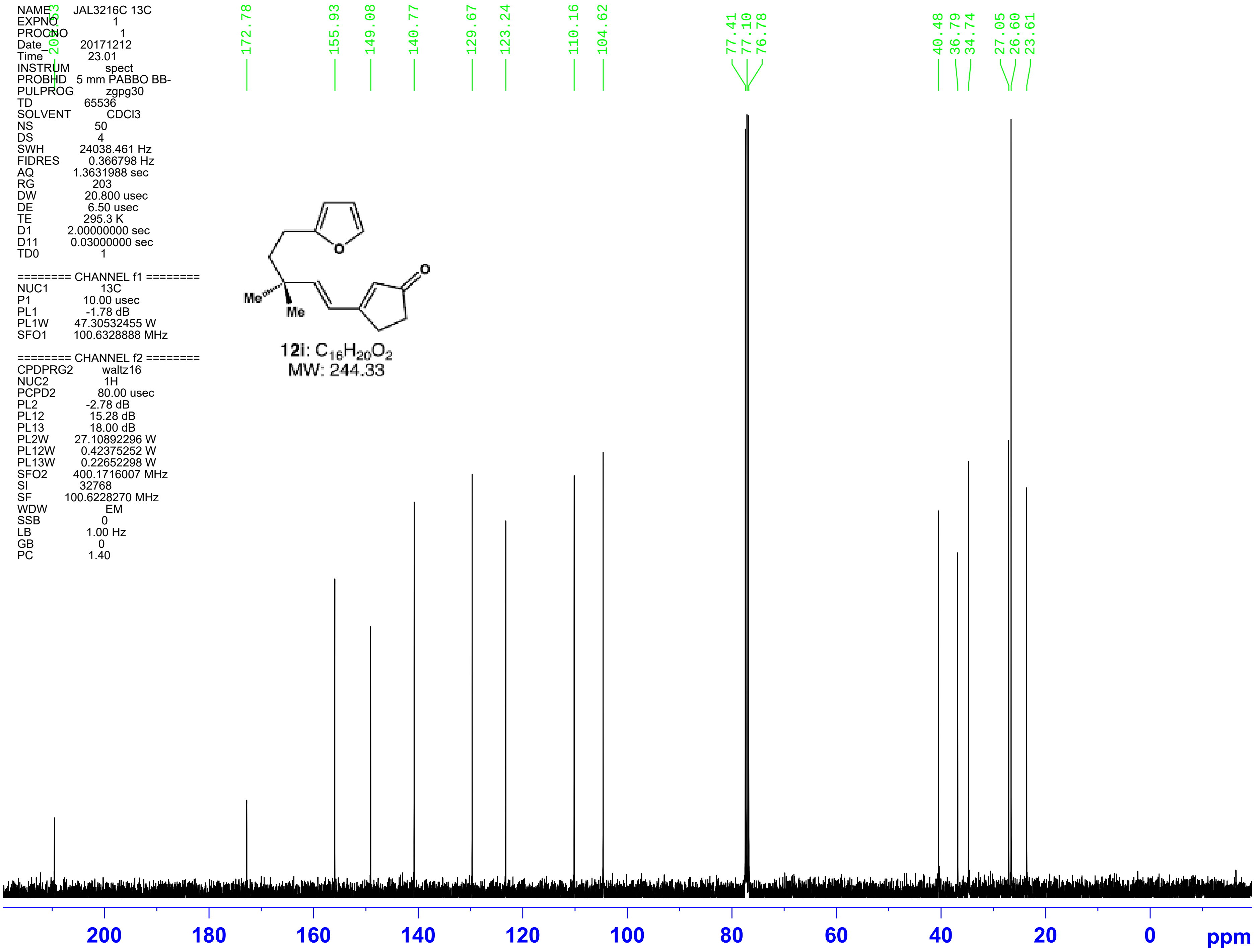
NAME JAL3216C 13C
EXPNO 1
PROCNO 1
Date_ 20171212
Time 23.01
INSTRUM spect
PROBHD 5 mm PABBO BB-
PULPROG zgpg30
TD 65536
SOLVENT CDCl3
NS 50
DS 4
SWH 24038.461 Hz
FIDRES 0.366798 Hz
AQ 1.3631988 sec
RG 203
DW 20.800 usec
DE 6.50 usec
TE 295.3 K
D1 2.00000000 sec
D11 0.03000000 sec
TD0 1
======== CHANNEL f1 ========
NUC1 13C
P1 10.00 usec
PL1 -1.78 dB
PL1W 47.30532455 W
SFO1 100.6328888 MHz
======== CHANNEL f2 ========
CPDPRG2 waltz16
NUC2 1H
PCPD2 80.00 usec
PL2 -2.78 dB
PL12 15.28 dB
PL13 18.00 dB
PL2W 27.10892296 W
PL12W 0.42375252 W
PL13W 0.22652298 W
SFO2 400.1716007 MHz
SI 32768
SF 100.6228270 MHz
WDW EM
SSB 0
LB 1.00 Hz
GB 0
PC 1.40
172.78
155.93
149.08
140.77
129.67
123.24
110.16
104.62
77.41
77.10
76.78
40.48
36.79
34.74
27.05
26.60
23.61
Me
Me
O
O
12i: C16H20O2
MW: 244.33
200
180
160
140
120
100
80
60
40
20
0
ppm

NAME AES 2233c
EXPNO 1
PROCNO 1
Date_ 20160615
Time 2.04
INSTRUM spect
PROBHD 5 mm PABBO BB-
PULPROG zg30
TD 65536
SOLVENT CDCl3
NS 16
DS 2
SWH 8223.685 Hz
FIDRES 0.125483 Hz
AQ 3.9846387 sec
RG 57
DW 60.800 usec
DE 6.50 usec
TE 293.6 K
D1 1.00000000 sec
TD0 1

======== CHANNEL f1 ========
NUC1 1H
P1 10.00 usec
PL1 -2.78 dB
PL1W 27.10892296 W
SFO1 400.1724712 MHz
SI 32768
SF 400.1700088 MHz
WDW EM
SSB 0
LB 0.30 Hz
GB 0
PC 1.00

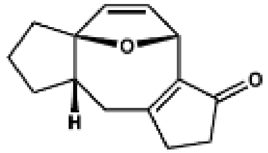

3: $C_{14}H_{16}O_2$
MW: 216.28

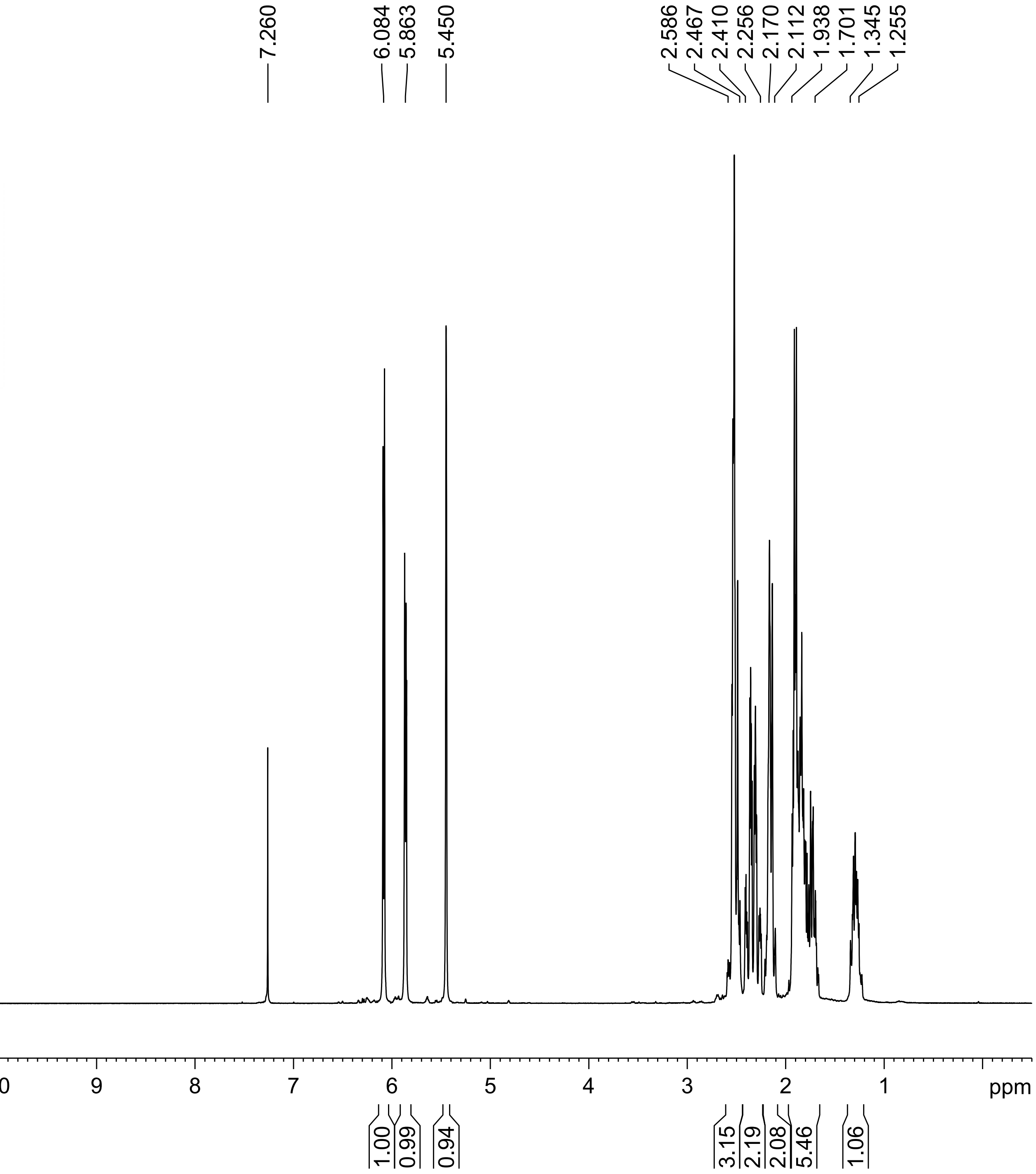

NAME AES 2235 pure C13
EXPNO 1
PROCNO 1
Date_ 20160618
Time 3.31
INSTRUM spect
PROBHD 5 mm PABBO BB-
PULPROG zgpg30
TD 65536
SOLVENT CDCl3
NS 256
DS 4
SWH 24038.461 Hz
FIDRES 0.366798 Hz
AQ 1.3631988 sec
RG 203
DW 20.800 usec
DE 6.50 usec
TE 294.8 K
D1 2.00000000 sec
D11 0.03000000 sec
TD0 1

======== CHANNEL f1 ========
NUC1 13C
P1 10.00 usec
PL1 -1.78 dB
PL1W 47.30532455 W
SFO1 100.6328888 MHz

======== CHANNEL f2 ========
CPDPRG2 waltz16
NUC2 1H
PCPD2 80.00 usec
PL2 -2.78 dB
PL12 15.28 dB
PL13 18.00 dB
PL2W 27.10892296 W
PL12W 0.42375252 W
PL13W 0.22652298 W
SFO2 400.1716007 MHz
SI 32768
SF 100.6228270 MHz
WDW EM
SSB 0
LB 1.00 Hz
GB 0
PC 1.40

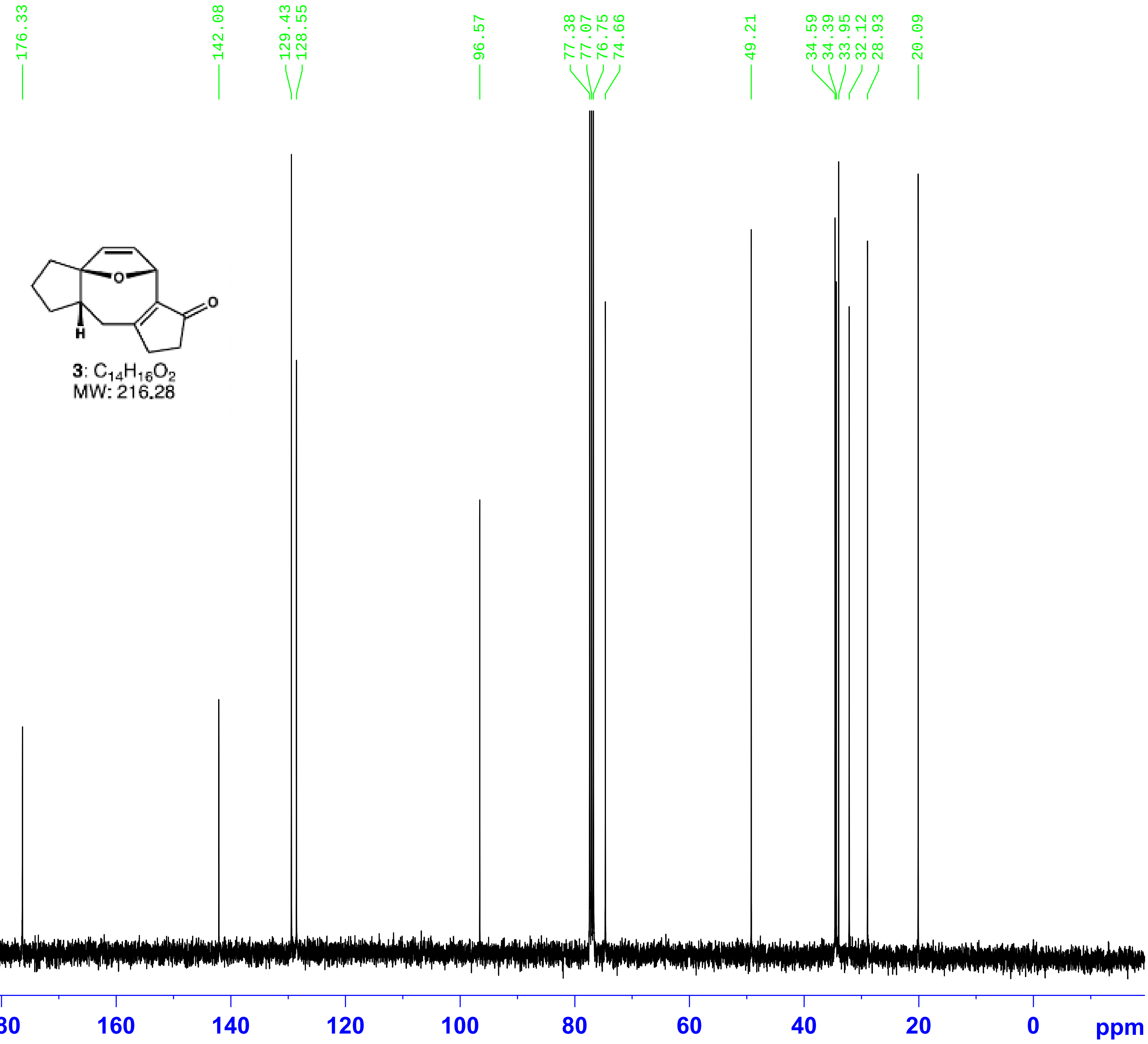

NAME AES 2181 1H
EXPNO 1
PROCNO 1
Date_ 20160511
Time 18.28
INSTRUM spect
PROBHD 5 mm PABBO BB-
PULPROG zg30
TD 65536
SOLVENT CDCl3
NS 16
DS 2
SWH 12335.526 Hz
FIDRES 0.188225 Hz
AQ 2.6564426 sec
RG 144
DW 40.533 usec
DE 6.50 usec
TE 293.6 K
D1 1.00000000 sec
TD0 1

======== CHANNEL f1 ========
NUC1 1H
P1 14.00 usec
PL1 -1.40 dB
PL1W 16.22728539 W
SFO1 600.1337060 MHz
SI 32768
SF 600.1300160 MHz
WDW EM
SSB 0
LB 0.30 Hz
GB 0
PC 1.00

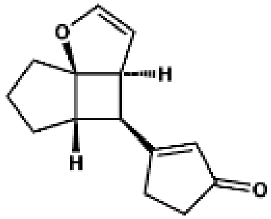

5: $C_{14}H_{16}O_2$
MW: 216.28

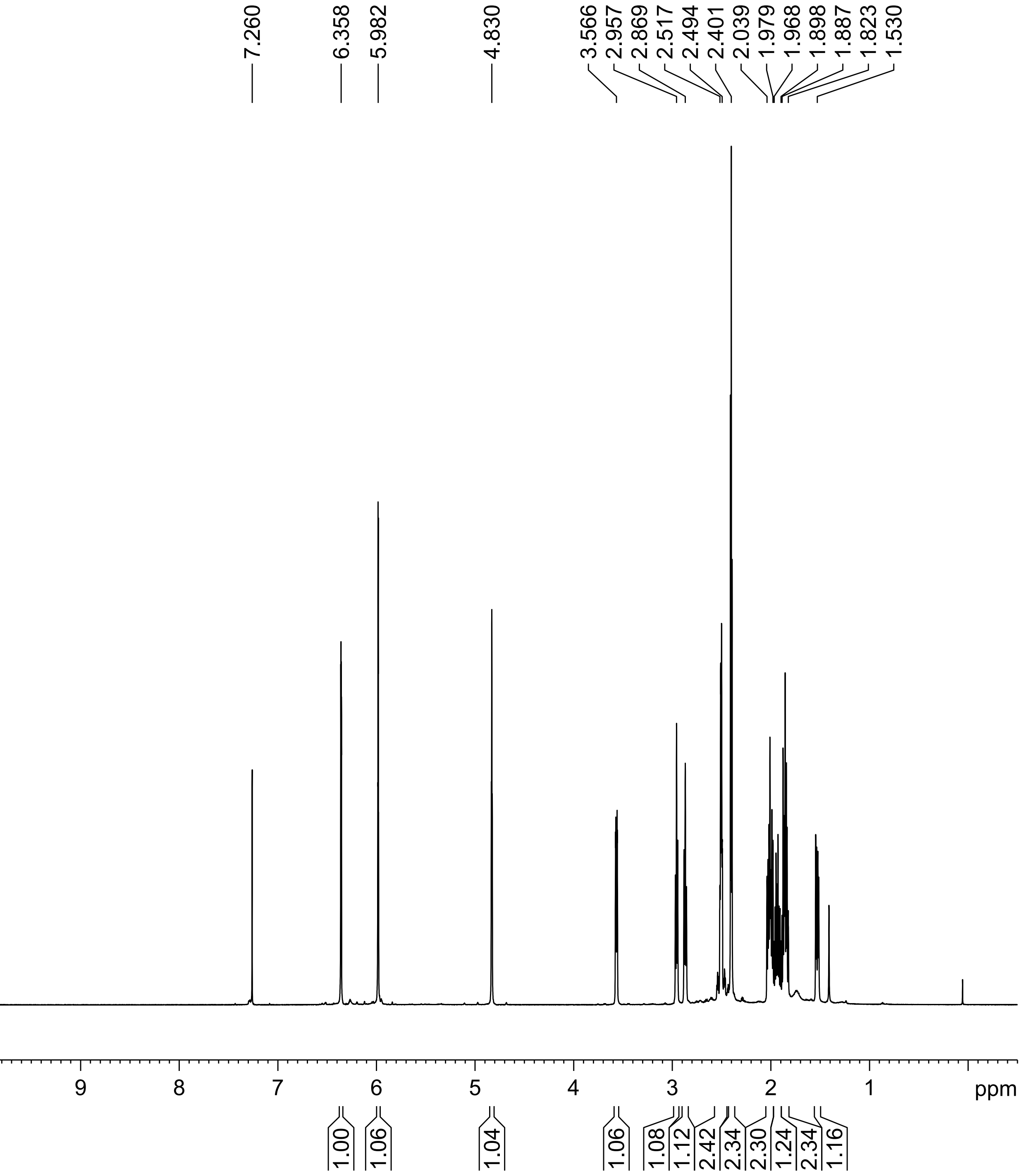

NAME AES 2181 C13
EXPNO 1
PROCNO 1
Date_ 20160511
Time 21.59
INSTRUM spect
PROBHD 5 mm PABBO BB-
PULPROG zgpg30
TD 65536
SOLVENT CDCl3
NS 4096
DS 4
SWH 36057.691 Hz
FIDRES 0.550197 Hz
AQ 0.9088159 sec
RG 203
DW 13.867 usec
DE 6.50 usec
TE 298.3 K
D1 2.00000000 sec
D11 0.03000000 sec
TD0 1

======== CHANNEL f1 ==
NUC1 13C
P1 9.50 usec
PL1 3.00 dB
PL1W 59.92485046 W
SFO1 150.9178988 MHz

======== CHANNEL f2 ========
CPDPRG2 waltz16
NUC2 1H
PCPD2 80.00 usec
PL2 -1.40 dB
PL12 13.74 dB
PL13 13.70 dB
PL2W 16.22728539 W
PL12W 0.49687356 W
PL13W 0.50147104 W
SFO2 600.1324005 MHz
SI 32768
SF 150.9028090 MHz
WDW EM
SSB 0
LB 1.00 Hz
GB 0
PC 1.40

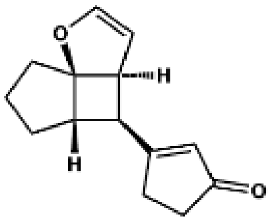

5: $C_{14}H_{16}O_2$
MW: 216.28

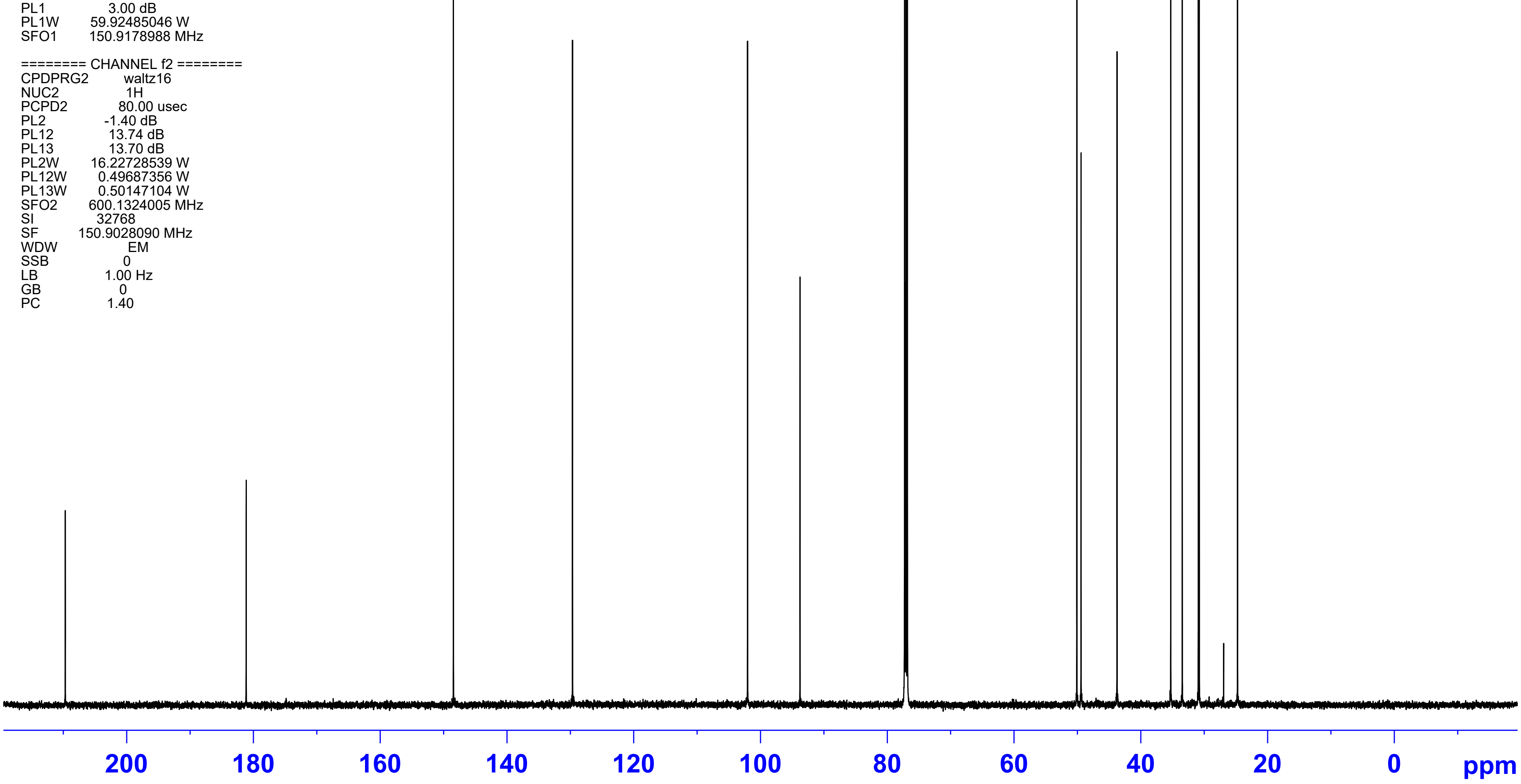

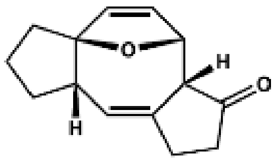

6: $C_{14}H_{16}O_2$
MW: 216.28

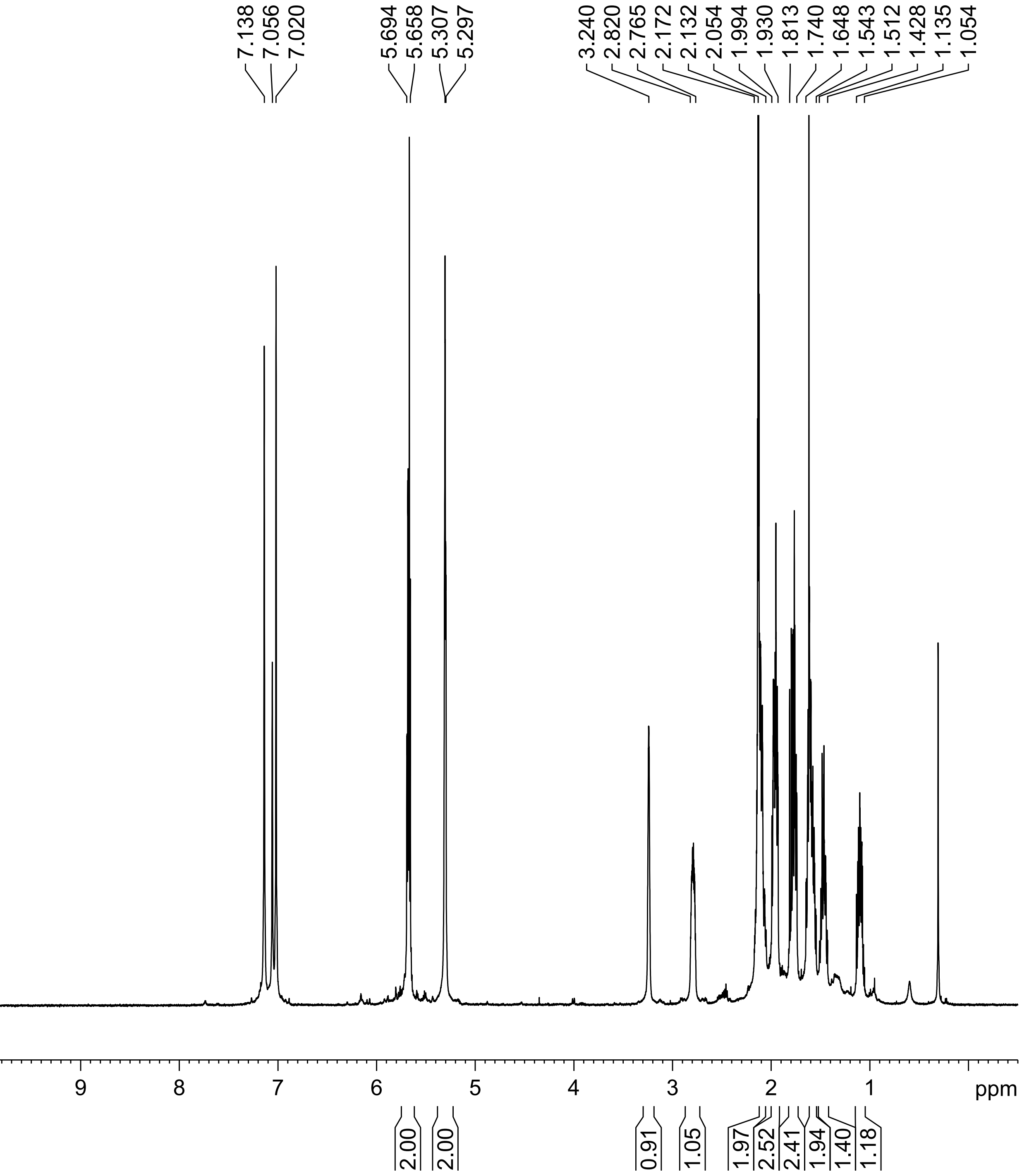

NAME AES 5186 Cope 1.5h 100C C13
EXPNO 1
PROCNO 1
Date_ 20180116
Time 21.11
INSTRUM spect
PROBHD 5 mm PABBO BB-
PULPROG zgpg30
TD 65536
SOLVENT Tol
NS 13990
DS 4
SWH 39062.500 Hz
FIDRES 0.596046 Hz
AQ 0.8389108 sec
RG 203
DW 12.800 usec
DE 6.50 usec
TE 298.3 K
D1 2.00000000 sec
D11 0.03000000 sec
TD0 1

======== CHANNEL f1 ========
NUC1 13C
P1 9.50 usec
PL1 3.00 dB
PL1W 59.92485046 W
SFO1 150.9178988 MHz

======== CHANNEL f2 ========
CPDPRG2 waltz16
NUC2 1H
PCPD2 80.00 usec
PL2 -1.40 dB
PL12 14.38 dB
PL13 13.70 dB
PL2W 16.22728539 W
PL12W 0.42879117 W
PL13W 0.50147104 W
SFO2 600.1324005 MHz
SI 32768
SF 150.9028090 MHz
WDW EM
SSB 0
LB 1.00 Hz
GB 0
PC 1.40

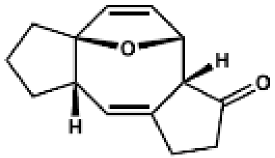

6: $C_{14}H_{16}O_2$
MW: 216.28

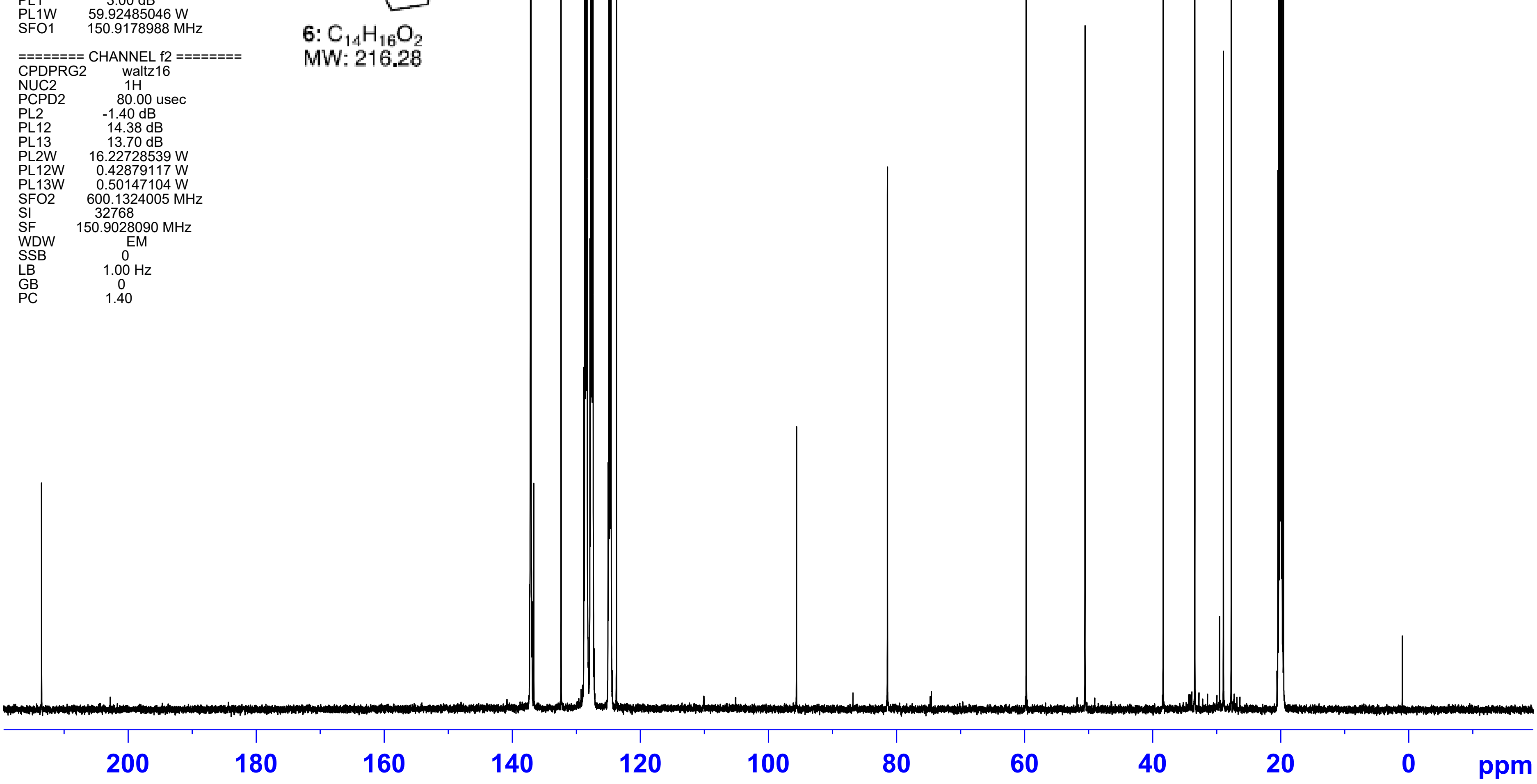

NAME AES 5050b
EXPNO 1
PROCNO 1
Date_ 20171030
Time 9.46
INSTRUM spect
PROBHD 5 mm PABBO BB-
PULPROG zg30
TD 65536
SOLVENT CDCl3
NS 8
DS 2
SWH 8223.685 Hz
FIDRES 0.125483 Hz
AQ 3.9846387 sec
RG 181
DW 60.800 usec
DE 6.50 usec
TE 293.9 K
D1 1.00000000 sec
TD0 1

======== CHANNEL f1 ========
NUC1 1H
P1 10.00 usec
PL1 -2.78 dB
PL1W 27.10892296 W
SFO1 400.1724712 MHz
SI 32768
SF 400.1700094 MHz
WDW EM
SSB 0
LB 0.30 Hz
GB 0
PC 1.00

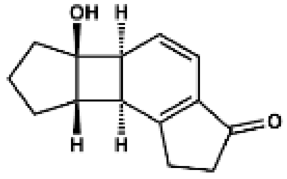

11: $C_{14}H_{16}O_2$
MW: 216.28

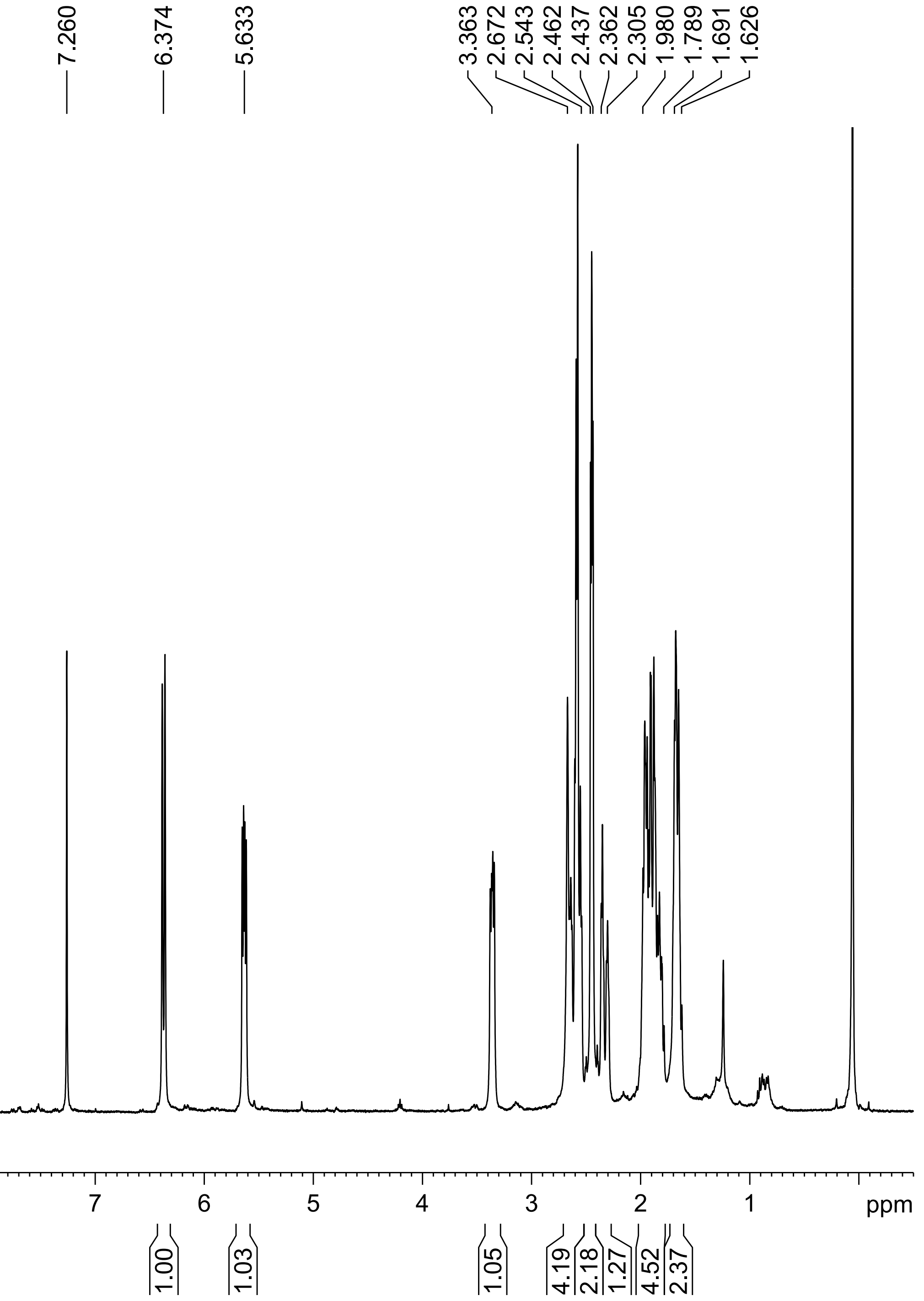

NAME AES 5052b C13
EXPNO 1
PROCNO 1
Date_ 20171031
Time 10.41
INSTRUM spect
PROBHD 5 mm PABBO BB-
PULPROG zgpg30
TD 65536
SOLVENT CDCl3
NS 636
DS 4
SWH 39062.500 Hz
FIDRES 0.596046 Hz
AQ 0.8389108 sec
RG 203
DW 12.800 usec
DE 6.50 usec
TE 297.9 K
D1 2.00000000 sec
D11 0.03000000 sec
TD0 1

======== CHANNEL f1 ========
NUC1 13C
P1 9.50 usec
PL1 3.00 dB
PL1W 59.92485046 W
SFO1 150.9178988 MHz

======== CHANNEL f2 ========
CPDPRG2 waltz16
NUC2 1H
PCPD2 80.00 usec
PL2 -1.40 dB
PL12 14.38 dB
PL13 13.70 dB
PL2W 16.22728539 W
PL12W 0.42879117 W
PL13W 0.50147104 W
SFO2 600.1324005 MHz
SI 32768
SF 150.9028090 MHz
WDW EM
SSB 0
LB 1.00 Hz
GB 0
PC 1.40

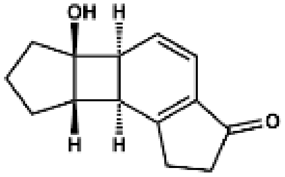

11: $C_{14}H_{16}O_2$
MW: 216.28

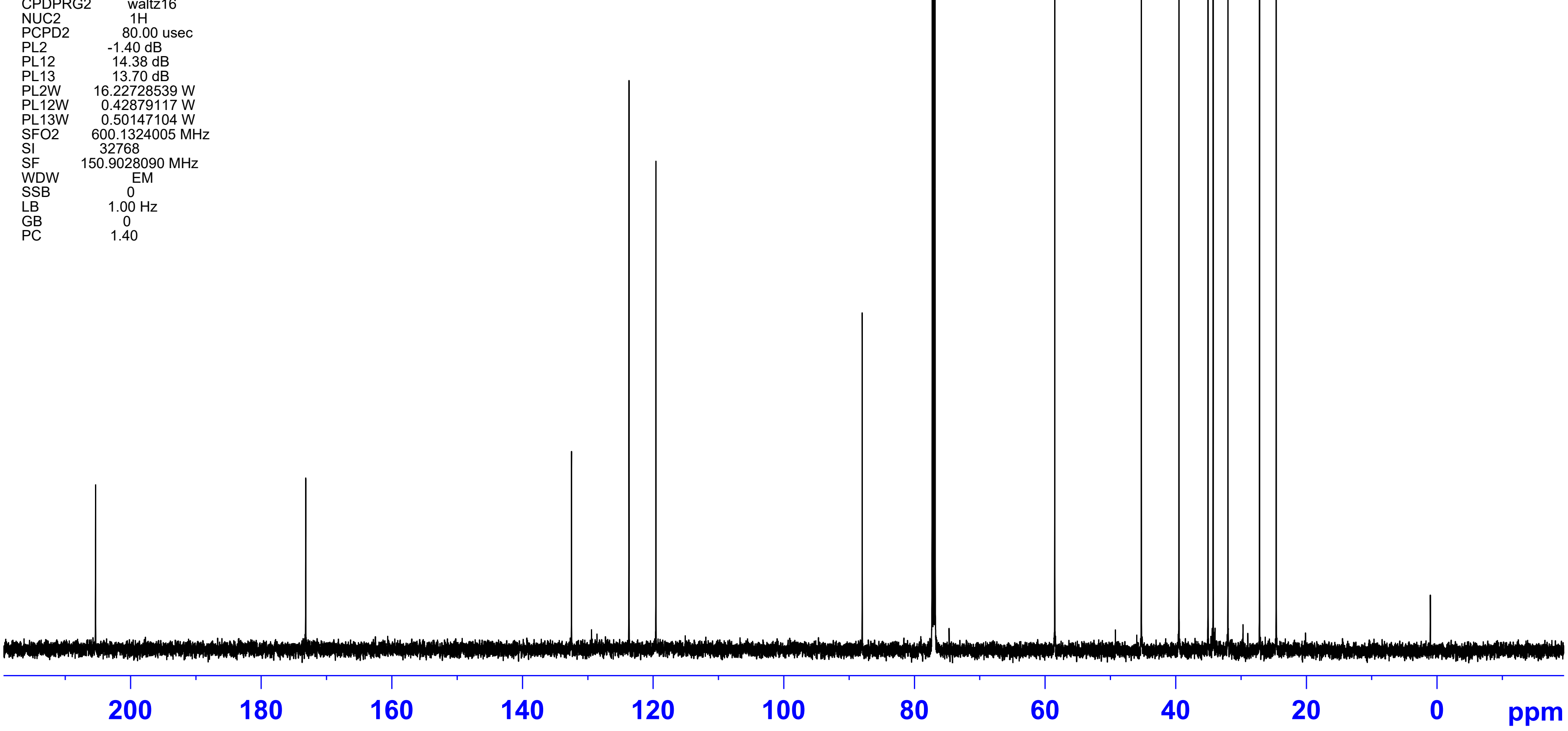

NAME AES 3017 1H
EXPNO 1
PROCNO 1
Date_ 20160824
Time 0.20
INSTRUM spect
PROBHD 5 mm PABBO BB-
PULPROG zg30
TD 65536
SOLVENT CDCl3
NS 16
DS 2
SWH 8223.685 Hz
FIDRES 0.125483 Hz
AQ 3.9846387 sec
RG 71.8
DW 60.800 usec
DE 6.50 usec
TE 297.9 K
D1 1.00000000 sec
TD0 1

======== CHANNEL f1 ========
NUC1 1H
P1 10.00 usec
PL1 -2.78 dB
PL1W 27.10892296 W
SFO1 400.1724712 MHz
SI 32768
SF 400.1700089 MHz
WDW EM
SSB 0
LB 0.30 Hz
GB 0
PC 1.00

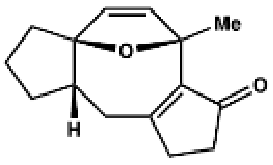

13a: $C_{15}H_{18}O_2$
MW: 230.31

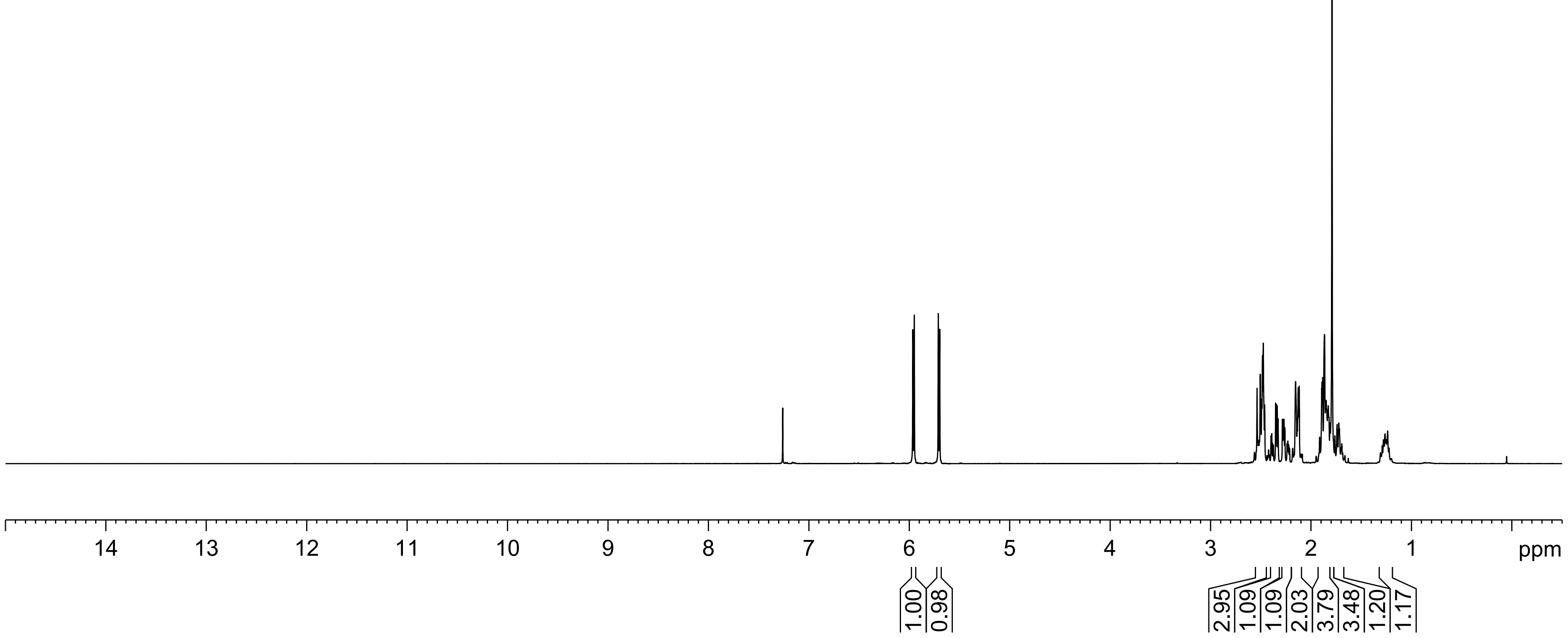

NAME AES 3017 C13
EXPNO 1
PROCNO 1
Date_ 20160824
Time 0.54
INSTRUM spect
PROBHD 5 mm PABBO BB-
PULPROG zgpg30
TD 65536
SOLVENT CDCl3
NS 512
DS 4
SWH 24038.461 Hz
FIDRES 0.366798 Hz
AQ 1.3631988 sec
RG 203
DW 20.800 usec
DE 6.50 usec
TE 298.1 K
D1 2.00000000 sec
D11 0.03000000 sec
TD0 1

======== CHANNEL f1 ========
NUC1 13C
P1 10.00 usec
PL1 -1.78 dB
PL1W 47.30532455 W
SFO1 100.6328888 MHz

======== CHANNEL f2 ========
CPDPRG2 waltz16
NUC2 1H
PCPD2 80.00 usec
PL2 -2.78 dB
PL12 15.28 dB
PL13 18.00 dB
PL2W 27.10892296 W
PL12W 0.42375252 W
PL13W 0.22652298 W
SFO2 400.1716007 MHz
SI 32768
SF 100.6228270 MHz
WDW EM
SSB 0
LB 1.00 Hz
GB 0
PC 1.40

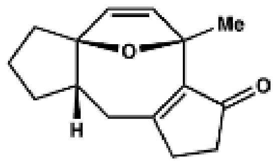

13a: $C_{15}H_{18}O_2$
MW: 230.31

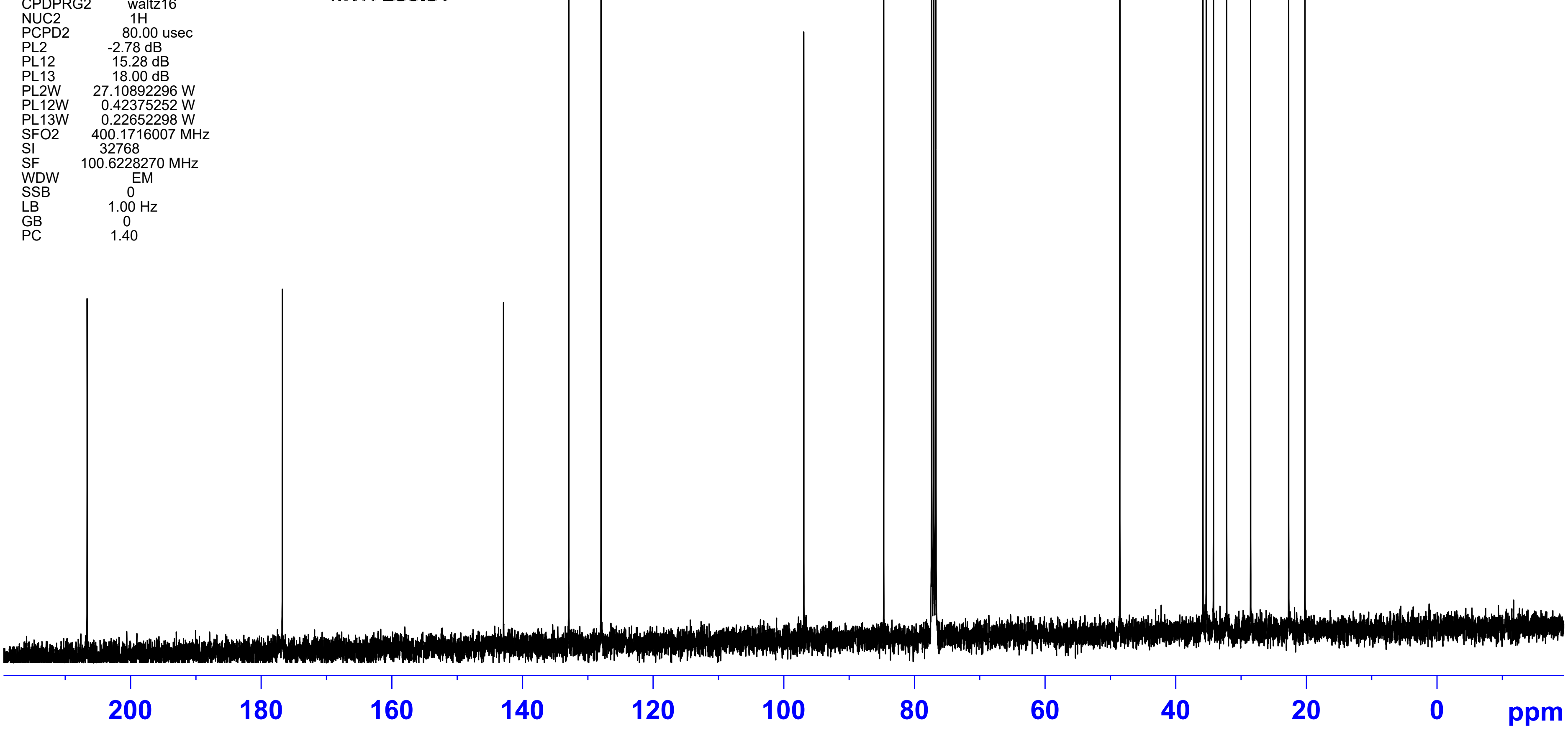

NAME AES 3293a
EXPNO 1
PROCNO 1
Date_ 20170407
Time 20.51
INSTRUM spect
PROBHD 5 mm PABBO BB-
PULPROG zg30
TD 65536
SOLVENT CDCl3
NS 8
DS 2
SWH 8223.685 Hz
FIDRES 0.125483 Hz
AQ 3.9846387 sec
RG 144
DW 60.800 usec
DE 6.50 usec
TE 292.9 K
D1 1.00000000 sec
TD0 1

======== CHANNEL f1 ========
NUC1 1H
P1 10.00 usec
PL1 -2.78 dB
PL1W 27.10892296 W
SFO1 400.1724712 MHz
SI 32768
SF 400.1700087 MHz
WDW EM
SSB 0
LB 0.30 Hz
GB 0
PC 1.00

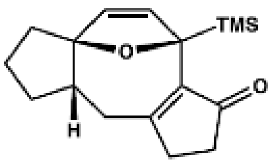

13b: $C_{17}H_{24}O_2Si$
MW: 288.46

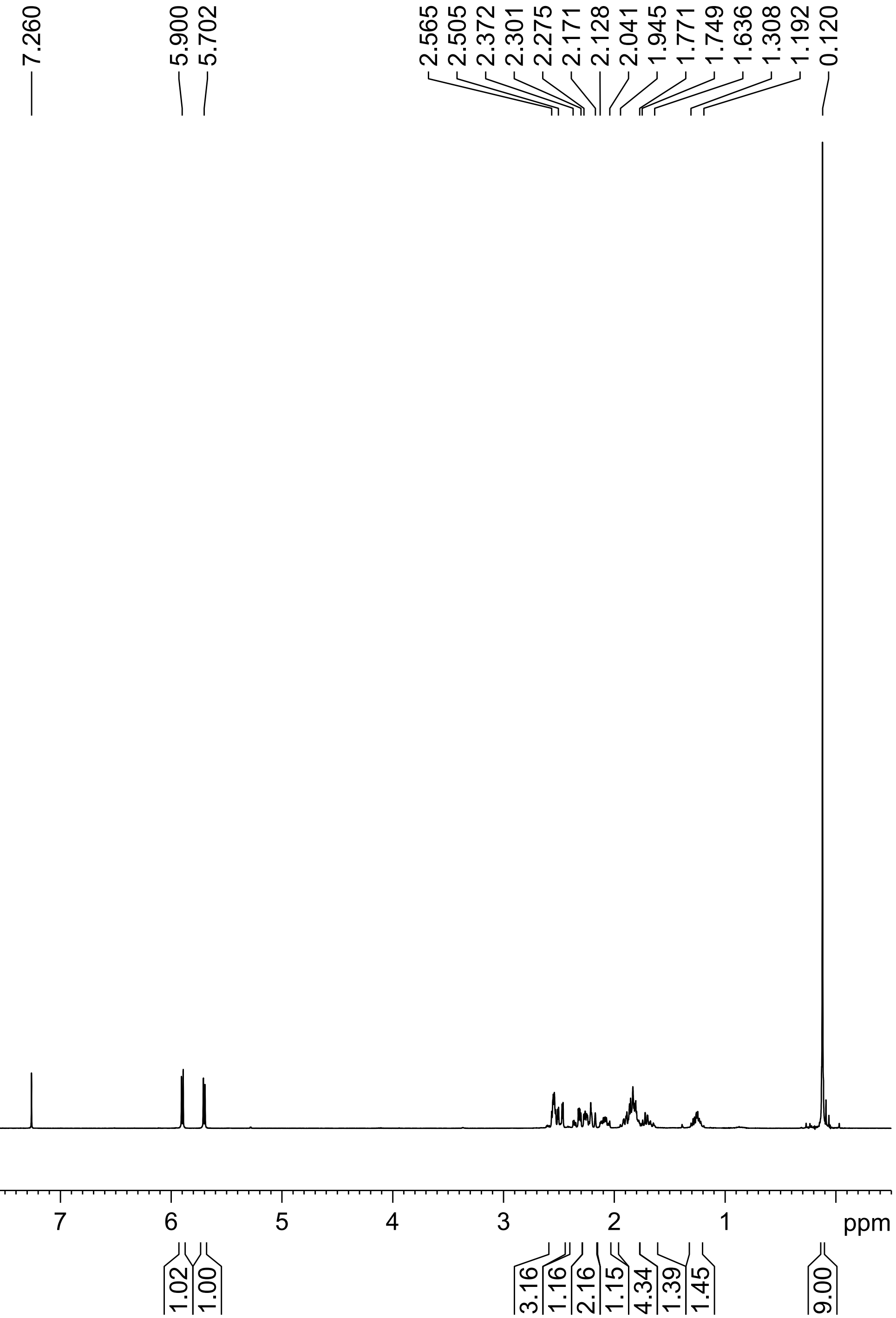

NAME AES 3293a C13
EXPNO 1
PROCNO 1
Date_ 20170407
Time 22.03
INSTRUM spect
PROBHD 5 mm PABBO BB-
PULPROG zgpg30
TD 65536
SOLVENT CDCl3
NS 256
DS 4
SWH 24038.461 Hz
FIDRES 0.366798 Hz
AQ 1.3631988 sec
RG 203
DW 20.800 usec
DE 6.50 usec
TE 294.0 K
D1 2.00000000 sec
D11 0.03000000 sec
TD0 1

======== CHANNEL f1 ========
NUC1 13C
P1 10.00 usec
PL1 -1.78 dB
PL1W 47.30532455 W
SFO1 100.6328888 MHz

======== CHANNEL f2 ========
CPDPRG2 waltz16
NUC2 1H
PCPD2 80.00 usec
PL2 -2.78 dB
PL12 15.28 dB
PL13 18.00 dB
PL2W 27.10892296 W
PL12W 0.42375252 W
PL13W 0.22652298 W
SFO2 400.1716007 MHz
SI 32768
SF 100.6228270 MHz
WDW EM
SSB 0
LB 1.00 Hz
GB 0
PC 1.40

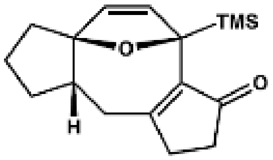

13b: $C_{17}H_{24}O_2Si$
MW: 288.46

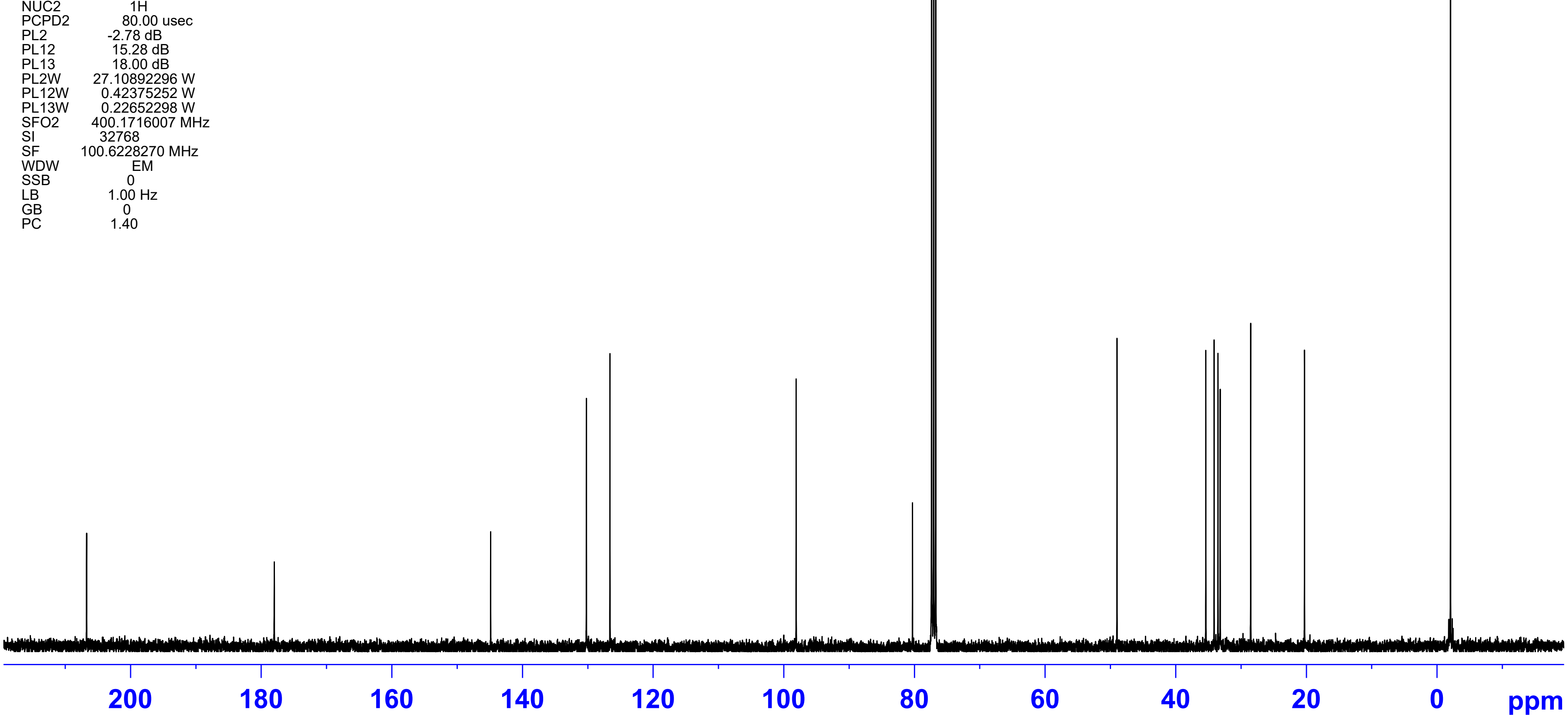

NAME AES 3300b 1H
EXPNO 1
PROCNO 1
Date_ 20170412
Time 22.50
INSTRUM spect
PROBHD 5 mm PABBO BB-
PULPROG zg30
TD 65536
SOLVENT CDCl3
NS 3
DS 2
SWH 8223.685 Hz
FIDRES 0.125483 Hz
AQ 3.9846387 sec
RG 114
DW 60.800 usec
DE 6.50 usec
TE 293.3 K
D1 1.00000000 sec
TD0 1

======== CHANNEL f1 ========
NUC1 1H
P1 10.00 usec
PL1 -2.78 dB
PL1W 27.10892296 W
SFO1 400.1724712 MHz
SI 32768
SF 400.1700091 MHz
WDW EM
SSB 0
LB 0.30 Hz
GB 0
PC 1.00

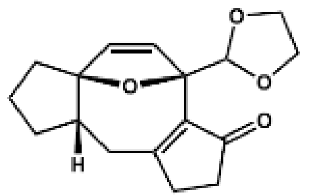

13c: $C_{17}H_{20}O_4$
MW: 288.34

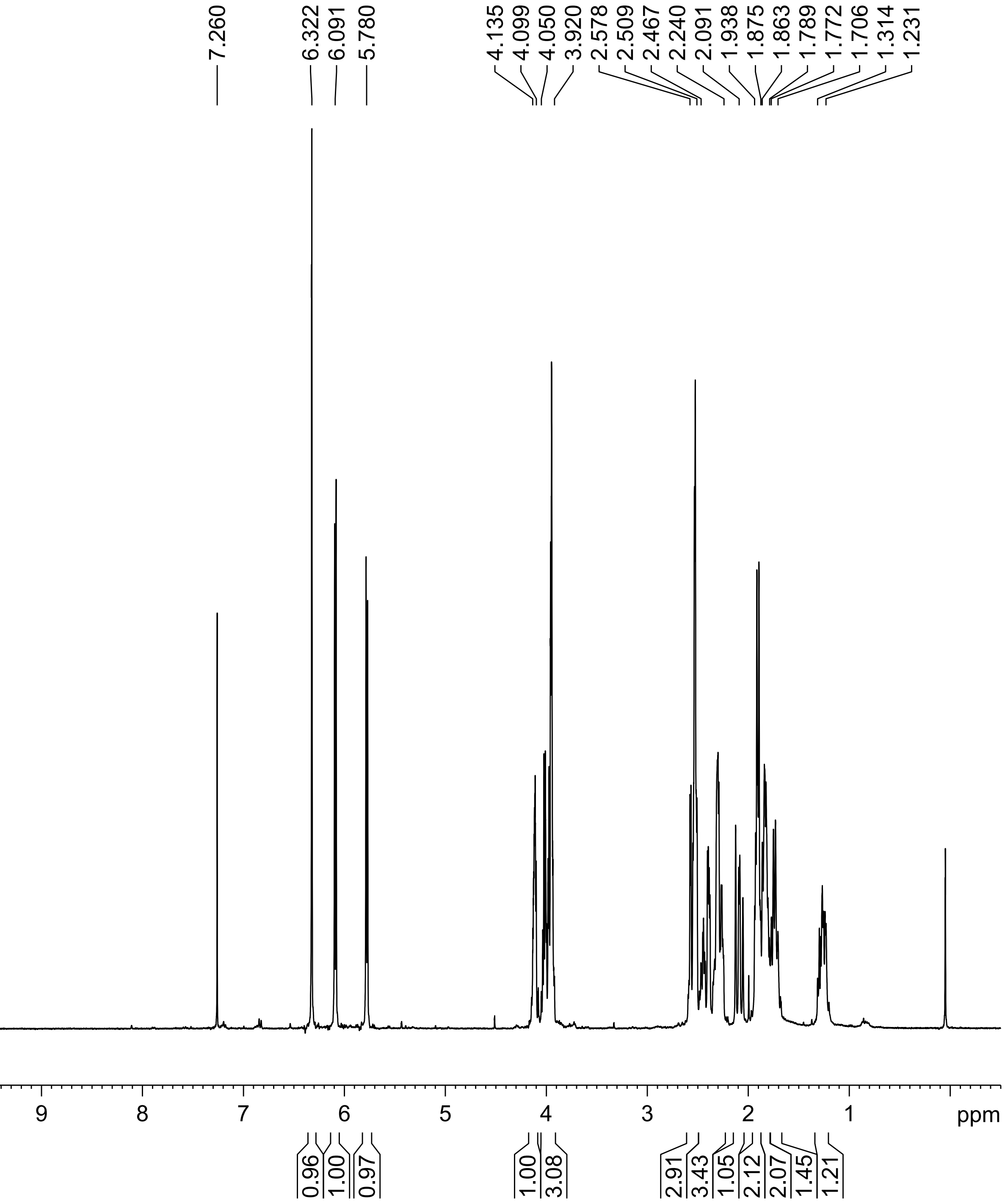

NAME AES 3300b C13
EXPNO 2
PROCNO 1
Date_ 20170414
Time 23.04
INSTRUM spect
PROBHD 5 mm PABBO BB-
PULPROG zgpg30
TD 65536
SOLVENT CDCl3
NS 1024
DS 4
SWH 36057.691 Hz
FIDRES 0.550197 Hz
AQ 0.9088159 sec
RG 203
DW 13.867 usec
DE 6.50 usec
TE 298.0 K
D1 2.00000000 sec
D11 0.03000000 sec
TD0 1

======== CHANNEL f1 ========
NUC1 13C
P1 9.50 usec
PL1 3.00 dB
PL1W 59.92485046 W
SFO1 150.9178988 MHz

======== CHANNEL f2 ========
CPDPRG2 waltz16
NUC2 1H
PCPD2 80.00 usec
PL2 -1.40 dB
PL12 13.74 dB
PL13 13.70 dB
PL2W 16.22728539 W
PL12W 0.49687356 W
PL13W 0.50147104 W
SFO2 600.1324005 MHz
SI 32768
SF 150.9028090 MHz
WDW EM
SSB 0
LB 1.00 Hz
GB 0
PC 1.40

13c: $C_{17}H_{20}O_4$
MW: 288.34

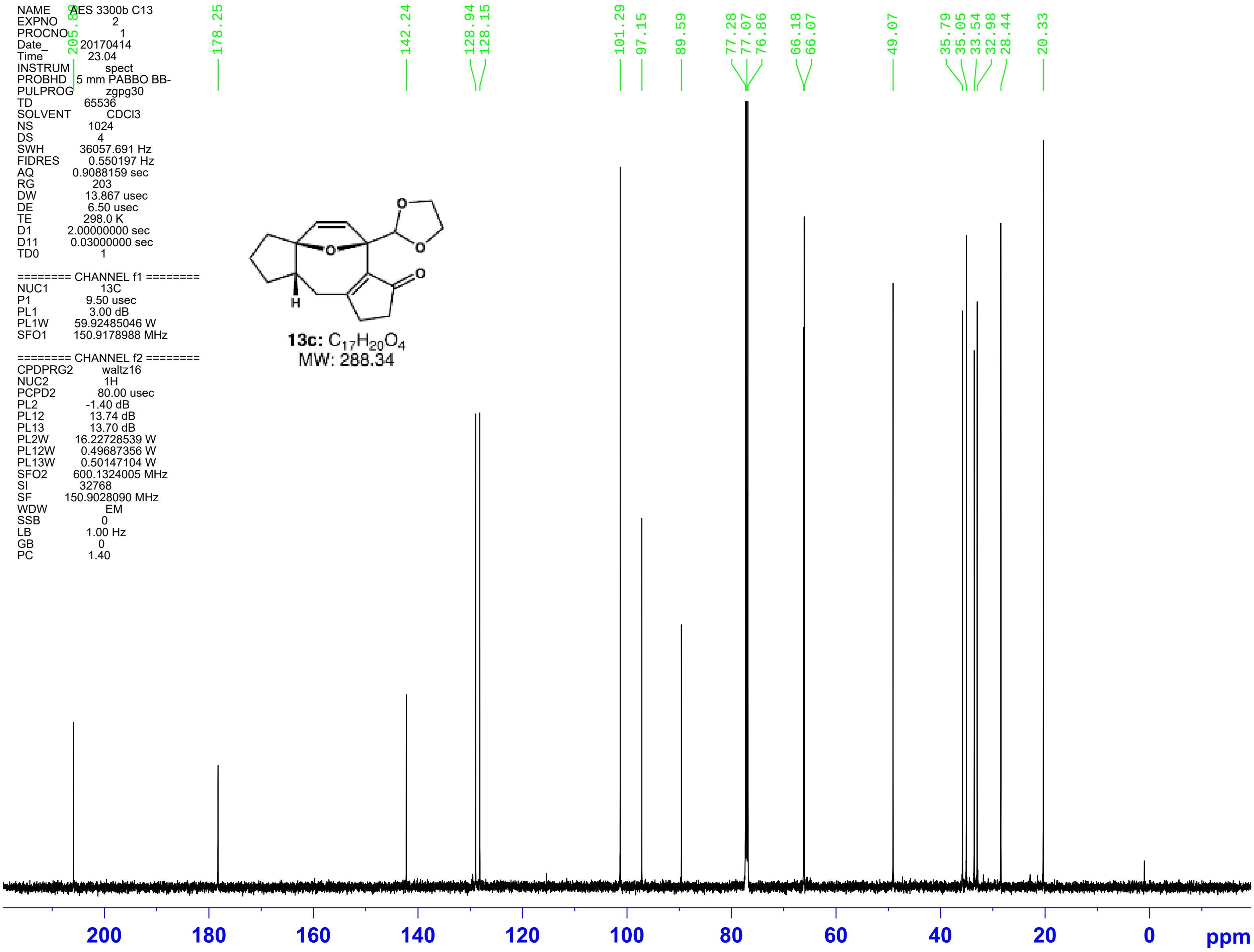

NAME AES 3007 1H
EXPNO 1
PROCNO 1
Date_ 20160816
Time 1.33
INSTRUM spect
PROBHD 5 mm PABBO BB-
PULPROG zg30
TD 65536
SOLVENT CDCl3
NS 16
DS 2
SWH 8223.685 Hz
FIDRES 0.125483 Hz
AQ 3.9846387 sec
RG 203
DW 60.800 usec
DE 6.50 usec
TE 295.3 K
D1 1.00000000 sec
TD0 1

======== CHANNEL f1 ========
NUC1 1H
P1 10.00 usec
PL1 -2.78 dB
PL1W 27.10892296 W
SFO1 400.1724712 MHz
SI 32768
SF 400.1700091 MHz
WDW EM
SSB 0
LB 0.30 Hz
GB 0
PC 1.00

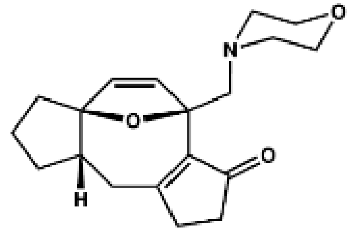

13d: $C_{19}H_{25}NO_3$
MW: 315.41

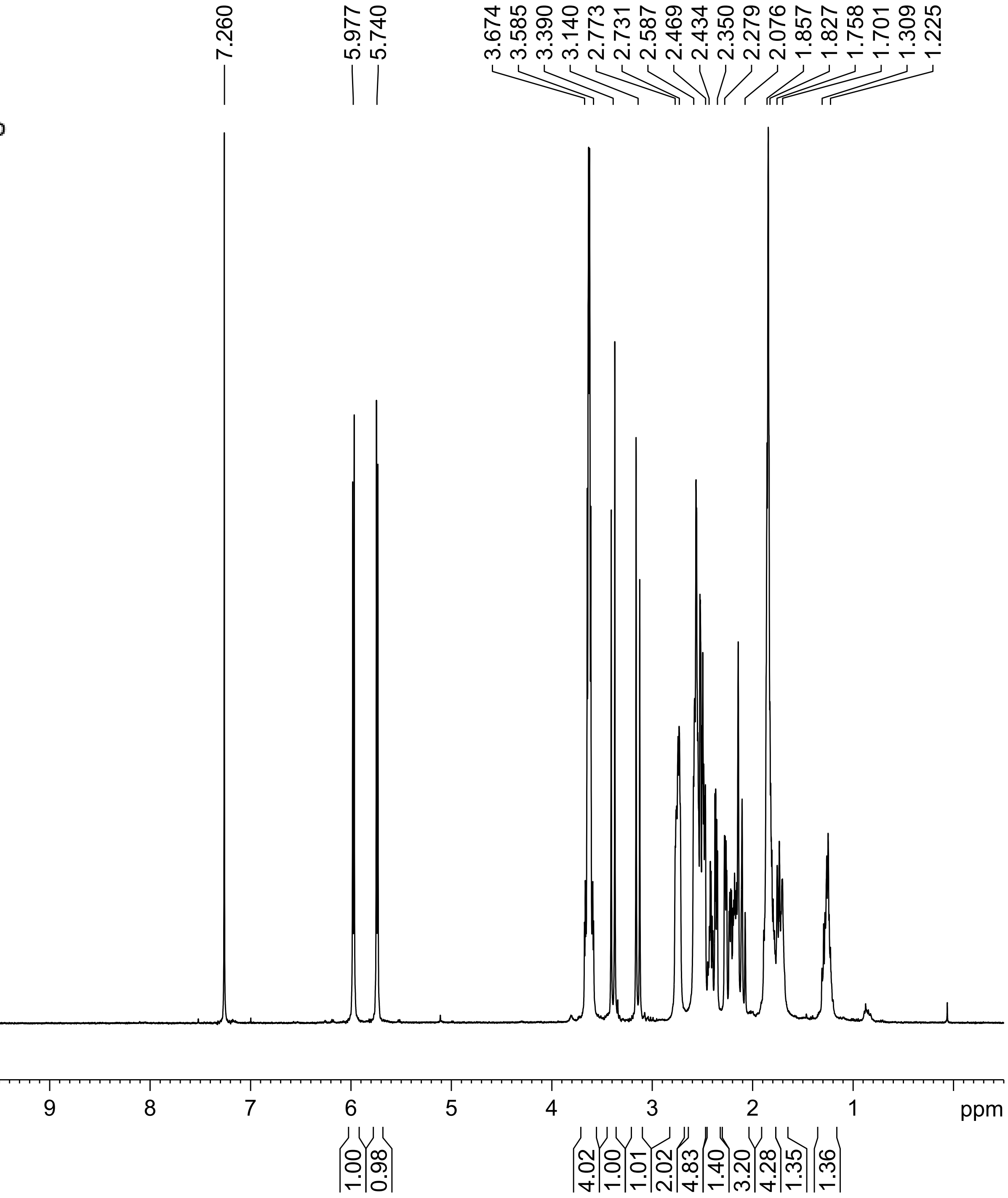

NAME AES 3007 C13
EXPNO 1
PROCNO 1
Date_ 20160816
Time 2.01
INSTRUM spect
PROBHD 5 mm PABBO BB-
PULPROG zgpg30
TD 65536
SOLVENT CDCl3
NS 256
DS 4
SWH 24038.461 Hz
FIDRES 0.366798 Hz
AQ 1.3631988 sec
RG 203
DW 20.800 usec
DE 6.50 usec
TE 296.4 K
D1 2.00000000 sec
D11 0.03000000 sec
TD0 1

======== CHANNEL f1 ========
NUC1 13C
P1 10.00 usec
PL1 -1.78 dB
PL1W 47.30532455 W
SFO1 100.6328888 MHz

======== CHANNEL f2 ========
CPDPRG2 waltz16
NUC2 1H
PCPD2 80.00 usec
PL2 -2.78 dB
PL12 15.28 dB
PL13 18.00 dB
PL2W 27.10892296 W
PL12W 0.42375252 W
PL13W 0.22652298 W
SFO2 400.1716007 MHz
SI 32768
SF 100.6228270 MHz
WDW EM
SSB 0
LB 1.00 Hz
GB 0
PC 1.40

13d: $C_{19}H_{25}NO_3$
MW: 315.41

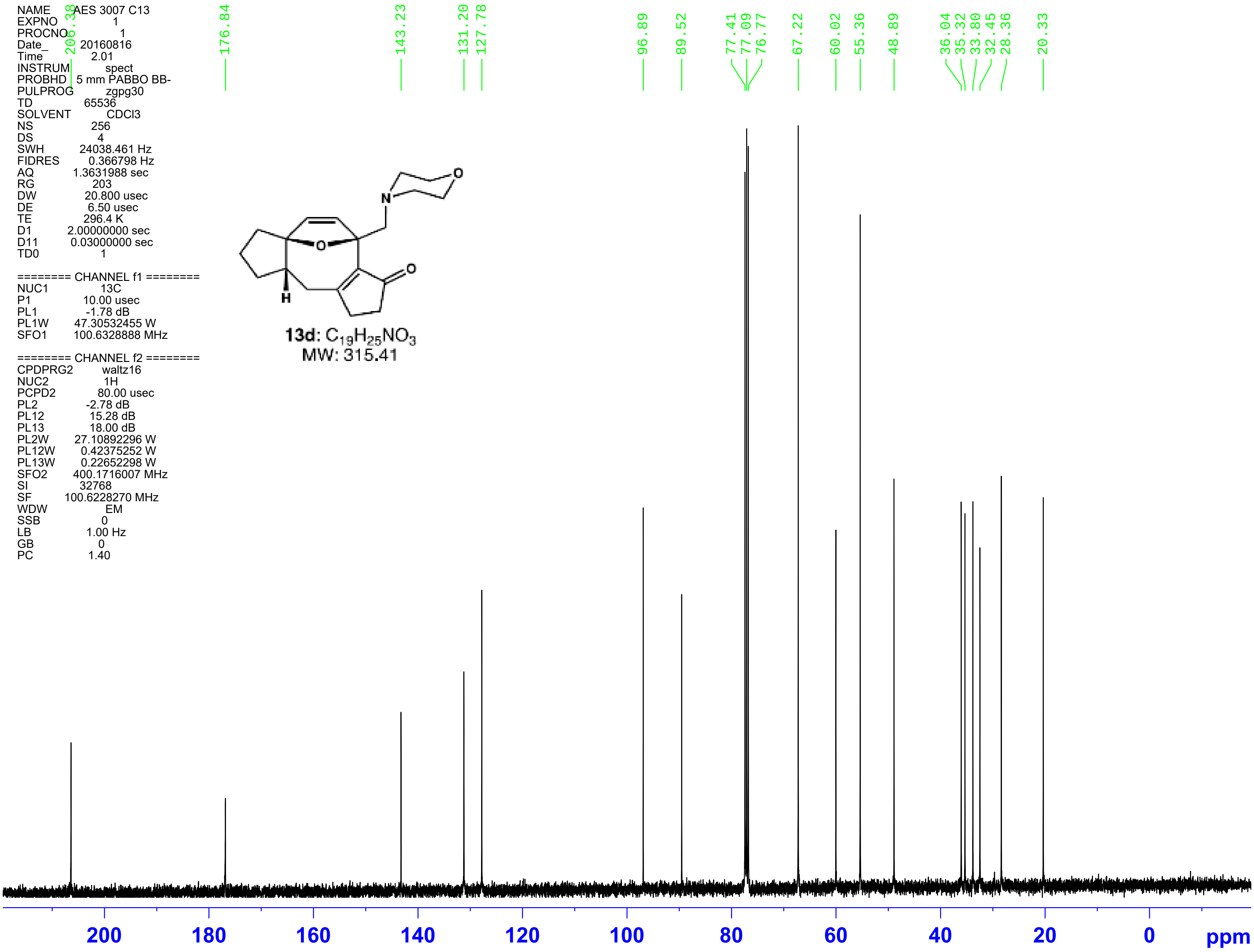

NAME AES 4003b
EXPNO 1
PROCNO 1
Date_ 20170413
Time 17.11
INSTRUM spect
PROBHD 5 mm PABBO BB-
PULPROG zg30
TD 65536
SOLVENT CDCl3
NS 8
DS 2
SWH 8223.685 Hz
FIDRES 0.125483 Hz
AQ 3.9846387 sec
RG 64
DW 60.800 usec
DE 6.50 usec
TE 293.3 K
D1 1.00000000 sec
TD0 1

======== CHANNEL f1 ========
NUC1 1H
P1 10.00 usec
PL1 -2.78 dB
PL1W 27.10892296 W
SFO1 400.1724712 MHz
SI 32768
SF 400.1700093 MHz
WDW EM
SSB 0
LB 0.30 Hz
GB 0
PC 1.00

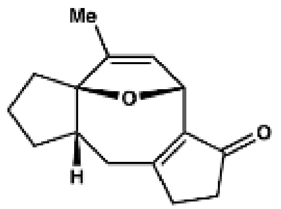

13e: $C_{15}H_{18}O_2$
MW: 230.31

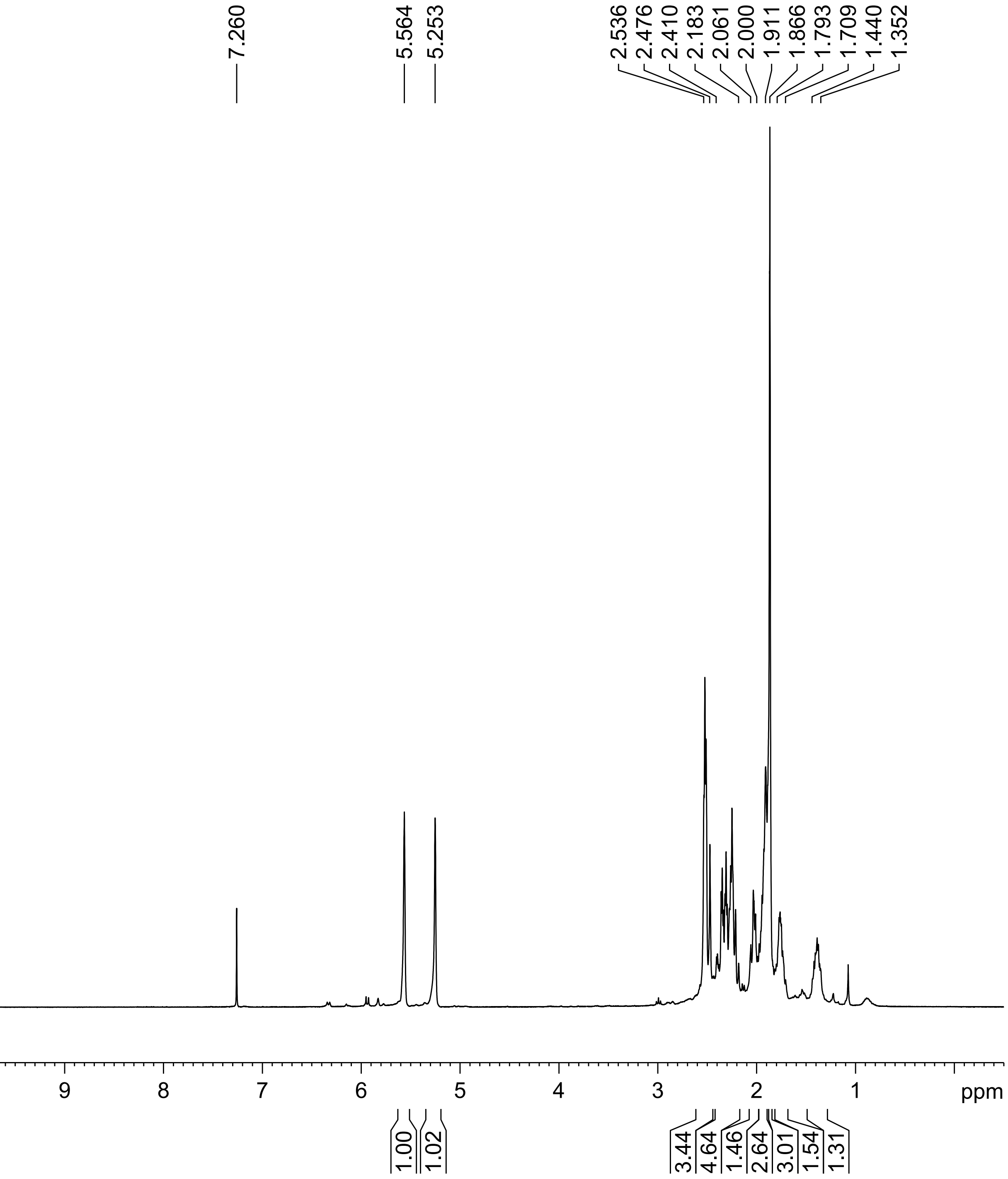

NAME AES 4003b C13
EXPNO 1
PROCNO 1
Date_ 20170414
Time 1.03
INSTRUM spect
PROBHD 5 mm PABBO BB-
PULPROG zgpg30
TD 65536
SOLVENT CDCl3
NS 256
DS 4
SWH 24038.461 Hz
FIDRES 0.366798 Hz
AQ 1.3631988 sec
RG 203
DW 20.800 usec
DE 6.50 usec
TE 294.7 K
D1 2.00000000 sec
D11 0.03000000 sec
TD0 1

======== CHANNEL f1 ========
NUC1 13C
P1 10.00 usec
PL1 -1.78 dB
PL1W 47.30532455 W
SFO1 100.6328888 MHz

======== CHANNEL f2 ========
CPDPRG2 waltz16
NUC2 1H
PCPD2 80.00 usec
PL2 -2.78 dB
PL12 15.28 dB
PL13 18.00 dB
PL2W 27.10892296 W
PL12W 0.42375252 W
PL13W 0.22652298 W
SFO2 400.1716007 MHz
SI 32768
SF 100.6228270 MHz
WDW EM
SSB 0
LB 1.00 Hz
GB 0
PC 1.40

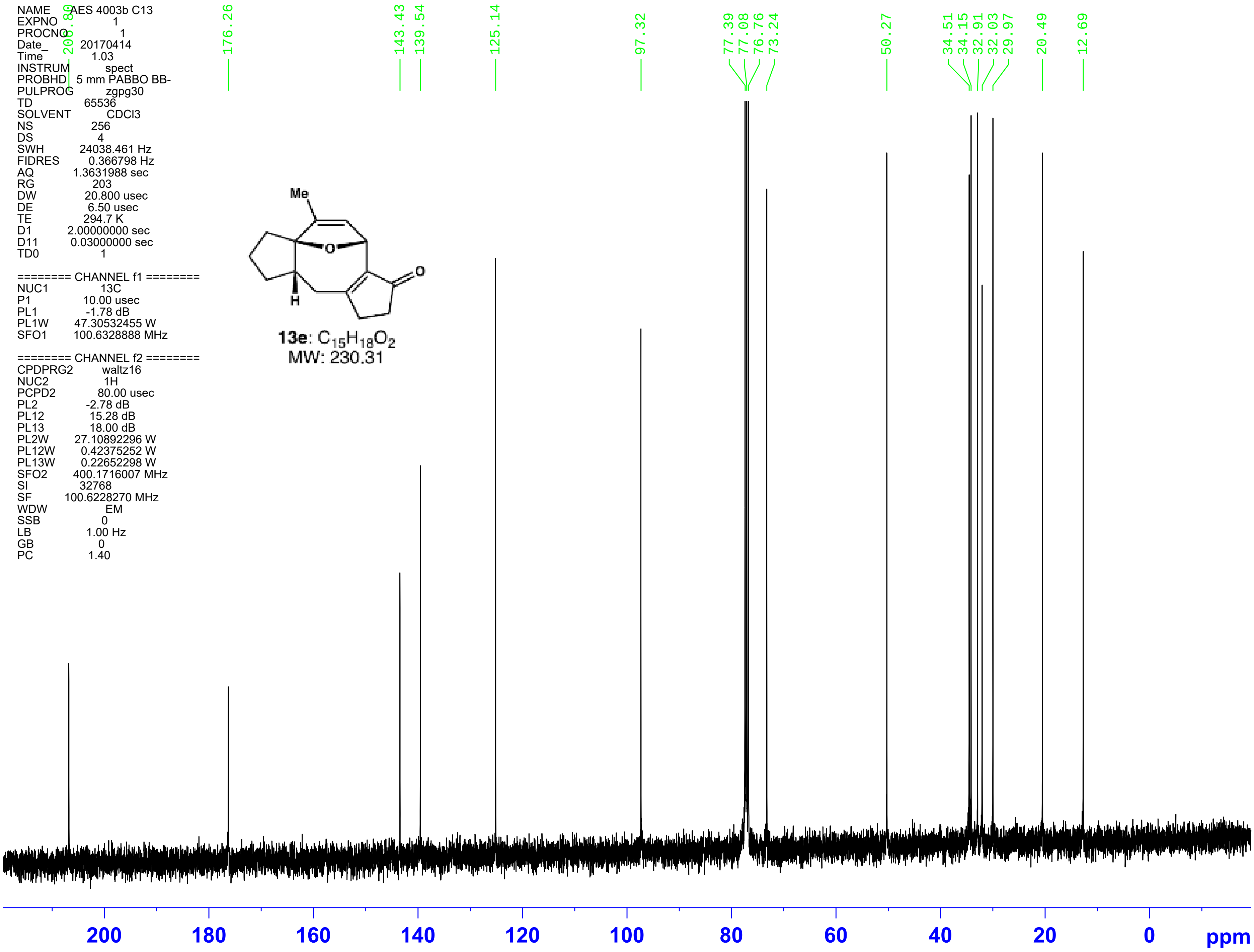

13e: $C_{15}H_{18}O_2$
MW: 230.31

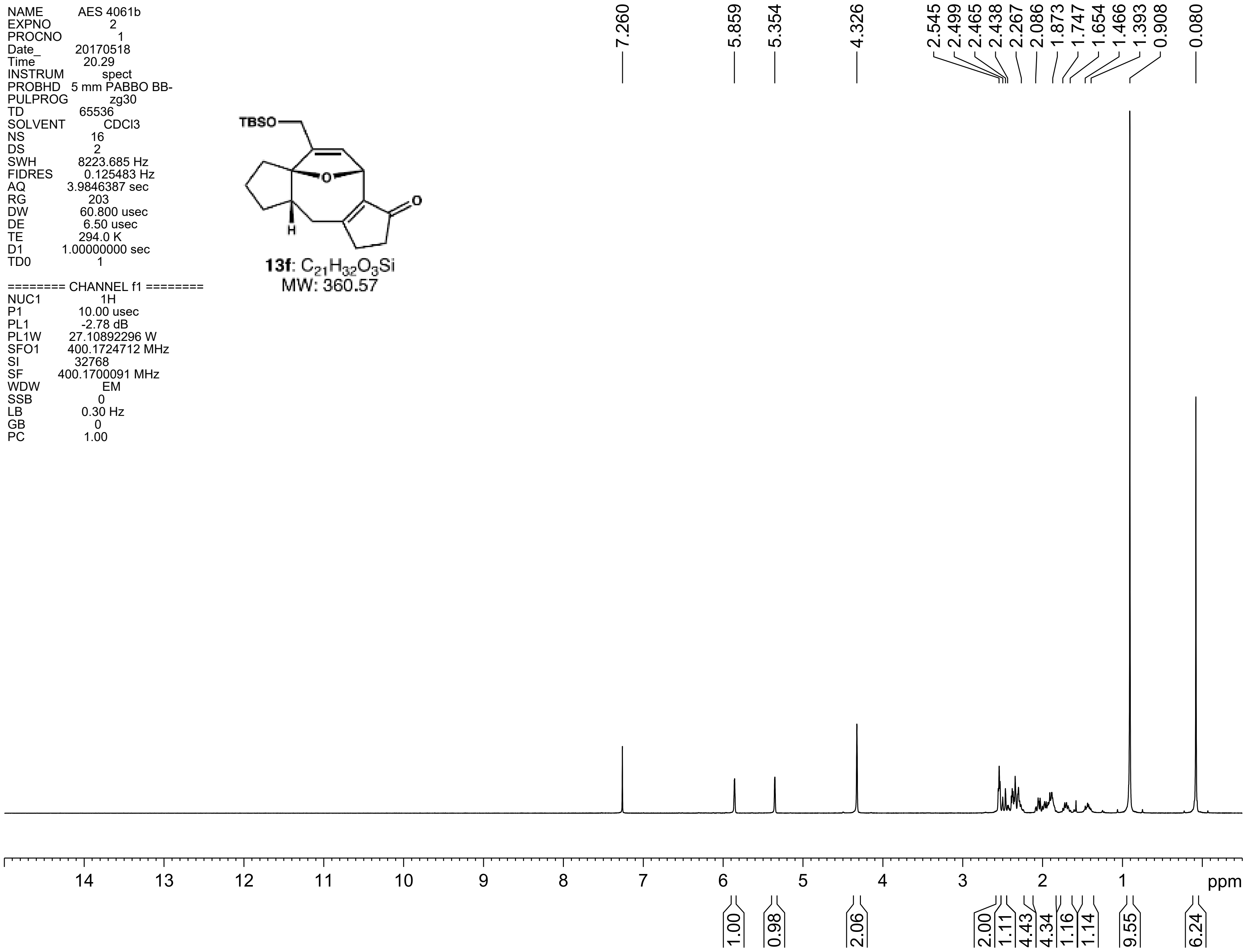
NAME AES 4061b
EXPNO 2
PROCNO 1
Date_ 20170518
Time 20.29
INSTRUM spect
PROBHD 5 mm PABBO BB-
PULPROG zg30
TD 65536
SOLVENT CDCl3
NS 16
DS 2
SWH 8223.685 Hz
FIDRES 0.125483 Hz
AQ 3.9846387 sec
RG 203
DW 60.800 usec
DE 6.50 usec
TE 294.0 K
D1 1.00000000 sec
TD0 1
======== CHANNEL f1 ========
NUC1 1H
P1 10.00 usec
PL1 -2.78 dB
PL1W 27.10892296 W
SFO1 400.1724712 MHz
SI 32768
SF 400.1700091 MHz
WDW EM
SSB 0
LB 0.30 Hz
GB 0
PC 1.00
TBSO
O
H
13f: $C_{21}H_{32}O_3Si$
MW: 360.57
7.260
5.859
5.354
4.326
2.545
2.499
2.465
2.438
2.267
2.086
1.873
1.747
1.654
1.466
1.393
0.908
0.080
14
13
12
11
10
9
8
7
6
5
4
3
2
1
ppm
1.00
0.98
2.06
2.00
1.11
4.43
4.34
1.16
1.14
9.55
6.24

NAME AES 4061b C13
EXPNO 1
PROCNO 1
Date_ 20170519
Time 0.46
INSTRUM spect
PROBHD 5 mm PABBO BB-
PULPROG zgpg30
TD 65536
SOLVENT CDCl3
NS 256
DS 4
SWH 24038.461 Hz
FIDRES 0.366798 Hz
AQ 1.3631988 sec
RG 203
DW 20.800 usec
DE 6.50 usec
TE 294.2 K
D1 2.00000000 sec
D11 0.03000000 sec
TD0 1

======== CHANNEL f1 ========
NUC1 13C
P1 10.00 usec
PL1 -1.78 dB
PL1W 47.30532455 W
SFO1 100.6328888 MHz

======== CHANNEL f2 ========
CPDPRG2 waltz16
NUC2 1H
PCPD2 80.00 usec
PL2 -2.78 dB
PL12 15.28 dB
PL13 18.00 dB
PL2W 27.10892296 W
PL12W 0.42375252 W
PL13W 0.22652298 W
SFO2 400.1716007 MHz
SI 32768
SF 100.6228270 MHz
WDW EM
SSB 0
LB 1.00 Hz
GB 0
PC 1.40

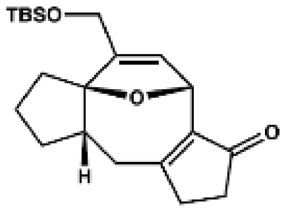

13f: $C_{21}H_{32}O_3Si$
MW: 360.57

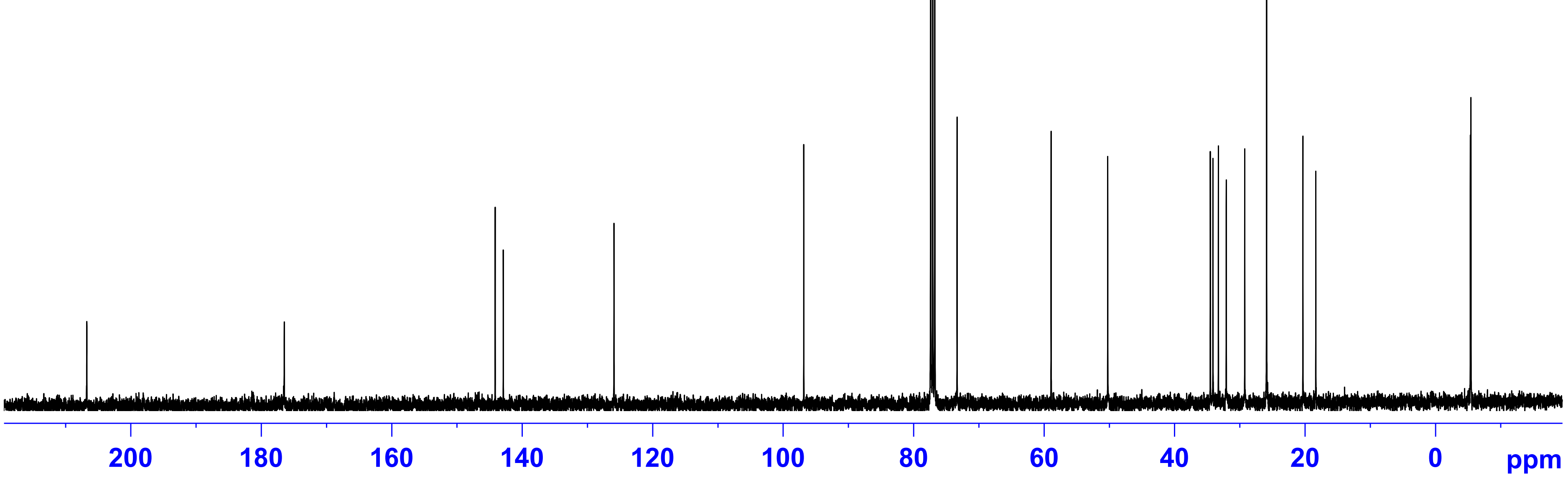

NAME AES 5108 [2+2] 20h then 1h 80 C et3N
EXPNO 1
PROCNO 1
Date_ 20171129
Time 19.05
INSTRUM spect
PROBHD 5 mm PABBO BB-
PULPROG zg30
TD 65536
SOLVENT CDCl3
NS 16
DS 2
SWH 8223.685 Hz
FIDRES 0.125483 Hz
AQ 3.9846387 sec
RG 203
DW 60.800 usec
DE 6.50 usec
TE 294.9 K
D1 1.00000000 sec
TD0 1

======== CHANNEL f1 ========
NUC1 1H
P1 10.00 usec
PL1 -2.78 dB
PL1W 27.10892296 W
SFO1 400.1724712 MHz
SI 32768
SF 400.1700090 MHz
WDW EM
SSB 0
LB 0.30 Hz
GB 0
PC 1.00

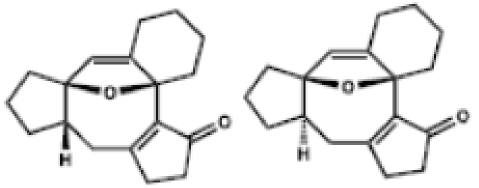

13g: $C_{18}H_{22}O_2$ MW: 270.37 **S14**: $C_{18}H_{22}O_2$ MW: 270.37

d.r = 10.1:1 (unpurified)

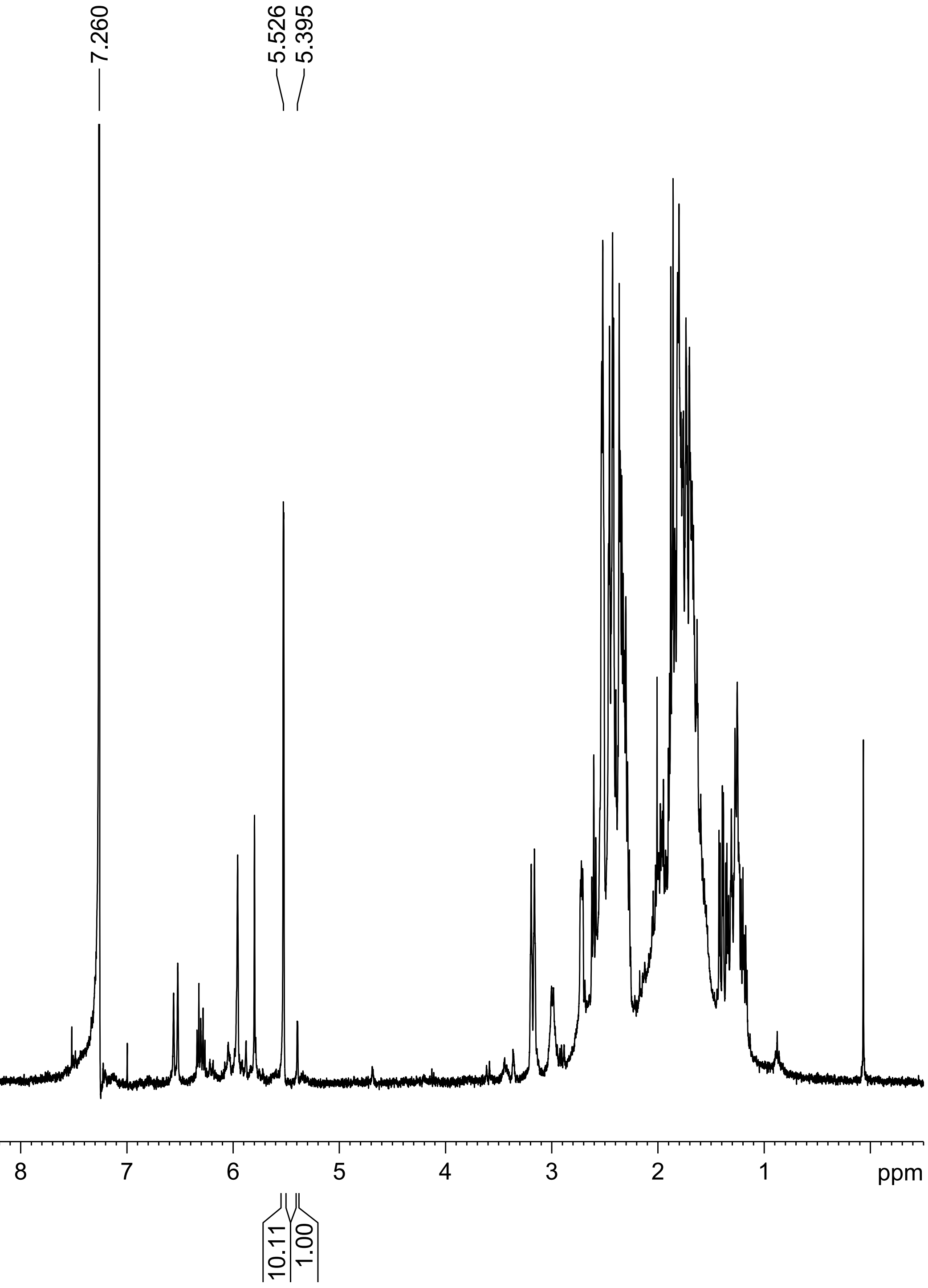

NAME AES 5108c
EXPNO 1
PROCNO 1
Date_ 20171130
Time 12.53
INSTRUM spect
PROBHD 5 mm PABBO BB-
PULPROG zg30
TD 65536
SOLVENT CDCl3
NS 8
DS 2
SWH 8223.685 Hz
FIDRES 0.125483 Hz
AQ 3.9846387 sec
RG 203
DW 60.800 usec
DE 6.50 usec
TE 294.7 K
D1 1.00000000 sec
TD0 1

======== CHANNEL f1 ========
NUC1 1H
P1 10.00 usec
PL1 -2.78 dB
PL1W 27.10892296 W
SFO1 400.1724712 MHz
SI 32768
SF 400.1700091 MHz
WDW EM
SSB 0
LB 0.30 Hz
GB 0
PC 1.00

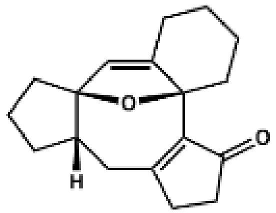

13g: $C_{18}H_{22}O_2$
MW: 270.37

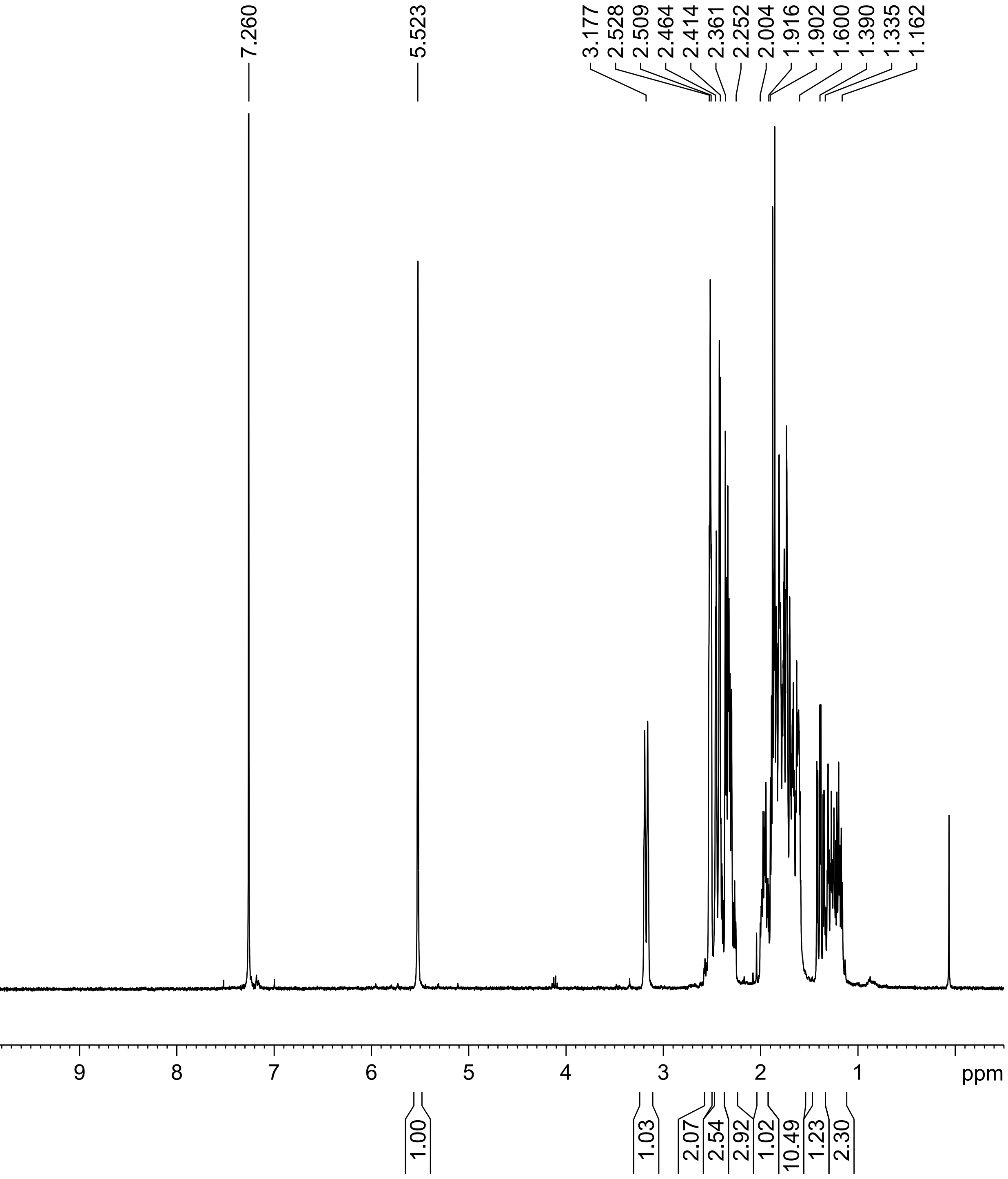

NAME AES 4084c C13
EXPNO 1
PROCNO 1
Date_ 20170607
Time 23.00
INSTRUM spect
PROBHD 5 mm PABBO BB-
PULPROG zgpg30
TD 65536
SOLVENT CDCl3
NS 256
DS 4
SWH 24038.461 Hz
FIDRES 0.366798 Hz
AQ 1.3631988 sec
RG 203
DW 20.800 usec
DE 6.50 usec
TE 296.5 K
D1 2.00000000 sec
D11 0.03000000 sec
TD0 1

======== CHANNEL f1 ========
NUC1 13C
P1 10.00 usec
PL1 -1.78 dB
PL1W 47.30532455 W
SFO1 100.6328888 MHz

======== CHANNEL f2 ========
CPDPRG2 waltz16
NUC2 1H
PCPD2 80.00 usec
PL2 -2.78 dB
PL12 15.28 dB
PL13 18.00 dB
PL2W 27.10892296 W
PL12W 0.42375252 W
PL13W 0.22652298 W
SFO2 400.1716007 MHz
SI 32768
SF 100.6228270 MHz
WDW EM
SSB 0
LB 1.00 Hz
GB 0
PC 1.40

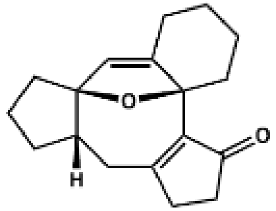

13g: $C_{18}H_{22}O_2$
MW: 270.37

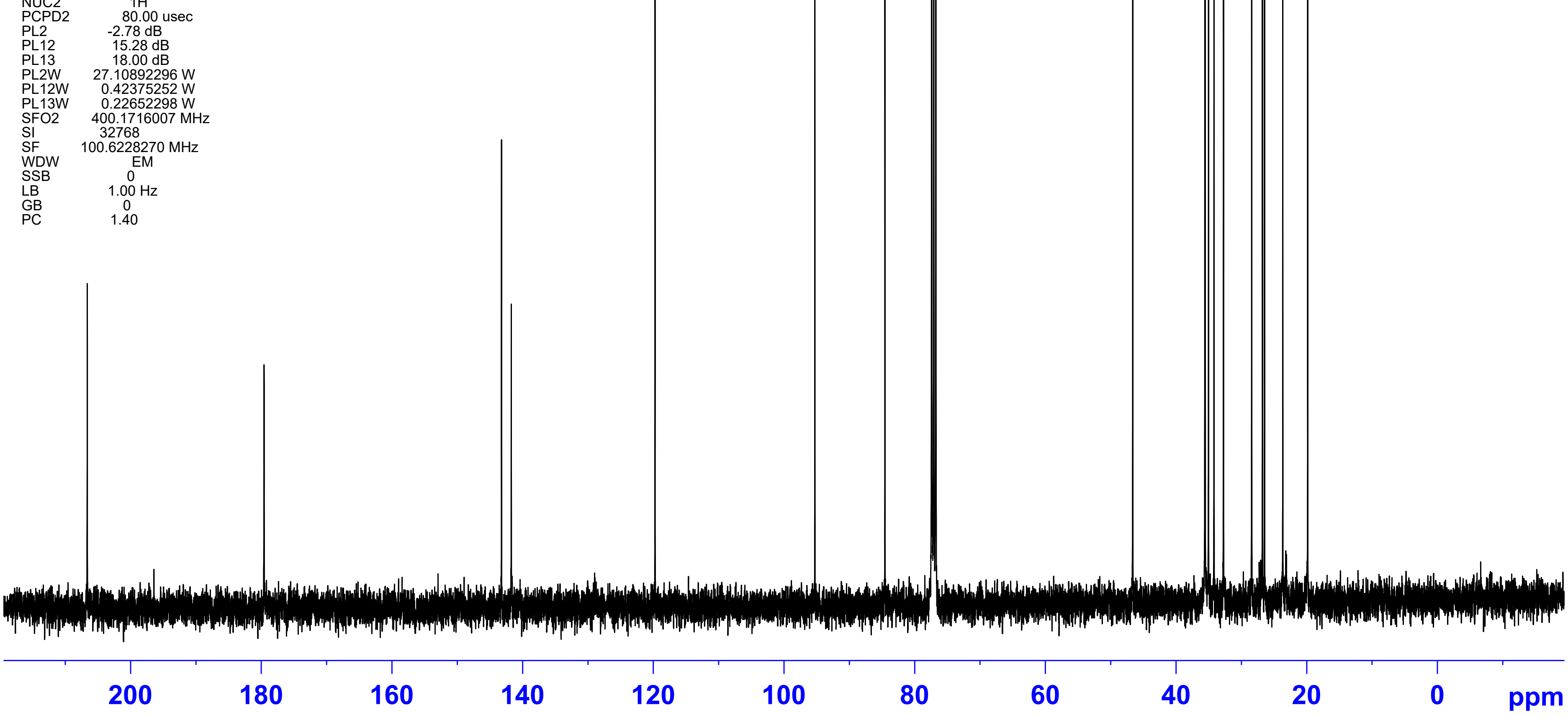

NAME AES 4062b_1H
EXPNO 1
PROCNO 1
Date_ 20170525
Time 0.58
INSTRUM spect
PROBHD 5 mm PABBO BB-
PULPROG zg30
TD 65536
SOLVENT CDCl3
NS 16
DS 2
SWH 8223.685 Hz
FIDRES 0.125483 Hz
AQ 3.9846387 sec
RG 144
DW 60.800 usec
DE 6.50 usec
TE 293.8 K
D1 1.00000000 sec
TD0 1

======== CHANNEL f1 ========
NUC1 1H
P1 10.00 usec
PL1 -2.78 dB
PL1W 27.10892296 W
SFO1 400.1724712 MHz
SI 32768
SF 400.1700091 MHz
WDW EM
SSB 0
LB 0.30 Hz
GB 0
PC 1.00

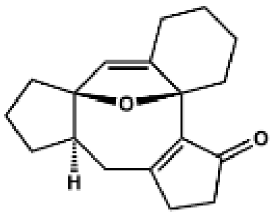

S14: $C_{18}H_{22}O_2$
MW: 270.37

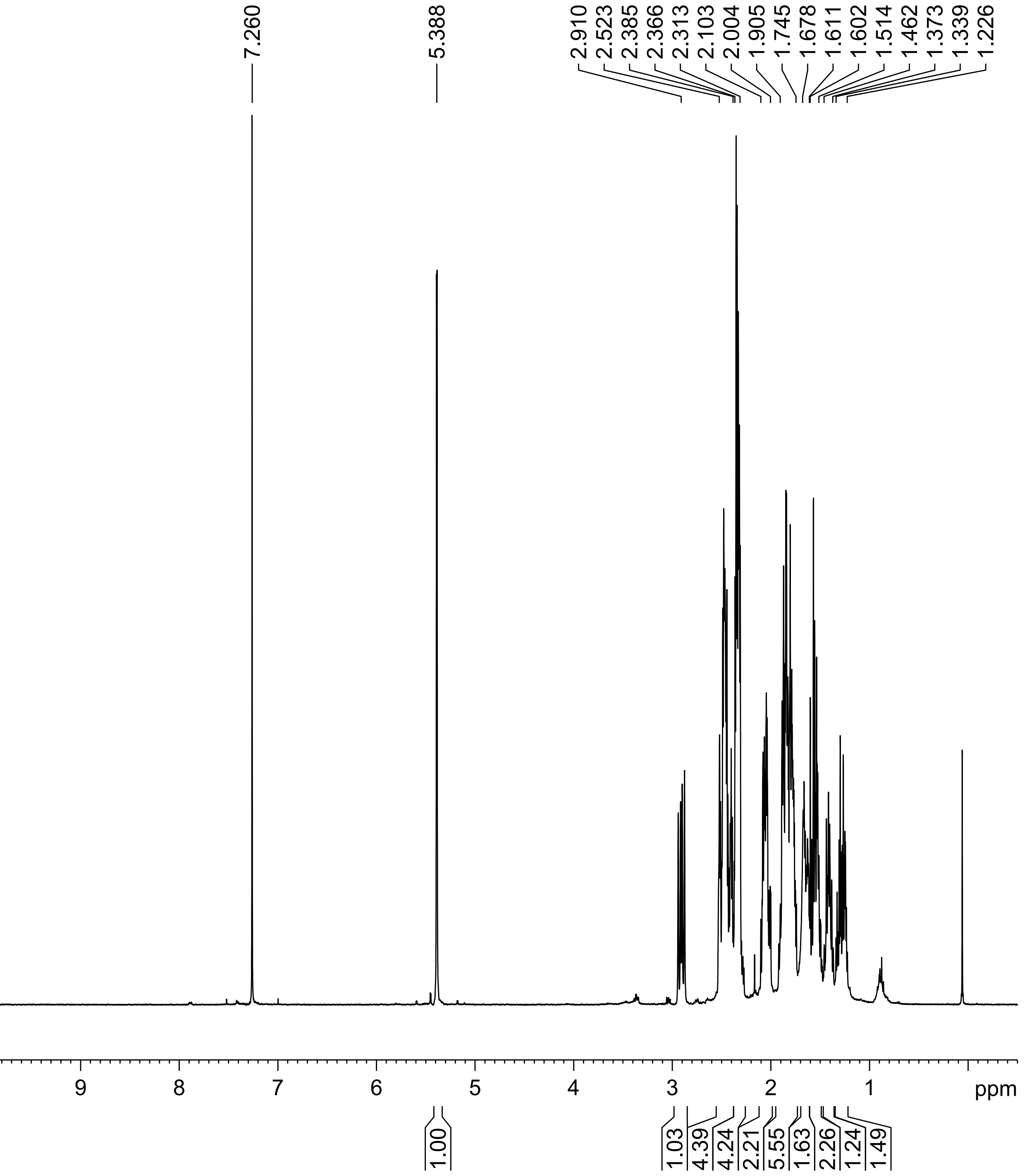

NAME AES 4062b C13
EXPNO 1
PROCNO 1
Date_ 20170523
Time 19.30
INSTRUM spect
PROBHD 5 mm PABBO BB-
PULPROG zgpg30
TD 65536
SOLVENT CDCl3
NS 1024
DS 4
SWH 36057.691 Hz
FIDRES 0.550197 Hz
AQ 0.9088159 sec
RG 203
DW 13.867 usec
DE 6.50 usec
TE 298.3 K
D1 2.00000000 sec
D11 0.03000000 sec
TD0 1

======== CHANNEL f1 ========
NUC1 13C
P1 9.50 usec
PL1 3.00 dB
PL1W 59.92485046 W
SFO1 150.9178988 MHz

======== CHANNEL f2 ========
CPDPRG2 waltz16
NUC2 1H
PCPD2 80.00 usec
PL2 -1.40 dB
PL12 14.38 dB
PL13 13.70 dB
PL2W 16.22728539 W
PL12W 0.42879117 W
PL13W 0.50147104 W
SFO2 600.1324005 MHz
SI 32768
SF 150.9028090 MHz
WDW EM
SSB 0
LB 1.00 Hz
GB 0
PC 1.40

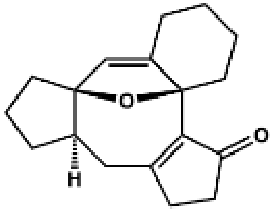

S14: $C_{18}H_{22}O_2$
MW: 270.37

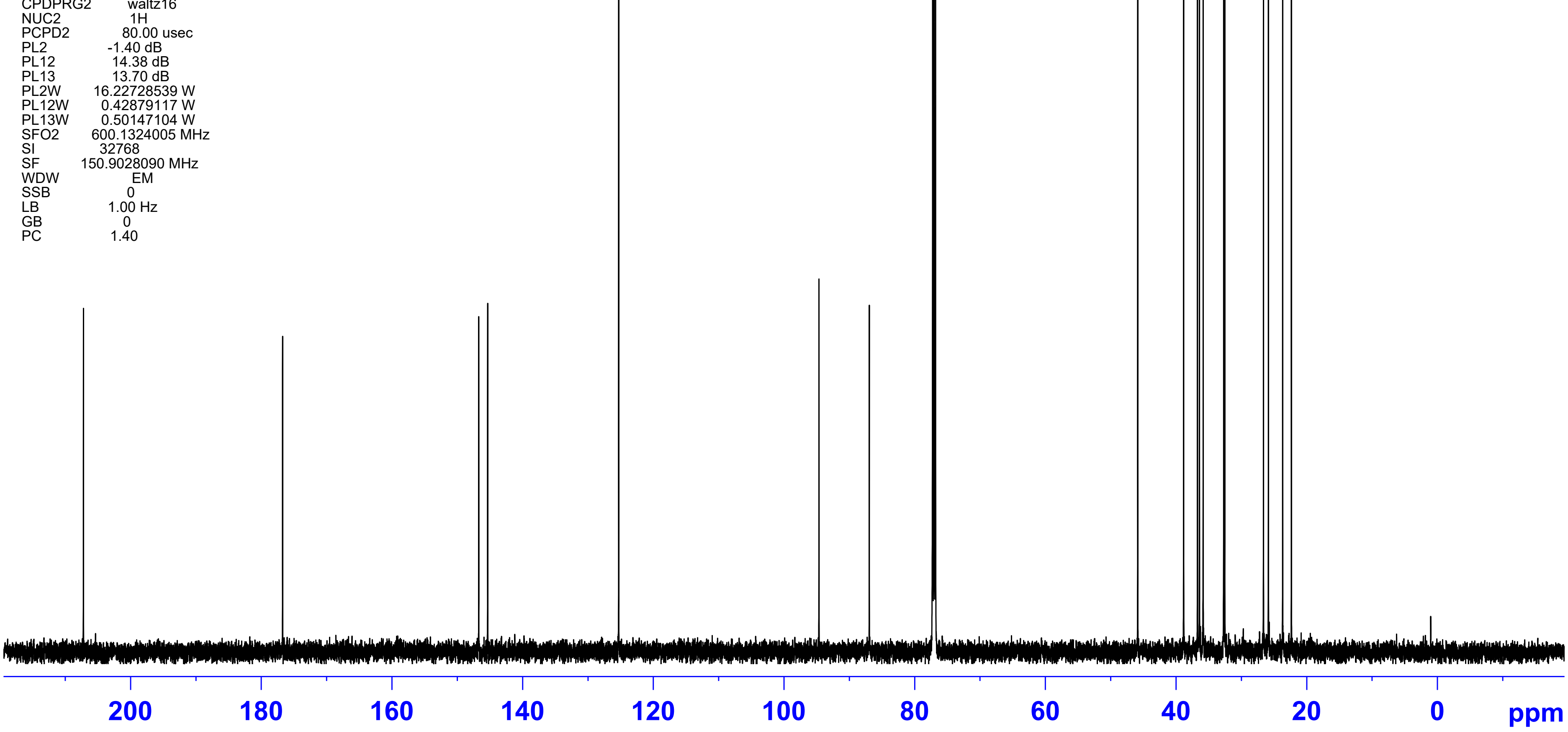

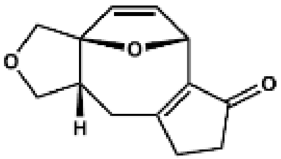

13h: $C_{13}H_{14}O_3$
MW: 218.25

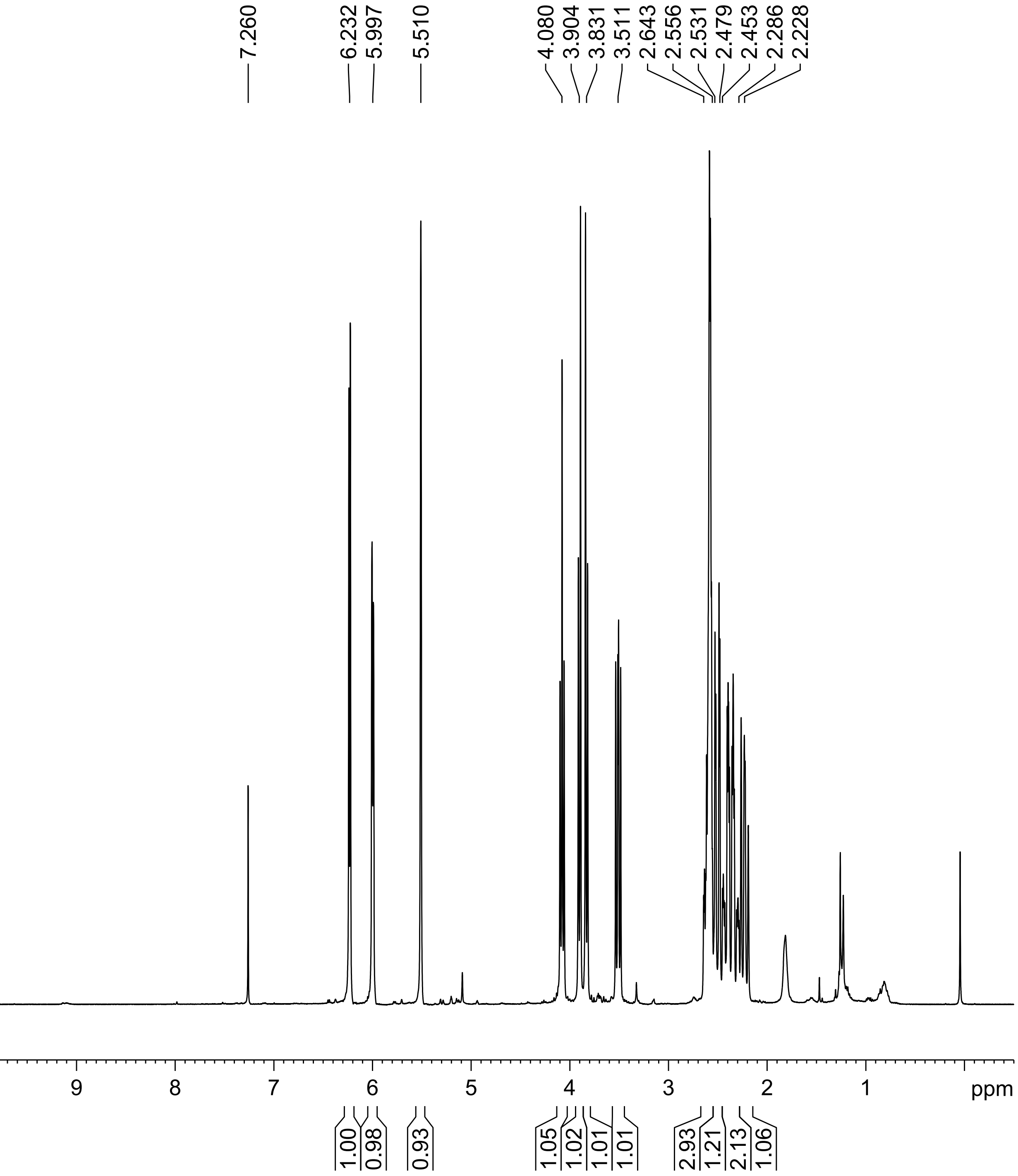

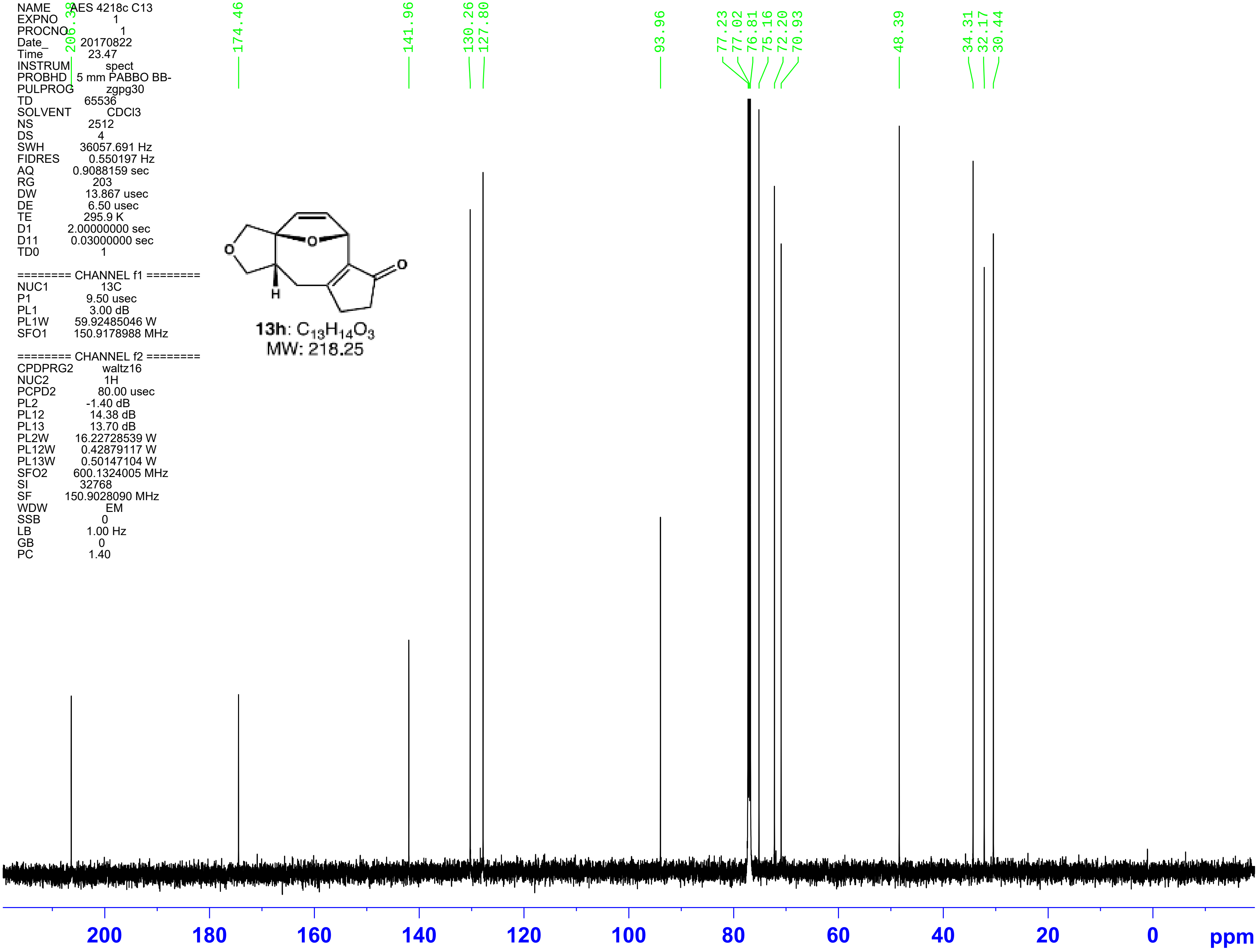

NAME AES 4218c C13
EXPNO 1
PROCNO 1
Date_ 20170822
Time 23.47
INSTRUM spect
PROBHD 5 mm PABBO BB-
PULPROG zgpg30
TD 65536
SOLVENT CDCl3
NS 2512
DS 4
SWH 36057.691 Hz
FIDRES 0.550197 Hz
AQ 0.9088159 sec
RG 203
DW 13.867 usec
DE 6.50 usec
TE 295.9 K
D1 2.00000000 sec
D11 0.03000000 sec
TD0 1

======== CHANNEL f1 ========
NUC1 13C
P1 9.50 usec
PL1 3.00 dB
PL1W 59.92485046 W
SFO1 150.9178988 MHz

======== CHANNEL f2 ========
CPDPRG2 waltz16
NUC2 1H
PCPD2 80.00 usec
PL2 -1.40 dB
PL12 14.38 dB
PL13 13.70 dB
PL2W 16.22728539 W
PL12W 0.42879117 W
PL13W 0.50147104 W
SFO2 600.1324005 MHz
SI 32768
SF 150.9028090 MHz
WDW EM
SSB 0
LB 1.00 Hz
GB 0
PC 1.40

13h: $C_{13}H_{14}O_3$
MW: 218.25

NAME AES 5160 1H
EXPNO 1
PROCNO 1
Date_ 20180103
Time 16.09
INSTRUM spect
PROBHD 5 mm PABBO BB-
PULPROG zg30
TD 65536
SOLVENT CDCl3
NS 16
DS 2
SWH 12335.526 Hz
FIDRES 0.188225 Hz
AQ 2.6564426 sec
RG 114
DW 40.533 usec
DE 6.50 usec
TE 298.2 K
D1 1.00000000 sec
TD0 1

======== CHANNEL f1 ========
NUC1 1H
P1 13.00 usec
PL1 -1.40 dB
PL1W 16.22728539 W
SFO1 600.1337060 MHz
SI 32768
SF 600.1300167 MHz
WDW EM
SSB 0
LB 0.30 Hz
GB 0
PC 1.00

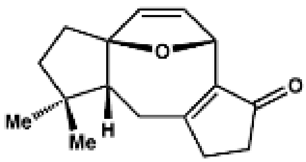

13i: $C_{16}H_{20}O_2$
MW: 244.33

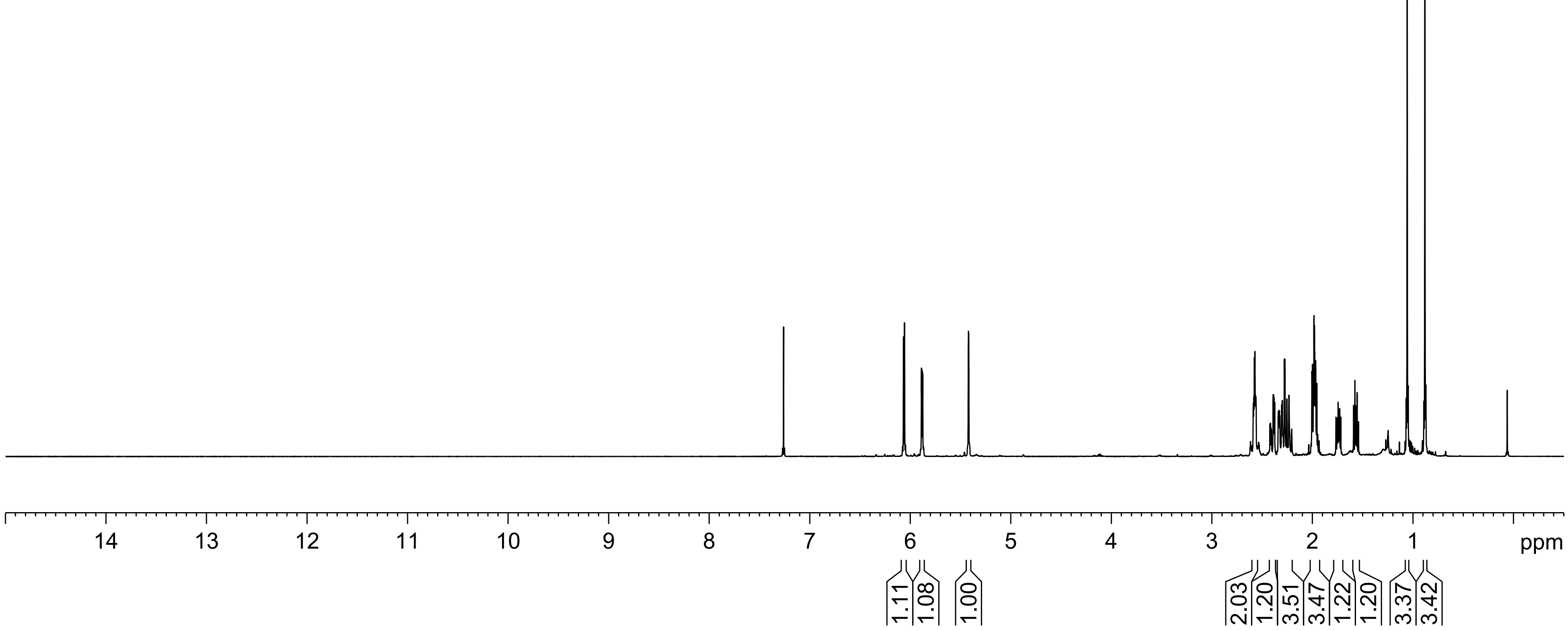

NAME AES 5160 C13
EXPNO 1
PROCNO 1
Date_ 20180103
Time 17.45
INSTRUM spect
PROBHD 5 mm PABBO BB-
PULPROG zgpg30
TD 65536
SOLVENT CDCl3
NS 2625
DS 4
SWH 36057.691 Hz
FIDRES 0.550197 Hz
AQ 0.9088159 sec
RG 203
DW 13.867 usec
DE 6.50 usec
TE 298.2 K
D1 2.00000000 sec
D11 0.03000000 sec
TD0 1

======== CHANNEL f1 ========
NUC1 13C
P1 9.50 usec
PL1 3.00 dB
PL1W 59.92485046 W
SFO1 150.9178988 MHz

======== CHANNEL f2 ========
CPDPRG2 waltz16
NUC2 1H
PCPD2 80.00 usec
PL2 -1.40 dB
PL12 14.38 dB
PL13 13.70 dB
PL2W 16.22728539 W
PL12W 0.42879117 W
PL13W 0.50147104 W
SFO2 600.1324005 MHz
SI 32768
SF 150.9028090 MHz
WDW EM
SSB 0
LB 1.00 Hz
GB 0
PC 1.40

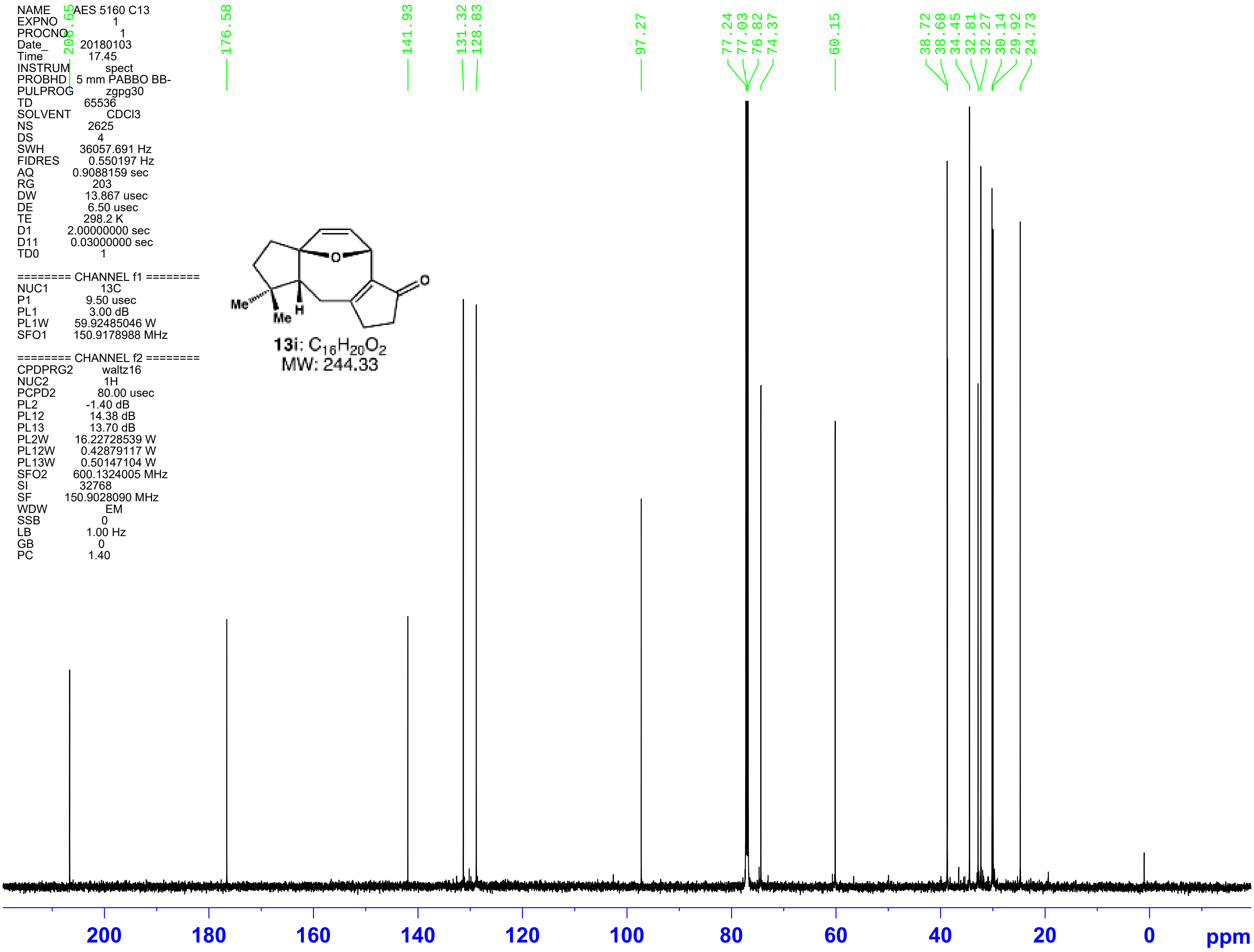

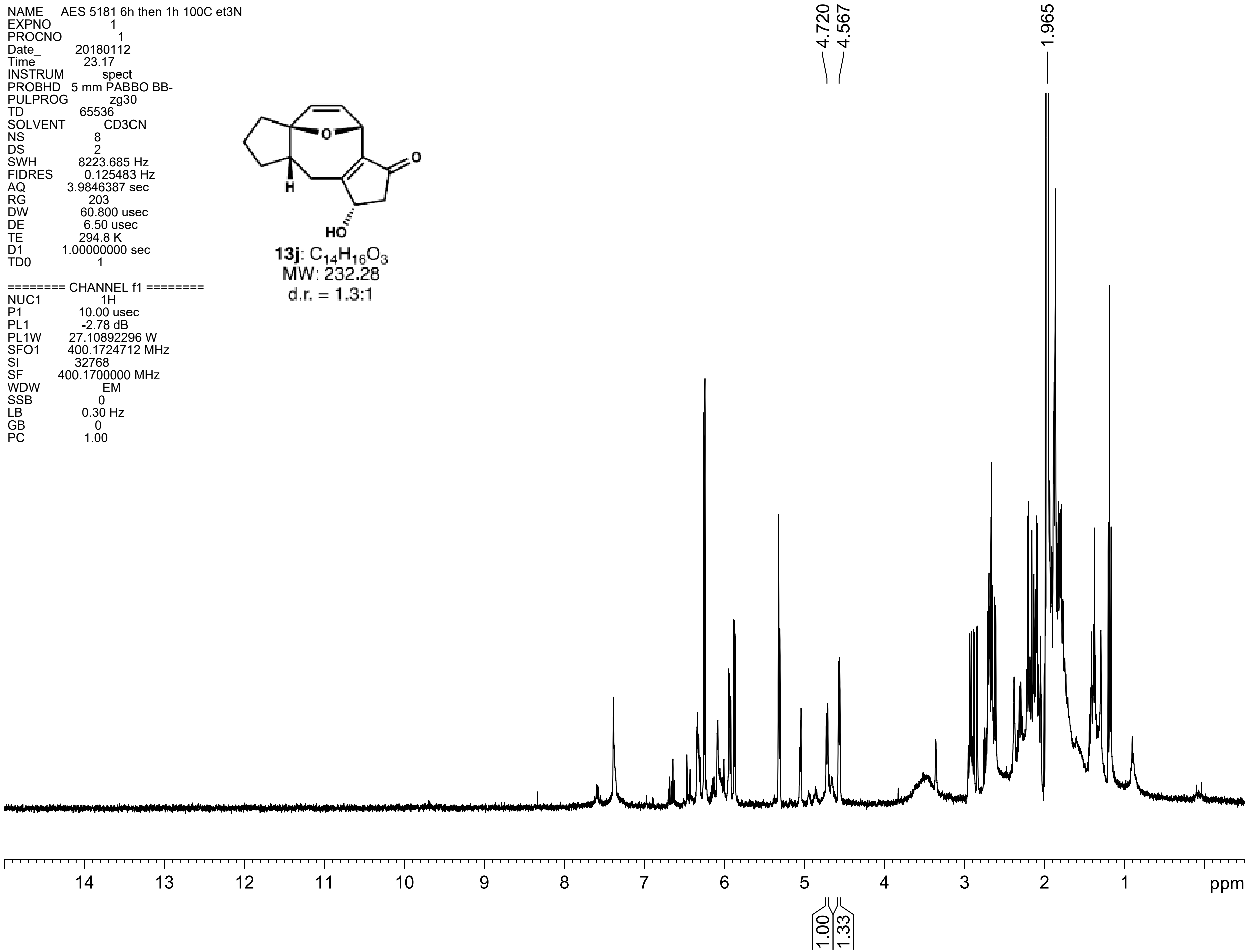

13j: $C_{14}H_{16}O_3$
MW: 232.28
d.r. = 1.3:1

NAME AES 5155c
EXPNO 2
PROCNO 1
Date_ 20171218
Time 16.37
INSTRUM spect
PROBHD 5 mm PABBO BB-
PULPROG zg30
TD 65536
SOLVENT CDCl3
NS 8
DS 2
SWH 8223.685 Hz
FIDRES 0.125483 Hz
AQ 3.9846387 sec
RG 57
DW 60.800 usec
DE 6.50 usec
TE 294.8 K
D1 1.00000000 sec
TD0 1

======== CHANNEL f1 ========
NUC1 1H
P1 10.00 usec
PL1 -2.78 dB
PL1W 27.10892296 W
SFO1 400.1724712 MHz
SI 32768
SF 400.1700083 MHz
WDW EM
SSB 0
LB 0.30 Hz
GB 0
PC 1.00

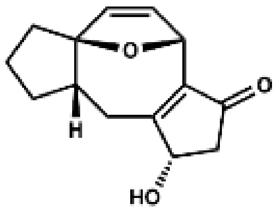

13j: $C_{14}H_{16}O_{3}$
MW: 232.28
d.r. = 1.3:1

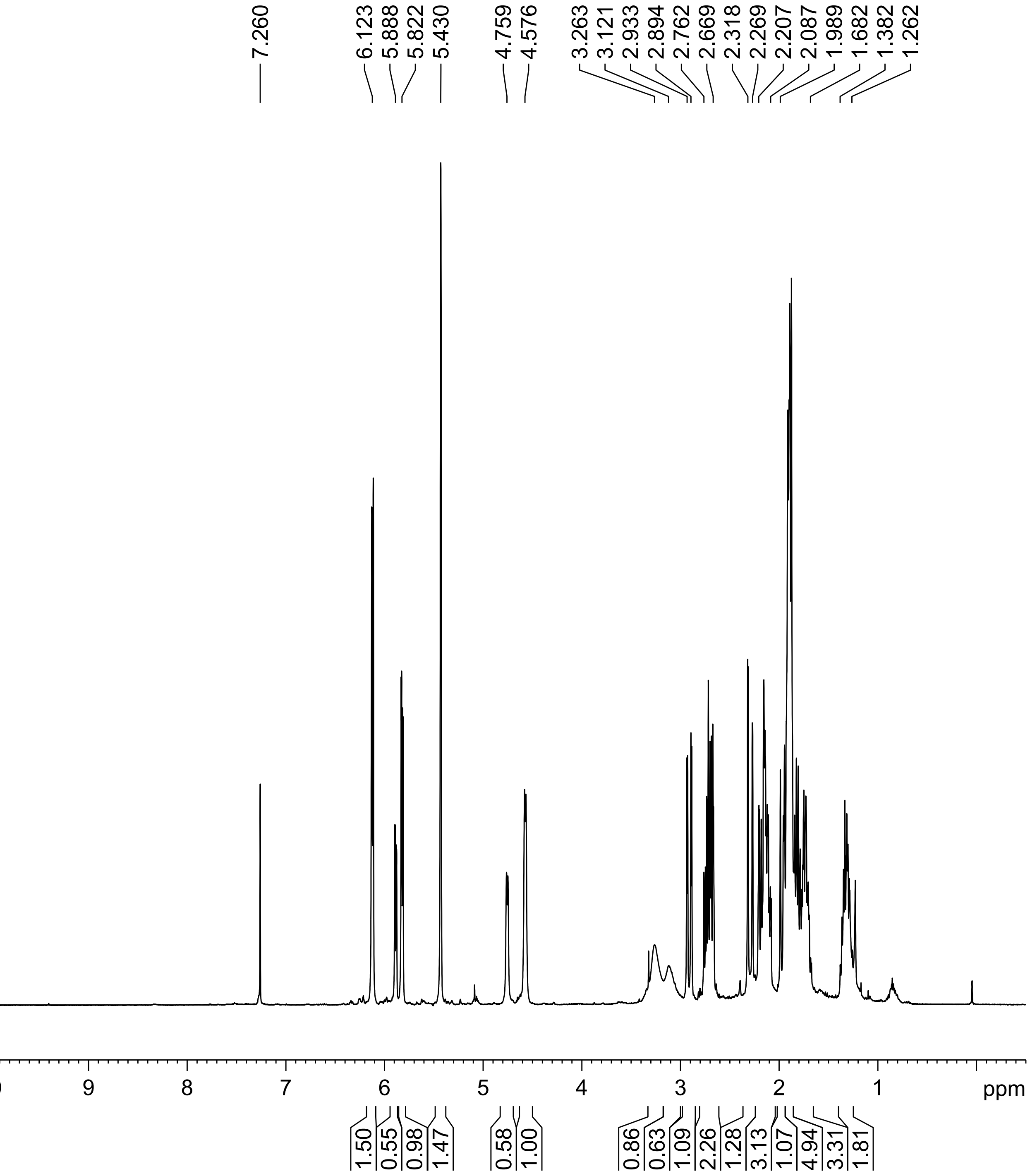

NAME AES 5151c C13
EXPNO 1
PROCNO 1
Date_ 20171216
Time 11.25
INSTRUM spect
PROBHD 5 mm PABBO BB-
PULPROG zgpg30
TD 65536
SOLVENT CDCl3
NS 2994
DS 4
SWH 39062.500 Hz
FIDRES 0.596046 Hz
AQ 0.8389108 sec
RG 203
DW 12.800 usec
DE 6.50 usec
TE 298.3 K
D1 2.00000000 sec
D11 0.03000000 sec
TD0 1

======== CHANNEL f1 ========
NUC1 13C
P1 9.50 usec
PL1 3.00 dB
PL1W 59.92485046 W
SFO1 150.9178988 MHz

======== CHANNEL f2 ========
CPDPRG2 waltz16
NUC2 1H
PCPD2 80.00 usec
PL2 -1.40 dB
PL12 14.38 dB
PL13 13.70 dB
PL2W 16.22728539 W
PL12W 0.42879117 W
PL13W 0.50147104 W
SFO2 600.1324005 MHz
SI 32768
SF 150.9028090 MHz
WDW EM
SSB 0
LB 1.00 Hz
GB 0
PC 1.40

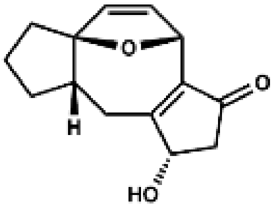

13j: $C_{14}H_{16}O_3$
MW: 232.28
d.r. = 1.3:1

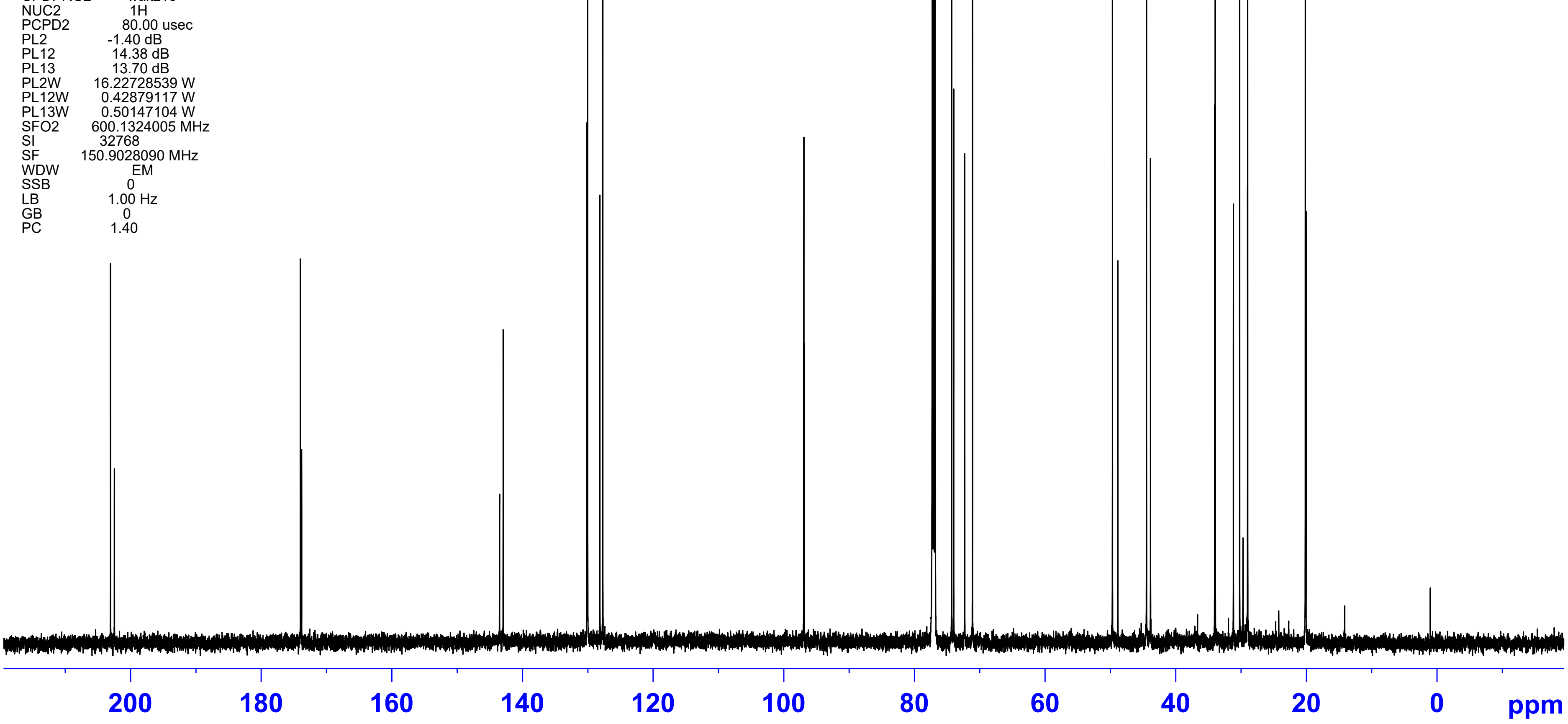

NAME AES 2288 1H
EXPNO 1
PROCNO 1
Date_ 20160721
Time 19.27
INSTRUM spect
PROBHD 5 mm PABBO BB-
PULPROG zg30
TD 65536
SOLVENT CDCl3
NS 16
DS 2
SWH 12335.526 Hz
FIDRES 0.188225 Hz
AQ 2.6564426 sec
RG 64
DW 40.533 usec
DE 6.50 usec
TE 294.8 K
D1 1.00000000 sec
TD0 1

======== CHANNEL f1 ========
NUC1 1H
P1 14.00 usec
PL1 -1.40 dB
PL1W 16.22728539 W
SFO1 600.1337060 MHz
SI 32768
SF 600.1300167 MHz
WDW EM
SSB 0
LB 0.30 Hz
GB 0
PC 1.00

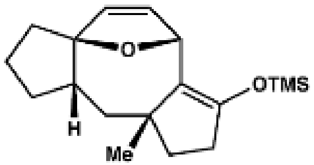

14: $C_{18}H_{28}O_2Si$
MW: 304.51

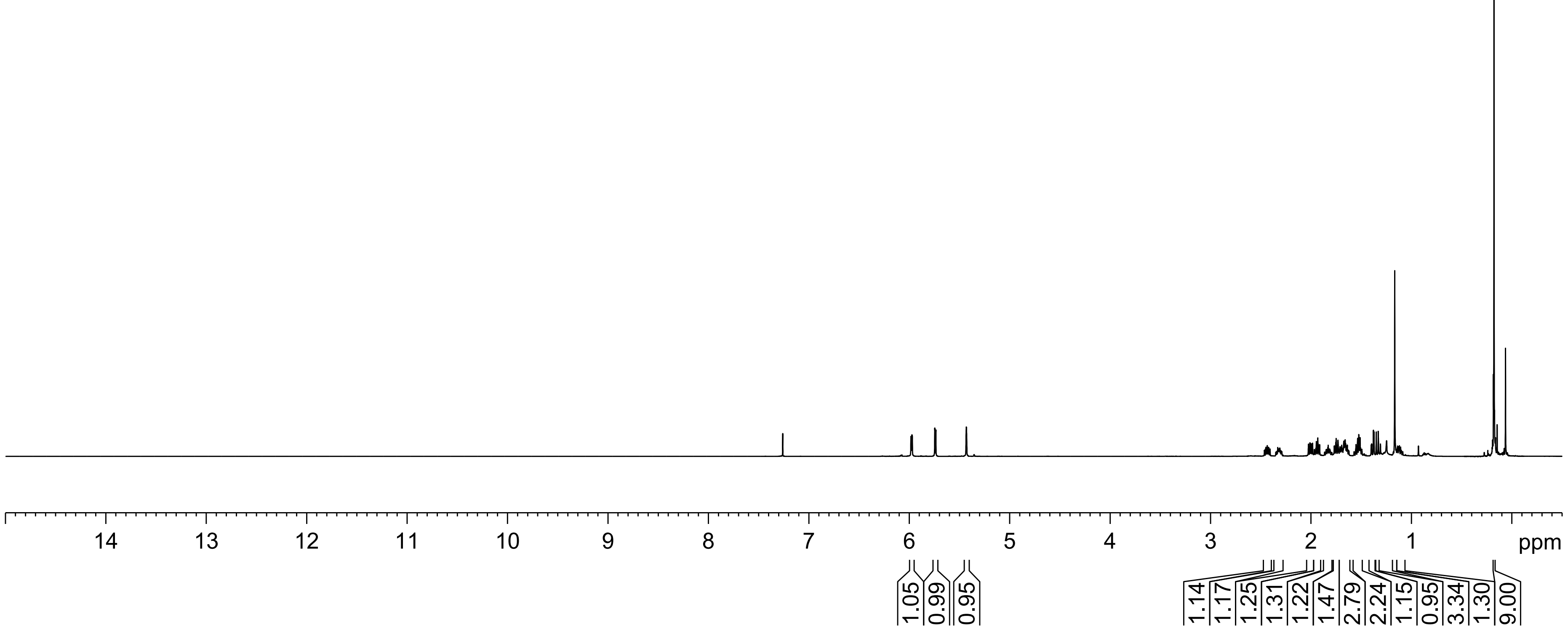

NAME AES 2288 C13
EXPNO 1
PROCNO 1
Date_ 20160721
Time 20.21
INSTRUM spect
PROBHD 5 mm PABBO BB-
PULPROG zgpg30
TD 65536
SOLVENT CDCl3
NS 1024
DS 4
SWH 39062.500 Hz
FIDRES 0.596046 Hz
AQ 0.8389108 sec
RG 203
DW 12.800 usec
DE 6.50 usec
TE 298.1 K
D1 2.00000000 sec
D11 0.03000000 sec
TD0 1

======== CHANNEL f1 ========
NUC1 13C
P1 9.50 usec
PL1 3.00 dB
PL1W 59.92485046 W
SFO1 150.9178988 MHz

======== CHANNEL f2 ========
CPDPRG2 waltz16
NUC2 1H
PCPD2 80.00 usec
PL2 -1.40 dB
PL12 13.74 dB
PL13 13.70 dB
PL2W 16.22728539 W
PL12W 0.49687356 W
PL13W 0.50147104 W
SFO2 600.1324005 MHz
SI 32768
SF 150.9028090 MHz
WDW EM
SSB 0
LB 1.00 Hz
GB 0
PC 1.40

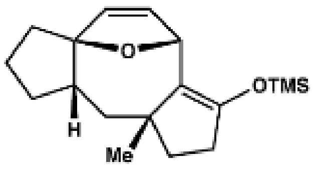

14: $C_{18}H_{28}O_2Si$
MW: 304.51

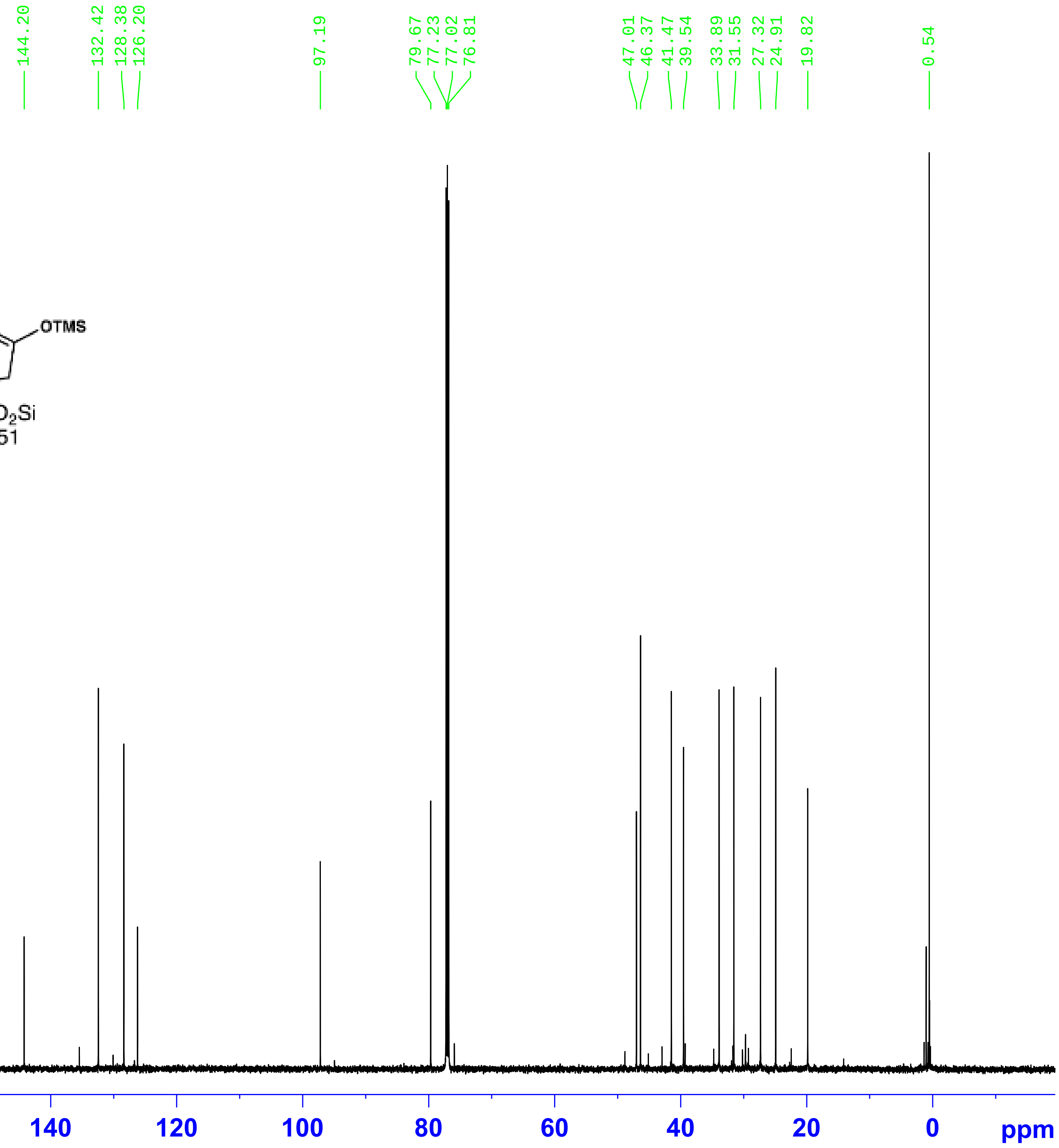

NAME AES 2267 1H
EXPNO 1
PROCNO 1
Date_ 20160706
Time 20.53
INSTRUM spect
PROBHD 5 mm PABBO BB-
PULPROG zg30
TD 65536
SOLVENT CDCl3
NS 16
DS 2
SWH 12335.526 Hz
FIDRES 0.188225 Hz
AQ 2.6564426 sec
RG 57
DW 40.533 usec
DE 6.50 usec
TE 293.1 K
D1 1.00000000 sec
TD0 1

======== CHANNEL f1 ========
NUC1 1H
P1 14.00 usec
PL1 -1.40 dB
PL1W 16.22728539 W
SFO1 600.1337060 MHz
SI 32768
SF 600.1300168 MHz
WDW EM
SSB 0
LB 0.30 Hz
GB 0
PC 1.00

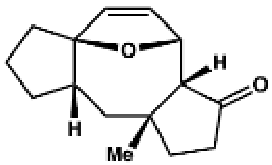

15: $C_{15}H_{20}O_2$
MW: 232.32

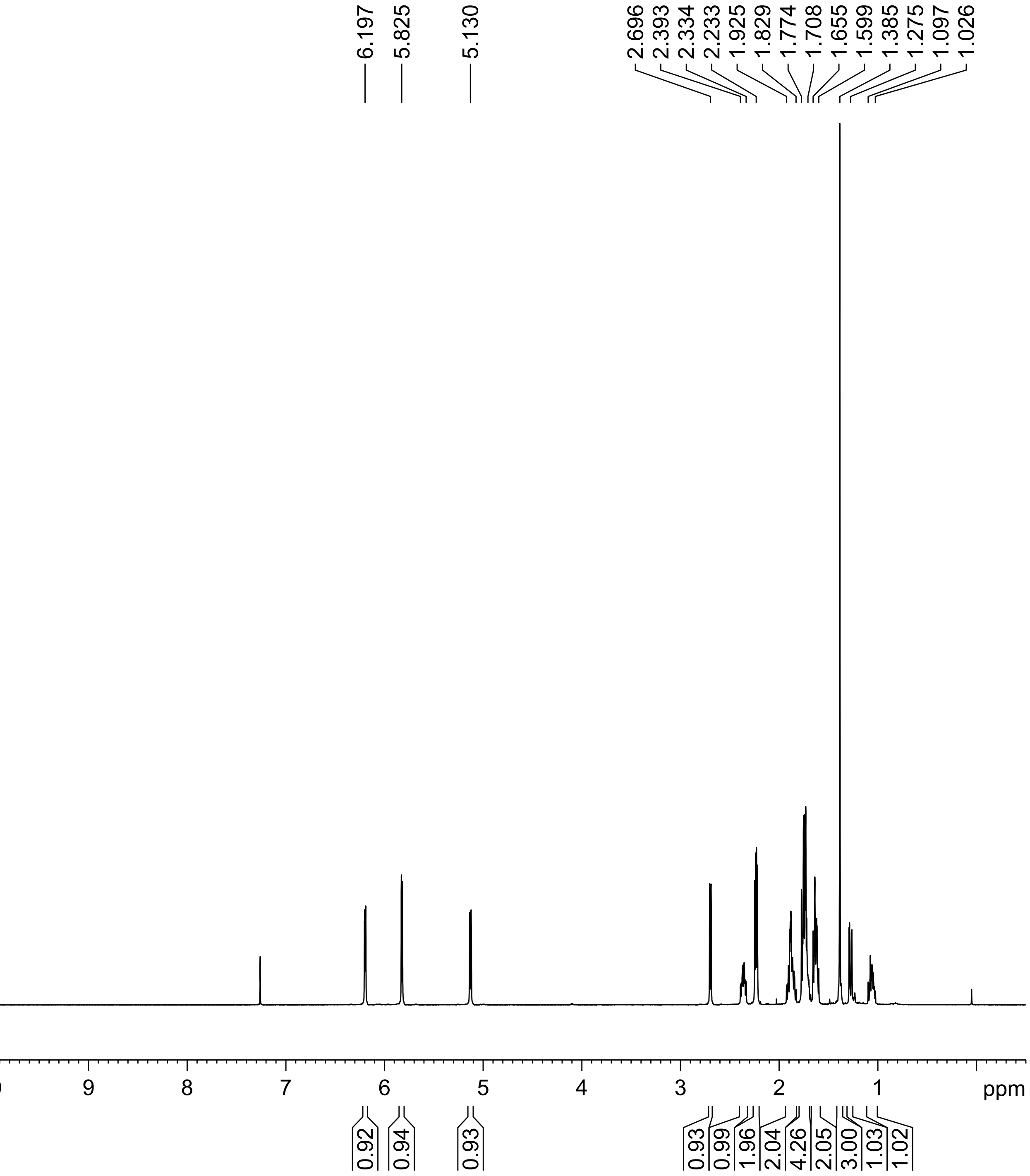

NAME AES 2267 C13
EXPNO 4
PROCNO 1
Date_ 20160706
Time 23.16
INSTRUM spect
PROBHD 5 mm PABBO BB-
PULPROG zgpg30
TD 65536
SOLVENT CDCl3
NS 1024
DS 4
SWH 39062.500 Hz
FIDRES 0.596046 Hz
AQ 0.8389108 sec
RG 203
DW 12.800 usec
DE 6.50 usec
TE 296.7 K
D1 2.00000000 sec
D11 0.03000000 sec
TD0 1

======== CHANNEL f1 ========
NUC1 13C
P1 9.50 usec
PL1 3.00 dB
PL1W 59.92485046 W
SFO1 150.9178988 MHz

======== CHANNEL f2 ========
CPDPRG2 waltz16
NUC2 1H
PCPD2 80.00 usec
PL2 -1.40 dB
PL12 13.74 dB
PL13 13.70 dB
PL2W 16.22728539 W
PL12W 0.49687356 W
PL13W 0.50147104 W
SFO2 600.1324005 MHz
SI 32768
SF 150.9028090 MHz
WDW EM
SSB 0
LB 1.00 Hz
GB 0
PC 1.40

15: $C_{15}H_{20}O_2$
MW: 232.32

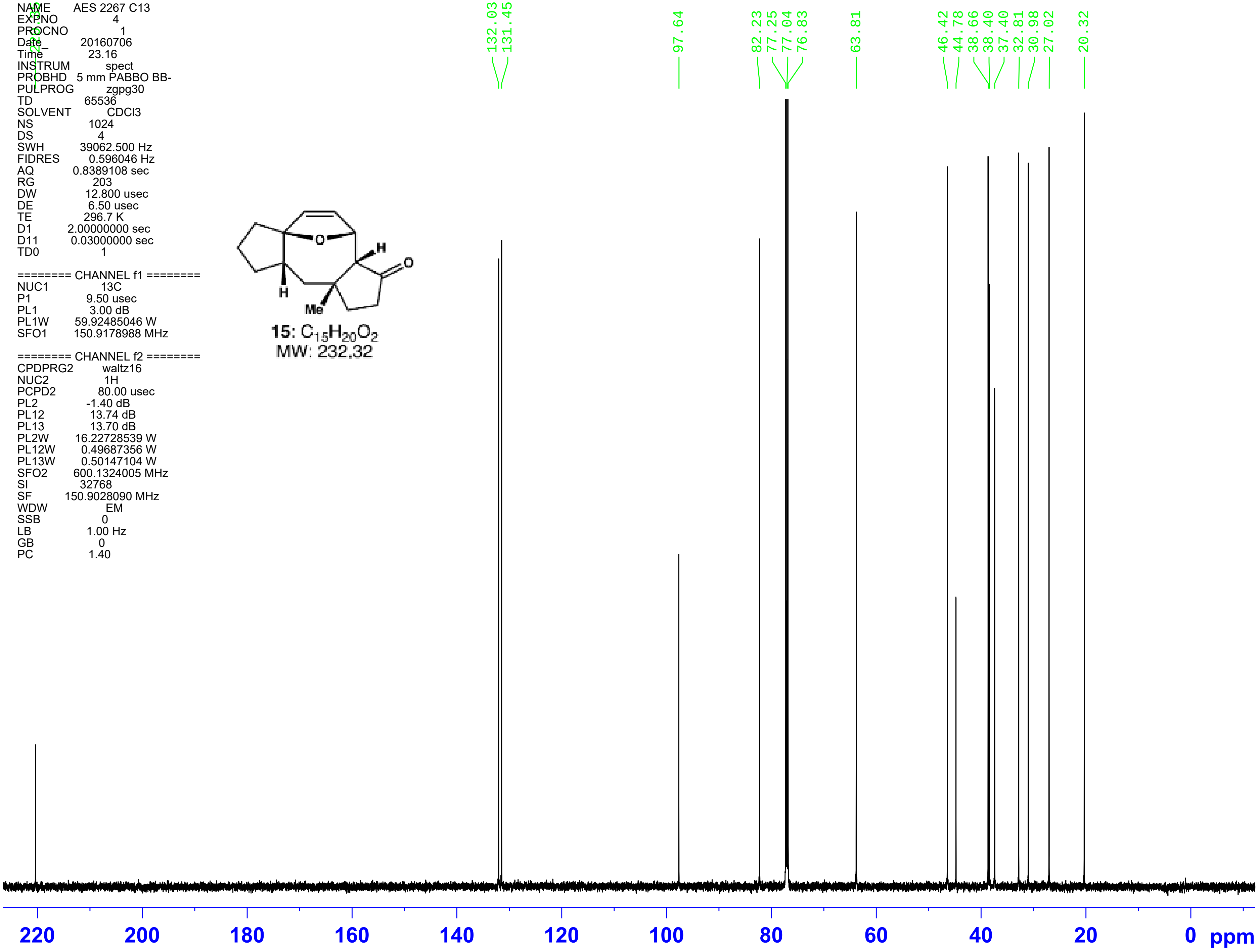

NAME AES 3009 1H
EXPNO 1
PROCNO 1
Date_ 20160814
Time 18.13
INSTRUM spect
PROBHD 5 mm PABBO BB-
PULPROG zg30
TD 65536
SOLVENT CDCl3
NS 16
DS 2
SWH 12335.526 Hz
FIDRES 0.188225 Hz
AQ 2.6564426 sec
RG 101
DW 40.533 usec
DE 6.50 usec
TE 294.5 K
D1 1.00000000 sec
TD0 1

======== CHANNEL f1 ========
NUC1 1H
P1 14.00 usec
PL1 -1.40 dB
PL1W 16.22728539 W
SFO1 600.1337060 MHz
SI 32768
SF 600.1300164 MHz
WDW EM
SSB 0
LB 0.30 Hz
GB 0
PC 1.00

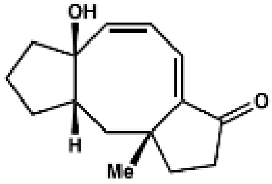

16: $C_{15}H_{20}O_2$
MW: 232.32

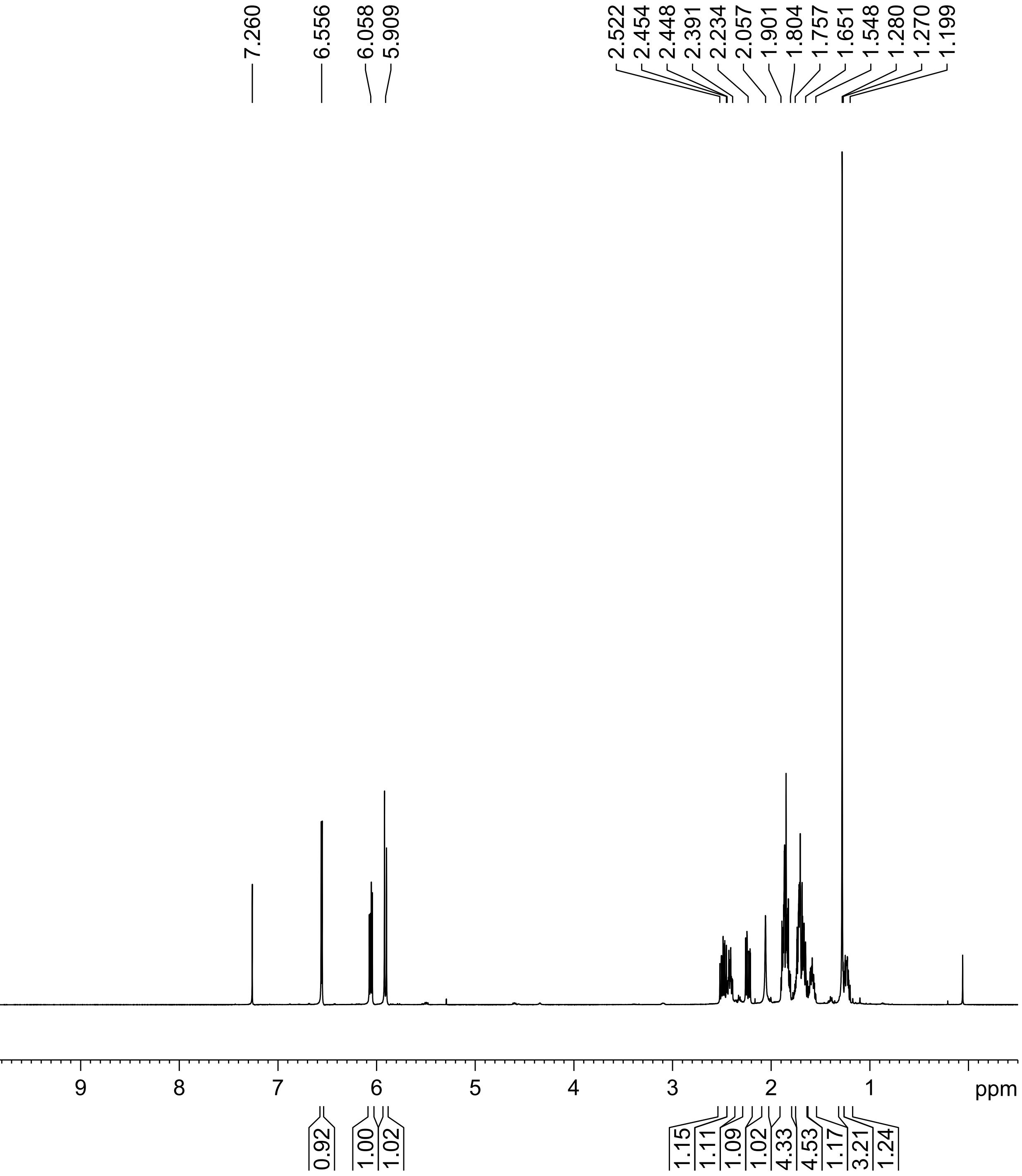

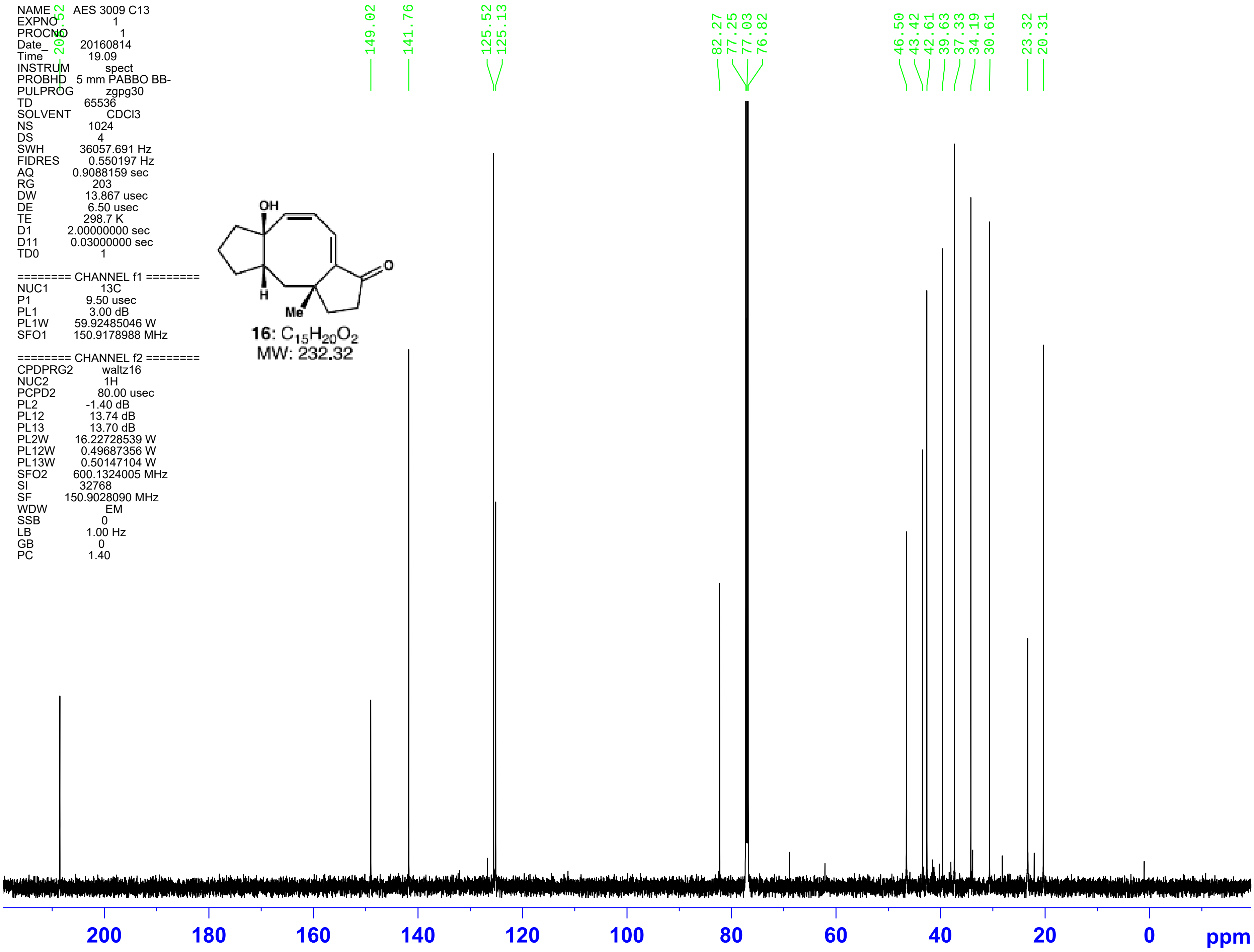
NAME AES 3009 C13
EXPNO 1
PROCNO 1
Date_ 20160814
Time 19.09
INSTRUM spect
PROBHD 5 mm PABBO BB-
PULPROG zgpg30
TD 65536
SOLVENT CDCl3
NS 1024
DS 4
SWH 36057.691 Hz
FIDRES 0.550197 Hz
AQ 0.9088159 sec
RG 203
DW 13.867 usec
DE 6.50 usec
TE 298.7 K
D1 2.00000000 sec
D11 0.03000000 sec
TD0 1
======== CHANNEL f1 ========
NUC1 13C
P1 9.50 usec
PL1 3.00 dB
PL1W 59.92485046 W
SFO1 150.9178988 MHz
======== CHANNEL f2 ========
CPDPRG2 waltz16
NUC2 1H
PCPD2 80.00 usec
PL2 -1.40 dB
PL12 13.74 dB
PL13 13.70 dB
PL2W 16.22728539 W
PL12W 0.49687356 W
PL13W 0.50147104 W
SFO2 600.1324005 MHz
SI 32768
SF 150.9028090 MHz
WDW EM
SSB 0
LB 1.00 Hz
GB 0
PC 1.40
OH
H
Me
O
16: C15H20O2
MW: 232.32
208.52
149.02
141.76
125.52
125.13
82.27
77.25
77.03
76.82
46.50
43.42
42.61
39.63
37.33
34.19
30.61
23.32
20.31
200
180
160
140
120
100
80
60
40
20
0
ppm

NAME JAL3209ii crude 1H
EXPNO 1
PROCNO 1
Date_ 20180103
Time 14.48
INSTRUM spect
PROBHD 5 mm PABBO BB-
PULPROG zg30
TD 65536
SOLVENT C6D6
NS 8
DS 2
SWH 8223.685 Hz
FIDRES 0.125483 Hz
AQ 3.9846387 sec
RG 203
DW 60.800 usec
DE 6.50 usec
TE 294.3 K
D1 1.00000000 sec
TD0 1

======== CHANNEL f1 ========
NUC1 1H
P1 10.00 usec
PL1 -2.78 dB
PL1W 27.10892296 W
SFO1 400.1724712 MHz
SI 32768
SF 400.1700001 MHz
WDW EM
SSB 0
LB 0.30 Hz
GB 0
PC 1.00

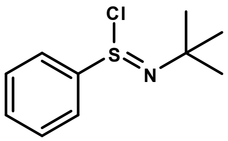

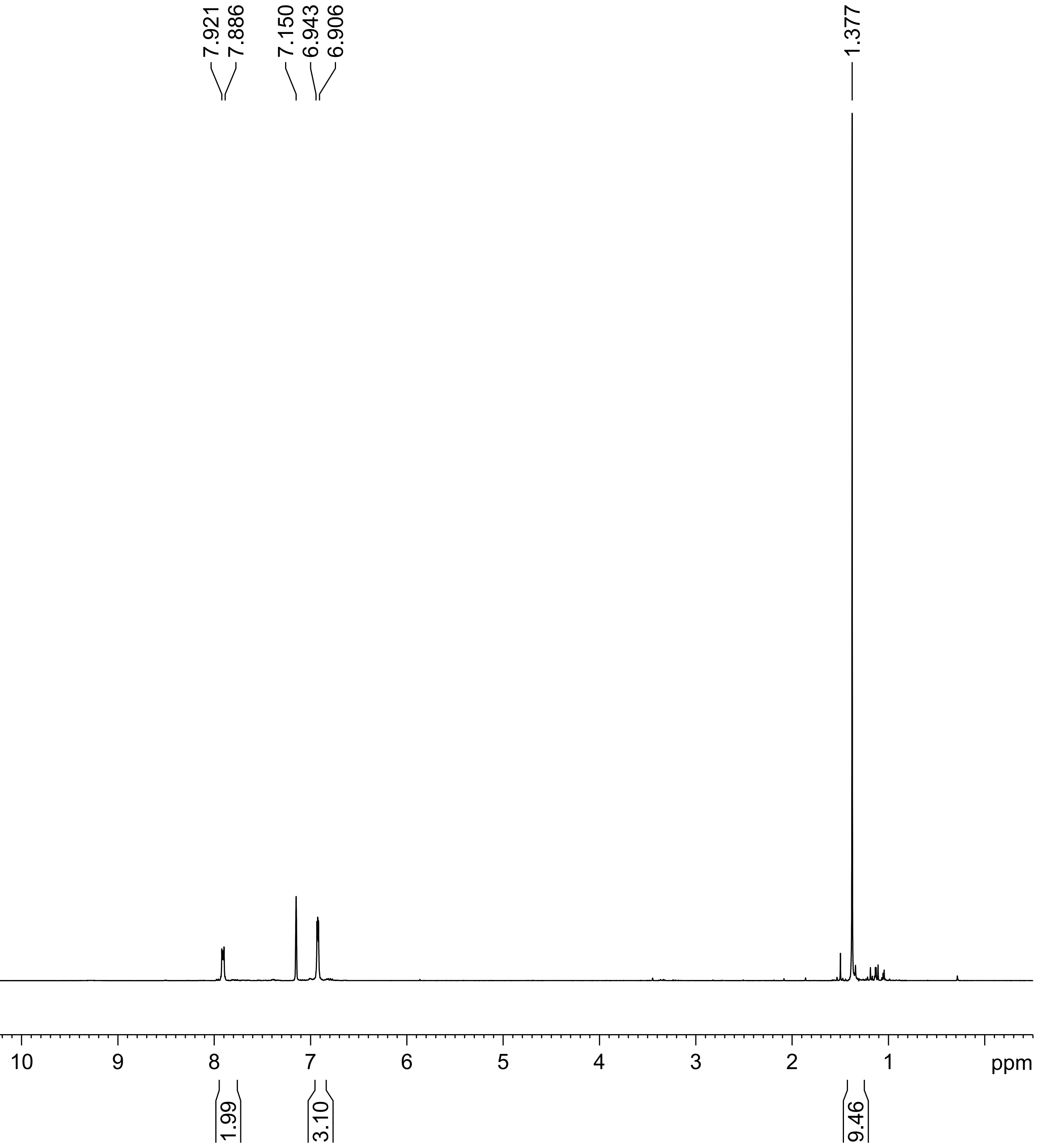

Supplement: Supplementary file 1 [file SC-009-C8SC00999F-s001.pdf]
